# Regioselective and Stereodivergent C–H Functionalization of Internal Alkenes

**DOI:** 10.1021/jacs.6c17366

**Published:** 2026-10-15

**Authors:** Elizabeth A. Ryutov, Peter J. Verardi, Song N. Dang, Karina Targos, Leah C. Garman, Zachary K. Wickens

**Affiliations:** Department of Chemistry, 5228 University of Wisconsin-Madison, Madison, Wisconsin 53706, United States

## Abstract

Herein, we introduce a new strategy for the regioselective and stereodivergent C–H functionalization of internal alkenes. We discovered that the regioselectivity of internal alkene thianthrenation is exceptionally electronically sensitive and can be controlled by native allylic and homoallylic functional groups. We also found that both alkenyl thianthrenium stereoisomers can be selectively accessed by using different workup conditions. While thianthrenation is typically stereoretentive, the addition of Cs_2_CO_3_ in the workup induces isomerization through reversible conjugate addition. We show that both stereoisomers of the alkenyl linchpins can be readily engaged in stereoretentive cross coupling reactions. This selective thianthrenation-coupling sequence introduces a generalizable platform for stereodefined trisubstituted alkene synthesis.

## Graphical abstract

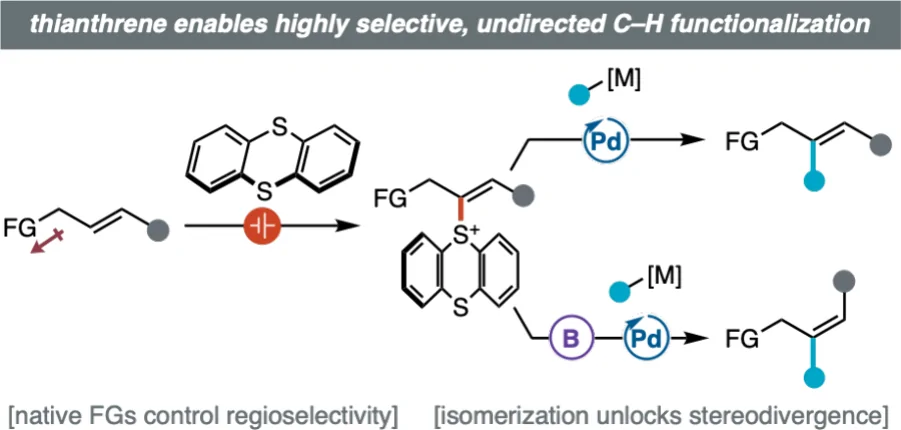

The regio- and stereoselective synthesis of alkenes is a cornerstone of synthetic methodology. Decades of research in this area have enabled facile access to mono- and disubstituted alkenes.^1^ However, more-substituted alkenes remain notoriously challenging to prepare (Scheme 1A). Alkyne semihydrogenation, a reliable method for disubstituted alkene synthesis, is fundamentally precluded for alkenes bearing three or more substituents.^2^ While carbonyl olefination can provide multisubstituted alkenes in some cases, these methods become increasingly inefficient and poorly stereoselective for trisubstituted alkene synthesis.^3^ Such alkenes are instead typically prepared by hydrometalation of activated alkynes (e.g., aryl alkynes).^4–7^ As a result, the development of new strategies to access trisubstituted alkenes remains a topic of contemporary interest, with exciting recent progress leveraging boronate chemistry,^8^ photochemistry,^9^ and olefin metathesis.^10^

**Scheme 1:**
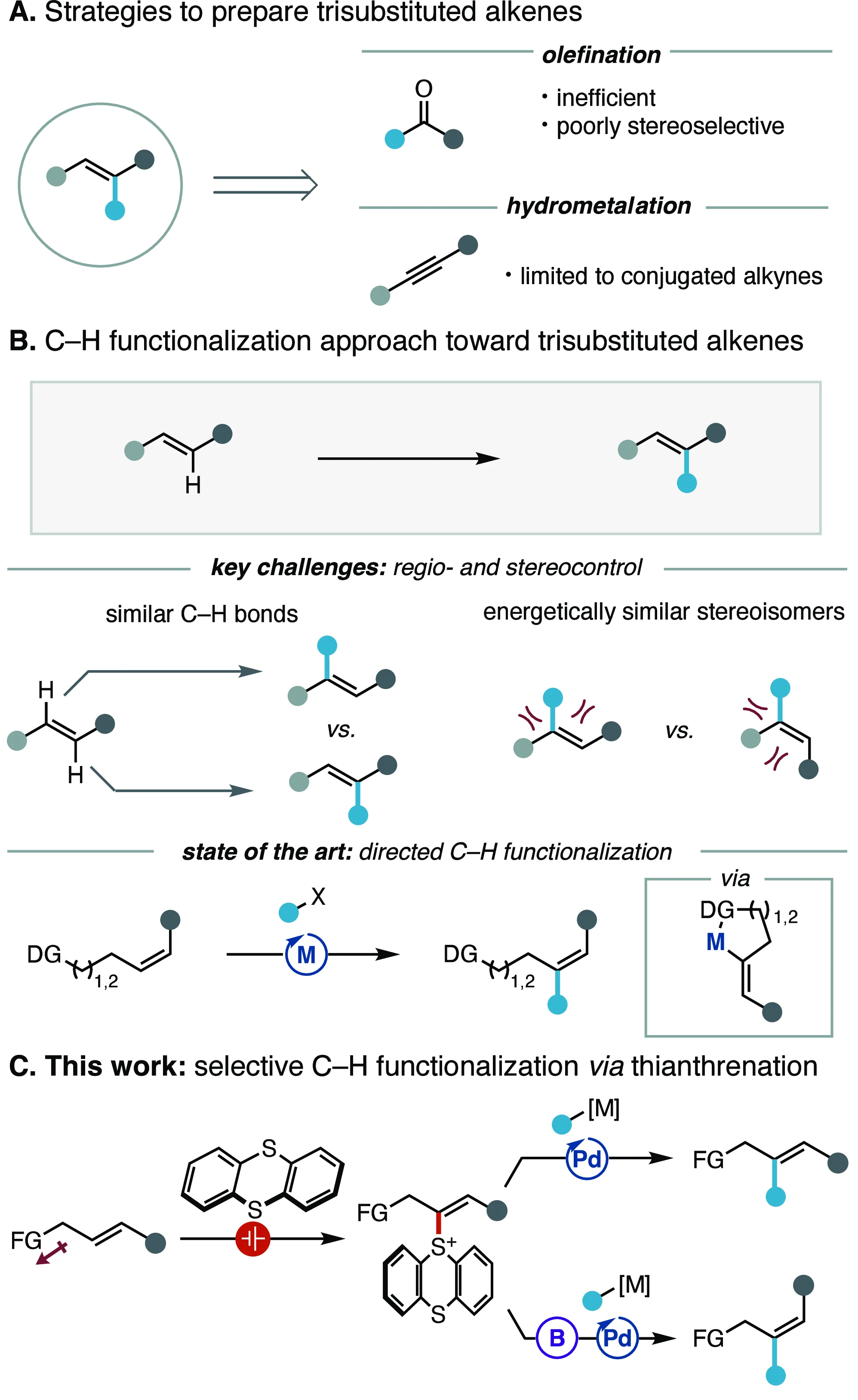
Project Overview^a^

a DG = directing group; M = metal; FG = functional group; B = base.

In principle, C–H functionalization of readily available 1,2-disubstituted alkenes would offer an attractive strategy to prepare trisubstituted alkenes (Scheme 1B). However, control over regio- and stereoselectivity in such processes requires distinguishing between both (1) similar alkenyl C–H bonds as well as (2) stereoisomeric alkene products that often have similar 1,3-allylic strain.^11^ Conjugated alkenes (e.g., styrene and acrylate derivatives) can provide sufficient electronic differentiation to enable regioselective Heck-type reactions.^12^ Functionalization of unactivated internal alkenes relies on *Z*-configured substrates bearing rigid, Lewis basic auxiliaries to control both regio- and stereochemistry via well-defined metallacycles.^13–15^ Accordingly, the introduction of a mechanistically distinct approach to internal alkene C–H functionalization is poised to have a significant synthetic impact.

We questioned whether thianthrenation would offer a complementary strategy for the C–H functionalization of internal alkenes (Scheme 1C). While recent progress has demonstrated that thianthrenation of terminal alkenes is highly regio- and stereoselective,^16–18^ internal alkenes present much smaller differences between their two reactive sites. Initial studies have indicated that internal alkenes do undergo stereospecific thianthrenation,^19^ but the factors governing regioselectivity for such substrates remain poorly understood.^20^ However, we reasoned that the elimination of dicationic alkene-thianthrenium adducts, the regiodetermining step of alkene thianthrenation, might be exceptionally electronically sensitive.^21^ If correct, native allylic functional groups could be sufficiently withdrawing to reliably induce regioselectivity.^22^ Herein, we illustrate that internal alkene thianthrenation can be rendered both regioselective and stereodivergent. Since thianthrenium salts can be engaged in a growing array of coupling reactions,^16,17,23–25^ these findings establish a versatile platform for internal alkene C–H functionalization.

At the outset of our investigation, we selected representative internal alkenes bearing functional groups in varied positions to probe the impact of electronic effects on thianthrenation regioselectivity (Scheme 2). We employed conditions that selectively generate a single dicationic alkene-thianthrenium adduct^26^ and treated the resultant crude mixture with basic alumina.^27^ First, we probed the elimination from a dicationic adduct derived from an electronically unbiased alkene (entry 1) to provide a baseline for the influence of modest steric differences on regioselectivity. This adduct eliminates with modest selectivity favoring thianthrenation at the less sterically hindered β position (24:76). Excitingly, the presence of an inductively withdrawing allylic acetate (entry 2) completely overturned the steric preference, resulting in exclusive α regioselectivity (>98:2). Even a homoallylic acetate (entry 3), which induces a more subtle electronic bias, still provides α regioselectivity (78:22).

**Scheme 2:**
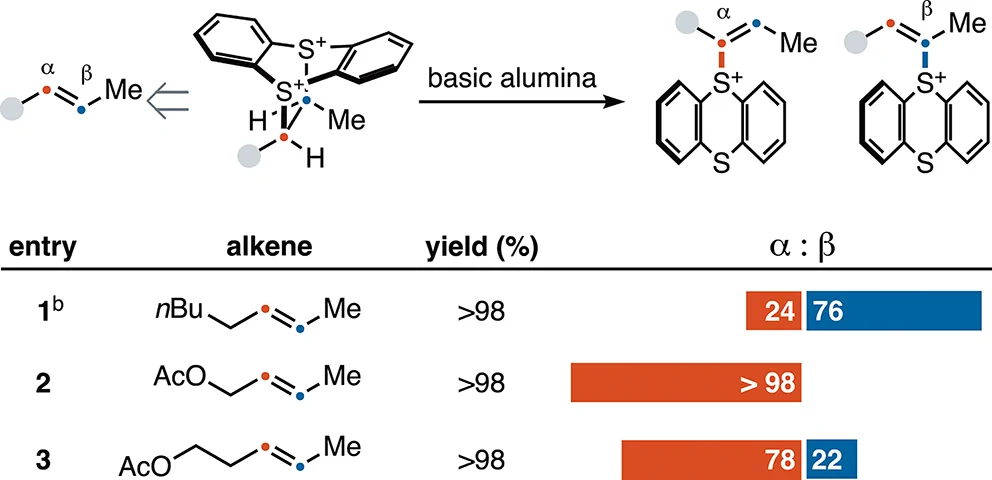
Validating the Electronic Sensitivity of Adduct Elimination^a^

a Yields and regioselectivities were determined by ^1^H NMR with CH_2_Br_2_ standard. Unless otherwise noted, the reaction was conducted electrochemically using alkene (0.2 mmol), TT (0.3 mmol), and 4 mL of MeCN (0.2 M KPF_6_), followed by basic alumina (5 g).
b The reaction was conducted using alkene (0.2 mmol), thianthrene *S*-oxide (0.2 mmol), trifluoroacetic anhydride (0.6 mmol), trifluoromethanesulfonic acid (0.2 mmol), and 0.8 mL of MeCN, followed by basic alumina (6 g).

Next, we investigated the breadth of functional groups that confer regioselectivity in internal alkene thianthrenation (Scheme 3). We found that a variety of internal alkenes bearing esters at the allylic position (**1**–**3**) are transformed into single alkenyl thianthrenium isomers, even those with substantial steric encumbrance (**3**). Protected allylic amines, such as nosyl (**4**) and phthalimide (**5**), are similarly functionalized with high regioselectivity. To rule out carbonyl- or sulfonyl-assisted deprotonation, we tested an allylic trifluoromethyl group (**6**) and found that this purely inductively withdrawing substituent still enforces regiocontrol. Furthermore, although electron-deficient internal alkenes react more slowly with oxidized thianthrene than terminal alkenes, we only observe reaction at the alkene in the presence of other π-systems known to undergo thianthrenation (**7**, **8**).^28–30^ Intramolecular competitions between allylic and more distal electron-withdrawing substituents revealed that thianthrenation selectively occurs proximal to the allylic substituent (**9**, **10**). The competition between two allylic groups, acetate and phthalimide (**11**), still resulted in selective thianthrenation despite the fact that each of these substituents independently enforces exclusive regioselectivity.

**Scheme 3:**
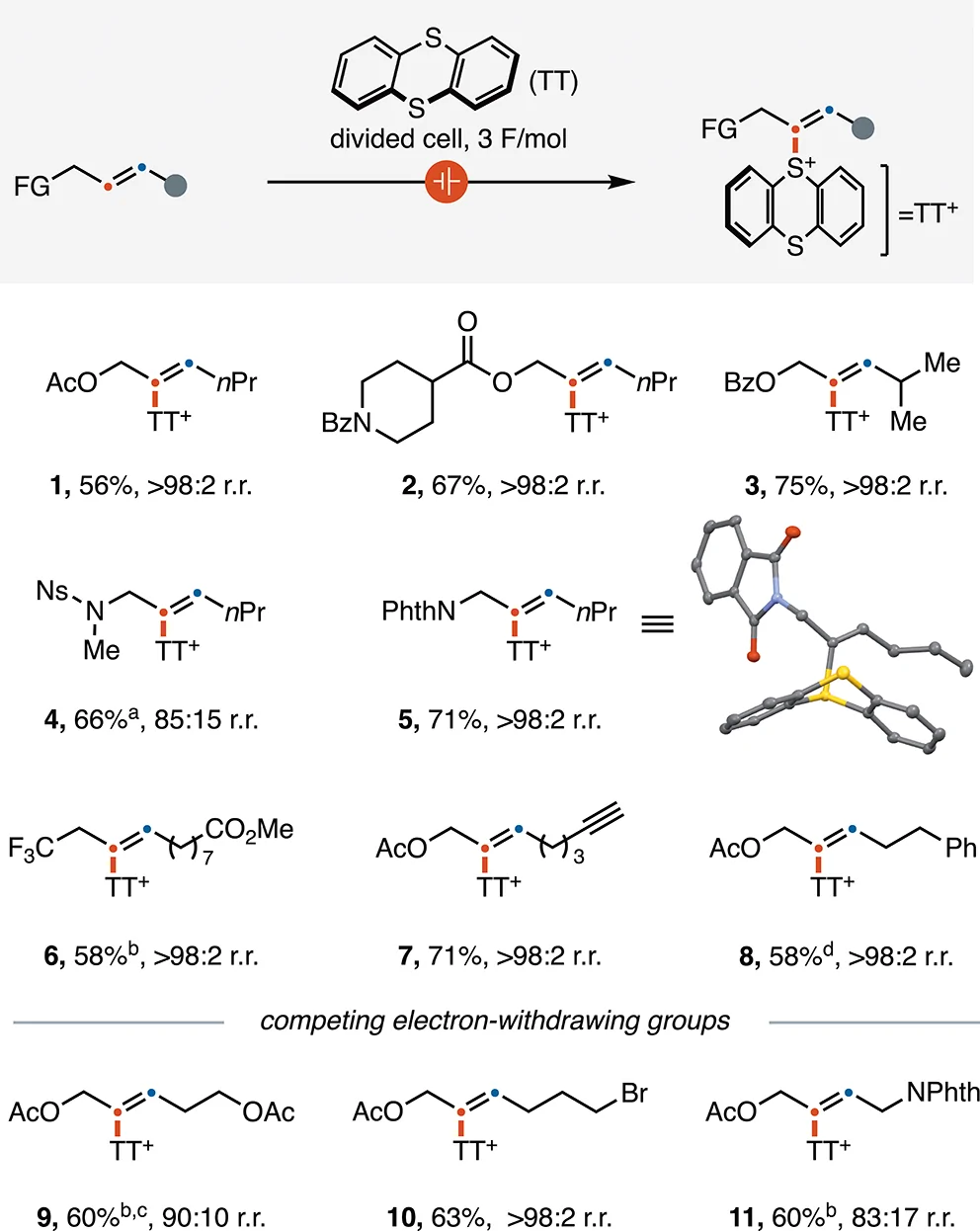
Scope of Regioselective Thianthrenation^a^

a 93:7 d.r.
b Yield and regioselectivity determined by quantitative ^1^H NMR using CH_2_Br_2_ standard.
c 90:10 d.r., reflective of d.r. of starting alkene.
d 2.5 F/mol alkene were passed.
e Unless otherwise noted, d.r. is >98:2. Yields and regioselectivities reflect those of the isolated products. Reactions were conducted electrochemically using alkene (0.4 mmol), TT (0.6 mmol), and 8 mL of MeCN (0.2 M KPF_6_), followed by basic alumina (10 g). All salts were isolated with PF_6_^–^ counteranion. The counteranion and H atoms are excluded from the crystallographic drawing.

Over the course of this investigation, we made a surprising observation. We observed that the stereochemical outcome of thianthrenation depended on the basic workup. While basic alumina always provided the stereoretentive *Z*-isomer, other bases resulted in eroded or even inverted stereoselectivity, giving rise to the *E*-isomer instead (Table S3). These data led to an *E*-selective quenching protocol that employs Cs_2_CO_3_ in the workup (Figure 1A). These modified conditions were effective across a variety of alkenes from our initial investigation (**12**–**19**), illustrating that we have established a stereodivergent protocol.

**Figure 1:**
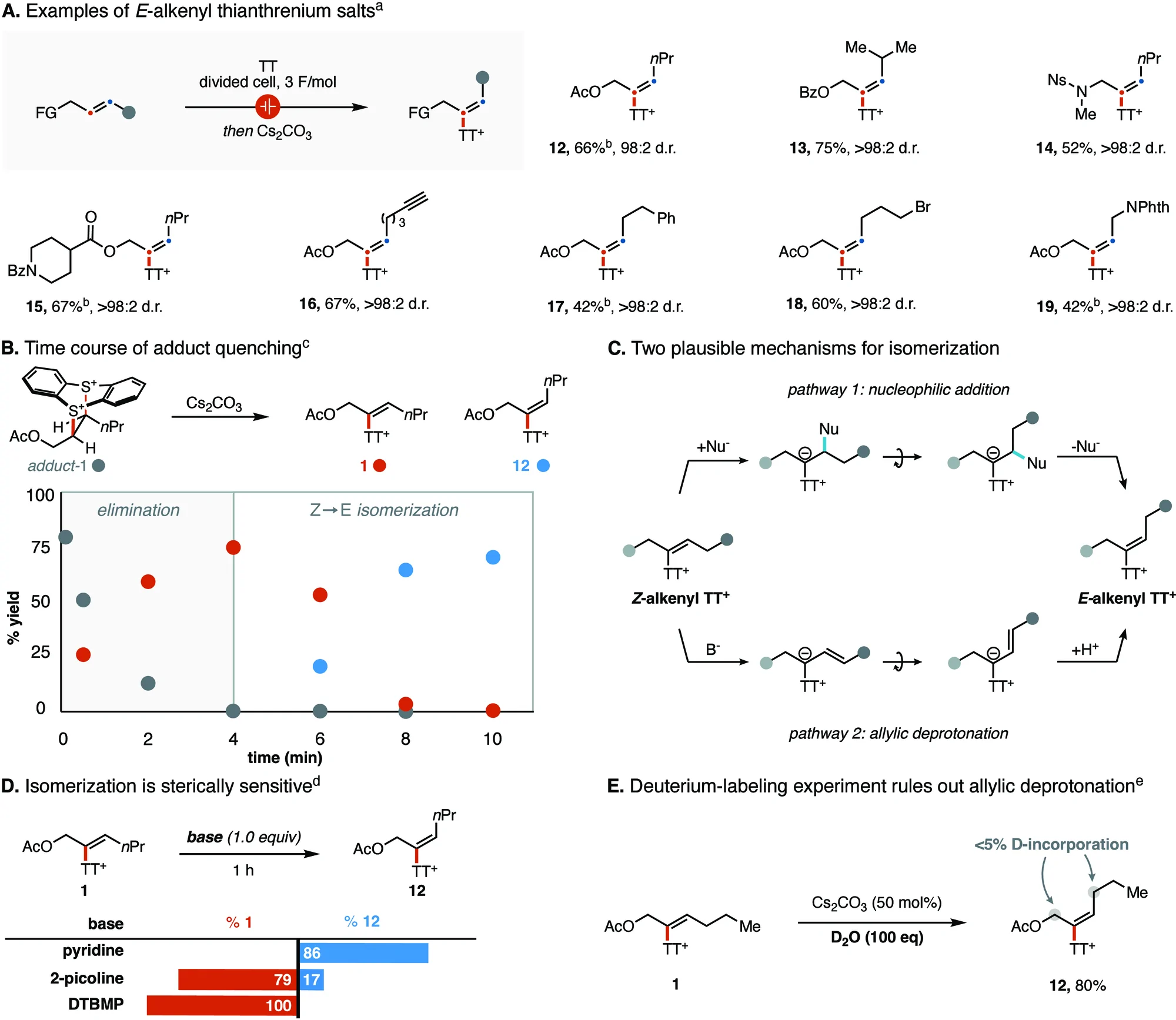
Discovery of a stereodivergent protocol and mechanistic investigations. TT = thianthrene, DTBMP = 2,6-di-*tert*-butyl-4-methylpyridine. In all cases, the regioselectivity of isolated products was >98:2. All salts were isolated with PF_6_^–^ counteranion. Unless otherwise noted, yields and stereoselectivities reflect those of isolated products. ^a^Reactions were conducted electrochemically using alkene (0.4 mmol), TT (0.6 mmol), and 8 mL of MeCN (0.2 M KPF_6_), followed by basic alumina (10 g) and then Cs_2_CO_3_ (0.2 mmol). ^b^Yield and stereoselectivity were determined by quantitative ^1^H NMR using CH_2_Br_2_ standard. ^c^Adduct was generated electrochemically using alkene (0.4 mmol), TT (0.6 mmol), and 8 mL of MeCN (0.2 M KPF_6_), followed by Cs_2_CO_3_ (2 mmol). Yields were monitored by quantitative ^1^H NMR using CH_2_Br_2_ standard. ^d^Reactions were conducted on a 0.1 mmol scale in 1 mL of MeCN. ^e^Reaction was conducted on a 0.05 mmol scale in 1 mL of MeCN.

To establish the origin of the distinct stereochemical outcome under these modified conditions, we next studied the elimination of the alkene-thianthrenium adduct with Cs_2_CO_3_. We found the elimination initially provides the stereoretentive product, **1**, which then rapidly converts to stereoisomer **12** (Figure 1B). We suspect that the thermodynamic preference for **12** arises from the minimization of steric clashes between the thianthrenium unit and the alkyl group. Given that isomerization involves formal π-bond rotation, we considered two possible mechanisms for reversible π-bond cleavage: (1) nucleophilic addition^31,32^ and (2) allylic deprotonation^33^ (Figure 1C).

Next, we conducted a series of experiments to distinguish between the distinct isomerization mechanisms. We anticipated that the conjugate addition pathway would be sensitive to the steric profile of the added Lewis base. Considering that pyridine efficiently promotes isomerization, we probed the impact of sterically hindered pyridine analogues on the reaction (Figure 1D). Isomerization in the presence of 2-picoline was significantly slower, and 2,6-di-*tert*-butyl-4-methylpyridine (DTBMP) did not induce isomerization at all. This acute steric sensitivity is consistent with a conjugate addition mechanism. However, since a particularly sterically sensitive allylic deprotonation pathway remained plausible, we directly probed the transient formation of an allylic ylide intermediate. Cs_2_CO_3_-catalyzed isomerization of thianthrenium salt **1** in the presence of excess D_2_O revealed minimal (less than 5%) allylic deuteration (Figure 1E). These data unambiguously exclude allylic deprotonation as the primary isomerization pathway.^34^

We next aimed to validate the synthetic utility of this stereodivergent C–H functionalization protocol (Figure 2). We found that the thianthrenation step translated well to preparative scale, selectively delivering each stereoisomer (**20**, **5**). When followed by stereoretentive cross coupling, this protocol allows an internal alkene to be readily transformed into diverse *E*- and *Z*-trisubstituted alkene products (**21**–**26**). All of the cross couplings were stereospecific, despite in some cases relying on the same carbonate bases that we had found to promote stereoisomerization (Tables S8 and S9). The precise stereocontrol enabled by this approach starkly contrasts with traditional trisubstituted alkene synthesis, where distinct synthetic routes must be devised to access each stereoisomer.

**Figure 2:**
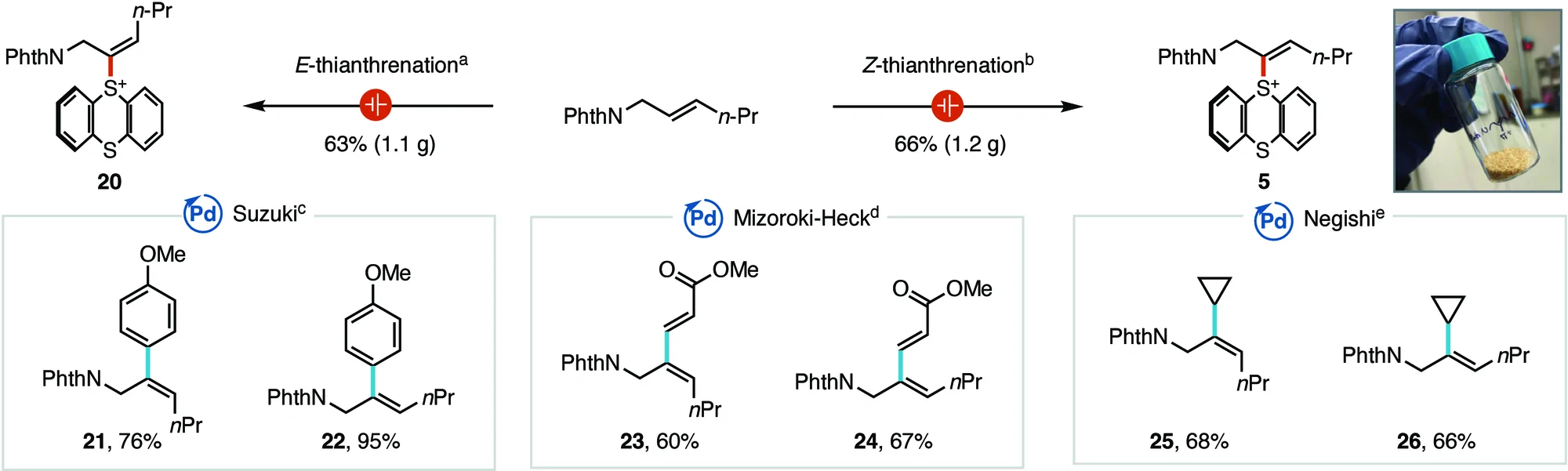
Validating the synthetic utility of trisubstituted alkenyl thianthrenium salts. In all cases, r.r. is >98:2, and d.r. is >98:2. All salts were isolated with PF_6_^–^ counteranion. ^a^Reaction was conducted electrochemically using alkene (3.0 mmol), TT (4.5 mmol), and 60 mL of MeCN (0.2 M KPF_6_), followed by basic alumina (75 g) and then Cs_2_CO_3_ (1.5 mmol). ^b^Reaction was conducted electrochemically using alkene (3.0 mmol), TT (4.5 mmol), and 60 mL of MeCN (0.2 M KPF_6_), followed by basic alumina (75 g). ^c^Reactions were conducted with alkene (0.20 mmol), Pd_2_(dba)_3_ (2.5 mol %), P­(*o*-tolyl)_3_ (11 mol %), Cs_2_CO_3_ (0.30 mmol), boronic acid (0.30 mmol), and 4 mL of THF. ^d^Reactions were conducted with alkene (0.20 mmol), Pd_2_(dba)_3_ (5 mol %), K_2_CO_3_ (0.50 mmol), acrylate (0.50 mmol), and 2 mL of THF. ^e^Reactions were conducted with alkene (0.20 mmol), Pd­(PPh_3_)_2_Cl_2_ (5 mol %), alkylzinc halide (0.30 mmol), and 1.5 mL of THF.

In summary, we have introduced a versatile platform for the C–H functionalization of internal alkenes. Our approach was enabled by the discovery that the regioselectivity of alkene-thianthrenium adduct elimination is exceptionally sensitive to electronic biases. We found that a variety of common allylic functional groups are sufficiently inductively withdrawing to induce highly regioselective internal alkene thianthrenation. This stereoretentive process can be followed by an isomerization, providing protocols to access either stereoisomer. Mechanistic studies suggest that the isomerization proceeds through reversible nucleophilic addition. Since these alkenyl thianthrenium salts readily engage in stereoretentive cross coupling, our studies advance a stereodivergent approach to transform internal alkenes into trisubstituted analogues. We anticipate that these methods will be broadly adopted in the synthesis of stereodefined alkenes.

## Supplementary Material



## Notes

Experimental procedures, additional data, characterization data, and NMR spectra of all compounds (PDF)
