# Supplementary material for: Regioselective and Stereodivergent C–H Functionalization of Internal Alkenes
Source: J Am Chem Soc. 2026 Oct 15;148(42):45330–6. doi: 10.1021/jacs.6c17366 (PMC13721155; doi:10.1021/jacs.6c17366)
Supplement: Supplementary file 1 [file ja6c17366_si_001.pdf]

Regioselective and Stereodivergent C–H Functionalization of Internal Alkenes

Elizabeth A. Ryutov[a], Peter J. Verardi[a], Song N. Dang[a], Karina Targos[a], Leah C. Garman[a]
Zachary K. Wickens*[a]

[a]Department of Chemistry, University of Wisconsin-Madison, 1101 University Avenue, Madison, WI 53706, United States

Table of Contents

1. **General Methods and Materials**
   - Isolation of alkenyl thianthrenium salts
   - Electrochemical methods and materials
   - *General Procedure A. 0.4 mmol scale electrochemical adduct generation*
   - *General Procedure B. Preparation of* Z-*alkenyl thianthrenium salt*
   - *General Procedure C. Preparation of* E-*alkenyl thianthrenium salt*
   - 3 mmol scale batch electrolysis procedures
2. **Supplementary Data**
   - Impact of thianthrenium adduct identity on thianthrenation stereoselectivity
   - Detection of thianthrene oxidation state required for adduct formation
   - Discussion of stereoretentive adduct formation and elimination
   - Procedures for determining elimination regioselectivity
   - Optimization of electrochemical thianthrenium adduct formation
   - Regioselectivity of chemical thianthrenation
   - Screening of bases for adduct elimination
   - Impact of base identity on regioselectivity
   - Probing steric sensitivity of isomerization
   - Probing the deuteration of reaction intermediate
   - Isomerization time course
   - Investigating electrophile stability under cross-coupling conditions
3. **Substrate Preparation and Characterization**
4. **Cross-coupling procedures**
   - *General Procedure D.* $C(sp^2)$-$C(sp^2)$ Suzuki Coupling
   - *General Procedure E.* $C(sp^2)$-$C(sp^2)$ Mizoroki-Heck Coupling
   - *General Procedure F.* $C(sp^2)$-$C(sp^3)$ Negishi Coupling
5. **Product Isolation and Characterization**
6. **X-Ray Crystallography Data**
7. **NMR Spectra**

1. General Methods and Materials

DMF, THF, Et_2O, and DCM were dried by passing through activated alumina columns. Potassium hexafluorophosphate (KPF_6) was dried under vacuum prior to use. Unless otherwise noted, other commercially available reagents were used as received. Crude mixtures were evaluated by thin-layer chromatography using EMD/Merck silica gel 60 F254 pre-coated plates (0.25 mm) and were visualized by UV and/or $KMnO_4$ staining. Flash chromatography was performed with a Biotage Isolera One automated chromatography system with re-packed silica or C2 silica (vide infra) columns (technical grade silica, pore size 60 Å, 230-400 mesh particle size, 40-63 particle size) unless otherwise noted. Purified materials were dried in vacuo (0.050 Torr) to remove trace solvent. ^1H, ^{13}C, ^{19}F, and ^{31}P spectra were taken using a Bruker Avance400 with a BBFO Probe, a Bruker Avance-500 with a DCH Cryoprobe, or a Bruker Avance Neo 500 with a Prodigy probe. NMR data are reported relative to residual $CHCl_3$ (^1H, δ = 7.26 ppm), $CDCl_3$ (^{13}C, δ = 77.16 ppm), CH_3CN (^1H, δ = 1.94 ppm), or CD_3CN (^{13}C, δ = 118.26 ppm). Data for ^1H-NMR spectra are reported as follows: chemical shift (δ ppm) (multiplicity, coupling constant (Hz), integration). Multiplicity and qualifier abbreviations are as follows: s = singlet, d = doublet, t = triplet, q = quartet, p = pentet, hept = heptet, m = multiplet, br = broad. All NMR yields were determined via reference against an internal standard (dibromomethane or mesitylene for ^1H-NMR). Mass spectrometry data was collected on a Thermo Scientific Q Exactive Plus Mass Spectrometer, a Waters Acquity™ LCMS, or a Shimadzu GCMS-TQ8040NX™.

Abbreviations: DCM—dichloromethane, DMAP — 4-dimethylamino pyridine, DMF—dimethyl formamide, EtOAc—ethyl acetate, MeCN—acetonitrile, MeOH—methanol, *n*-Pr—*n*-propyl, OAc—acetate, RVC—reticulated vitreous carbon, SS—stainless steel, Ph—phenyl, TFA—trifluoroacetic acid, THF—tetrahydrofuran, RSM—returned starting material.

Isolation of alkenyl thianthrenium salts.

Although many previously reported alkenyl thianthrenium salts can be purified by flash chromatography, we found that many of the products disclosed herein decomposed when subjected to silica gel chromatography. We therefore isolated most products by wet loading onto deactivated C2 silica, which improved the purity and recovery of isolated products. The salts can also be isolated by HPLC or recrystallization.

Procedure for preparing C2 silica. Adapted from a protocol developed by Fox and coworkers (*J. Am. Chem. Soc.* **2007**, *129*, 1, 22–23). Flash silica (200 g, technical grade, 60 Å) was suspended in 400 mL of anhydrous (dried over molecular sieves) $CHCl_3$ in a round-bottom flask under a nitrogen atmosphere. The flask was chilled by an ice bath. Ethyltrichlorosilane (10.2 g, 62 mmol) was added via syringe. After the addition was completed, the flask was shaken vigorously under nitrogen atmosphere to mix (HCl is formed). The mixture was allowed to sit at room temperature with occasional shaking for 16 h (suspension becomes yellow). The silica gel was filtered with a

Buchner funnel and washed with $CHCl_3$ (2 x 400 mL), followed by MeOH (3 x 300 mL, until filtrate comes out clear). The silica gel was transferred to a round-bottom flask and dried by heating (40°C) under vacuum. The C2 silica was reused (10+ times) by the following procedure: upon completion of a chromatography gradient, the column was flushed with 5 CV isopropyl alcohol. The C2 silica can be stored dry or in isopropyl alcohol until the next use.

Electrochemical methods and materials.

3 mmol constant current electrolysis experiments were carried out using a Jesverty 30 Volt DC Power Supply (purchasable on Amazon, ASIN: B09YSJQWRG). Constant current electrolysis experiments on the 0.40 mmol (scope-scale) and 0.20 mmol (reaction optimization) scales carried out using a custom–made low current power supply (see Figure S1) which was externally calibrated with a multimeter using a 10–Ohm resistor.

Low-current Power Supply: Original design and fabrication by Dr. Blaise J. Thompson. Provides an operational range of ±0.01 – 14.99 mA, tunable by slide resistor, delivering power to banana socket pair. The power supply is limited to ±15V for bulk electrolysis and is powered by an 18V wall wart. Circuitry is housed within an aluminum enclosure.

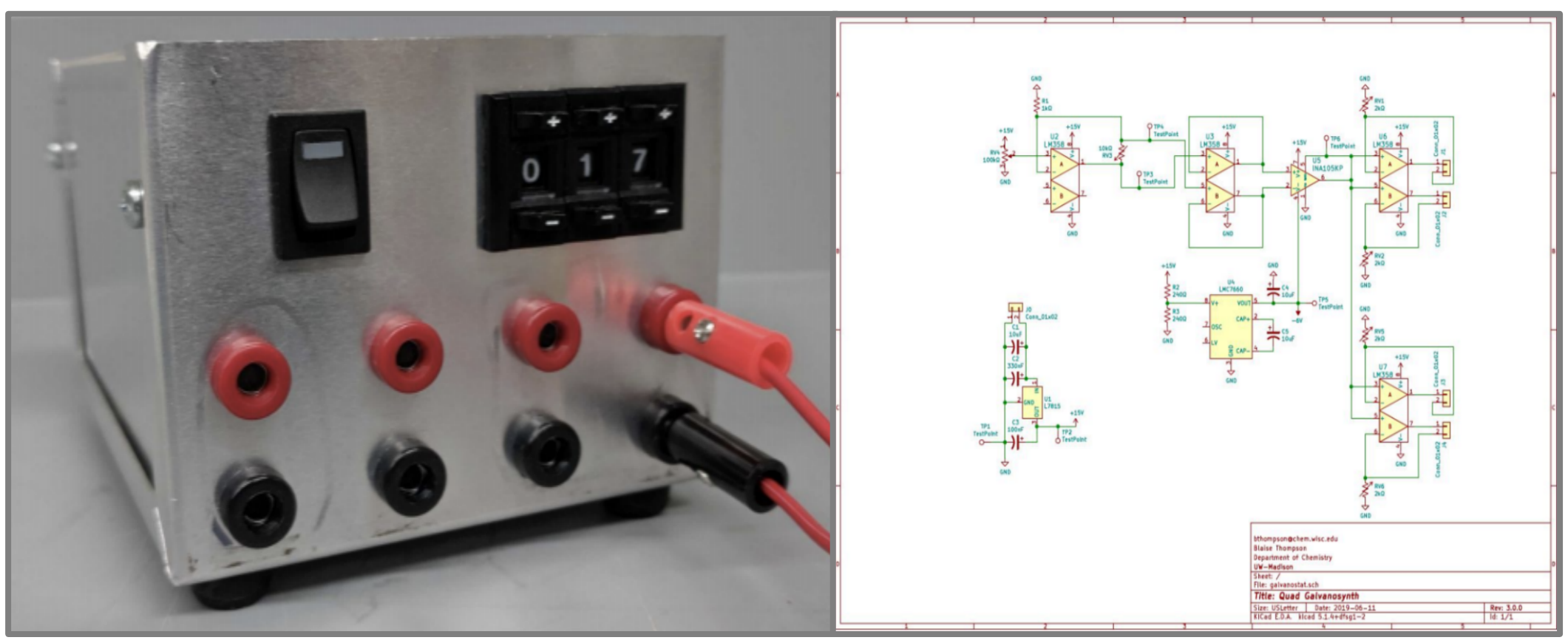

Figure S1. Custom low-current power supply.

Divided cell electrode fabrication and assembly. Divided cell fabricated in house. Glass frit purchased from Ace Glass (7176-36). Anode electrode assembled via affixing end of the silver wire (Belden Hook-Up wire, item no. 83005 007100) around the pencil (JuneGold 2B graphite 2 mm), wrapping in Teflon tape to prevent exposure, then piercing RVC with pencil. PTFE tubing (Cole-Parmer; 1/32” ID, 1/16” OD, item number EW-06407-41) connects both sides of the divided cell to normalize pressure. Septa inner diameter 16 mm. Stainless steel purchased from Grainger; stainless steel lockwire, 0.025" diameter, item number 16Y043.

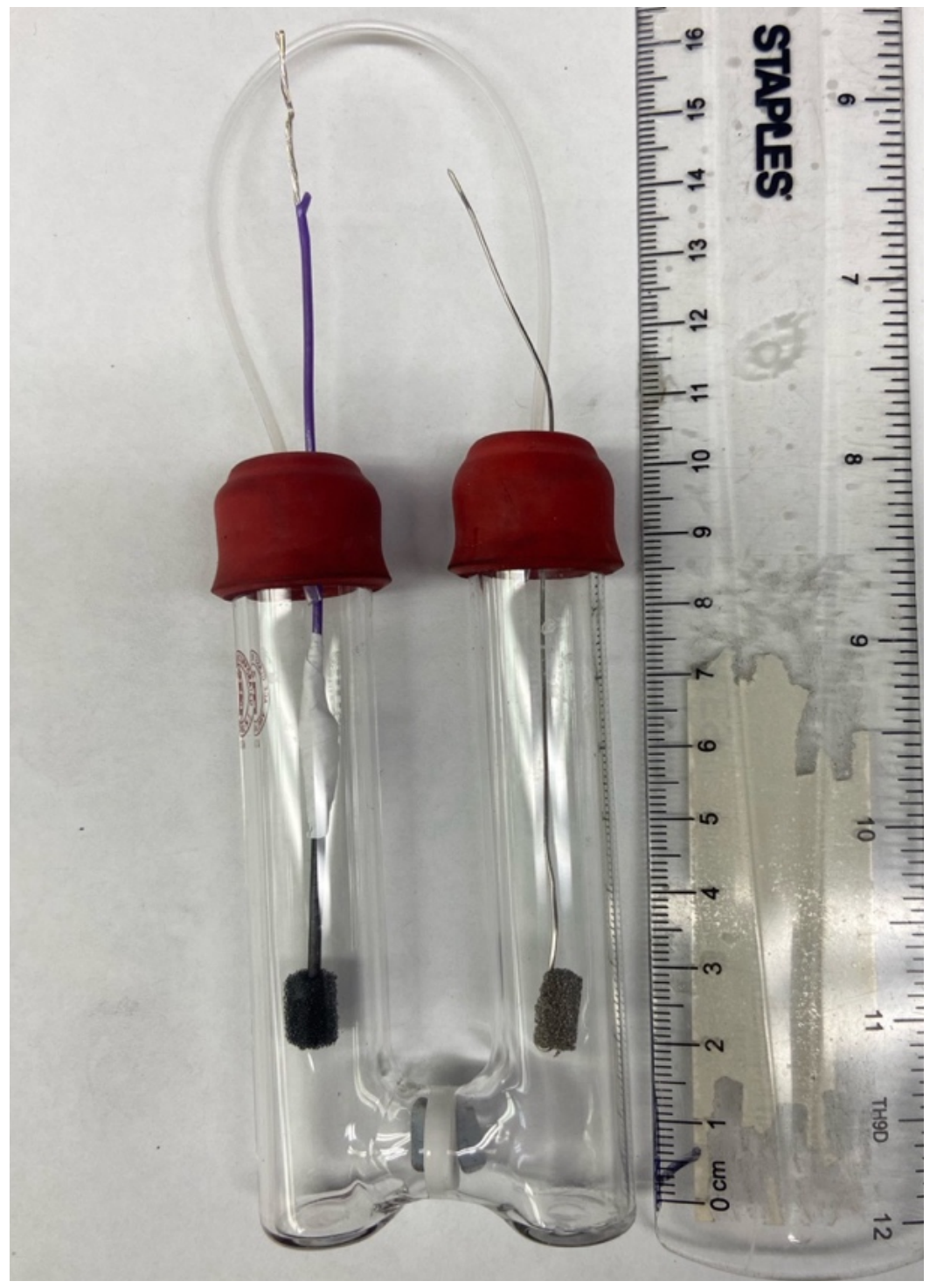

Figure S2. Standard scale divided cell electrochemical setup.

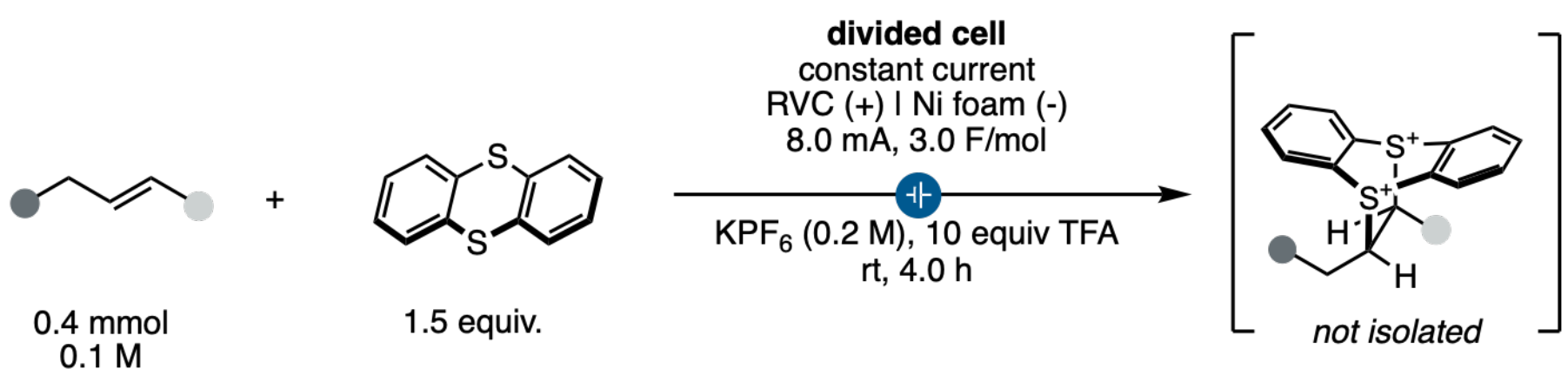

General Procedure A. *0.4 mmol scale electrochemical adduct generation.*

To an oven-dried electrochemical H-cell equipped with magnetic stir bars was added thianthrene (130 mg, 1.5 Eq, 0.600 mmol) to the anode compartment and dried KPF_6 (294 mg, 4 Eq, 1.60 mmol) to both compartments. The cell was equipped with two septa containing a stainless-steel wire/Ni foam cathode assembly (1.0 x 1.0 cm) and a pencil/RVC anode assembly (8 × 5 × 5 mm) connected with Teflon tubing to equalize pressure. MeCN (8.0 mL) was added to the cathode compartment, and the glass frit was allowed to become saturated (<1 min). MeCN (8.0 mL) was added to the anode compartment, followed by alkene (1 Eq, 0.400 mmol) (electrode depth: 1 cm). Trifluoroacetic acid (456 mg, 306 μL, 10 Eq, 4.00 mmol) was added to the cathode compartment and both sides of the cell were stirred at room temperature and electrolyzed under a constant current of 8.0 mA for 4.00 h (3.0 F/mol alkene).

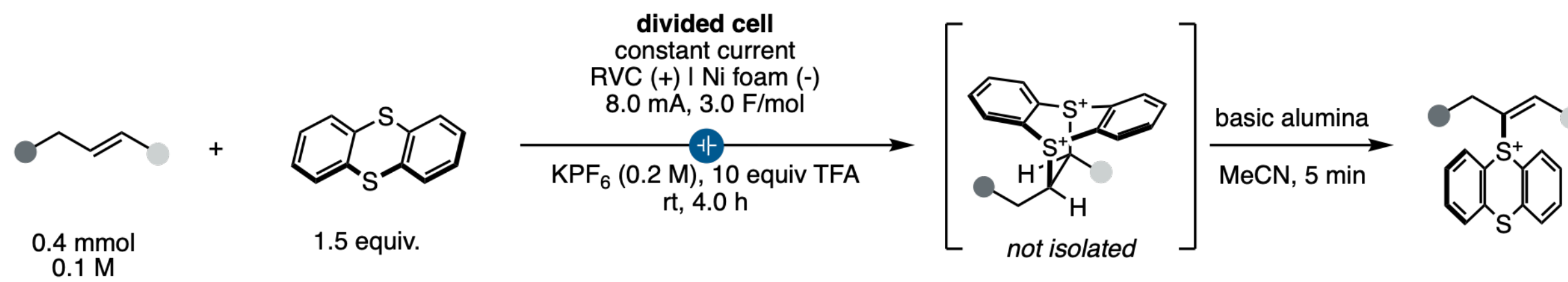

General Procedure B. *0.4 mmol-scale preparation of Z-alkenyl thianthrenium salt*

The thianthrenium adduct was electrochemically generated following ***General Procedure A.*** Upon completion of electrolysis, the anode solution was then transferred to a 100 mL round-bottom flask equipped with a magnetic stir bar and the anode compartment was rinsed forward with MeCN (5 x 1.2 mL). Basic alumina (10 g, activated, basic, Brockmann II) was added to the anode solution and the mixture was stirred for 5 minutes. The solids were removed by vacuum filtration, rinsing the alumina pad with MeCN (2 x 8 mL), taking care to fully disperse the alumina in solution using a spatula. From the filtrate, MeCN was removed in vacuo. The residue was dissolved in DCM (40 mL) and 5% wt/wt aq. KPF_6 was added (40 mL). The phases were separated and the aqueous layer washed 2x with DCM. The combined organic layer was dried over Na_2SO_4, solids were removed by filtration, and solvent removed in vacuo. The resulting alkenyl thianthrenium salt was isolated by recrystallization or flash chromatography using C2 silica.

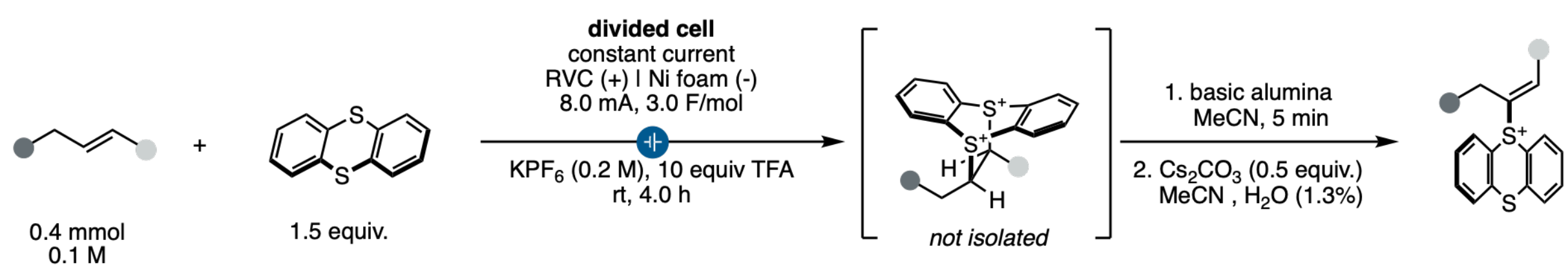

General procedure C. *0.4 mmol scale preparation of E-alkenyl thianthrenium salt.*

The thianthrenium adduct was electrochemically generated following ***General Procedure A.*** Upon completion of electrolysis, the anode solution was transferred to a 100 mL round-bottom flask equipped with a magnetic stir bar and the anode compartment was rinsed forward with MeCN (5 x 1.2 mL). Basic alumina (10 g, activated, basic, Brockmann II) was added to the anode solution and the mixture was stirred for 5 minutes. The solids were removed by vacuum filtration, rinsing the alumina pad with MeCN (2 x 8 mL), taking care to fully disperse the alumina in solution using a spatula. From the filtrate, MeCN was removed in vacuo. The residue was then dissolved in anhydrous MeCN (8 mL) and DI H_2O (0.10 mL). Cs_2CO_3 (0.50 Eq) was added, and the contents were stirred vigorously until complete isomerization was observed by NMR (10 min – 2 h, monitoring by NMR). The mixture was then diluted with ethyl acetate (50 mL) and transferred to a separatory funnel. 5% wt/wt aq. KPF_6 was added (40 mL) and the layers were separated. The aqueous layer was washed with ethyl acetate (2 x 40 mL). The organic layers were combined and dried over $MgSO_4$. The solids were removed by filtration, and the solvent was removed in vacuo. The residue was redissolved in DCM (60 mL), and solids removed by filtration. The solvent was

removed in vacuo, and the resulting salt was isolated by recrystallization or flash chromatography using C2 silica.

Note: Quenching with Brockmann II alumina provided higher recovery than Brockmann I alumina. Brockmann II alumina was prepared by addition of water to Brockmann I alumina (Sorbent Technologies. Deactivation of Alumina. Sorbtech. https://www.sorbtech.com/chromatography/adsorbents/alumina/activated-alumina-chromatographic/deactivation-of-alumina/).

3 mmol batch electrolysis procedures.

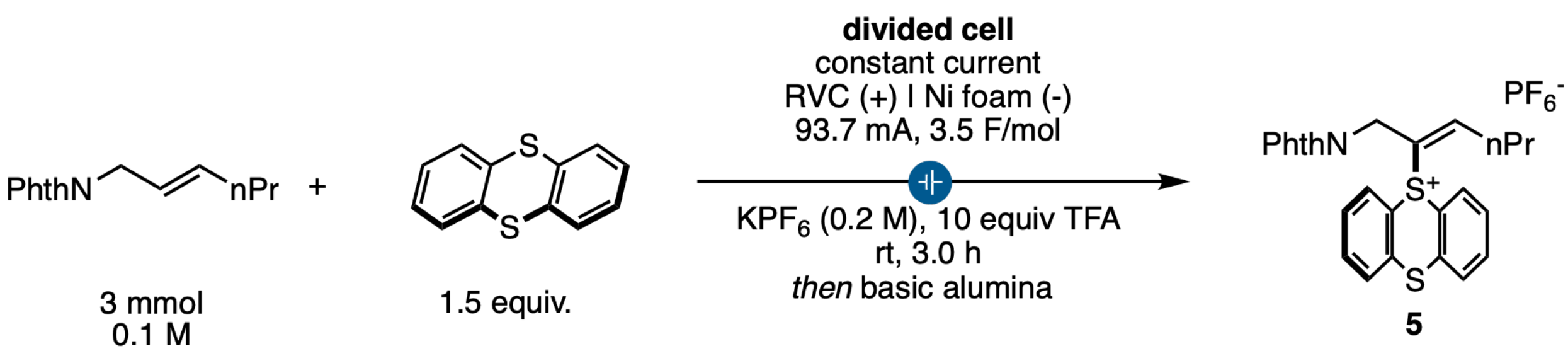

3 mmol scale batch preparation of (*Z*)-5-(1-(1,3-dioxoisoindolin-2-yl)hex-2-en-2-yl)-5*H*-thianthren-5-ium hexafluorophosphate(V). To an oven-dried electrochemical H-cell equipped with magnetic stir bars was added thianthrene (973.4 mg, 1.5 Eq, 4.500 mmol) to the anode compartment and dried KPF_6 (2.209 g, 4 Eq, 12.00 mmol) to both compartments. The cell was equipped with two septa containing a stainless-steel wire/Ni foam cathode assembly (3.5 x 3.5 cm) and a pencil/RVC anode assembly (30 × 15 × 15 mm) connected with Teflon tubing to equalize pressure. MeCN (60 mL) was added to the cathode compartment, and the glass frit was allowed to become saturated (<1 min). MeCN (60 mL) was added to the anode compartment, followed by (E)-2-(hex-2-en-1-yl)isoindoline-1,3-dione (687.8 mg, 1 Eq, 3.000 mmol) (Electrode depth: 1 cm). TFA (3.421 g, 2.311 mL, 10 Eq, 30.00 mmol) was added to the cathode compartment and both sides of the cell were stirred at room temperature and electrolyzed under a constant current of 93.75 mA for 3.0 h (3.5 F/mol alkene). Upon completion of electrolysis, the anode solution was transferred to a 250-mL round bottom flask, and the anode compartment was rinsed with MeCN (5 x 3 mL). Basic alumina (75 g, activated, basic, Brockmann II) was added to the anode solution. The solids were then removed by vacuum filtration, rinsing the alumina pad with MeCN (3 x 50 mL). Solvent was removed in vacuo, then the residue was dissolved in DCM (150 mL) and 5% wt/wt aq. KPF_6 was added (120 mL). The phases were separated and the aqueous layer washed 2x with DCM. The combined organic layer was dried over Na_2SO_4, solids were removed by filtration, and the solvent was removed in vacuo. The resulting alkenyl thianthrenium salt was isolated by recrystallization from DCM/ethyl acetate.

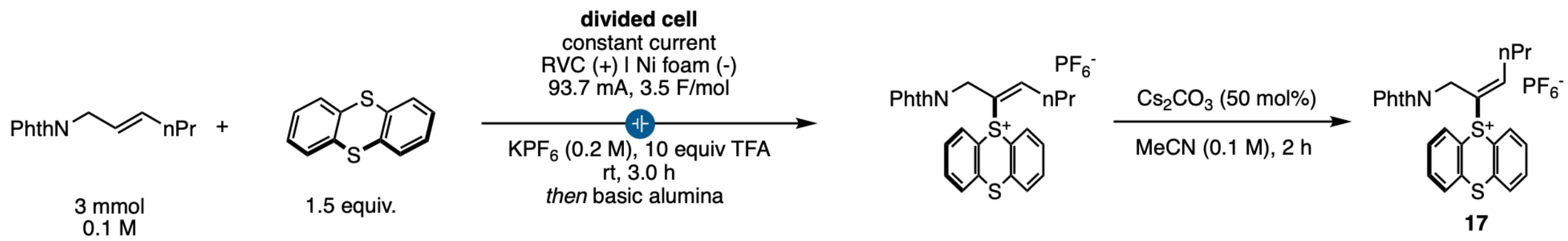

3 mmol scale batch preparation of (*E*)-5-(1-(1,3-dioxoisoindolin-2-yl)hex-2-en-2-yl)-5*H*-thianthren-5-ium hexafluorophosphate(V). To an oven-dried electrochemical H-cell equipped with magnetic stir bars was added thianthrene (973.4 mg, 1.5 Eq, 4.500 mmol) to the anode compartment and dried KPF_6 (2.209 g, 4 Eq, 12.00 mmol) to both compartments. The cell was equipped with two septa containing a stainless-steel wire/Ni foam cathode assembly (3.5 x 3.5 cm) and a pencil/RVC anode assembly (30 × 15 × 15 mm) connected with Teflon tubing to equalize pressure. MeCN (60 mL) was added to the cathode compartment, and the glass frit was allowed to become saturated (<1 min). MeCN (60 mL) was added to the anode compartment, followed by (E)-2-(hex-2-en-1-yl)isoindoline-1,3-dione (687.8 mg, 1 Eq, 3.000 mmol) (Electrode depth: 1 cm). TFA (3.421 g, 2.311 mL, 10 Eq, 30.00 mmol) was added to the cathode compartment and both sides of the cell were stirred at room temperature and electrolyzed under a constant current of 93.75 mA for 3.0 h (3.5 F/mol alkene). Upon completion of electrolysis, the anode solution was transferred to a 250-mL round bottom flask, and the anode compartment was rinsed with MeCN (5 x 3 mL). Basic alumina (75 g, activated, basic, Brockmann II) was added to the anode solution. The solids were then removed by vacuum filtration, rinsing the alumina pad with MeCN (3 x 50 mL). From the filtrate, MeCN was removed in vacuo. The residue was redissolved in anhydrous MeCN (60 mL) and Cs_2CO_3 (488.7 mg, 0.5 Eq, 1.50 mmol) was added. The contents were stirred until isomerization was complete by NMR (2 h). MeCN was removed in vacuo, and the mixture was dissolved in ethyl acetate (180 mL) and transferred to a separatory funnel. 5% wt/wt aq. KPF_6 was added (180 mL) and the layers were separated. The aqueous layer was washed with ethyl acetate (2 x 100 mL). The organic layers were combined and dried over $MgSO_4$. The solids were removed by filtration, and the solvent was removed in vacuo. The residue was redissolved in DCM (100 mL), and solids were removed by filtration. The solvent was removed in vacuo, and the resulting salt was isolated by recrystallization from DCM/ethyl acetate.

2. Supplementary Data

Impact of thianthrenium adduct identity on thianthrenation stereoselectivity.

Electrochemical alkene thianthrenation is known to generate adducts with varying stoichiometries of thianthrene and alkenes, which form from different oxidation states of thianthrene. *Mono*-thianthrenium adducts are believed to form by [4+2] cycloaddition of the alkene with TT^{2+} (*Angew. Chem. Int. Ed.* **2020**, *59*, 14, 5616-5620), whereas *bis*-thianthrenium adducts are believed to form by sequential radical addition of $TT^{\cdot+}$ (*Science* **2025**, *389*, 6766, 1239-1245). In the present work, we have observed that electron-deficient internal alkenes, such as those bearing allylic functional groups, exclusively form *mono*-thianthrenium adducts. In contrast, substrates without any electron-withdrawing groups, such as *trans*-2-octene, can form mixtures of *mono*- and *bis*-thianthrenium adducts under electrochemical thianthrenation conditions. We hypothesize that for electron-rich alkenes, radical addition of $TT^{\cdot+}$ is kinetically feasible, but electron-deficient alkenes can react only with TT^{2+}, thus forming *mono*-thianthrenium adducts only (*vide infra*).

We studied the stereochemistry of elimination from the electrochemically generated mixture of *mono*- and *bis*- thianthrenium adducts generated from *trans*-2-octene (Figure S3). The mixture eliminated to an unselective mixture of regio- and stereoisomers. However, quenching the isolated *mono*-thianthrenium adduct (isolated by precipitation with DCM), revealed that this adduct undergoes stereoretentive elimination with moderate regioselectivity (Figure S4). This indicates that thianthrenation *via* the *bis*-thianthrenium adduct is not stereoretentive. These data are consistent with previous work that has demonstrated that *mono*-thianthrenium adduct formation and elimination is stereoretentive, but *bis*-adduct elimination is not (*J. Org. Chem.* **2003**, *68* (23), 8910–8917). Considering that the moderately electron-deficient alkenes employed in this study exclusively form *mono*-thianthrenium adducts, we observe high stereoretention in all cases.

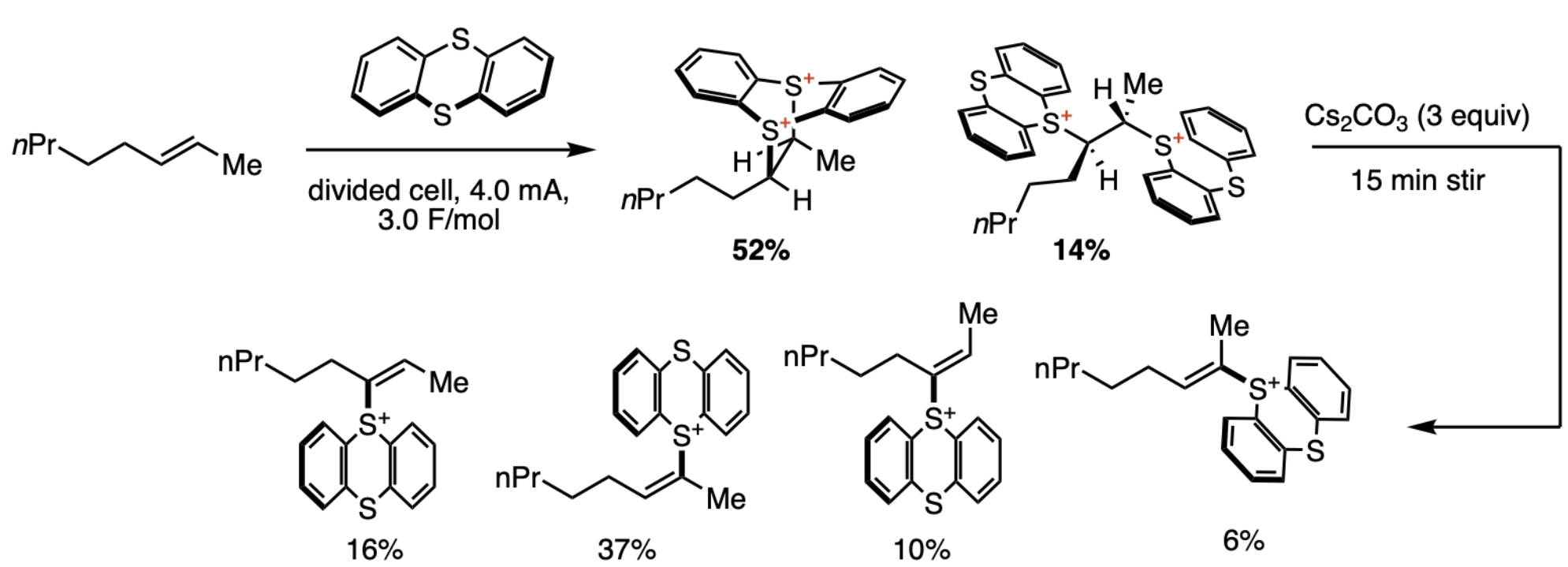

Figure S3. Elimination selectivity of mixture of *mono*- and *bis*- thianthrenium adducts.

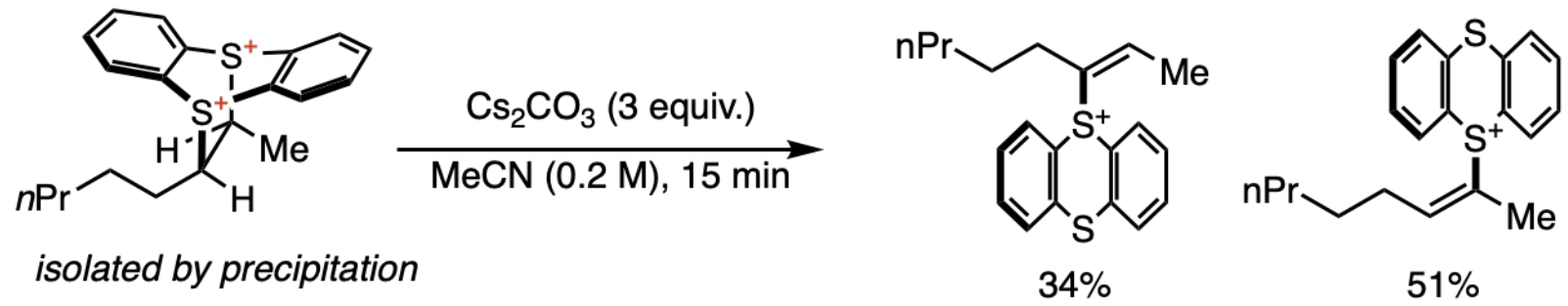

Figure S4. Elimination selectivity of isolated *mono*-thianthrenium adduct derived from *trans*-2-octene.

Detection of thianthrene oxidation state required for adduct formation.

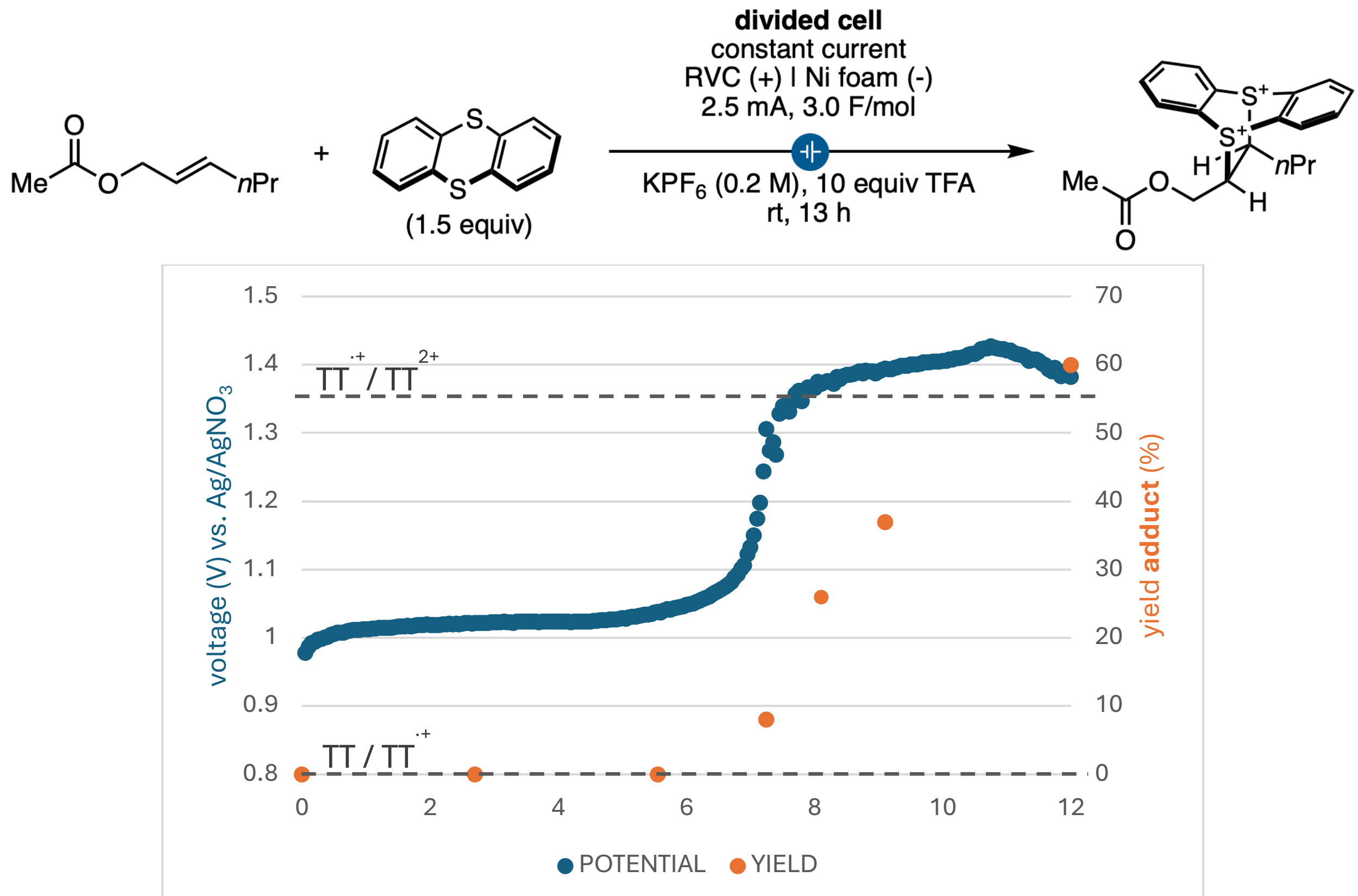

Figure S5. Working potential vs. thianthrenium adduct yield.

Procedure for monitoring working potential: To an oven-dried divided electrochemical cell equipped with magnetic stir bars was added thianthrene (130 mg, 1.5 Eq, 0.600 mmol) to the anode compartment and dried KPF_6 (294 mg, 4.00 Eq, 1.60 mmol) to both compartments. The cell was equipped with two septa containing a stainless-steel wire/Ni foam cathode assembly (1.5 x 0.8 cm) and a pencil/RVC anode assembly (7 × 6 × 6 mm) connected with a Teflon tubing to equalize pressure. The anode assembly also contained an $Ag/AgNO_3$ reference electrode. MeCN (8 mL, cathode) was added to the cathode compartment, and the glass frit was allowed to become saturated (<1 min). MeCN (8 mL, anode) was added to the anode compartment (Electrode depth: 1 cm). Then, (E)-hex-2-en-1-yl acetate (56.9 mg, 63.3 µL, 1 Eq, 0.400 mmol) was added to the anode compartment, along with dibromomethane (70.00 mg, 28.00 µL, 1.00 Eq, 0.400 mmol). Trifluoroacetic acid (456 mg, 306 µL, 10 Eq, 4.00 mmol) was added to the cathode compartment

and both sides of the cell were stirred at 22 °C and electrolyzed under a constant current of 2.5 mA for 12.9 h (3 F/mol alkene), using a Pine Wavenow Potentiostat with a (reference electrode Ag/AgNO3). At each time point, 25 µL aliquots were removed and diluted in CD_3CN for quantitative ^{1}H-NMR analysis (10 second relaxation delay between pulses).

Discussion: Consistent with our previous mechanistic studies in terminal alkene thianthrenation (*Nature* **2021**, *596*, 74–79), we found that standard constant current conditions operate above the requisite voltage to oxidize TT to $TT^{\cdot+}$, but below the voltage required to oxidize $TT^{\cdot+}$ to TT^{2+}. This is consistent with generation of TT^{2+} *via* an uphill disproportionation of $TT^{\cdot+}$, followed by rapid and favorable trapping of TT^{2+} by [4+2] cyclization with the internal alkene. We observe the *mono*-thianthrenium adduct as the exclusive alkene-derived product of this transformation.

Discussion of stereoretentive adduct formation and elimination.

Discussion. Our working mechanistic model for *mono*-thianthrenium-adduct formation involves a [4+2] inverse electron-demand cycloaddition between TT^{2+} and the alkene. This concerted process retains the stereochemistry of the alkene, resulting in thianthrenium adducts in which the alkyl groups are either *trans* or *cis* to one another. Because rotation about the C–C bond of the *mono*-thianthrenium adduct is not possible, the relationship of the alkyl groups is maintained upon elimination, resulting in a stereoretentive thianthrenation. This proposal is consistent with the rationale for stereoretentive chemical thianthrenation previously advanced by Ritter and coworkers (*Angew. Chem. Int. Ed.* **2020**, *59*, 14, 5616-5620).

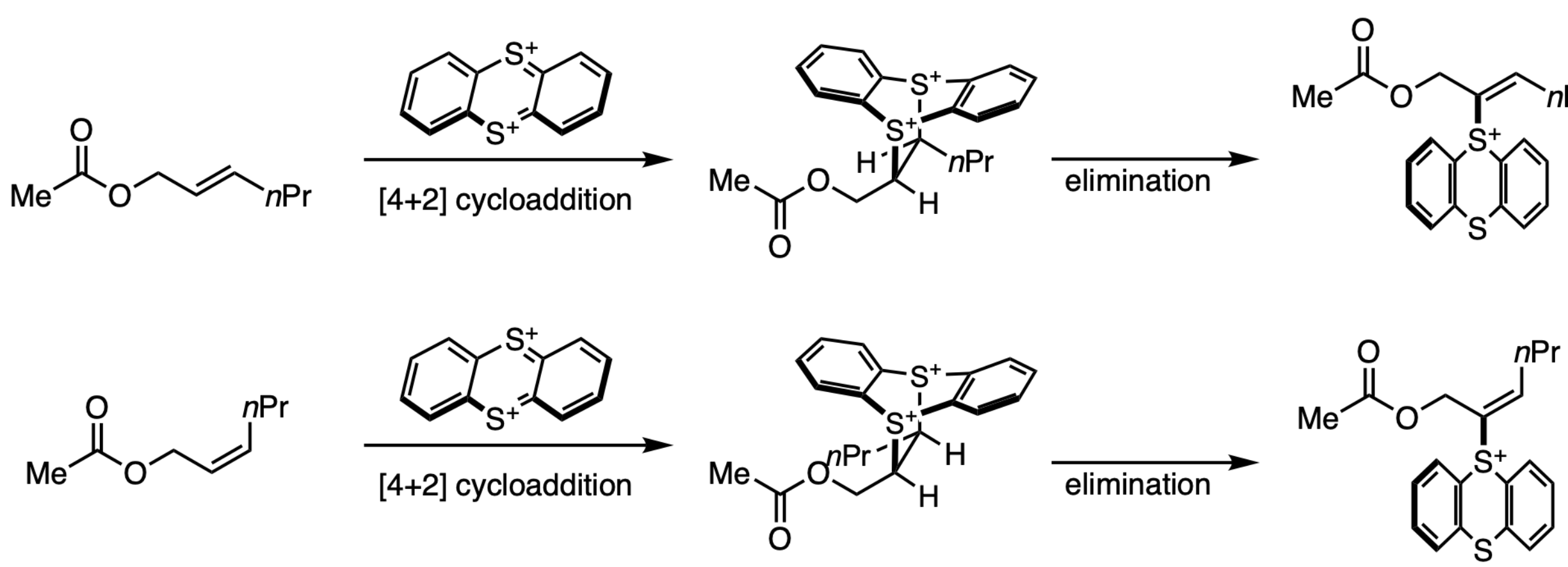

To validate the stereoretentive nature of the electrochemical thianthrenation of unsymmetrical alkenes that are the focus of the present study, we subjected both (*E)*- and (*Z*)-hex-2-en-1-yl acetate to the thianthrenation conditions. Both stereoisomers were thianthrenated stereoretentively.

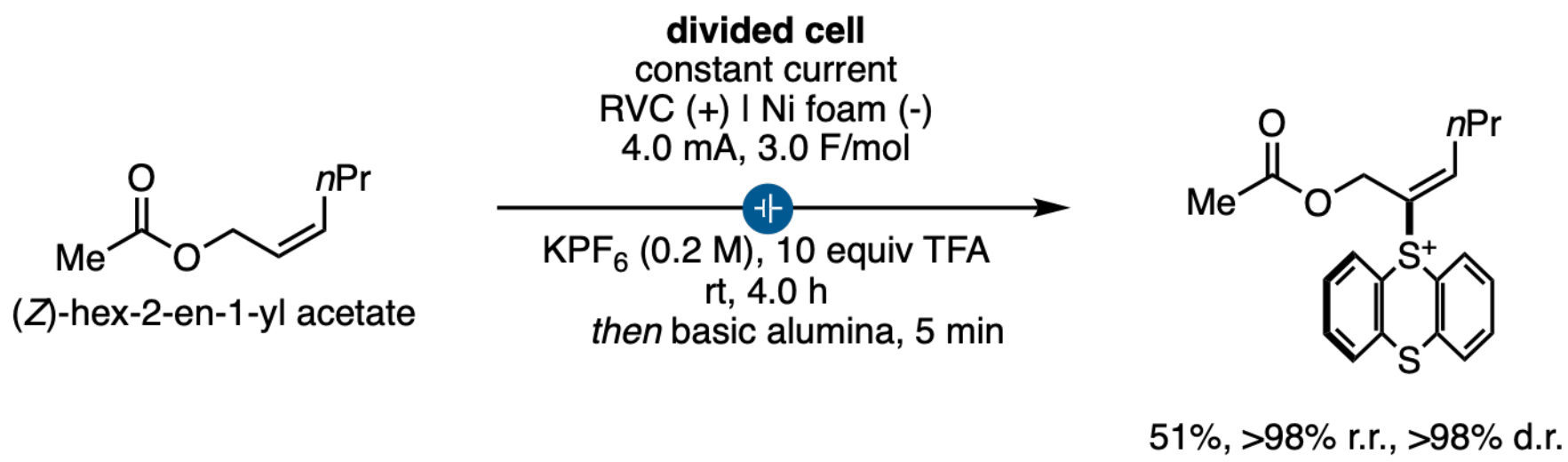

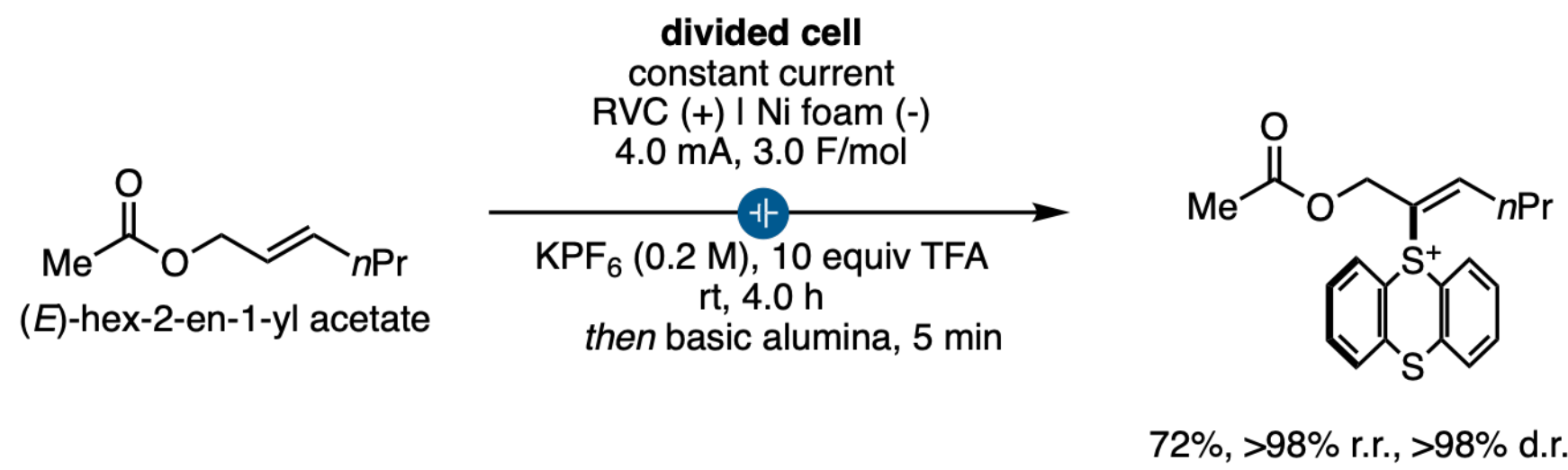

Procedures for determining elimination regioselectivity.

To study the electronic sensitivity of thianthrenium adduct elimination, we prepared thianthrenium adducts from several alkenes. The thianthrenium adduct from *trans*-2-octene was prepared using chemical thianthrenation, due to the high selectivity of this method for generating *mono*-thianthrenium adducts over *bis*-thianthrenium adducts.

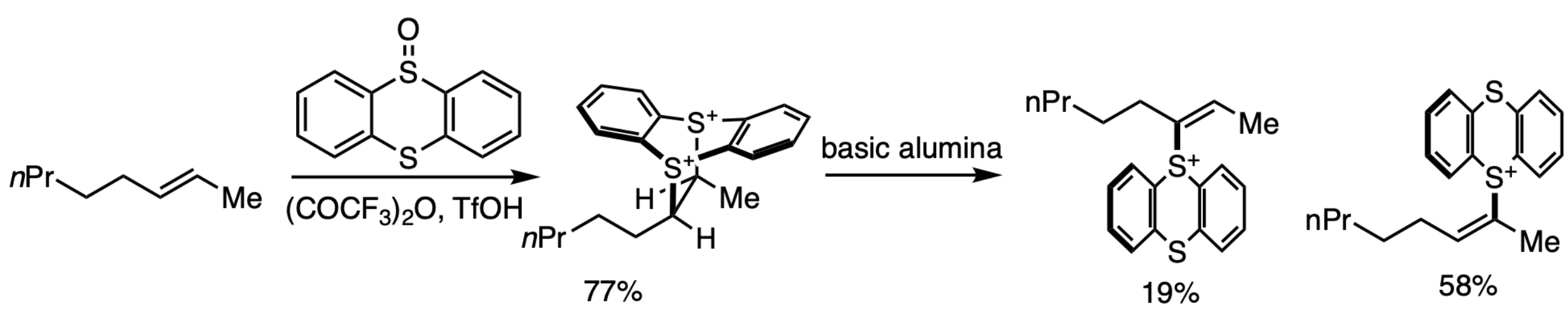

Procedure for determining regioselectivity: Adapted from a procedure reported by Ritter and coworkers (*Angew. Chem. Int. Ed.* **2020**, *59*, 14, 5616-5620). Under ambient atmosphere, a 2-dram vial equipped with a magnetic stir bar was charged with thianthrene *S*-oxide (47.9 mg, 0.206 mmol, 1.03 Eq), anhydrous MeCN (0.80 mL), and (E)-oct-2-ene (31.3 µL, 0.200 mmol, 1.00 Eq). After cooling to 0°C, trifluoroacetic anhydride (84.7 µL, 0.600 mmol, 3.0 Eq) was added dropwise, followed by dropwise addition of HOTf (21.3 µL, 0.240 mmol, 1.2 Eq). The contents were stirred for 30 minutes at 0°C, then for 3 h at room temperature. Dibromomethane standard (14.0 µL, 0.20 mmol, 1.0 Eq) was added to the solution. After stirring for 1 min, a 25-µL aliquot was removed and diluted with CD_3CN in an NMR tube for quantitative ^{1}H-NMR analysis (10-second relaxation delay between pulses). The mixture was then diluted with MeCN (10 mL), and alumina (6g, basic, Brockmann II) was added. After stirring the suspension for 30 minutes, another 25 µL aliquot was removed and diluted with CD_3CN in an NMR tube for quantitative ^{1}H-NMR analysis (10-second relaxation delay between pulses). Then, the solids were removed by vacuum filtration, rinsing the alumina pad with MeCN (2 x 5 mL), taking care to fully disperse the alumina in solution using a

spatula. From the filtrate, MeCN was removed in vacuo. Reported selectivity ratio was determined by ^{1}H-NMR analysis of the crude mixture. See details below regarding assignment of major regioisomer.

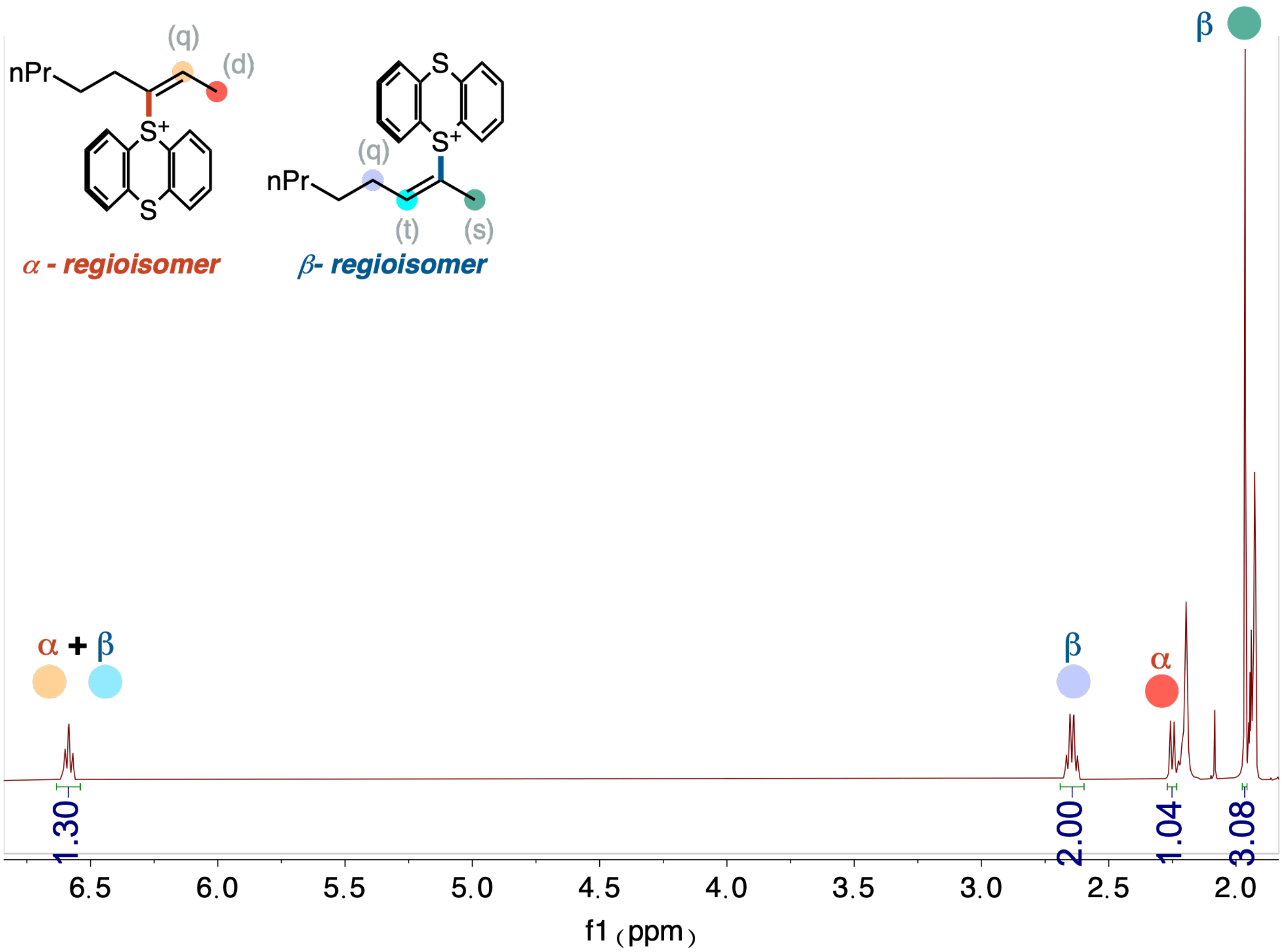

Figure S6. Determination of major regioisomer of thianthrenation of *trans*-2-octene. Ratio was determined based on relative integrations of methyl peaks on each regioisomer, and corroborated with aromatic peaks of the thianthrenium units.

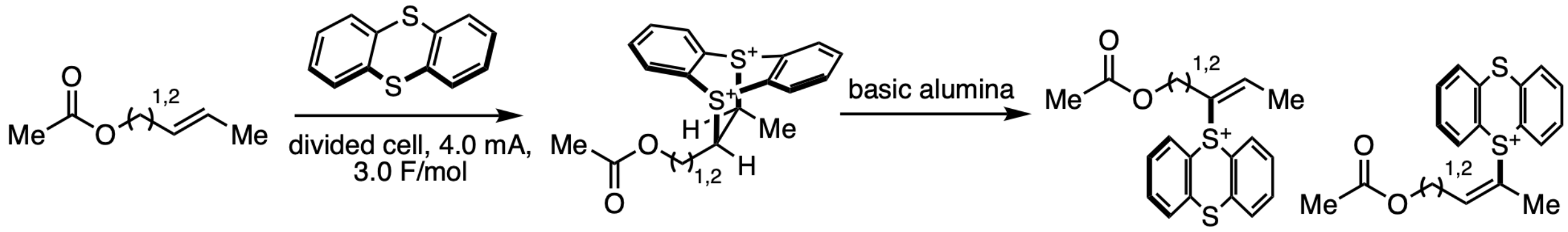

Procedure for determining regioselectivity. To an oven-dried divided electrochemical cell equipped with magnetic stir bars was added thianthrene (64.9 mg, 1.5 Eq, 0.300 mmol) to the anode compartment and dried KPF_6 (147 mg, 4 Eq, 0.800 mmol) to both compartments. The cell was equipped with two septa containing a stainless-steel wire/Ni foam cathode assembly (1.5 x 0.8 cm) and a pencil/RVC anode assembly (5 × 3 × 3 mm) connected with a Teflon tubing to equalize pressure. MeCN (4.0 mL) was added to the cathode compartment, and the glass frit was allowed to become saturated (<1 min). MeCN (4.0 mL) was added to the anode compartment, followed by alkene (E)-pent-3-en-1-yl acetate (25.6 mg, 1 Eq, 0.200 mmol) (electrode depth: 1 cm). TFA (228 mg, 154 µL, 10 Eq, 2.00 mmol) was added to the cathode compartment and both sides of the cell were stirred at room temperature and electrolyzed under a constant current of 4.0 mA for 4.0 h (3.0 F/mol alkene). Upon completion of the electrolysis, dibromomethane standard (14.0 µL, 0.200 mmol, 1.0 equiv.) was added to the anodic compartment. After stirring for 1 min, a 25-µL aliquot was removed and diluted with CD_3CN in an NMR tube for quantitative ^{1}H-NMR analysis (10-second relaxation delay between pulses). The anode solution was transferred to a 50 mL round-bottom flask equipped with a magnetic stir bar and the anode was rinsed with MeCN (5 x 0.50 mL). Alumina (5 g, activated, basic, Brockmann II) was added to the anode solution. After stirring the suspension for 15 minutes, another 25-µL aliquot was removed and diluted with CD_3CN in an NMR tube for quantitative ^{1}H-NMR analysis (10-second relaxation delay between pulses). Then, the solids were removed by vacuum filtration, rinsing the alumina pad with MeCN (2 x 5 mL), taking care to fully disperse the alumina in solution using a spatula. From the filtrate, MeCN was removed in vacuo. Reported selectivity ratio was determined by ^{1}H-NMR analysis of the crude mixture. See details below regarding assignment of major regioisomer.

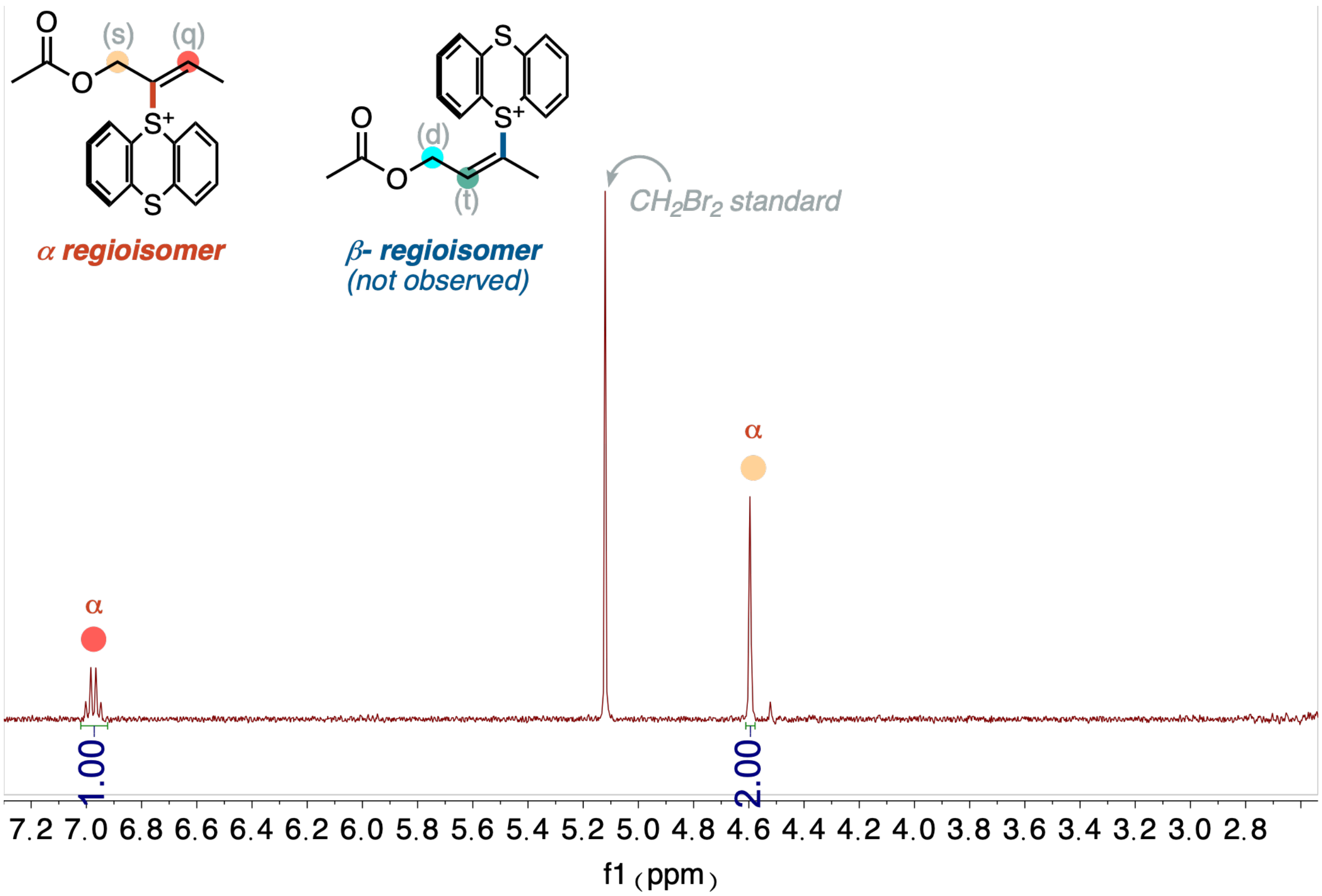

Figure S7. Determination of major regioisomer of thianthrenation of (E)-but-2-en-1-yl acetate. Regioisomer 1 is the only observed product. For all substrates bearing allylic functional groups, regioselectivity was determined based on the splitting of the allylic signals.

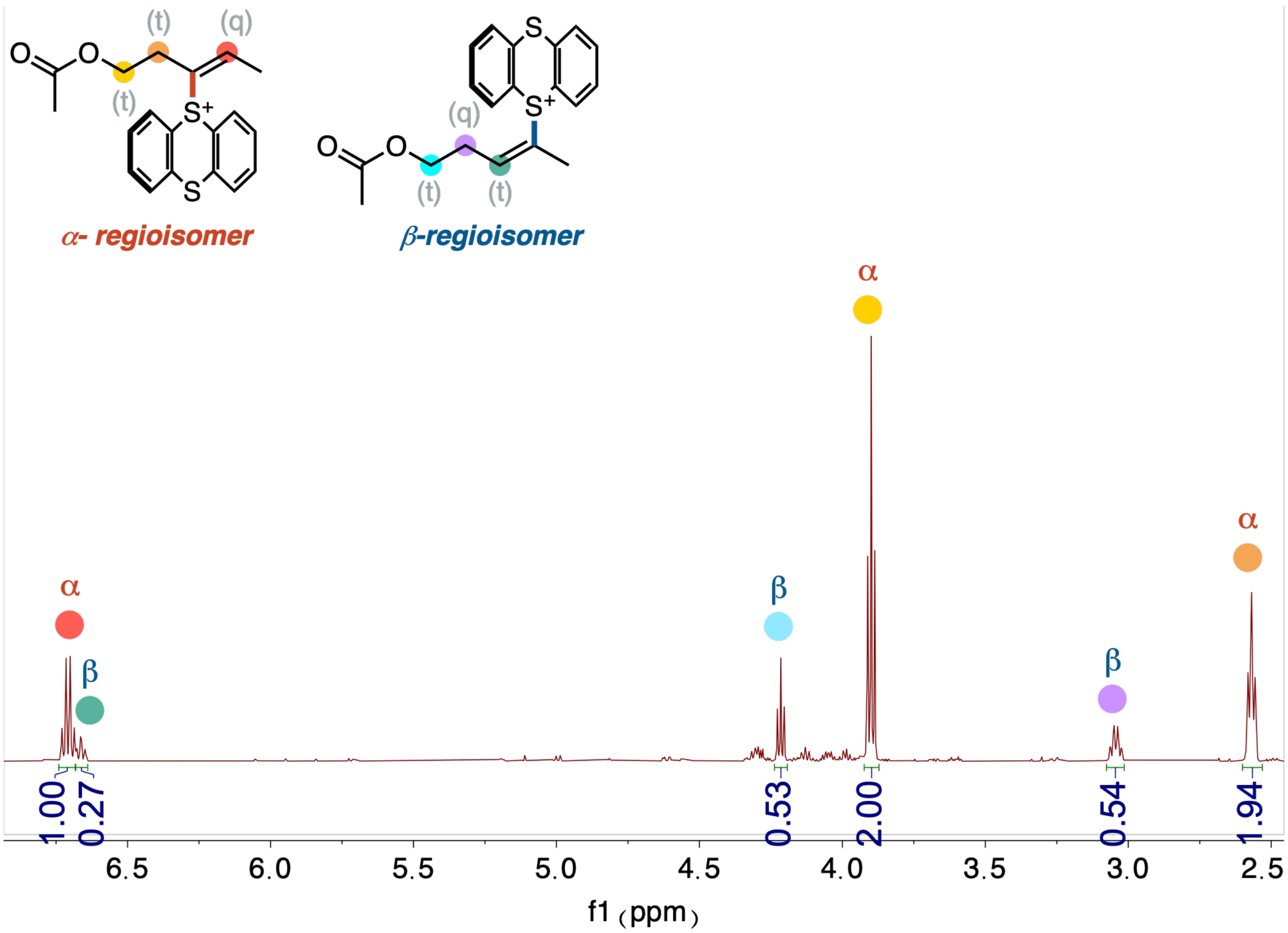

Figure S8. Determination of major regioisomer of thianthrenation of (*E*)-pent-3-en-1-yl acetate. Ratio was determined based on relative integrations of both allylic peaks on each regioisomer. Ratios were corroborated with the allylic peaks on both regioisomers.

Optimization of electrochemical thianthrenium adduct formation.

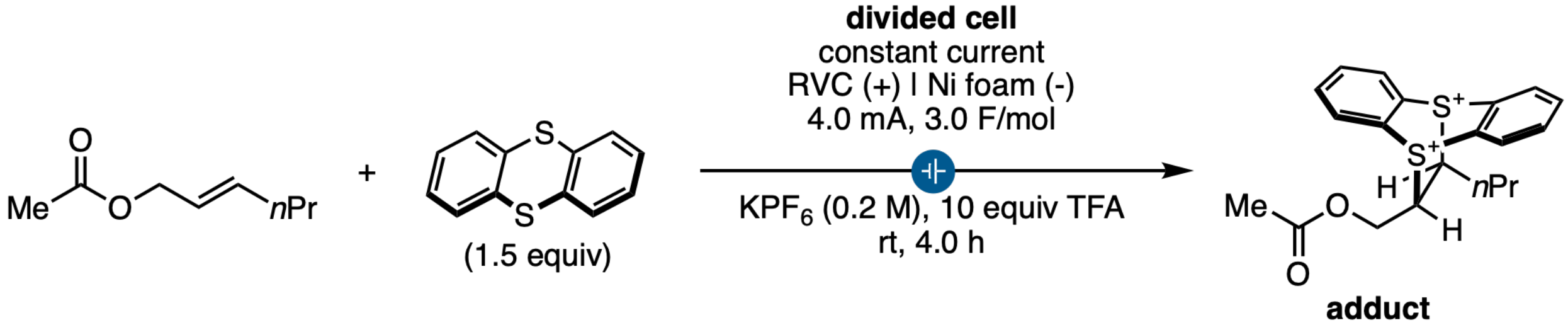

changes from conditions	% yield adduct	% RSM alkene
none	72%	0%
CWP @ 1.1 V vs. Fc/Fc^+ (6 h)	66%	0%
1.5x electrode size	70%	0%
3 equiv. TT	12%	58%
8.5 mA, 2.5 F/mol	50%	18%
undivided cell electrolysis*, 12 mA, 8 F/mol	0%	65%

Table S1. Optimization of electrochemical thianthrenium adduct formation.
*undivided cell electrolysis conditions adapted from *Science* **2025**, *389*, 6766, 1239-1245.

Discussion. The standard conditions for generating thianthrenium adducts from internal alkenes involve constant current electrolysis of 1.5 equiv. thianthrene in a divided cell. Constant working potential electrolysis between the oxidation potential of TT and $TT^{\cdot+}$ is comparably effective to constant current electrolysis. Decreasing the current density made no measurable impact on reaction outcome. Consistent with our mechanistic model invoking disproportionation of $TT^{\cdot+}$ as a key step in adduct generation, increasing the amount of neutral thianthrene, which participates in comproportionation with TT^{2+}, dramatically slows the reaction. We found that rapid constant current electrolysis resulted in incomplete and irreproducible alkene conversion, likely due to high cell resistivity. Undivided cell electrolysis resulted in unproductive alkene consumption and no generation of alkene-thianthrenium adducts or the corresponding alkenyl thianthrenium salts.

Regioselectivity of chemical thianthrenation.

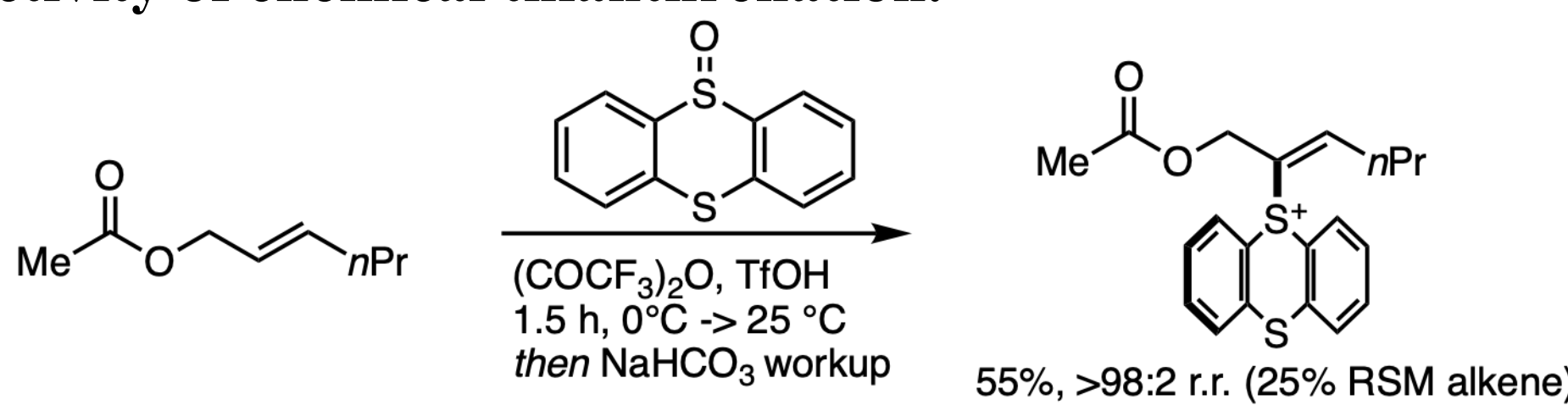

Procedure. Adapted from a procedure reported by Ritter and coworkers (*Angew. Chem. Int. Ed.* **2020**, *59*, 14, 5616-5620). Under ambient atmosphere, a 100 mL round-bottom flask equipped with a magnetic stir bar was charged with thianthrene 5-oxide (120 mg, 1.03 Eq, 0.515 mmol), (E)-hex-2-en-1-yl acetate (71.1 mg, 79.2 µL, 1 Eq, 0.500 mmol), and MeCN (2.00 mL) (0.25M). After cooling to 0 °C, 2,2,2-trifluoroacetic anhydride (315 mg, 209 µL, 3 Eq, 1.50 mmol) was added dropwise followed by dropwise addition of trifluoromethanesulfonic acid (90.0 mg, 53.6 µL, 1.2 Eq, 0.600 mmol) within 10 seconds. After stirring the deep blue mixture at 0 °C for 60 min followed by stirring at room temperature (~25 °C) for 30 min, the resulting light purple mixture was concentrated under reduced pressure and subsequently diluted with DCM (~30 mL). Saturated aqueous $NaHCO_3$ solution (~20 mL) was slowly poured into the flask to quench the dicationic adducts and acids. The combined mixture was poured into a separatory funnel and the layers were separated. The aqueous layer was further extracted with DCM (2 x 30 mL). The combined DCM solution was then washed with 5% wt/wt aq. KPF_6 (30 mL). The DCM layer was collected, dried over Na_2SO_4, filtered, and the solvent was removed under reduced pressure. The resulting crude mixture was dissolved in $CDCl_3$, dibromomethane (70.0 mg, 28.0 µL, 0.80 Eq, 0.40 mmol) standard was added and stirred for 10 sec. A 25 µL aliquot was removed and diluted in $CDCl_3$ for quantitative ^{1}H-NMR analysis.

Discussion. This unoptimized result demonstrates that trisubstituted alkenyl thianthrenium salts can be regioselectively prepared using chemical thianthrenation conditions reported by Ritter and coworkers.

Screening of bases for adduct elimination.

Procedure. To an oven-dried divided electrochemical H-cell equipped with magnetic stir bars was added thianthrene (130 mg, 1.5 Eq, 0.600 mmol) to the anode compartment and dried KPF_6 (294 mg, 4 Eq, 1.60 mmol) to both compartments. The cell was equipped with two septa containing a stainless-steel wire/Ni foam cathode assembly (1.0 x 1.0 cm) and a pencil/RVC anode assembly (8 × 5 × 5 mm) connected with Teflon tubing to equalize pressure. MeCN (8.0 mL) was added to the cathode compartment and the glass frit was allowed to become saturated (<1 min). MeCN (8.0 mL) was added to the anode compartment, followed by alkene (1 Eq, 0.400 mmol). (Electrode depth: 1 cm). Trifluoroacetic acid (456 mg, 306 μL, 10 Eq, 4.00 mmol) was added to the cathode compartment and both sides of the cell were stirred at room temperature and electrolyzed under a constant current of 8.0 mA for 4.00 h (3.0 F/mol alkene). Upon completion of electrolysis, CH_2Br_2 standard (14 μL, 0.2 mmol, 1.0 equiv.) was added. After stirring for 60 sec, a 25-μL aliquot was removed and diluted in CD_3CN for ^{1}H-NMR analysis. The anodically generated mixture was transferred to a 20-mL scintillation vial equipped with a magnetic stir bar, and the base was added and stirred for the specified amount of time. Upon completion, the yield and stereoselectivity were determined by quantitative NMR analysis in CD_3CN (10 sec relaxation delay between pulses).

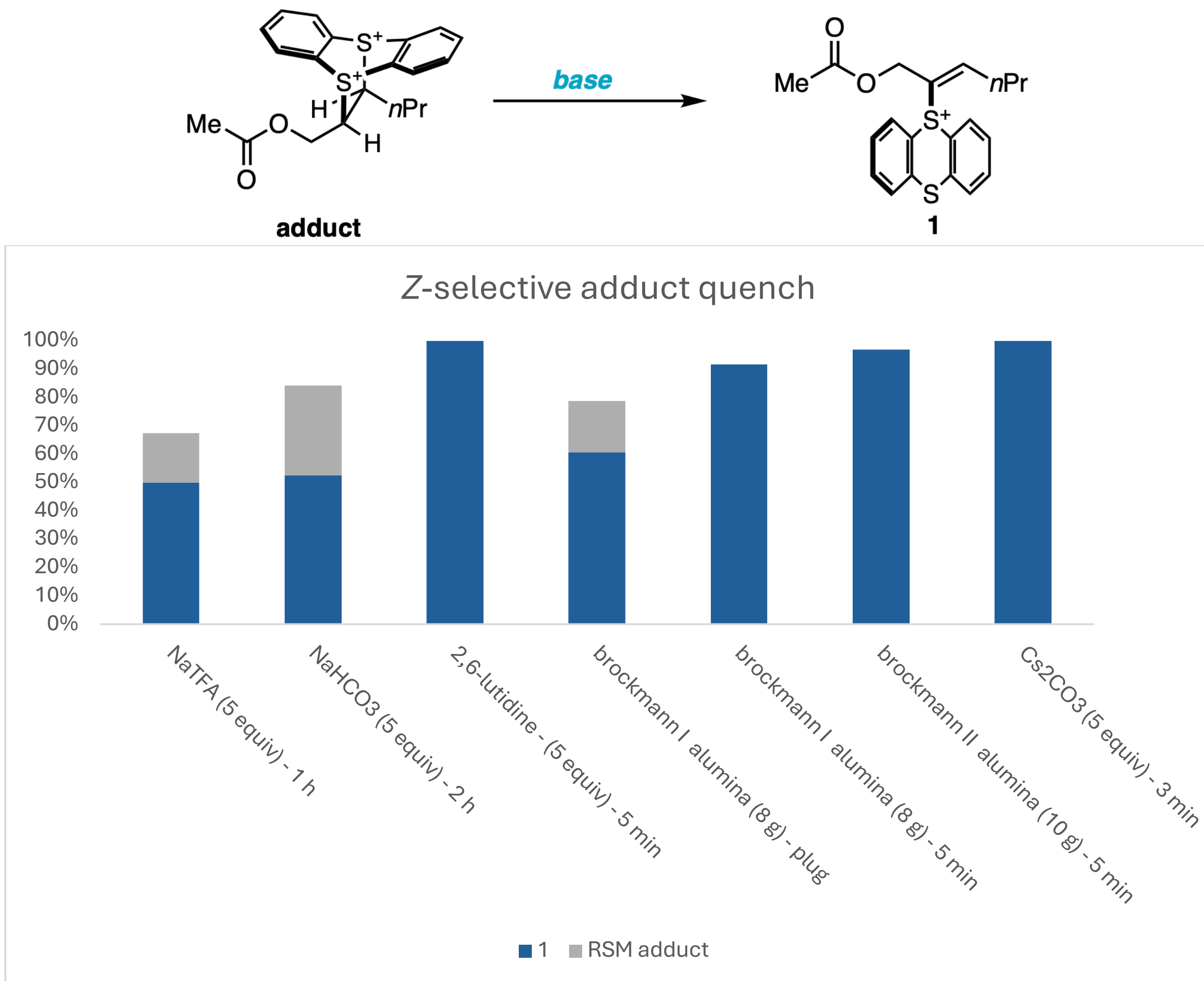

Table S2. Z-selective adduct quenching conditions. In all cases, stereoselectivity and regioselectivity were >98:2.

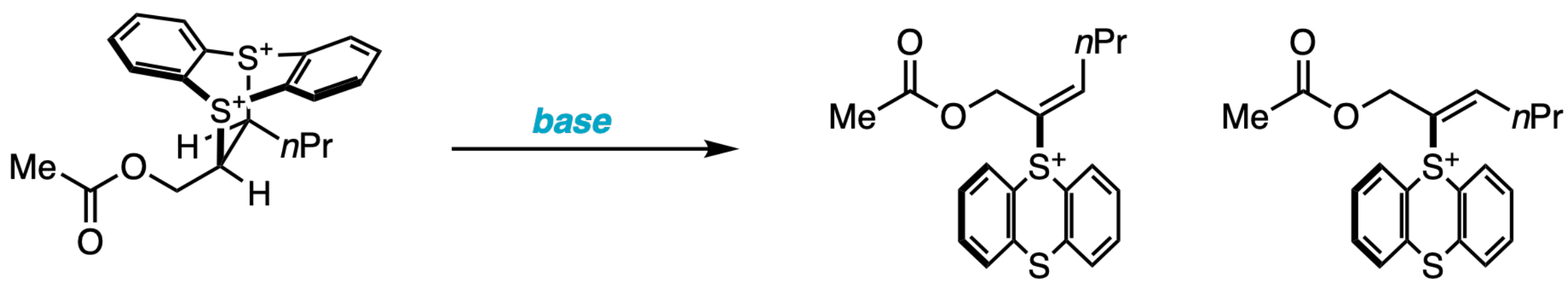

adduct **12** **1**

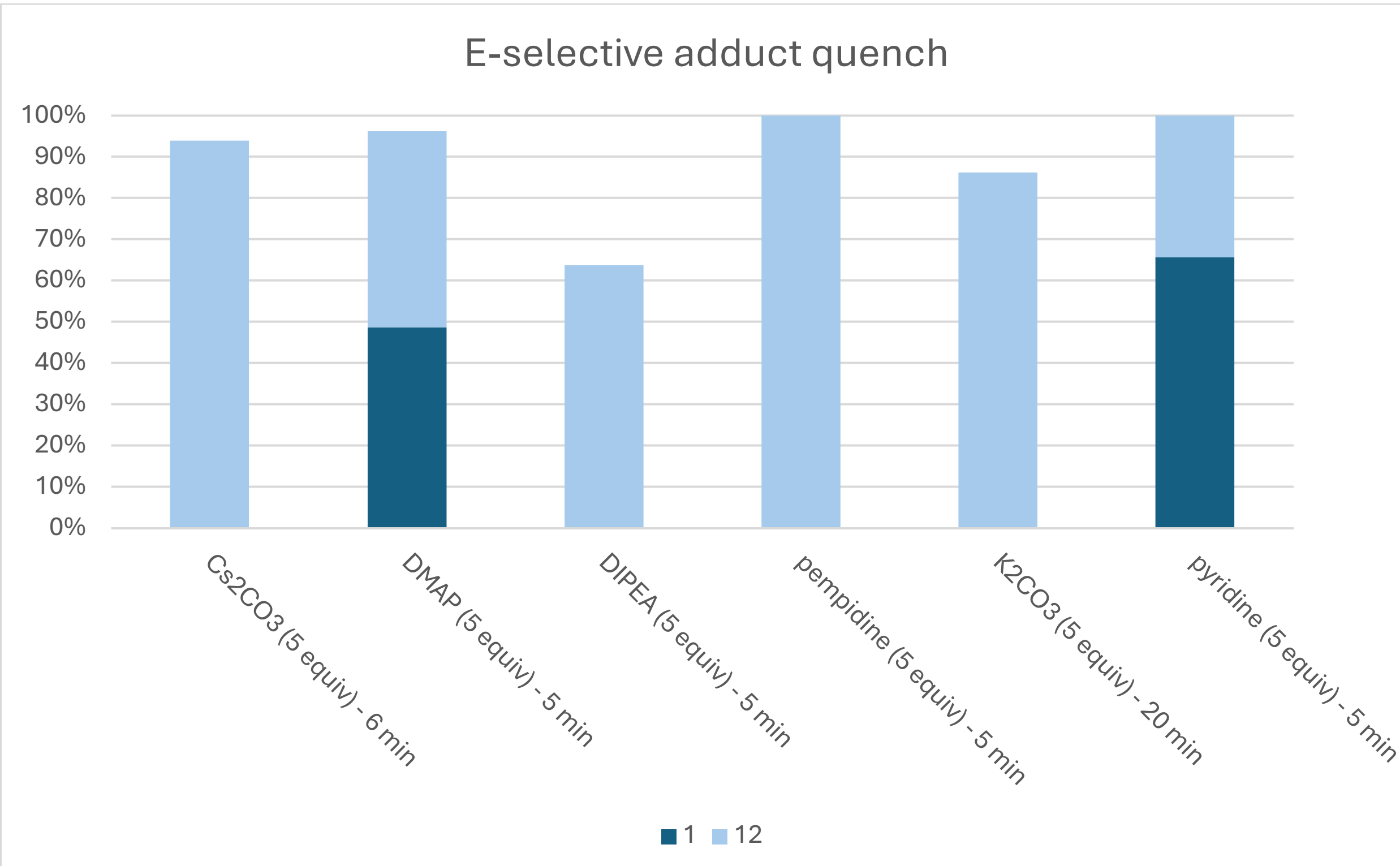

Table S3. E-*selective adduct quenching conditions.* In all cases, regioselectivity was >98:2.

Discussion. Several bases can effectively quench the anodically generated thianthrenium adduct mixture to provide high yields of alkenyl thianthrenium salts. In general, weaker or less-nucleophilic bases provided the kinetically favorable *Z*-alkenyl thianthrenium salts upon adduct elimination. Due to its operational simplicity, we selected a basic alumina quench for our optimized *Z*-selective conditions. We found that slightly deactivated Brockmann II basic alumina, prepared by addition of water to Brockmann I basic alumina, provided improved mass balance and reproducibility in the adduct elimination. Several amine bases provided *E*-selective elimination; however, we found that these bases complicated purification and led to product decomposition over time. Thus, we selected inorganic bases K_2CO_3 and Cs_2CO_3, as the optimal bases. Due to its improved solubility in MeCN, Cs_2CO_3 resulted in more rapid and general isomerization, and was thus selected as the optimal base for *E*-selective thianthrenation conditions. We found that we

could use sub-stoichiometric amounts of Cs_2CO_3 to promote isomerization of the *Z*-alkenyl thianthrenium salts. This provided high reproducibility and prevented base-induced byproduct formation.

Impact of base identity on regioselectivity.

To probe the impact of base identity on regioselectivity, we studied the regioselectivity of elimination for substrates which provided detectable amounts of both regioisomers.

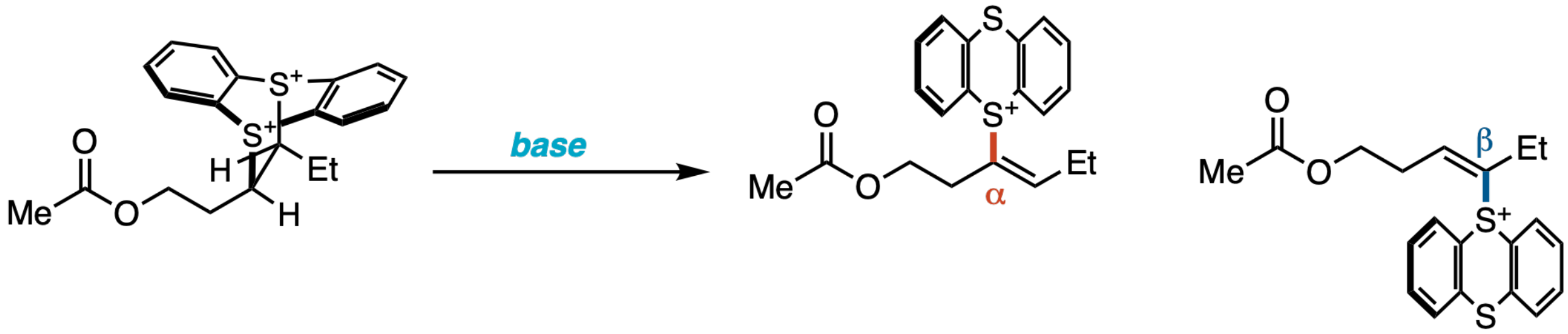

base	RSM adduct	yield	α:β
alumina (5 g)	0%	100%	5.6:1
Cs_2CO_3 (5 equiv.) - 10 min	0%	100%	3.2:1
KOtBu (5 equiv.) - 3 min	0%	46%	2.5:1
pyridine (5 equiv.) - 30 min	67%	0%	n.d.

Table S4. Yield and regioselectivity of adduct elimination of (E)-hex-3-en-1-yl acetate. Yields and regioselectivities determined by quantitative ^{1}H-NMR with dibromomethane as an internal standard.

Discussion. We observed a dependence of regioselectivity on base identity. In general, weaker bases provided higher regioselectivity whereas stronger bases led to diminished selectivity and lower yields. The alkene bearing a homoallylic acetate was more sensitive to base identity. We hypothesize that the electronic sensitivity of regioselectivity depends on the magnitude of negative charge buildup in the elimination transition state. Because elimination reactions involving weaker bases incur greater charge buildup in the transition state, these eliminations are more sensitive to electronic effects and provide higher regioselectivity.

Probing steric sensitivity of isomerization.

Procedure. To a 2-dram vial equipped with a magnetic stir bar was added **1** (alkenyl thianthrenium salt, 0.100 mmol). The solid was dissolved in anhydrous MeCN (1 mL), and CH_2Br_2 (1 equiv) was added as an internal standard. Base (1 equiv.) was added, and the reaction was stirred for 4 h. At each time point, a 25 µL aliquot was removed and dissolved in CD_3CN for quantitative NMR analysis (10 second relaxation delay between pulses).

Discussion. We observed that the sterically unhindered pyridine facilitated rapid *Z*- to *E*-isomerization. However, the *E*-alkenyl thianthrenium salt **12** disappeared in the presence of pyridine over time. Isomerization in the presence of 2-picoline was observed but was significantly slower. Finally, DTBMP did not facilitate isomerization. These data are consistent with an isomerization that is sensitive to the steric bulk of the participating nucleophile.

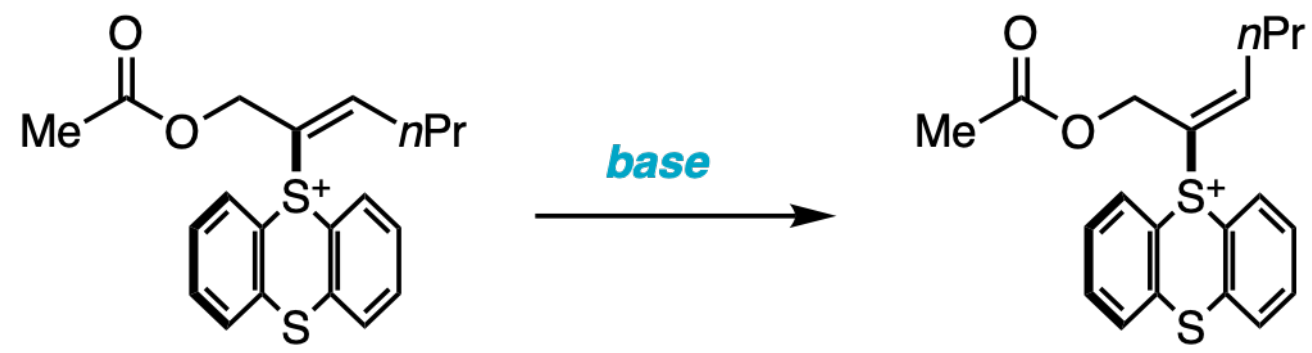

1 12

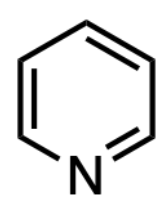

**time** (h)	**1** (%)	**12** (%)
0	100	0
0.5	90	10
1	0	86
3	0	77

Table S5. Isomerization with pyridine.

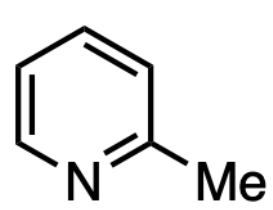

**time** (h)	**1** (%)	**12** (%)
0	100	0
0.5	94	6
1	79	17
3	65	29

Table S6. Isomerization with 2-picoline.

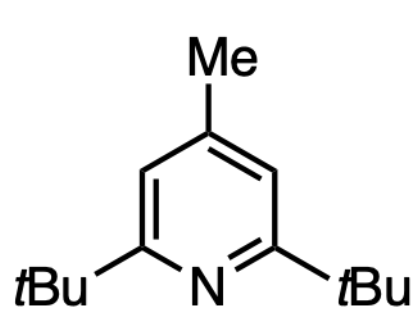

**time** (h)	**1** (%)	**12** (%)
0	100	0
0.5	100	0
1	100	0
3	100	0

Table S7. Isomerization with DTBMP.

Probing the deuteration of reaction intermediate.

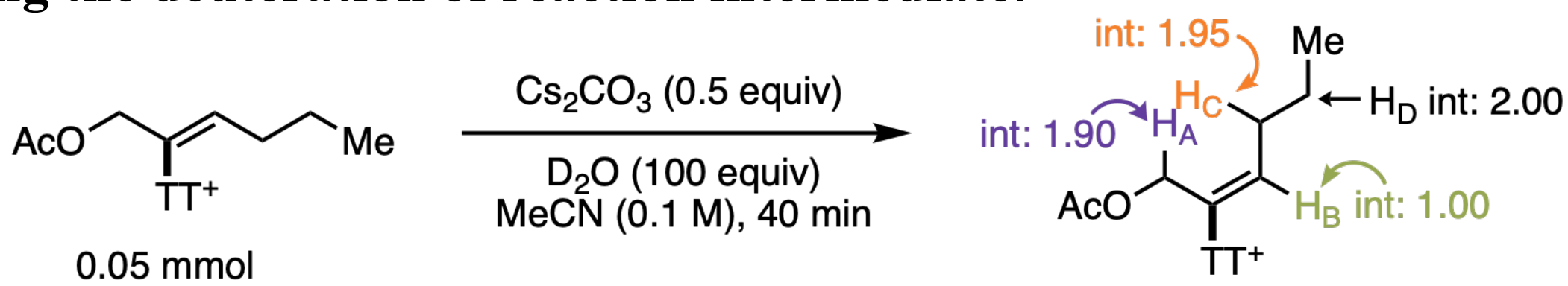

Procedure. A 1-dram vial equipped with a magnetic stir bar was charged with **1** (25.0 mg, 0.05 mmol, 1.0 equiv) and dissolved in anhydrous MeCN (1 mL). D_2O (90 µL, 5.0 mmol, 100 equiv.) was added, followed by Cs_2CO_3 (8.11 mg, 0.50 equiv., 0.025 mmol). The contents were stirred for 40 min, at which point CH_2Br_2 was added (1 equiv.) and the contents were stirred for 1 min. A 25-µL aliquot was removed and dissolved in CD_3CN for quantitative NMR analysis (10 sec relaxation delay between pulses). The product was isolated for analytical characterization according to the workup in **General Procedure C** followed by flash chromatography with C2 silica.

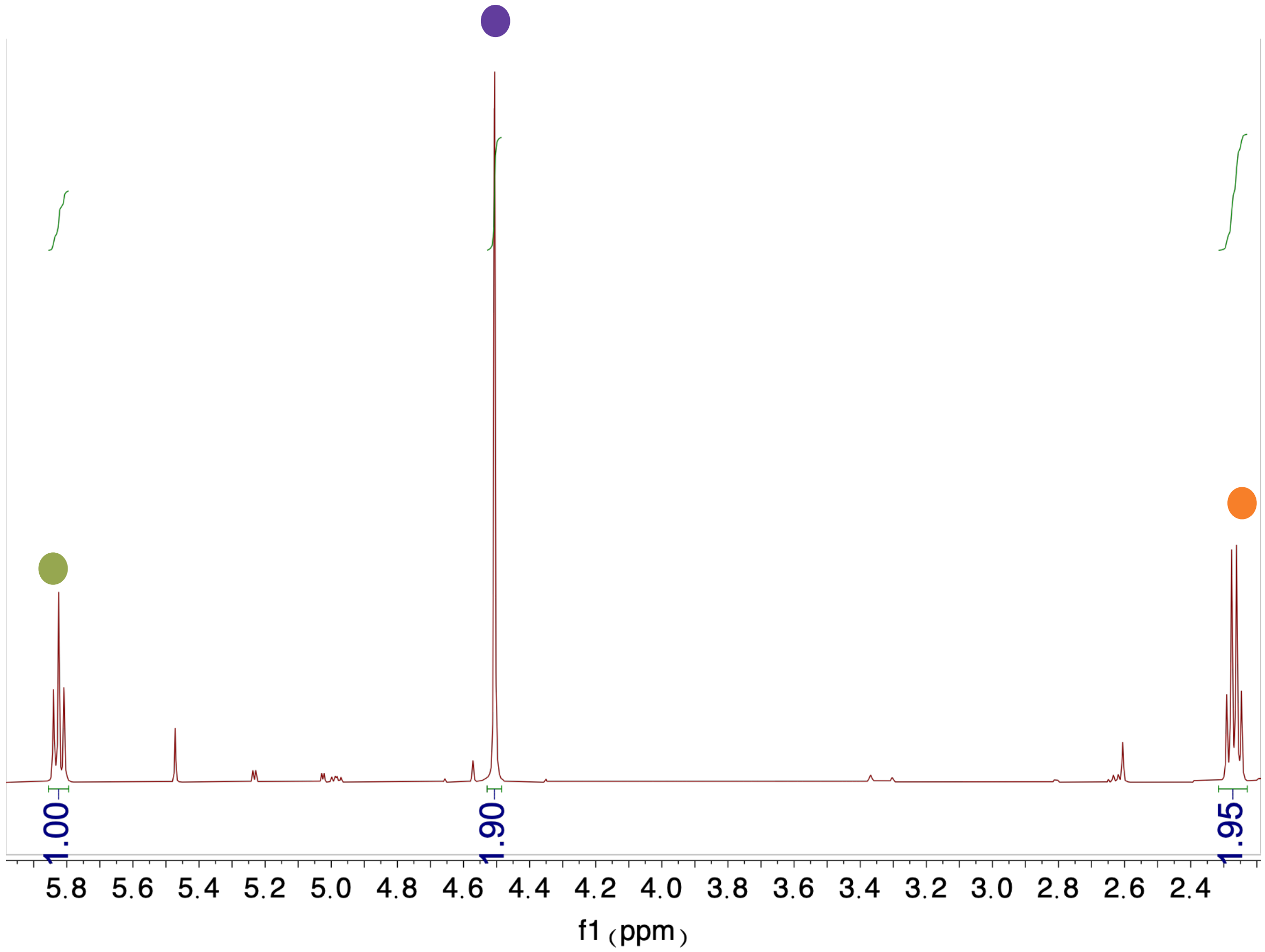

Figure S9. Example ^{1}H-NMR spectrum of isomerized E-alkenyl thianthrenium product in presence of D_2O. The alkenyl (H_B) and homoallylic (H_D) protons were used for validation and comparison to determine percent loss in proton signal of potentially reactive sites.

Isomerization time course.

The thianthrenium adduct was prepared according to **General Procedure A.** Upon completion of electrolysis, the anodic mixture was transferred to an oven-dried round-bottom flask equipped with a magnetic stir bar. The anode and electrode were rinsed with anhydrous MeCN (5 x 1.5 mL) to ensure quantitative transfer. CH_2Br_2 (1.0 equiv, 0.4 mmol, 28 μL) was added as an internal standard. A 25-μL aliquot was taken and diluted in CD_3CN for quantitative NMR analysis (10 sec relaxation delay between pulses). Then, dried Cs_2CO_3 (5.0 equiv, 2 mmol, 652 mg) was added and the mixture was stirred vigorously for 15 min. At each time-point, a 25-μL aliquot was removed and diluted in CD_3CN for NMR analysis (10 sec relaxation delay between pulses).

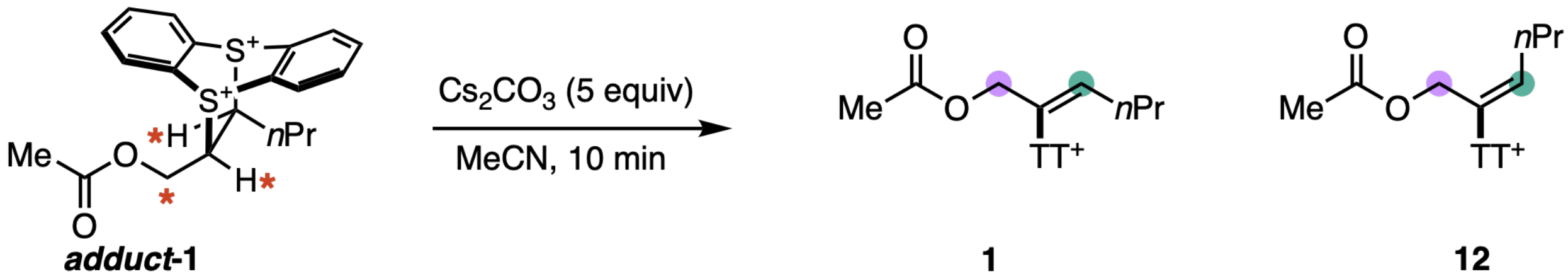

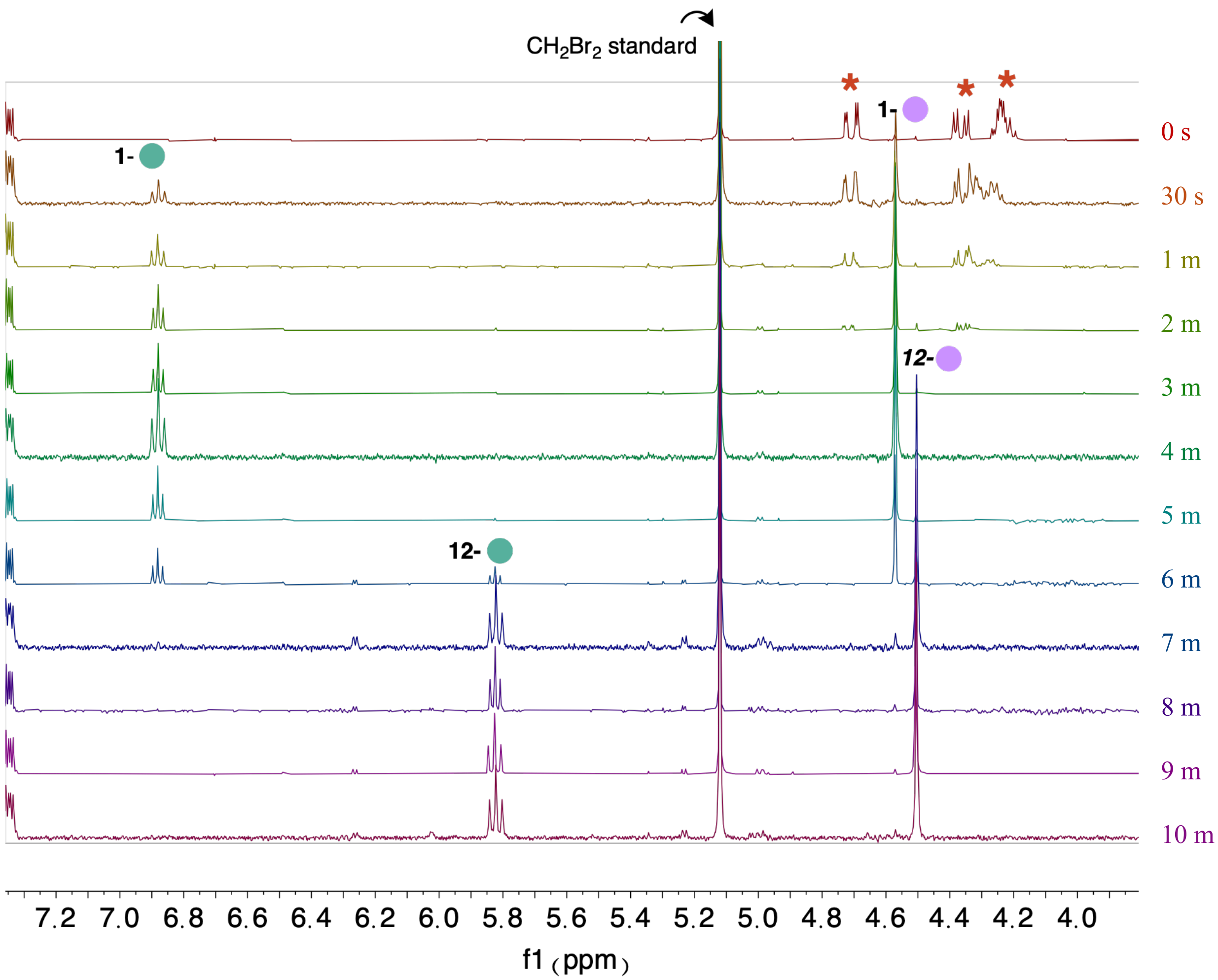

Figure S10. Isomerization time course.

Investigating electrophile stability under cross coupling conditions.

A time course of the Suzuki reaction revealed that the cross coupling of compound **5** occurs rapidly (full conversion within 30 min). However, in the absence of the Pd catalyst and cross coupling partner, compound **5** undergoes rapid isomerization and decomposition under the reaction conditions. These data demonstrate that the stereoretention is a result of the cross coupling outcompeting Cs_2CO_3-induced isomerization.

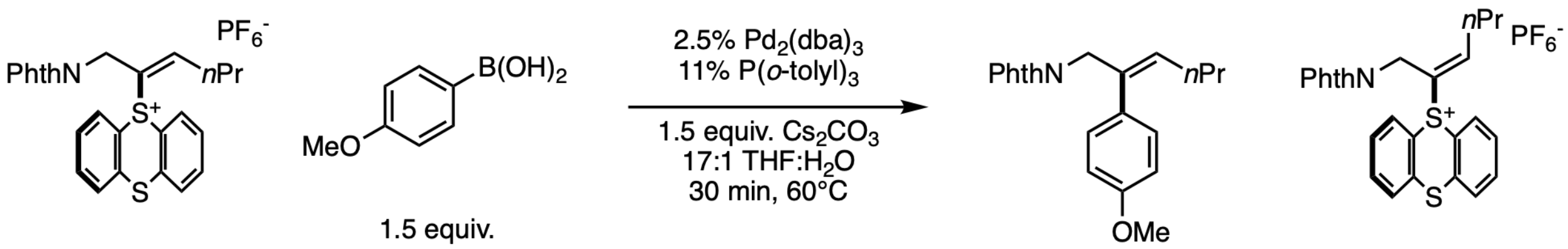

5 **22** **20**

time (min)	5 (%)	22 (%)	20 (%)
0.5	-	18	0
10	-	82	0
30	-	97	0

*Table S8. Time course of Suzuki reaction. NMR peaks of **5** were obscured by other peaks in the mixture preventing its quantification. Yield determined by ^{1}H-NMR using 1,3,5-trimethoxybenzene as standard.*

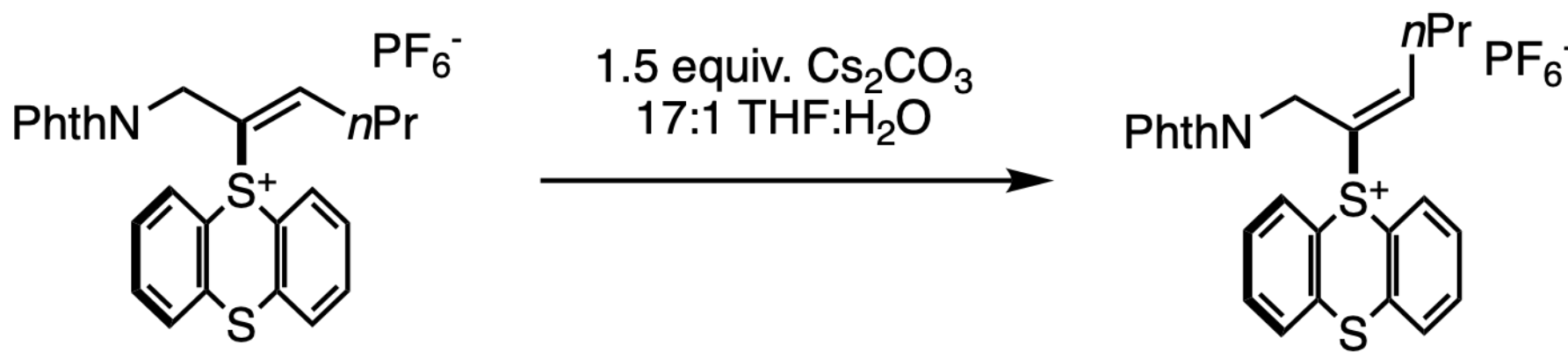

5 **20**

time (min)	5 (%)	20 (%)
10	74	12
60	0	87
5 hour	0	0

Table S9. Time course of decomposition under Suzuki conditions. Yield determined by ^{1}H-NMR using 1,3,5-trimethoxybenzene as standard.

3. Substrate preparation and characterization

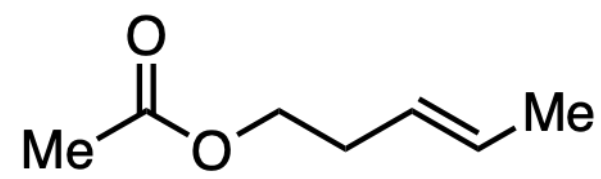

(*E*)-pent-3-en-1-yl acetate: To a flame-dried 100 mL round-bottom flask equipped with a magnetic stir bar was added $LiAlH_4$ (1.92 g, 50.60 mmol, 1.00 eq.) and the vessel was evacuated and flushed with nitrogen (3x). Dry Et_2O (50.00 mL) was added, and the suspension was cooled to 0 °C. (*E*)-pent-3-enoic acid (5.00 mL, 50.60 mmol, 1.00 eq.) was carefully added in small portions. The reaction mixture was allowed to warm up to room temperature overnight. The reaction mixture was cooled to 0 °C again and was then quenched carefully with H_2O. When no hydrogen was released anymore, saturated aqueous Rochelle's salt (10 mL) was added to the reaction mixture and the suspension was stirred for 1 h. The phases were separated and the aqueous layer washed with Et_2O (30 mL). The organic layer was dried over $MgSO_4$, filtered, and the solvent was carefully removed in vacuo. The flask containing the crude mixture was equipped with a magnetic stir bar and anhydrous DCM (12 mL) was added. At room temperature, triethylamine (3.04 g, 4.18 mL, 2.00 Eq, 30.0 mmol) was added, followed by DMAP (91.6 mg, 0.05 Eq, 0.750 mmol). The flask was cooled in an ice bath and acetic anhydride (1.68 g, 1.56 mL, 1.1 Eq, 16.5 mmol) was added dropwise via syringe. The resulting solution was stirred at room temperature overnight. Upon completion, the reaction mixture was quenched with saturated $NaHCO_3$ (20 mL). The aqueous layer was combined and washed with DCM (3 x 15 mL). The combined organic layers were then washed with brine (30 mL), dried over Na_2SO_4, and filtered. The solvent was removed in vacuo, and product was loaded onto silica and isolated by flash chromatography (Et_2O/hexanes) to yield the title compound (772 mg, 6.02 mmol, 40.2%).^{1}H NMR (500 MHz, $CDCl_3$) δ 5.53 (dqt, J = 15.4, 6.2, 1.3 Hz, 1H), 5.39 (dtq, J = 15.1, 6.7, 1.6 Hz, 1H), 4.06 (t, J = 6.9 Hz, 2H), 2.30 (dddt, J = 8.2, 6.8, 5.6, 1.3 Hz, 2H), 2.04 (s, 3H), 1.66 (dq, J = 6.4, 1.4 Hz, 3H), consistent with reported spectra (*J. Chem. Ecol.* **2004**, *30*, 991–1000).

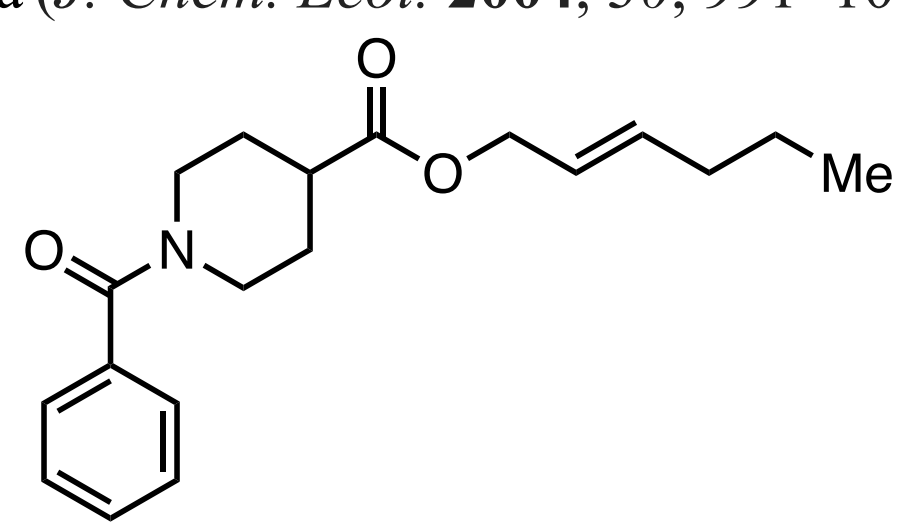

(*E*)-hex-2-en-1-yl 1-benzoylpiperidine-4-carboxylate: In a flame-dried 250 mL-bottom flask equipped with a magnetic stir bar, 1-benzoylpiperidine-4-carboxylic acid (1.96 g, 1.2 Eq, 8.40 mmol) was dissolved in anhydrous DCM (70.0 mL). At room temperature, (*E*)-hex-2-en-1-ol (701 mg, 1 Eq, 7.00 mmol) and DMAP (428 mg, 0.50 Eq, 3.50 mmol) were added. The mixture was cooled to 0 °C and 1-ethyl-3-(3-dimethylaminopropyl)carbodiimide (2.01 g, 1.5 Eq, 10.5 mmol) was added. The resulting solution was stirred overnight at rt. Upon completion, the solution was quenched with aqueous 1M HCl (30 mL). The layers were separated and the aqueous layer was extracted with DCM (3 × 30 mL). The combined organic layers were washed with brine (30 mL),

dried over Na_2SO_4, filtered, and concentrated in vacuo. The crude product was isolated by flash chromatography (ethyl acetate/hexanes) to yield the title compound (1.91 g, 6.06 mmol, 86.5 %). ^{1}H-NMR (500 MHz, $CDCl_3$) δ 7.42 (dd, *J* = 5.3, 1.7 Hz, 5H), 5.84 – 5.75 (m, 1H), 5.62 – 5.52 (m, 1H), 4.57 (m, *J* = 6.5 Hz, 3H), 3.77 (bs, 1H), 3.07 (bs, 2H), 2.61 (tt, *J* = 10.7, 4.0 Hz, 1H), 2.06 (q, *J* = 7.2 Hz, 2H), 1.76-2.15 (m, 4H), 1.44 (h, *J* = 7.4 Hz, 2H), 0.96 – 0.89 (m, 3H). ^{13}C NMR (126 MHz, $CDCl_3$) δ 173.93, 170.40, 136.64, 136.07, 129.61, 128.49, 126.84, 123.75, 65.46, 46.96 (bs), 41.49 (bs), 41.10, 34.29, 28.49 (bs), 28.05 (bs), 22.03, 13.62. **HRMS** (ESI+) calc: $[M+H]^+$ 316.1907, measured: 316.1904; difference = 0.9 ppm.

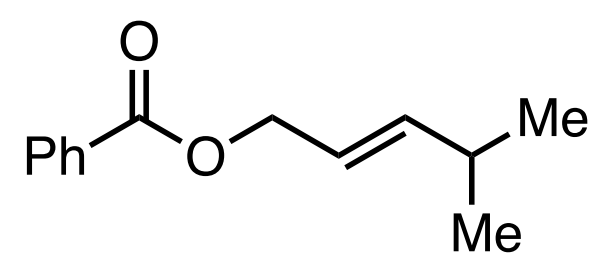

(*E*)-4-methylpent-2-en-1-yl benzoate: Adapted from a procedure reported by Mitsuya and coworkers (*Bioorg. Med. Chem*. **2011**, *19*, 13, 4028-4042). In a 100-mL round bottom flask equipped with a magnetic stir bar, (E)-4-methylpent-2-enoic acid (5.7 g, 1 Eq, 50 mmol) and MeOH (23 mL) were added. Concentrated sulfuric acid (4.9 g, 2.70 mL, 1 Eq, 50 mmol) was then slowly added at room temperature. The flask was equipped with a findenser and the mixture was stirred under reflux for 16 h. The solvent was then removed in vacuo. The resulting residue was slowly added to ice water (10 mL) and neutralized with saturated aqueous $NaHCO_3$. The layers were separated and the aqueous layer was extracted with ethyl acetate (2 x 30 mL). The combined organic layers were washed with brine, dried over $MgSO_4$, filtered, and concentrated in vacuo to yield methyl (E)-4-methylpent-2-enoate, which was used in the next step without further purification. In a three-neck, 250 mL round bottom flask equipped with an addition funnel and a magnetic stir bar, DIBAL-H (1 M in heptane, 140 mL, 140 mmol) was added dropwise to a solution of methyl (E)-4-methylpent-2-enoate (1.200 g, 1 Eq, 9.363 mmol) in DCM (39.0 mL) at −78 °C. The mixture was stirred at −78 °C for 1 h and quenched with 1 M HCl at 0 °C. The layers were separated and the aqueous layer was extracted with DCM (3 x 20 mL). The combined organic layers were washed with brine, dried over $MgSO_4$, filtered, then concentrated in vacuo to yield (*E*)-4-methylpent-2-en-1-ol which was used in the next step without further purification.

In an oven-dried round-bottom flask equipped with a magnetic stir bar, (*E*)-4-methylpent-2-en-1-ol (900 mg, 1.00 Eq, 8.99 mmol) was dissolved in anhydrous MeCN (9.00 mL). Triethylamine (2.73 g, 3.76 mL, 3.00 Eq, 27.0 mmol) and DMAP (110 mg, 0.10 Eq, 0.899 mmol) were added to the reaction mixture. Finally, benzoyl chloride (1.89 g, 1.56 mL, 1.50 Eq, 13.5 mmol) was added to the reaction dropwise at room temperature and the mixture was allowed to stir for 16 h. After reaction completion, the reaction was quenched with saturated aqueous $NaHCO_3$, then extracted with ethyl acetate. The organic layer was washed with brine, then dried over Na_2SO_4, filtered, and concentrated in vacuo. The product was isolated by flash chromatography (ethyl acetate/hexanes) to yield (E)-4-methylpent-2-en-1-yl benzoate (682 mg, 67% yield). ^{1}H-NMR (500 MHz, $CDCl_3$) δ 8.06 (d, *J* = 8.3 Hz, 2H), 7.55 (t, *J* = 6.7 Hz, 1H), 7.44 (t, *J* = 7.7 Hz, 2H), 5.83 (ddd, *J* = 15.5, 6.5, 1.4 Hz, 1H), 5.73 – 5.56 (m, 1H), 4.77 (d, *J* = 6.3 Hz, 2H), 2.35 (h, *J* = 6.8 Hz, 1H), 1.03 (dd,

J = 6.8, 1.5 Hz, 6H). ^{13}C NMR (126 MHz, $CDCl_3$) δ 166.95, 143.72, 133.33, 130.97, 130.12, 128.81, 121.50, 66.36, 31.31, 22.54. **HRMS** (ESI+) calc: $[M+Na]^+$ 227.1042, measured: 227.1041; difference = 0.4 ppm.

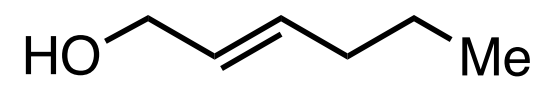

(*E*)-hex-2-en-1-ol: In a 250 mL round-bottom flask equipped with a magnetic stir bar, (E)-hex-2-en-1-yl acetate (13.5 g, 15.00 mL, 1 Eq, 94.7 mmol) was dissolved in MeOH (150 mL). Aqueous sodium hydroxide (0.60 g, 15 mL, 1 molar, 0.16 Eq, 15 mmol) was added and the reaction mixture was stirred overnight until complete by TLC monitoring. Then, MeOH was removed in vacuo and the aqueous layer was extracted with DCM (x3). The combined organic layers were dried over Na_2SO_4, filtered, and the solvent was removed in vacuo to yield the title compound (8.58 g, 85.7 mmol, 90.4 %). ^{1}H-NMR (500 MHz, $CDCl_3$) δ 5.73 – 5.58 (m, 2H), 4.08 (dd, *J* = 5.4, 1.1 Hz, 2H), 2.02 (q, *J* = 6.9 Hz, 2H), 1.57 (s, 1H), 1.40 (h, *J* = 7.4 Hz, 2H), 0.90 (t, *J* = 7.4 Hz, 3H), consistent with reported spectra (*Tetrahedron* **2015**, *71*, 7, 1132-1135).

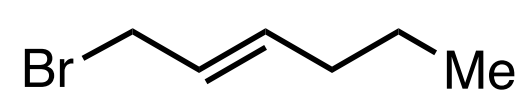

(*E*)-1-bromohex-2-ene: Adapted from a procedure reported by Tanino and coworkers (*Org. Lett.* **2021**, *23*, 24, 9606–9610). In a 250-mL round bottom flask equipped with a magnetic stir bar, (E)-hex-2-en-1-ol (8.500 g, 1 Eq, 84.86 mmol) was dissolved in anhydrous Et_2O (138 mL) and tribromophosphane (11.49 g, 4.002 mL, 0.5 Eq, 42.43 mmol) was added dropwise at 0°C. The resultant mixture was stirred at 0°C for 30 mins, until complete by TLC. Upon completion, the reaction was quenched by saturated $NaHCO_3$. The layers were separated and the aqueous layer was washed with Et_2O (2 x 30 mL). The combined organic extracts were dried over $MgSO_4$, filtered, and concentrated under reduced pressure to yield the title compound (6.8 g, 42 mmol, 49%). The product was used without further purification. ^{1}H-NMR (500 MHz, $CDCl_3$) δ 5.78 (dt, *J* = 15.1, 6.5 Hz, 1H), 5.72 – 5.64 (m, 1H), 3.95 (d, *J* = 7.3 Hz, 2H), 2.04 (q, *J* = 7.1 Hz, 2H), 1.41 (h, *J* = 7.4 Hz, 2H), 0.91 (t, *J* = 7.4 Hz, 3H), consistent with reported spectra (*Helv. Chim. Acta* **1991**, *74*, 941–950).

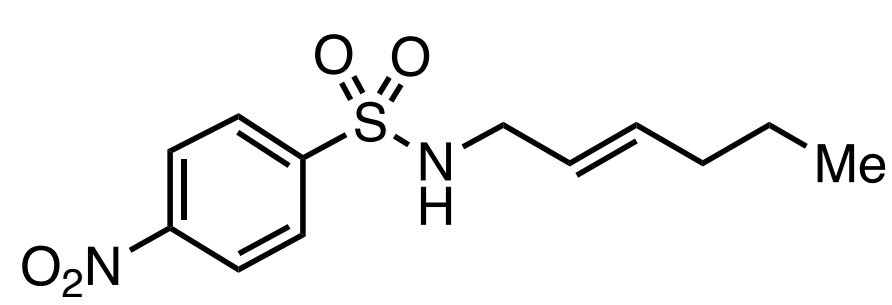

(*E*)-*N*-(hex-2-en-1-yl)-4-nitrobenzenesulfonamide: To a flame-dried two-neck 100 mL round-bottom flask equipped with a magnetic stir bar and a findenser, 4-nitrobenzenesulfonamide (2.832 g, 2.0 Eq, 14.01 mmol) and potassium carbonate (1.452 g, 1.5 Eq, 10.51 mmol) were added. The solids were dissolved in anhydrous MeCN (28.01 mL) and (E)-1-bromohex-2-ene (1.142 g, 1 Eq, 7.004 mmol) was added at room temperature. The reaction mixture was stirred at 60 °C for 16

h. The reaction was cooled to room temperature and quenched with saturated aqueous NH_4Cl (20 mL). The mixture was extracted with ethyl acetate (3 × 15 mL). The organic layers were combined, washed with brine (10 mL), then dried over $MgSO_4$ and filtered. The solvent was removed in vacuo and the crude product was purified via flash chromatography (ethyl acetate/hexanes) to the title compound (1.116 g, 3.925 mmol, 56.04%). ^{1}H-NMR (500 MHz, $CDCl_3$) δ 8.63 – 8.22 (m, 2H), 8.16 – 7.91 (m, 2H), 5.59 (dtd, J = 15.2, 6.8, 1.5 Hz, 1H), 5.30 (dtt, J = 15.3, 6.4, 1.4 Hz, 1H), 4.56 (t, J = 6.2 Hz, 1H), 3.85 – 3.39 (m, 2H), 1.92 (q, J = 7.2 Hz, 2H), 1.31 (h, J = 7.5 Hz, 2H), 0.84 (t, J = 7.3 Hz, 3H). ^{13}C NMR (126 MHz, $CDCl_3$) δ 150.47, 146.68, 136.23, 128.76, 124.76, 124.30, 45.92, 34.49, 22.40, 13.95. **HRMS** (ESI+) calc: $[M+NH_4^+]^+$ 302.1169, measured: 302.1166; difference = 1.0 ppm.

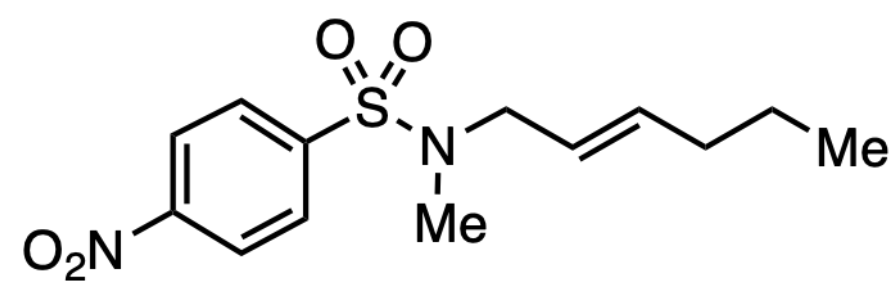

(*E*)-*N*-(hex-2-en-1-yl)-*N*-methyl-4-nitrobenzenesulfonamide. In a flame-dried round-bottom flask equipped with a magnetic stir bar, (E)-N-(hex-2-en-1-yl)-4-nitrobenzenesulfonamide (549 mg, 1 Eq, 1.93 mmol) was dissolved in DMF (6.44 mL). Potassium carbonate (534 mg, 2.00 Eq, 3.86 mmol) was added, followed by iodomethane (301 mg, 132 µL, 1.1 Eq, 2.12 mmol). The flask was equipped with a findenser and the solution was heated to 50° C and stirred at that temperature overnight. The solution was cooled to rt and then partitioned between 50 mL water and 50 mL ethyl acetate. The layers were separated, and the aqueous phase was extracted with ethyl acetate (50 mL). The combined organic phases were washed with 5% LiCl (3 x 20 mL) solution, dried over $MgSO_4$, filtered, and concentrated. The product was purified by flash chromatography (ethyl acetate/hexanes) to yield the title compound (537 mg, 1.80 mmol, 93.2 %). ^{1}H-NMR (500 MHz, $CDCl_3$) δ 8.39 – 8.33 (m, 2H), 8.01 – 7.94 (m, 2H), 5.61 (dtt, J = 15.0, 6.7, 1.3 Hz, 1H), 5.30 (dtt, J = 15.2, 6.7, 1.5 Hz, 1H), 3.66 (dd, J = 6.7, 1.3 Hz, 2H), 2.73 (s, 3H), 1.98 (q, J = 6.9 Hz, 2H), 1.35 (h, J = 7.4 Hz, 2H), 0.86 (t, J = 7.4 Hz, 3H). ^{13}C NMR (126 MHz, $CDCl_3$) δ 150.16, 144.18, 137.08, 128.69, 124.45, 123.32, 52.54, 34.29, 34.04, 22.22, 13.70. **HRMS** (ESI+) calc: $[M+NH_4^+]^+$ 316.1326, measured: 316.1325; difference = 0.3 ppm.

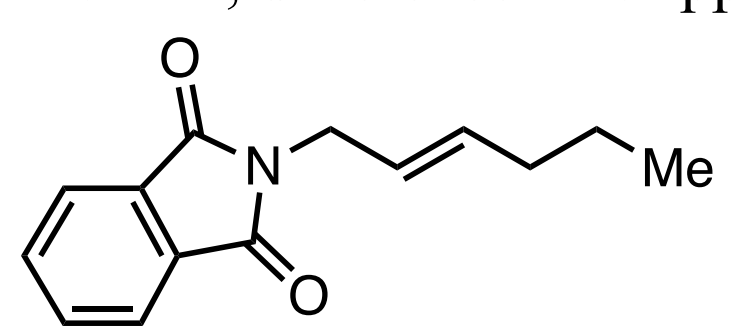

(*E*)-2-(hex-2-en-1-yl)isoindoline-1,3-dione: Synthesized according to a protocol reported by Zhang and coworkers (*J. Am. Chem. Soc.* **2016**, *138*, 29, 9017–9020). In a round-bottom flask equipped with a magnetic stir bar, (E)-hex-2-en-1-ol (4.21 g, 1 Eq, 42.0 mmol), isoindoline-1,3-dione (6.80 g, 1.10 Eq, 46.2 mmol), and triphenylphosphine (12.1 g, 1.10 Eq, 46.2 mmol) were added and dissolved in anhydrous THF (210 mL) under nitrogen atmosphere. The resulting solution was cooled to 0 °C and diisopropyl azodicarboxylate (9.34 g, 8.98 mL, 1.10 Eq, 46.2 mmol) was added dropwise. The resulting solution was allowed to warm to room temperature and stirred at room temperature for 4 h. THF was removed in vacuo and the residue was dissolved in

DCM (250 mL) and DI water (250 mL) was added. The phases were separated and the aqueous layer was extracted with DCM (250 ml X 3). The combined organic layers were combined and dried over Na_2SO_4, filtered, and concentrated. The residue was purified by flash chromatography (ethyl acetate/hexanes) to give the title compound (7.07 g, 73% yield). ^{1}H-NMR (400 MHz, $CDCl_3$) δ 7.85 (dd, *J* = 5.4, 3.1 Hz, 2H), 7.72 (dd, *J* = 5.4, 3.0 Hz, 2H), 5.75 (dtt, *J* = 14.9, 6.7, 1.3 Hz, 1H), 5.52 (dtt, *J* = 15.4, 6.3, 1.5 Hz, 1H), 4.24 (dq, *J* = 6.3, 1.1 Hz, 2H), 2.08 – 1.94 (m, 2H), 1.38 (h, *J* = 7.4 Hz, 2H), 0.87 (t, *J* = 7.3 Hz, 3H), consistent with reported spectra.

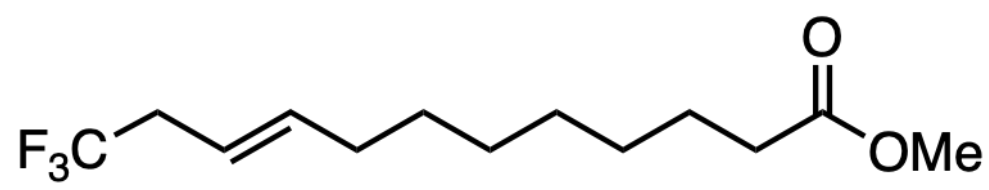

methyl (*E*)-12,12,12-trifluorododec-9-enoate: Synthesized according to a protocol developed by Omote and coworkers (*J. Org. Chem.* **2020**, *85*, 7, 5104–5108). In a 100 mL round-bottom flask equipped with a magnetic stir bar, silver(I) fluoride (1.01 g, 4.00 Eq, 8.00 mmol), copper (II) acetate (72.7 mg, 0.20 Eq, 0.400 mmol), and 2,2'-bipyridine (68.7 mg, 0.22 Eq, 0.440 mmol) were placed in a flask and suspended with anhydrous DMSO (15.4 mL). To the suspension, methyl undec-10-enoate (397 mg, 450 µL, 1 Eq, 2.00 mmol) and trimethyl(trifluoromethyl)silane (1.14 g, 1.18 mL, 4.00 Eq, 8.00 mmol) was added. After stirring for 5 min, the flask was equipped with a findenser and the mixture was heated at 70 °C for 3.5 h. The reaction was cooled to rt and quenched with aqueous 10% HCl. The mixture was extracted with Et_2O, the Et_2O layer was dried over $MgSO_4$ and filtered. The solvent was removed *in vacuo*, and the residue was purified by flash chromatography (DCM/hexanes) to give the title compound (393 mg, 1.48 mmol, 73.8 %, >98:2 d.r.). ^{1}H-NMR (500 MHz, $CDCl_3$) δ 5.68 (dt, *J* = 14.4, 6.9 Hz, 1H), 5.36 (dtt, *J* = 15.5, 7.1, 1.5 Hz, 1H), 3.66 (s, 3H), 2.81 – 2.70 (m, 2H), 2.30 (t, *J* = 7.5 Hz, 2H), 2.04 (q, *J* = 7.1 Hz, 2H), 1.62 (h, *J* = 6.6 Hz, 2H), 1.37 (t, *J* = 7.0 Hz, 2H), 1.33 – 1.25 (m, 6H), consistent with reported spectra.

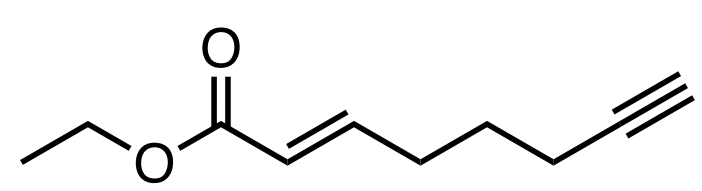

ethyl (*E*)-oct-2-en-7-ynoate: Adapted from a protocol reported by Breinbauer and coworkers (*J. Am. Chem. Soc.* **2019**, *141*, 37, 14931–14937). In a flame-dried and nitrogen-flushed 250 mL two-necked round-bottom flask equipped with a magnetic stir bar, DMSO (2.930 g, 2.661 mL, 2.50 Eq, 37.50 mmol) was added dropwise to a solution of oxalyl dichloride (2.856 g, 1.93 mL, 1.50 Eq, 22.50 mmol) in anhydrous DCM (75 mL) at -78 °C and stirred for 15 min. Then a solution of hex-5-yn-1-ol (1.472 g, 1 Eq, 15.00 mmol) in anhydrous DCM (19 mL) (prepared in a flame-dried flask) was slowly added. The resulting suspension was stirred for 15 min before triethylamine (7.589 g, 5.00 Eq, 75.00 mmol) was added. The reaction mixture was stirred for 30 min at -78 °C after which it was allowed to warm to rt (90 min, reaction monitoring via TLC). Then (carbethoxymethylene)triphenylphosphorane (6.271 g, 1.20 Eq, 18.00 mmol) was added in one portion and the suspension was stirred at rt for 16 h, then concentrated under reduced

pressure. The resulting residue was suspended in cold DCM and solids were filtered off. The filtrate was concentrated under reduced pressure and the product was isolated by flash chromatography (ethyl acetate/hexanes) to yield the title compound (1.341 g, 8.068 mmol, 53.78 %). ^{1}H-NMR (500 MHz, $CDCl_3$) δ 6.96 (dt, *J* = 15.6, 7.0 Hz, 1H), 5.88 (dt, *J* = 15.7, 1.5 Hz, 1H), 4.21 (q, *J* = 7.1 Hz, 2H), 2.36 (qd, *J* = 7.1, 1.6 Hz, 2H), 2.25 (td, *J* = 7.0, 2.7 Hz, 2H), 1.99 (t, *J* = 2.6 Hz, 1H), 1.71 (q, *J* = 7.3 Hz, 2H), 1.31 (t, *J* = 7.1 Hz, 3H), consistent with reported spectra.

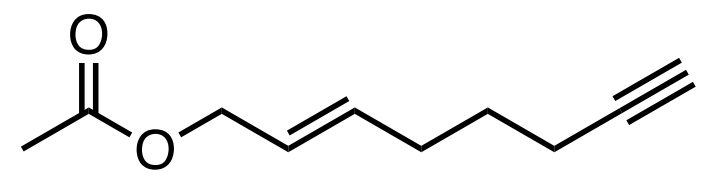

(E)-oct-2-en-7-yn-1-yl acetate: In a flame-dried, nitrogen-flushed 100 mL round-bottom flask equipped with an addition funnel and a magnetic stir bar, ethyl (E)-oct-2-en-7-ynoate (1.26 g, 7.6 mmol) was dissolved in anhydrous DCM (20 mL) and cooled to -78 °C (dry ice/acetone). Subsequently, DIBAL-H (16 mL, 1.0 M soln. in heptane, 16 mmol) was added over 15 min by addition funnel and the mixture was stirred at -78 °C for 30 min. Upon complete consumption of the starting material (according to TLC), the reaction mixture was quenched by the addition of 1 M HCl (20 mL) and slowly warmed to rt. The organic layer was separated and the aqueous layer was extracted with DCM (10 mL x 3). The combined organic layers were washed with H_2O (10 mL), dried over Na_2SO_4, filtered and concentrated under reduced pressure to give (E)-oct-2-en-7-yn-1-ol (941.3 mg, 7.580 mmol, 100.0 %), which was used without further purification. In a flame-dried, 25 mL round-bottom flask equipped with a magnetic stir bar, (E)-oct-2-en-7-yn-1-ol (1.010 g, 1 Eq, 8.133 mmol), was dissolved in anhydrous DCM (6.256 mL). At room temperature, triethylamine (2.090 g, 2.88 mL, 2.54 Eq, 20.66 mmol) was added, followed by DMAP (49.68 mg, 0.0500 Eq, 0.407 mmol). The flask was cooled in an ice bath, and acetic anhydride (2.109 g, 1.949 mL, 2.54 Eq, 20.66 mmol) was added dropwise via syringe. The resulting solution was stirred under nitrogen at room temperature overnight. Upon completion, the reaction mixture was quenched with saturated $NaHCO_3$ (2 × 15 mL). The aqueous layer was combined and washed with DCM (15 mL, x3). The combined organic layers were then washed with brine (60 mL) and dried over Na_2SO_4. Solvent was removed in vacuo, and product was isolated by flash chromatography (ethyl acetate/hexanes) to yield the title compound (1.109 g, 6.672 mmol, 82.03 %). ^{1}H-NMR (500 MHz, $CDCl_3$) δ 5.68 (dtt, *J* = 14.7, 6.7, 1.3 Hz, 1H), 5.54 (dtt, *J* = 15.5, 6.4, 1.5 Hz, 1H), 4.44 (dd, *J* = 6.4, 1.2 Hz, 2H), 2.13 (m, 4H), 1.99 (s, 3H), 1.88 (t, *J* = 2.6 Hz, 1H), 1.56 (p, *J* = 7.2 Hz, 2H). ^{13}C NMR (126 MHz, $CDCl_3$) δ 170.81, 135.01, 124.82, 84.04, 68.58, 65.07, 31.08, 27.59, 21.00, 17.80. **HRMS** (ESI+) calc: $[M+H]^+$ 167.1067, measured: 167.1066; difference = 0.6 ppm.

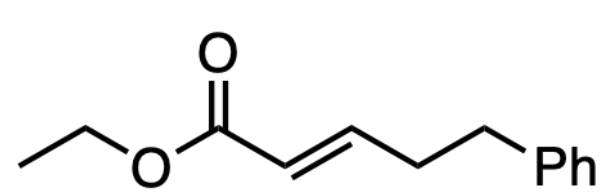

ethyl (*E*)-5-phenylpent-2-enoate: An oven dried 100mL round-bottom flask equipped with a magnetic stir bar was charged with LiCl (590 mg, 1.4 Eq, 14 mmol). The flask was capped, placed

under nitrogen atmosphere, and charged with anhydrous MeCN (16 mL) and DBU (1.8 g, 1.8 mL, 1.2 Eq, 12 mmol). The contents were cooled to 0° C, charged with ethyl 2-(diethoxyphosphoryl)acetate (2.9 g, 2.6 mL, 1.3 Eq, 13 mmol), then stirred for 10 min at 0° C. A separate oven dried 25 mL round-bottom flask equipped was charged with MeCN (4 mL) and distilled 3-phenylpropanal (1.34 g, 1.33 mL, 1.0 Eq, 10 mmol). The aldehyde solution was added all at once to the flask containing the phosphorus reagent at 0° C. The contents were stirred for 30 min at 0° C, then warmed to rt and continued stirring for an additional 30 min. The reaction was quenched with sat. aqueous $NaHCO_3$ (50 mL) and EtOAc was added (20 mL). The layers were separated and the aqueous layer was extracted with EtOAc (2 x 20 mL). The organic layers were combined and then washed with DI H_2O (1 x 20 mL), then dried over Na_2SO_4. The mixture was isolated by flash chromatography (ethyl acetate/hexanes) to yield the title compound (1.8 g, 8.7 mmol, 87%). ^{1}H-NMR (500 MHz, $CDCl_3$) δ 7.29 (t, *J* = 7.6 Hz, 2H), 7.24 – 7.16 (m, 3H), 7.00 (dt, *J* = 15.7, 6.8 Hz, 1H), 5.85 (dt, *J* = 15.7, 1.6 Hz, 1H), 4.19 (q, *J* = 7.2 Hz, 2H), 2.78 (t, *J* = 7.8 Hz, 2H), 2.53 (dtd, *J* = 8.9, 7.0, 1.6 Hz, 2H), 1.29 (t, *J* = 7.1 Hz, 3H), consistent with reported spectra (*Tetrahedron* **2013**, *54*, 21, 2658-2660).

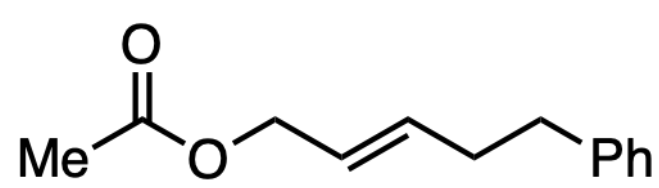

(*E*)-5-phenylpent-2-en-1-yl acetate: Adapted from a procedure reported by Zakarian and coworkers (*Org. Lett.* **2018**, *20*, 16, 4867–4870). A flame-dried 250mL three necked round bottom flask equipped with a magnetic stir bar and addition funnel was placed under nitrogen atmosphere and charged with ethyl (*E*)-5-phenylpent-2-enoate (1.787 g, 8.748 mmol, 1.0 eq.). DIBAL (1.0 molar in hexanes, 26.24 mL, 26.24 mmol, 3.0 eq.) was added dropwise over 1 h at -78°C, then stirred for an additional h at -78°C. The reaction mixture was then quenched by the dropwise addition of MeOH (2 mL) and stirred for 5 min. A saturated aqueous solution of Rochelle's salt (110 mL) was added, and the mixture was allowed to warm to room temperature while stirring vigorously for 2.5 h. The layers were then separated, and the aqueous layer was extracted with DCM (2 x 50 mL). The combined organic layers were washed with brine, dried over Na_2SO_4, filtered, and concentrated under reduced pressure. The crude (*E*)-5-phenylpent-2-en-1-ol was carried into the acetylation step without further purification. An oven-dried 100mL round-bottom flask equipped with a magnetic stir bar was charged sequentially with anhydrous pyridine (15.0 mL), (*E*)-5-phenylpent-2-en-1-ol (1.223 g, 7.539 mmol, 1.0 eq.), acetic anhydride (1.07 mL, 11.31 mmol, 1.5 eq.), and DMAP (5 mg, 0.037 mmol, 0.005 eq.) at room temperature. The mixture was stirred for 6 h, and saturated NH_4Cl (5 mL) was added. The resulting mixture was extracted with Et_2O (3 x 10 mL), and the combined organic layers were washed with saturated $NaHCO_3$ (10 mL), dried over Na_2SO_4, filtered, and concentrated under reduced pressure. The crude oil was purified by flash chromatography (ethyl acetate/hexanes) to give the title compound (1.18 g, 5.77 mmol, 77%, >98:2 d.r.).^{1}H-NMR (500 MHz, $CDCl_3$) δ 7.32 – 7.26 (m, 2H), 7.22 – 7.14 (m, 3H), 5.82 (dtt, *J* = 15.6, 6.7, 1.2 Hz, 1H), 5.60 (dtt, *J* = 15.4, 6.4, 1.5 Hz, 1H), 4.51 (d, *J* = 6.5 Hz, 2H), 2.71 (dd, *J* = 9.0, 6.7 Hz, 2H), 2.39 (q, *J* = 7.3 Hz, 2H), 2.06 (s, 3H), consistent with reported spectra (*Chem. Commun.* **2001**, *37*, 4, 357–358).

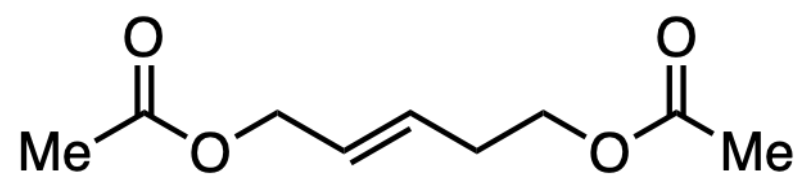

(*E*)-pent-2-ene-1,5-diyl diacetate: Synthesized according to a protocol developed by Stahl and coworkers (*J. Am. Chem. Soc.* **2010**, *132*, 15116–15119). To an oven-dried 50-mL round-bottom flask equipped with a magnetic stir bar were added $Pd(OAc)_2$ (56 mg, 0.250 mmol, 5.0 mol%), NaOAc (82 mg, 1.0 mmol, 0.20 eq.), and 4,5-diazafluorenone (46 mg, 0.250 mmol, 5.0 mol%). The flask was sealed, then charged sequentially with 1,4-dioxane (14.0 mL), pent-4-en-1-yl acetate (700 µL, 5.00 mmol, 1.0 eq.), and acetic acid (4.58 mL, 80.0 mmol, 16.0 eq.) via syringe. O_2 was bubbled through the solution for ca. 15 min with vigorous stirring. The reaction was then stirred vigorously at 60°C for 96 h under a balloon of O_2. The reaction was cooled to rt, and water (20 mL) was added. The mixture was extracted with ethyl acetate (3 x 20 mL). The combined organic layers were dried over Na_2SO_4, filtered, and concentrated under reduced pressure. The crude mixture was purified by flash chromatography (ethyl acetate/hexanes) to provide the title compound (615 mg, 66% yield, 90:10 d.r.). ^{1}H-NMR (500 MHz, $CDCl_3$) δ 5.74 (dt, J = 15.4, 6.3 Hz, 1H), 5.70 – 5.61 (m, 1H), 4.52 (d, J = 6.0 Hz, 2H), 4.11 (t, J = 6.8 Hz, 2H), 2.39 (q, J = 6.8 Hz, 2H), 2.06 (s, 3H), 2.04 (s, 3H)., consistent with reported spectra (*Org. Lett*. **2010**, *12*, 4, 824–827).

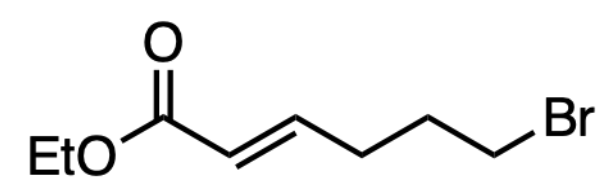

ethyl (*E*)-6-bromohex-2-enoate. Adapted from a protocol reported by Breinbauer and coworkers (*J. Am. Chem. Soc.* **2019**, *141*, 37, 14931–14937). In a flame-dried and nitrogen-flushed 1 L two-necked round-bottom flask, equipped with a magnetic stir bar, ethyl 4-bromobutanoate (3.901 g, 1 Eq, 20.00 mmol) was dissolved in anhydrous DCM (300 mL) and cooled to -78 °C. Subsequently, DIBAL-H (3.413 g, 24.00 mL, 1 molar, 1.200 Eq, 24.00 mmol) was added over 10 min and the mixture was stirred at -78 °C for 1 h. Upon complete consumption of the starting material (according to TLC), the reaction mixture was quenched by the addition of 1 M HCl (200 mL) and slowly warmed to rt. The organic layer was separated and the aqueous layer was extracted with DCM (3 x 50 mL). The combined organic layers were dried over Na_2SO_4, filtered and carefully concentrated under reduced pressure to a final volume of approx. 30 mL. The crude intermediate was quantitatively transferred into a 250 mL round-bottom flask, equipped with a magnetic stirring bar, and then a solution of (carbethoxymethylene)triphenylphosphorane (8.361 g, 1.200 Eq, 24.00 mmol) in DCM (30 mL) was added over 10 min at rt. The reaction mixture was stirred overnight (reaction monitoring via TLC) and concentrated under reduced pressure. The crude product was adsorbed onto silica and purified via flash chromatography (ethyl acetate/hexanes) to give the title compound (2.6 g, 58%). ^{1}H-NMR (400 MHz, $CDCl_3$) δ 6.91 (dt, J = 15.5, 7.0 Hz, 1H), 5.88 (dt, J = 15.6, 1.6 Hz, 1H), 4.19 (q, J = 7.1 Hz, 2H), 3.41 (t, J = 6.5 Hz, 2H), 2.38 (qd, J = 7.2, 1.6 Hz, 2H), 2.08 – 1.96 (m, 2H), 1.29 (t, J = 7.1 Hz, 3H), consistent with reported spectra.

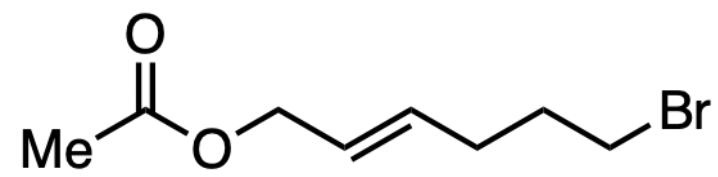

(*E*)-6-bromohex-2-en-1-yl acetate. In a flame-dried and nitrogen-flushed 250 mL round-bottom flask, equipped with a magnetic stirring bar, ethyl (E)-6-bromohex-2-enoate (2.40 g, 1 Eq, 10.9 mmol) was dissolved in anhydrous DCM (31.6 mL) and cooled to -78 °C. Subsequently, DIBAL-H (3.24 g, 22.8 mL, 1.0 molar, 2.10 Eq, 22.8 mmol) was added over 15 min and the mixture was stirred at -78 °C for 30 min. Upon complete consumption of the starting material (according to TLC), the reaction mixture was quenched by the addition of 1 M HCl (30 mL) and slowly warmed to rt. The organic layer was separated and the aqueous layer was extracted with DCM (2 x 20 mL). The combined organic layers were dried over Na_2SO_4, filtered and concentrated under reduced pressure. The resulting alcohol was used in the following step without purification. In a flame-dried, 250 mL round-bottom flask, equipped with a magnetic stir bar, were placed crude (E)-6-bromohex-2-en-1-ol (1.700 g, 1 Eq, 9.494 mmol), triethylamine (2.440 g, 3.36 mL, 2.54 Eq, 24.11 mmol) and dry DCM (7.303 mL) via syringe. The resulting solution was stirred under nitrogen at room temperature for 30 min, after which DMAP (57.99 mg, 0.0500 Eq, 0.474 mmol) was added. The flask was cooled in an ice bath, and acetic anhydride (2.462 g, 2.275 mL, 2.54 Eq, 24.11 mmol) was added dropwise via syringe. The resulting solution was stirred under nitrogen at room temperature overnight, until TLC showed no remaining starting material. The reaction was quenched by addition of saturated $NaHCO_3$ (15 mL), the layers were separated, and the aqueous layer was washed with DCM (2 x 15 mL). The combined organic fractions were then washed with brine (15 mL) and dried over Na_2SO_4, then filtered. Solvent was removed in vacuo, and crude mixture was isolated by flash chromatography (ethyl acetate/hexanes). Purified yield: 1.34 g, 64%. ^{1}H-NMR (500 MHz, $CDCl_3$) δ 5.78 – 5.67 (m, 1H), 5.67 – 5.57 (m, 1H), 4.50 (d, *J* = 6.3 Hz, 2H), 3.39 (t, *J* = 6.6 Hz, 2H), 2.21 (q, *J* = 7.1 Hz, 2H), 2.05 (s, 3H), 1.94 (p, *J* = 6.8 Hz, 2H). ^{13}C-NMR (126 MHz, $CDCl_3$) δ 170.87, 133.99, 125.54, 65.01, 33.03, 31.81, 30.64, 21.10. **HRMS** (ESI+) calc: $[M+Na]^+$ 242.9991, measured: 242.9987; difference = 1.6 ppm.

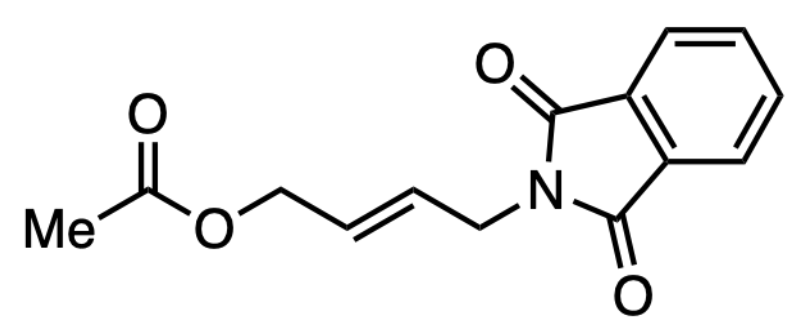

(*E*)-4-(1,3-dioxoisoindolin-2-yl)but-2-en-1-yl acetate: Synthesized according to a protocol developed by Stahl and coworkers (*J. Am. Chem. Soc.* **2010**, *132*, 15116–15119). To an oven-dried 50-mL round-bottom flask equipped with a magnetic stir bar were added $Pd(OAc)_2$ (56 mg, 0.250 mmol, 5.0 mol%), NaOAc (82 mg, 1.0 mmol, 0.20 eq.), and 4,5-diazafluorenone (46 mg, 0.250 mmol, 5.0 mol%). The flask was sealed, then charged sequentially with 1,4-dioxane (14.0 mL), 2-(but-3-en-1-yl)isoindoline-1,3-dione (1.01 g, 5.00 mmol, 1.0 eq.), and acetic acid (4.58 mL, 80.0 mmol, 16.0 eq.) via syringe. O_2 was bubbled through the solution for ca. 30 min with vigorous stirring. The reaction was then stirred vigorously at 60°C for 60 h under a balloon of O_2. The reaction was cooled to rt, and water (20 mL) was added. The mixture was extracted with ethyl

acetate (3 x 20 mL). The combined organic layers were dried over Na_2SO_4, filtered, and concentrated under reduced pressure. The crude mixture was purified by flash chromatography (ethyl acetate/hexanes) to provide the title compound (407 mg, 31% yield, 95:5 d.r.). ^{1}H-NMR (500 MHz, $CDCl_3$) δ 7.85 (dd, *J* = 5.5, 3.1 Hz, 2H), 7.72 (dd, *J* = 5.5, 3.0 Hz, 2H), 5.82 (td, *J* = 4.6, 3.6 Hz, 2H), 4.53 (dt, *J* = 4.4, 1.2 Hz, 2H), 4.30 (dt, *J* = 4.7, 1.2 Hz, 2H), 2.04 (s, 3H). ^{13}C NMR (126 MHz, $CDCl_3$) δ 170.74, 167.95, 134.17, 132.23, 127.94, 127.67, 123.49, 63.92, 38.96, 21.01. **HRMS** (ESI+) calc: $[M+NH4]^+$ 277.1183, measured: 277.1179; difference = 1.4 ppm.

4. Cross-Coupling Procedures

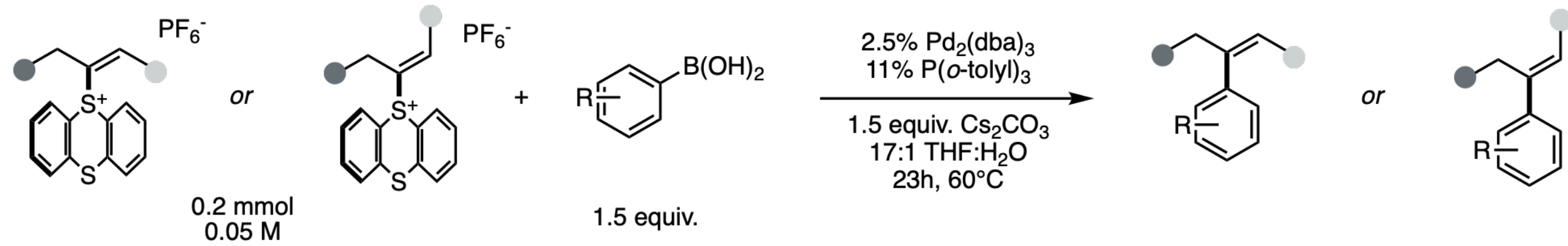

General Procedure D: C(sp²)-C(sp²) Suzuki Coupling. In an N_2-filled glovebox, to a 2-dram borosilicate vial equipped with a magnetic stir bar were added $Pd_2(dba)_3$ (4.6 mg, 0.005 mmol, 0.025 equiv.), $P(o\text{-tolyl})_3$ (6.70 mg, 0.022 mmol, 0.11 equiv.), Cs_2CO_3 (97.7 mg, 0.300 mmol, 1.5 equiv.), boronic acid coupling partner (0.300 mmol, 1.5 equiv.), and *Z*- or *E*- alkenyl thianthrenium salt (0.200 mmol). The vial was charged with anhydrous THF (4.0 mL), fitted with a septum screw cap, removed from the glovebox, and charged with degassed, deionized water (360 µL) via syringe. The resulting mixture was stirred vigorously in a heating block at 60° C for 23 h. At completion of reaction, the contents of the vial were cooled to room temperature and filtered through a short pad of Celite, eluting with ethyl acetate. The filtrate was concentrated under reduced pressure and the residue purified by flash chromatography to afford the pure Suzuki product.

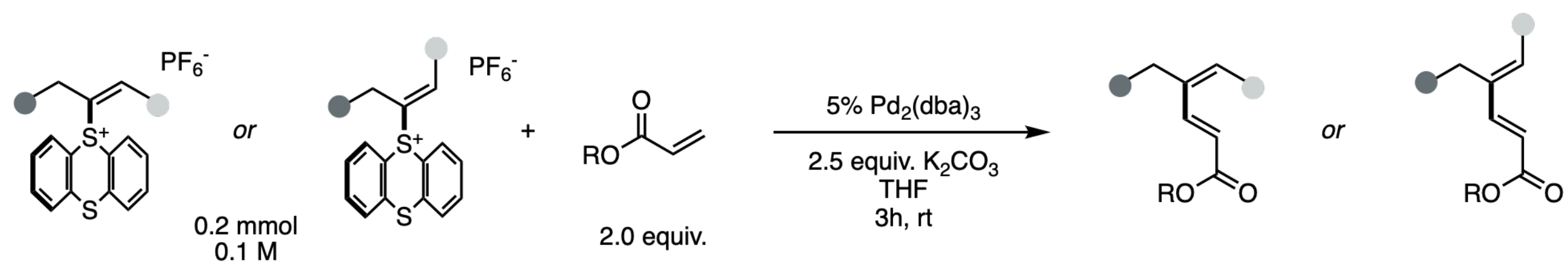

General Procedure E: C(sp²)-C(sp²) Mizoroki-Heck Coupling. In an N_2-filled glovebox, to a 2-dram borosilicate vial equipped with a magnetic stir bar were added $Pd_2(dba)_3$ (9.16 mg, 0.010 mmol, 0.05 equiv.), potassium carbonate (69.1 mg, 0.500 mmol, 2.5 equiv.), and *Z*- or *E*- alkenyl thianthrenium salt (0.20 mmol). The vial was charged with anhydrous THF (2.0 mL) and alkene coupling partner (0.4 mmol, 2.0 equiv.), fitted with a Teflon screw cap, and allowed to stir at room temperature for 3 h. At completion of reaction, the vial was removed from the glovebox, and the contents were filtered through a short pad of Celite, eluting with ethyl acetate. The filtrate was concentrated under reduced pressure and the residue purified by flash chromatography to afford the pure Heck product.

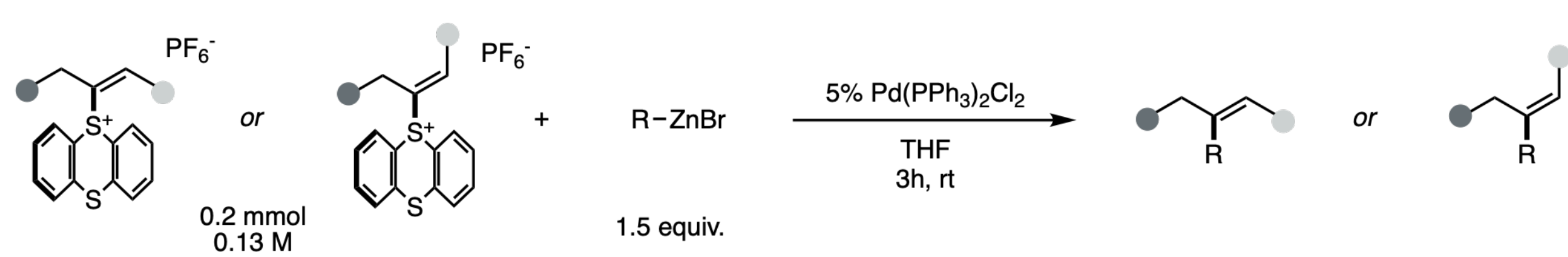

General Procedure F: C(sp²)-C(sp³) Negishi Coupling. In an N_2-filled glovebox, to a 1-dram borosilicate vial equipped with a magnetic stir bar were added $Pd(PPh_3)_2Cl_2$ (7.02 mg, 0.010 mmol, 0.05 equiv.) and *Z*- or *E*- alkenyl thianthrenium salt (0.2 mmol). The vial was charged with

anhydrous THF (1.400 mL) and alkylzinc bromide coupling partner (0.300 mmol, 1.5 equiv.), fitted with a Teflon screw cap, and allowed to stir at room temperature for 3 h. At completion of reaction, the vial was removed from the glovebox, and the contents were filtered through a short pad of Celite, eluting with ethyl acetate. The filtrate was concentrated under reduced pressure and the residue purified by flash chromatography to afford the pure Negishi product.

5. Product Isolation and Characterization

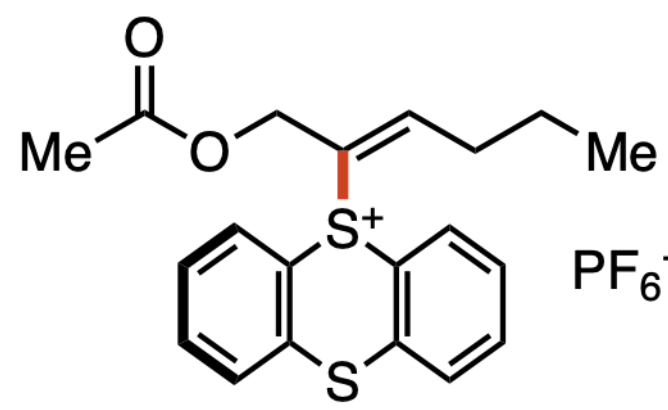

(*Z*)-5-(1-acetoxyhex-2-en-2-yl)-5*H*-thianthren-5-ium hexafluorophosphate (1): Prepared according to **General Procedure B** with the following modification: reaction run on a 0.8 mmol scale. Purified by flash chromatography using C2 silica and acetone/hexanes eluent, followed by recrystallization in DCM/ethyl acetate. Purified yield: 225 mg, 52%, >98:2 r.r.; >98:2 d.r. ^{1}**H-NMR** (500 MHz, CD_3CN) δ 8.11 (dd, *J* = 8.0, 1.3 Hz, 2H), 7.92 (dd, *J* = 7.9, 1.4 Hz, 2H), 7.83 (td, *J* = 7.7, 1.3 Hz, 2H), 7.72 (td, *J* = 7.7, 1.4 Hz, 2H), 6.88 (t, *J* = 7.7 Hz, 1H), 4.57 (s, 2H), 2.62 (q, *J* = 7.5 Hz, 2H), 1.74 (s, 3H), 1.49 (h, *J* = 7.4 Hz, 2H), 0.98 (t, *J* = 7.4 Hz, 3H). ^{13}**C-NMR** (126 MHz, DMSO) δ 169.27, 155.57, 135.25, 134.48, 134.08, 129.75, 129.72, 124.67, 117.37, 64.45, 31.55, 21.01, 19.99, 13.40. ^{19}**F-NMR** (377 MHz, CD_3CN) δ -72.90 (d, *J* = 706.4 Hz). ^{31}**P-NMR** (162 MHz, CD_3CN) δ -144.62 (hept, *J* = 706.3 Hz. **HRMS** (ESI+) calc: $[M]^+$ 357.0978, measured: 357.0975; difference = 0.8 ppm.

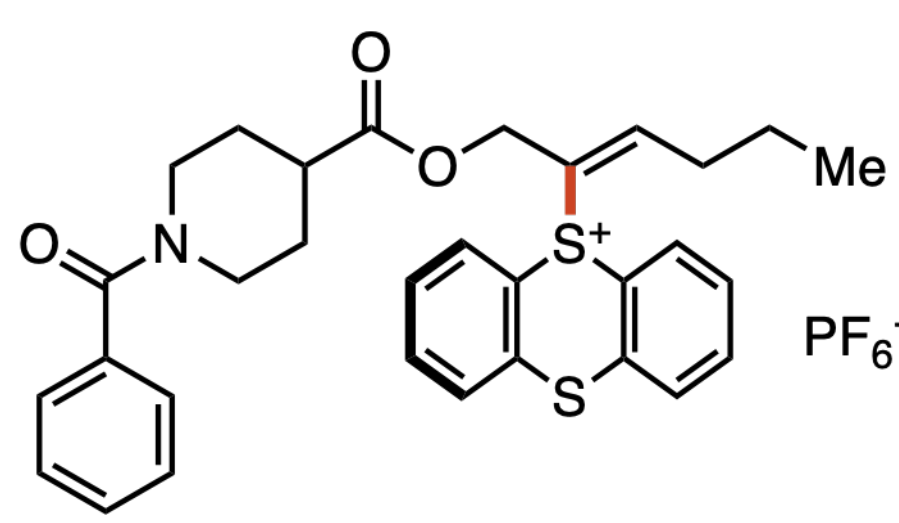

(*Z*)-5-(1-((1-benzoylpiperidine-4-carbonyl)oxy)hex-2-en-2-yl)-5*H*-thianthren-5-ium hexafluorophosphate (2): Prepared according to **General Procedure B**. Purified by flash chromatography using C2 silica and acetone/hexanes eluent. Purified yield: 180 mg, 67%, >98:2 r.r.; >98:2 d.r. ^{1}**H-NMR** (500 MHz, CD_3CN) δ 8.08 (dd, *J* = 8.0, 1.4 Hz, 2H), 7.89 (dd, *J* = 8.0, 1.3 Hz, 2H), 7.80 (td, *J* = 7.7, 1.4 Hz, 2H), 7.69 (td, *J* = 7.7, 1.4 Hz, 2H), 7.49 – 7.40 (m, 3H), 7.39 – 7.31 (m, 2H), 6.89 (t, *J* = 7.7 Hz, 1H), 4.62 (s, 2H), 4.35 (s, 1H), 3.55 (s, 1H), 2.90 (s, 2H), 2.65 (q, *J* = 7.4 Hz, 2H), 2.23 (tt, *J* = 11.1, 3.9 Hz, 1H), 1.64 (s, 2H), 1.50 (h, *J* = 7.4 Hz, 2H), 1.30 (d, *J* = 25.4 Hz, 2H), 0.98 (t, *J* = 7.3 Hz, 3H). ^{13}**C-NMR** (151 MHz, DMSO) δ 172.68, 169.02, 156.33, 136.07, 135.19, 134.49, 134.04, 129.86, 129.46, 128.45, 126.65, 124.92, 117.50, 64.52, 46.13,

45.75, 40.50, 31.67, 27.70, 27.15, 21.08, 13.43, 8.61. **^{19}F-NMR** (377 MHz, CD_3CN) δ -71.97, -73.84. **^{31}P-NMR** (162 MHz, CD_3CN) δ -131.54, -135.90, -140.26, -144.62, -148.98, -153.34, -157.70. **HRMS** (ESI+) calc: $[M]^+$ 530.1818, measured: 530.1821; difference = 0.6 ppm.

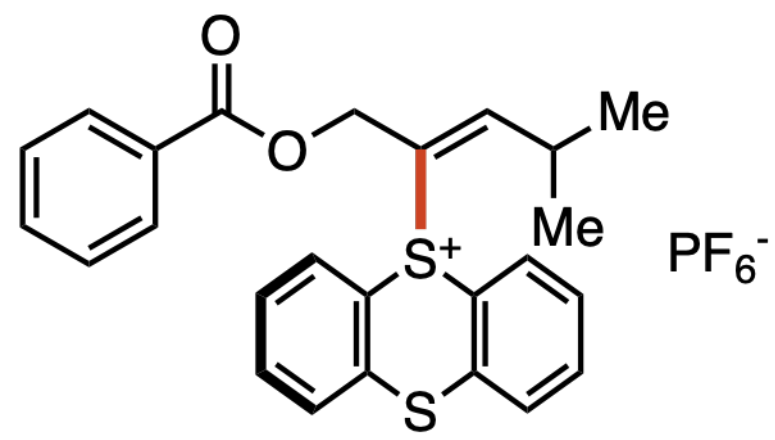

(*Z*)-5-(1-(benzoyloxy)-4-methylpent-2-en-2-yl)-5*H*-thianthren-5-ium hexafluorophosphate (3): Prepared according to **General Procedure B**. Purified by flash chromatography using C2 silica. Purified yield: 170 mg, 75%, >98:2 r.r.; >98:2 d.r. **^{1}H-NMR** (500 MHz, CD_3CN) δ 8.10 (dd, *J* = 8.1, 1.4 Hz, 2H), 7.81 (dd, *J* = 7.9, 1.4 Hz, 2H), 7.74 (td, *J* = 7.7, 1.2 Hz, 2H), 7.69 – 7.59 (m, 2H), 7.57 (d, *J* = 8.2 Hz, 1H), 7.43 (t, *J* = 7.7 Hz, 2H), 6.81 (d, *J* = 11.0 Hz, 1H), 4.83 (s, 2H), 3.47 – 3.36 (m, 1H), 1.10 (d, *J* = 6.3 Hz, 6H**). ^{13}C-NMR** (126 MHz, DMSO) δ 164.60, 161.92, 135.25, 134.46, 134.03, 133.82, 129.91, 129.88, 129.12, 128.63, 128.13, 122.89, 117.62, 65.32, 29.88, 21.28. **^{19}F-NMR** (471 MHz, CD_3CN) δ -72.91 (d, *J* = 706.4 Hz). **^{31}P-NMR** (202 MHz, CD_3CN) δ -144.62 (hept, *J* = 706.3 Hz). **HRMS** (ESI+) calc: $[M]^+$ 419.1134, measured: 419.1131; difference = 0.7 ppm.

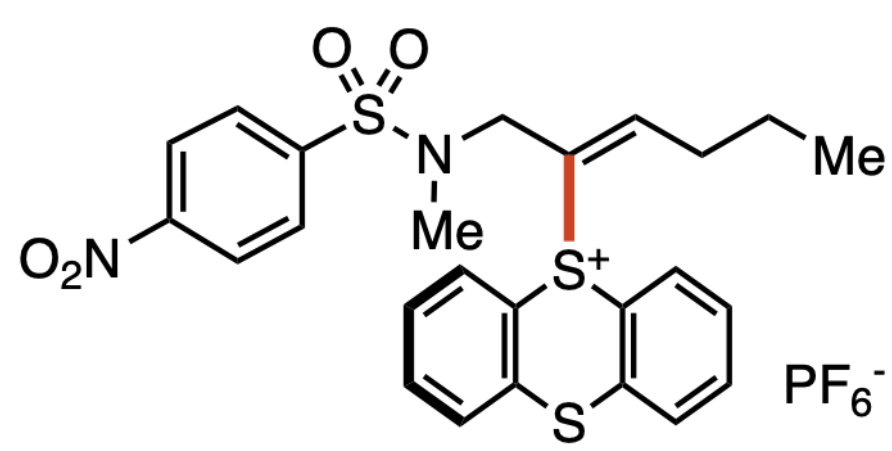

(*Z*)-5-(1-((*N*-methyl-4-nitrophenyl)sulfonamido)hex-2-en-2-yl)-5*H*-thianthren-5-ium hexafluorophosphate (4): Prepared according to **General Procedure B**. Purified by flash chromatography using C2 silica and acetone/hexanes eluent. Purified yield: 146 mg, 56%, 85:15 r.r.; 93:7 d.r. **^{1}H-NMR** (500 MHz, CD_3CN) δ 8.32 (d, *J* = 8.9 Hz, 1H), 8.09 (dd, *J* = 8.1, 1.4 Hz, 2H), 7.90 (dd, *J* = 8.0, 1.3 Hz, 2H), 7.82 (td, *J* = 7.7, 1.3 Hz, 2H), 7.78 (d, *J* = 8.9 Hz, 2H), 7.71 (td, *J* = 7.5, 1.4 Hz, 2H), 6.70 (td, *J* = 7.6, 1.2 Hz, 1H), 3.73 (d, *J* = 1.5 Hz, 2H), 2.55 (q, *J* = 7.3 Hz, 2H), 2.48 (s, 3H), 1.44 (h, *J* = 7.4 Hz, 2H), 0.94 (t, *J* = 7.4 Hz, 3H). **^{13}C-NMR** (126 MHz, CD_3CN) δ 153.65, 151.64, 142.36, 137.44, 135.71, 134.71, 131.29, 130.97, 129.84, 125.56, 117.71, 53.31, 35.62, 32.84, 22.33, 13.81. **^{19}F-NMR** (377 MHz, CD_3CN) δ -72.83 (d, *J* = 706.5 Hz). **^{31}P-NMR** (162 MHz, CD_3CN) δ -144.60 (hept, *J* = 706.7 Hz). **HRMS** (ESI+) calc: $[M]^+$ 513.0971, measured: 513.0962; difference = 1.8 ppm.

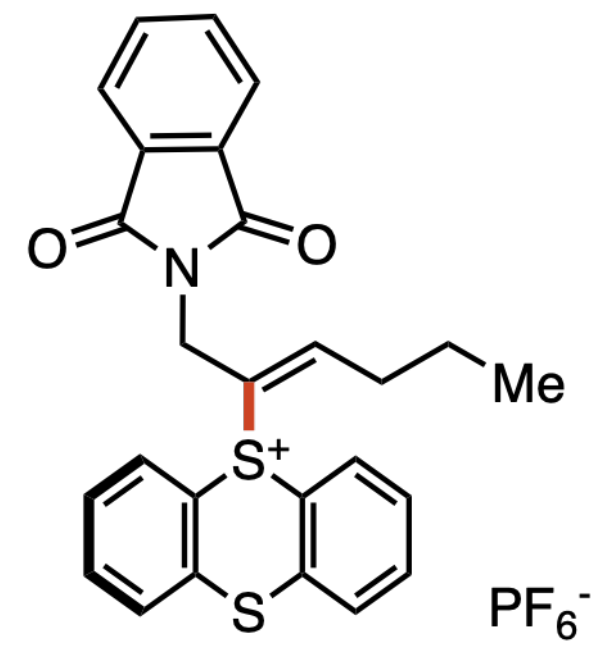

(*Z*)-5-(1-(1,3-dioxoisoindolin-2-yl)hex-2-en-2-yl)-5*H*-thianthren-5-ium hexafluorophosphate (5): Prepared according to **General Procedure B**. Purified by recrystallization from DCM/ethyl acetate. Purified yield: 168.4 mg, 71%, >98:2 r.r.; >98:2 d.r. **^{1}H-NMR** (500 MHz, CD_3CN) δ 8.07 (dd, *J* = 8.1, 1.4 Hz, 2H), 7.87 (dd, *J* = 7.9, 1.5 Hz, 2H), 7.82 – 7.75 (m, 6H), 7.67 (t, *J* = 7.8 Hz, 1H), 6.84 – 6.77 (m, 1H), 4.27 (s, 2H), 2.59 (q, *J* = 8.0 Hz, 2H), 1.43 (h, *J* = 7.4 Hz, 2H), 0.92 (td, *J* = 7.4, 1.2 Hz, 3H). **^{13}C-NMR** (126 MHz, DMSO) δ 166.89, 151.79, 134.58, 134.52, 134.34, 133.46, 131.44, 129.80, 129.78, 123.48, 123.16, 117.25, 38.59, 31.62, 21.04, 13.41. **^{19}F-NMR** (377 MHz, CD_3CN) δ -72.86 (d, *J* = 706.5 Hz).**^{31}P-NMR** (162 MHz, CD_3CN) δ -144.61 (hept, *J* = 706.5 Hz). **HRMS** (ESI+) calc: $[M]^+$ 444.1086, measured: 444.1088; difference = 0.4 ppm.

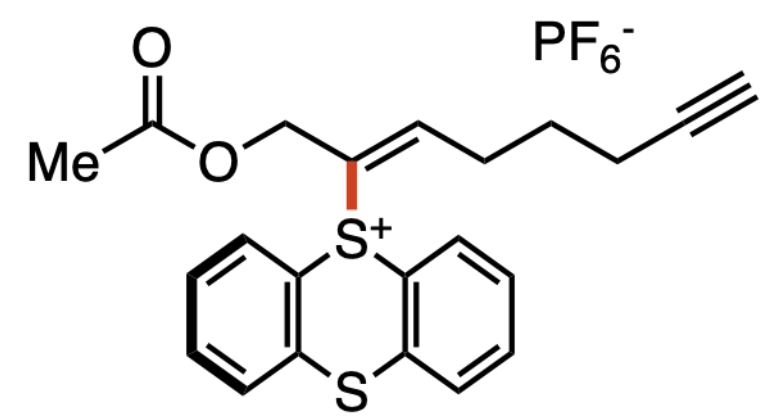

(*Z*)-5-(1-acetoxyoct-2-en-7-yn-2-yl)-5*H*-thianthren-5-ium hexafluorophosphate (7):
Prepared according to **General Procedure B**. Purified by flash chromatography using C2 silica and acetone/hexanes eluent. Purified yield: 149 mg, 71%, >98:2 r.r.; >98:2 d.r. **^{1}H-NMR** (500 MHz, CD_3CN) δ 8.14 (dd, *J* = 8.0, 1.3 Hz, 2H), 7.92 (dd, *J* = 8.1, 1.3 Hz, 2H), 7.84 (td, *J* = 7.7, 1.4 Hz, 2H), 7.73 (td, *J* = 7.7, 1.3 Hz, 2H), 6.88 (t, *J* = 7.7 Hz, 1H), 4.57 (s, 2H), 2.77 (q, *J* = 7.5 Hz, 2H), 2.34 (t, *J* = 2.7 Hz, 1H), 2.30 (td, *J* = 6.9, 2.6 Hz, 2H), 1.75 (s, 3H), 1.68 (p, *J* = 7.1 Hz, 2H). **^{13}C-NMR** (126 MHz, CD_3CN) δ 170.57, 155.98, 137.36, 135.72, 134.73, 131.05, 130.92, 126.42, 118.03, 84.17, 71.19, 65.30, 30.15, 27.44, 20.47, 18.19. **^{19}F-NMR** (377 MHz, CD_3CN) δ -72.93 (d, *J* = 706.3 Hz). **^{31}P-NMR** (162 MHz, CD_3CN) δ -144.63 (hept). **HRMS** (ESI+) calc: $[M]^+$ 381.0978, measured: 381.0978; difference = less than 0.1 ppm.

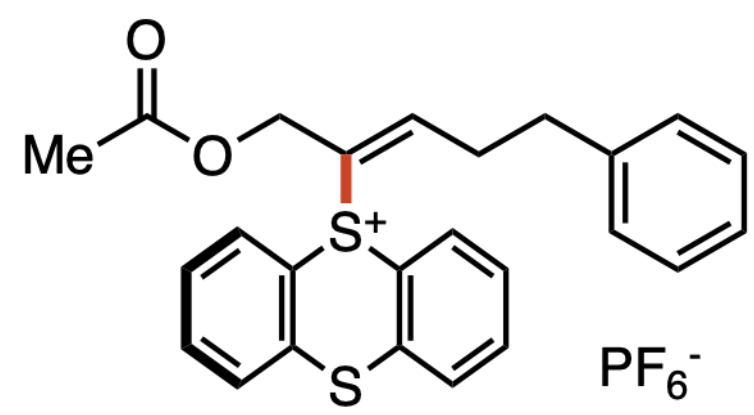

(*Z*)-5-(1-acetoxy-5-phenylpent-2-en-2-yl)-5*H*-thianthren-5-ium hexafluorophosphate (8): Prepared according to **General Procedure B** with the following modification: reaction was electrolyzed for 3.5 h (2.5 F/mol alkene). Purified by flash chromatography using C2 silica and acetone/hexanes eluent. Purified yield: 146 mg, 56%, >98:2 r.r. ; >98:2 d.r. **^{1}H-NMR** (500 MHz, CD_3CN) δ 7.82 (d, *J* = 8.0 Hz, 2H), 7.76 (dt, *J* = 8.2, 4.2 Hz, 2H), 7.60 (d, *J* = 4.2 Hz, 4H), 7.35 (dt, *J* = 13.2, 6.7 Hz, 2H), 7.25 (d, *J* = 6.3 Hz, 1H), 6.91 (t, *J* = 7.8 Hz, 1H), 4.54 (s, 2H), 3.03 (q, *J* = 7.2 Hz, 2H), 2.88 (t, *J* = 6.9 Hz, 2H), 1.66 (s, 3H). **^{13}C-NMR** (126 MHz, CD_3CN) δ 170.48, 156.63, 140.78, 136.90, 135.58, 134.26, 130.78, 130.77, 129.87, 129.76, 127.66, 127.08, 117.32, 64.94, 34.34, 33.15, 20.38. **^{19}F-NMR** (377 MHz, CD_3CN) δ -72.93 (d, *J* = 706.3 Hz). **^{31}P-NMR** (162 MHz, CD_3CN) δ -144.62 (hept, *J* = 708.1 Hz). **HRMS** (ESI+) calc: $[M]^+$ 419.1134, measured: 419.1129; difference = 1.2 ppm.

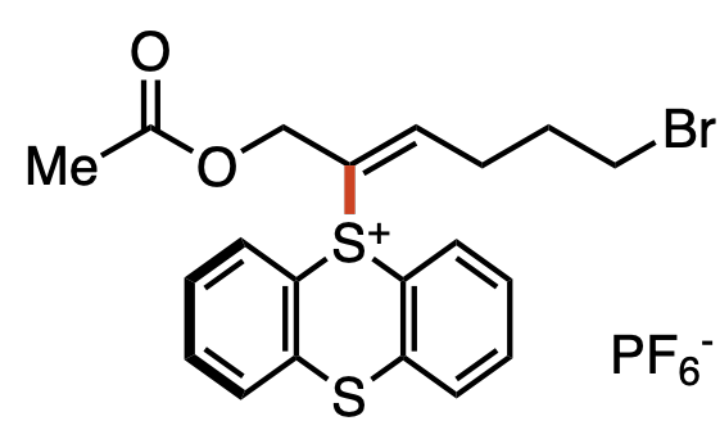

(*Z*)-5-(1-acetoxy-6-bromohex-2-en-2-yl)-5*H*-thianthren-5-ium hexafluorophosphate (10): Prepared according to **General Procedure B**. Purified by flash chromatography using C2 silica and acetone/hexanes eluent. Purified yield: 146 mg, 63%, >98:2 r.r.; >98:2 d.r. **^{1}H-NMR** (500 MHz, CD_3CN) δ 8.13 (dd, *J* = 8.0, 1.3 Hz, 2H), 7.90 (dd, *J* = 8.0, 1.4 Hz, 2H), 7.82 (td, *J* = 7.8, 1.3 Hz, 2H), 7.71 (td, *J* = 7.7, 1.4 Hz, 2H), 6.83 (t, *J* = 7.6 Hz, 1H), 4.55 (s, 2H), 3.51 (t, *J* = 6.4 Hz, 2H), 2.77 (q, *J* = 7.4 Hz, 2H), 2.00 (p, *J* = 6.7 Hz, 2H), 1.73 (s, 3H). **^{13}C-NMR** (126 MHz, CD_3CN) δ 170.58, 154.98, 137.41, 135.74, 134.77, 131.08, 130.93, 126.74, 118.00, 65.24, 33.82, 31.39, 29.80, 20.47. **^{19}F-NMR** (377 MHz, CD_3CN) δ -72.83 (d, *J* = 706.5 Hz). **^{31}P-NMR** (162 MHz, CD_3CN) δ -144.61 (hept, *J* = 706.5 Hz). **HRMS** (ESI+) calc: $[M]^+$ 435.0083, measured: 435.0083; difference = less than 0.1 ppm.

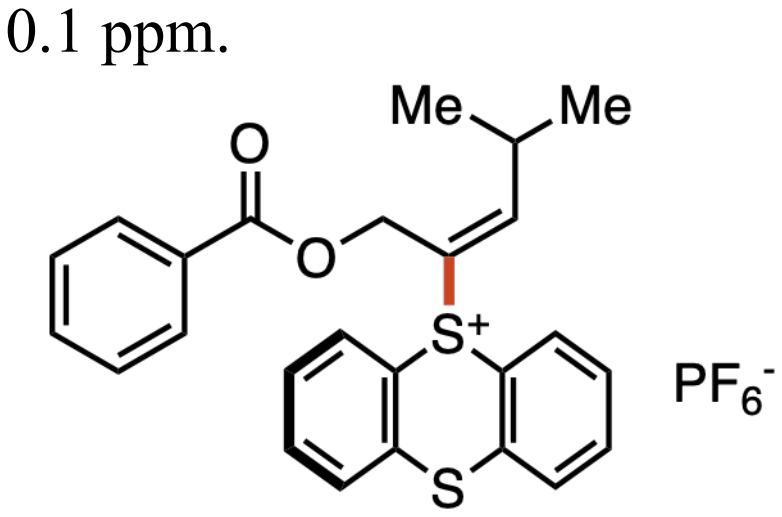

(*E*)-5-(1-(benzoyloxy)-4-methylpent-2-en-2-yl)-5*H*-thianthren-5-ium hexafluorophosphate (13): Prepared according to **General Procedure C** Purified by flash chromatography using C2 silica and a hexanes/acetone eluent. Isolated with a small amount (8%) of byproduct that arose

from rearrangement of the benzoyl group. Purified yield: 126 mg, 75%, >98:2 r.r.; 98:2 d.r. **^{1}H-NMR** (500 MHz, CD_3CN) δ 8.22 (dd, *J* = 8.0, 1.4 Hz, 2H), 7.92 (dd, *J* = 7.9, 1.3 Hz, 2H), 7.87 (d, *J* = 8.1 Hz, 1H), 7.80 (td, *J* = 7.7, 1.4 Hz, 2H), 7.76 – 7.64 (m, 4H), 7.53 (t, *J* = 7.8 Hz, 2H), 5.68 (d, *J* = 10.4 Hz, 1H), 4.79 (s, 2H), 2.95 (dp, *J* = 10.3, 6.6 Hz, 1H), 0.93 (d, *J* = 6.5 Hz, 6H). **^{13}C-NMR** (126 MHz, DMSO) δ 164.78, 154.69, 135.50, 135.48, 134.69, 133.95, 130.04, 129.62, 129.17, 128.82, 128.20, 117.78, 117.40, 58.08, 29.43, 21.46. **^{19}F-NMR** (471 MHz, CD_3CN) δ -72.89 (d, *J* = 706.2 Hz). **^{31}P-NMR** (202 MHz, CD_3CN) δ -144.61 (hept, *J* = 706.4 Hz). **HRMS** (ESI+) calc: $[M]^+$ 419.1134, measured: 419.1131; difference = 0.7 ppm.

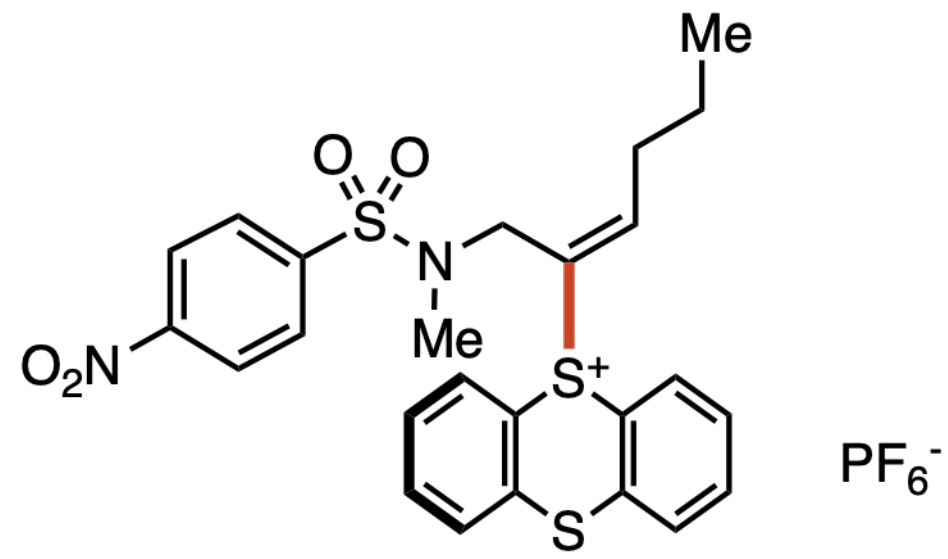

(*E*)-5-(1-((*N*-methyl-4-nitrophenyl)sulfonamido)hex-2-en-2-yl)-5*H*-thianthren-5-ium hexafluorophosphate (14): Prepared according to **General Procedure C**. Isolated by recrystallization from DCM/ethyl acetate. Purified yield: 137 mg, 52%, >98:2 r.r.; 98:2 d.r. **^{1}H-NMR** (500 MHz, CD_3CN) δ 8.38 (d, *J* = 8.9 Hz, 2H), 8.26 (d, *J* = 7.9 Hz, 2H), 7.99 (d, *J* = 7.9 Hz, 2H), 7.93 (d, *J* = 8.9 Hz, 2H), 7.88 (t, *J* = 7.7 Hz, 2H), 7.79 (t, *J* = 7.7 Hz, 2H), 5.68 (t, *J* = 7.8 Hz, 1H), 3.77 (s, 2H), 2.60 (s, 3H), 2.09 (q, *J* = 7.5 Hz, 2H), 1.24 (h, *J* = 7.5 Hz, 2H), 0.72 (t, *J* = 7.4 Hz, 3H). **^{13}C-NMR** (126 MHz, CD_3CN) δ 151.90, 148.57, 141.57, 138.29, 136.81, 135.96, 131.70, 130.98, 130.33, 125.70, 117.43, 117.09, 47.30, 35.12, 31.98, 22.23, 13.65. **^{19}F-NMR** (377 MHz, CD_3CN) δ -72.90 (d, *J* = 706.4 Hz). **^{31}P-NMR** (162 MHz, CD_3CN) δ -144.62 (hept, *J* = 708.1 Hz). **HRMS** (ESI+) calc: $[M]^+$ 513.0971, measured: 513.0972; difference = 0.2 ppm.

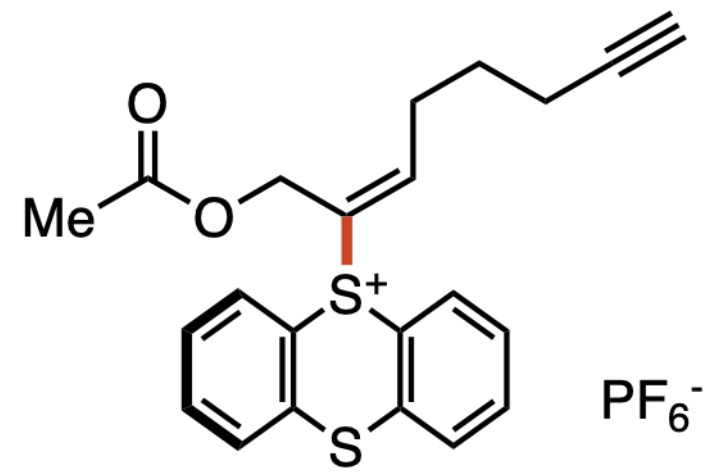

(*E*)-5-(1-acetoxyoct-2-en-7-yn-2-yl)-5*H*-thianthren-5-ium hexafluorophosphate (16): Prepared according to **General Procedure C**. Purified by flash chromatography using C2 silica and a hexanes/acetone eluent. Isolated with a small amount (6%) of byproduct that arose from rearrangement of the acetyl group. Purified yield: 140 mg, 67%, >98:2 r.r.; 98:2 d.r. **^{1}H NMR** (600 MHz, CD_3CN) δ 8.21 (dd, *J* = 7.9, 1.4 Hz, 2H), 7.97 (dd, *J* = 8.0, 1.3 Hz, 2H), 7.86 (td, *J* = 7.7, 1.4 Hz, 2H), 7.76 (td, *J* = 7.7, 1.3 Hz, 2H), 5.80 (td, *J* = 7.9, 2.1 Hz, 1H), 4.49 (s, 2H), 2.37 (q, *J* = 7.5 Hz, 2H), 2.16 (t, *J* = 2.7 Hz, 1H), 2.11 – 2.06 (m, 2H), 1.95 (s, 3H), 1.50 (p, *J* = 7.1 Hz, 2H). **^{13}C NMR** (151 MHz, CD_3CN) δ 170.76, 147.97, 137.48, 135.86, 135.60, 131.16, 130.41,

120.15, 117.48, 83.80, 70.21, 57.68, 29.03, 27.07, 20.15, 17.72. **^{19}F NMR** (376 MHz, CD_3CN) δ -72.92 (d, *J* = 706.4 Hz). **^{31}P NMR** (162 MHz, CD_3CN) δ -144.62 (hept, *J* = 706.4 Hz). **HRMS** (ESI+) calc: $[M]^+$ 381.0978, measured: 381.0974; difference = 1.0 ppm.

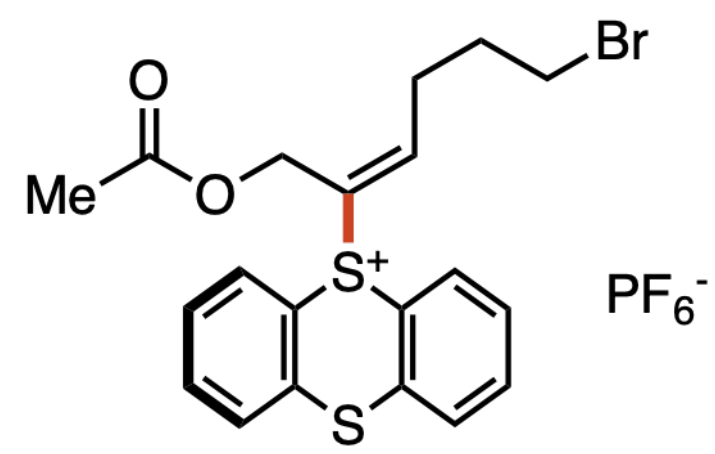

(*E*)-5-(1-acetoxy-6-bromohex-2-en-2-yl)-5*H*-thianthren-5-ium hexafluorophosphate (18): Prepared according to **General Procedure C**. Purified by flash chromatography using C2 silica and a hexanes/acetone eluent, followed by recrystallization from DCM/ethyl acetate. Purified yield: 139 mg, 60%, >98:2 r.r.; >98:2 d.r. **^{1}H-NMR** (500 MHz, CD_3CN) δ 8.21 (dd, *J* = 7.9, 1.4 Hz, 2H), 7.97 (dd, *J* = 8.0, 1.3 Hz, 2H), 7.86 (td, *J* = 7.7, 1.4 Hz, 2H), 7.76 (td, *J* = 7.7, 1.3 Hz, 2H), 5.80 (t, *J* = 7.9 Hz, 1H), 4.50 (s, 2H), 3.34 (t, *J* = 6.5 Hz, 2H), 2.42 (q, *J* = 7.5 Hz, 2H), 1.95 (s, 3H), 1.85 (p, *J* = 6.8 Hz, 2H). **^{13}C-NMR** (126 MHz, CD_3CN) δ 171.14, 147.58, 137.90, 136.29, 136.02, 131.58, 130.82, 120.95, 117.83, 58.11, 33.78, 31.49, 29.08, 20.54. **^{19}F-NMR** (377 MHz, CD_3CN) δ -72.89 (d, *J* = 706.1 Hz). **^{31}P-NMR** (162 MHz, CD_3CN) δ -144.62 (hept, *J* = 706.4 Hz). **HRMS** (ESI+) calc: $[M]^+$ 435.0083, measured: 435.0075; difference = 1.8 ppm.

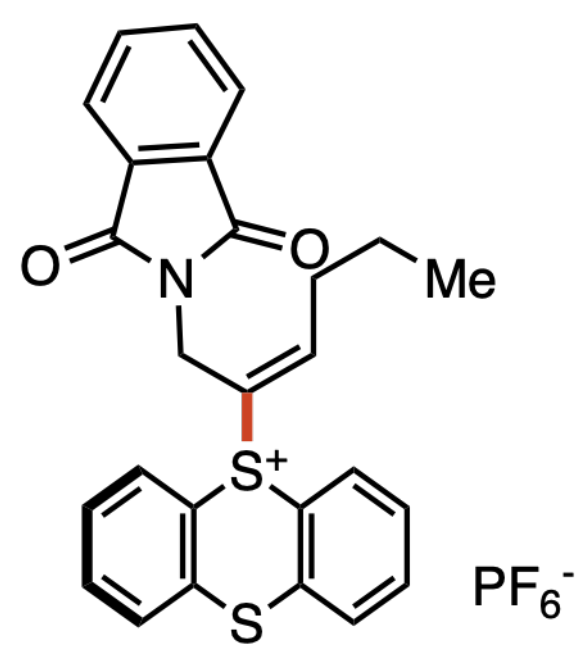

(*E*)-5-(1-(1,3-dioxoisoindolin-2-yl)hex-2-en-2-yl)-5*H*-thianthren-5-ium hexafluorophosphate (20): Prepared according to **3 mmol scale batch preparation**. Purified by recrystallization from DCM/ethyl acetate. Purified yield: 1.1 g, 63%, >98:2 r.r.; >98:2 d.r. **^{1}H-NMR** (500 MHz, CD_3CN) δ 8.33 (dd, *J* = 7.9, 1.4 Hz, 2H), 7.95 (dd, *J* = 8.0, 1.3 Hz, 2H), 7.92 – 7.88 (m, 2H), 7.86 (dt, *J* = 5.1, 3.7 Hz, 2H), 7.83 (td, *J* = 7.7, 1.4 Hz, 2H), 7.75 (td, *J* = 7.7, 1.3 Hz, 2H), 5.66 (t, *J* = 7.7 Hz, 1H), 4.22 (s, 2H), 2.41 (q, *J* = 7.4 Hz, 2H), 1.32 (h, *J* = 7.4 Hz, 2H), 0.79 (t, *J* = 7.4 Hz, 3H). **^{13}C-NMR** (126 MHz, CD_3CN) δ 168.43, 148.32, 137.65, 136.16, 135.48, 135.45, 132.41, 131.18, 130.44, 124.03, 117.24, 117.18, 33.67, 32.00, 21.83, 13.40. **^{19}F-NMR** (377 MHz, CD_3CN) δ -72.84 (d, *J* = 706.4 Hz). **^{31}P-NMR** (162 MHz, CD_3CN) δ -144.61 (hept, *J* = 706.5 Hz). **HRMS** (ESI+) calc: $[M]^+$ 444.1087, measured: 444.1080; difference = 1.6 ppm.

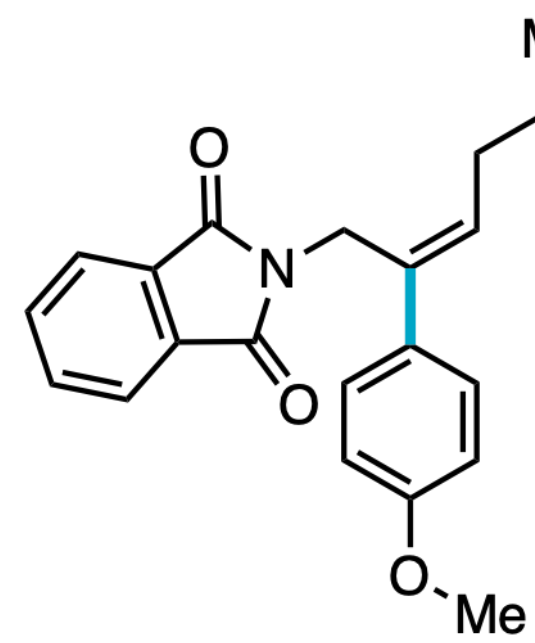

(*Z*)-2-(2-(4-methoxyphenyl)hex-2-en-1-yl)isoindoline-1,3-dione (21): Prepared according to **General Procedure D**. Purified by flash chromatography using ethyl acetate/hexanes. Purified yield: 50 mg, 75%, >98:2 r.r.; >98:2 d.r. **^{1}H-NMR** (500 MHz, $CDCl_3$) δ 7.72 (dd, *J* = 5.5, 3.1 Hz, 2H), 7.62 (dd, *J* = 5.5, 3.0 Hz, 2H), 7.29 (d, *J* = 8.8 Hz, 1H), 6.85 – 6.65 (m, 2H), 5.78 (t, *J* = 7.3 Hz, 1H), 4.74 (s, 2H), 3.73 (s, 3H), 2.44 (q, *J* = 7.4 Hz, 2H), 1.57 (dq, *J* = 14.7, 7.4 Hz, 3H), 1.02 (t, *J* = 7.4 Hz, 3H). **^{13}C-NMR** (126 MHz, $CDCl_3$) δ 168.45, 159.11, 134.22, 134.12, 133.30, 133.27, 132.38, 128.44, 123.47, 113.85, 55.55, 37.17, 31.02, 23.31, 14.36. **HRMS** (ESI+) calc: $[M+H]^+$ 336.1594, measured: 336.1583; difference = 3.2 ppm.

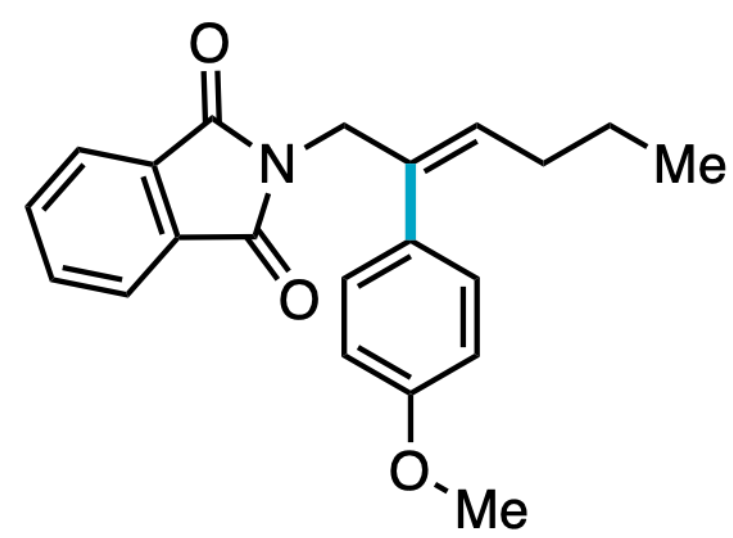

(*E*)-2-(2-(4-methoxyphenyl)hex-2-en-1-yl)isoindoline-1,3-dione (22): Prepared according to **General Procedure D**. Purified by flash chromatography using ethyl acetate/hexanes. Purified yield: 64 mg, 95%, >98:2 r.r.; >98:2 d.r. **^{1}H-NMR** (500 MHz, $CDCl_3$) δ 7.80 (dd, *J* = 5.4, 3.1 Hz, 2H), 7.68 (dd, *J* = 5.5, 3.0 Hz, 2H), 7.20 – 7.04 (m, 2H), 6.83 (d, *J* = 8.6 Hz, 2H), 5.58 (t, *J* = 7.3 Hz, 1H), 4.50 (s, 2H), 3.78 (s, 3H), 1.94 (q, *J* = 7.4 Hz, 2H), 1.33 (h, *J* = 7.4 Hz, 2H), 0.81 (t, *J* = 7.3 Hz, 3H). **^{13}C-NMR** (126 MHz, $CDCl_3$) δ 168.13, 158.73, 134.52, 133.96, 132.20, 130.51, 130.04, 129.70, 123.37, 113.67, 55.28, 44.65, 30.85, 22.99, 13.86. **HRMS** (ESI+) calc: $[M+H]^+$ 336.1594, measured: 336.1582; difference = 3.6 ppm.

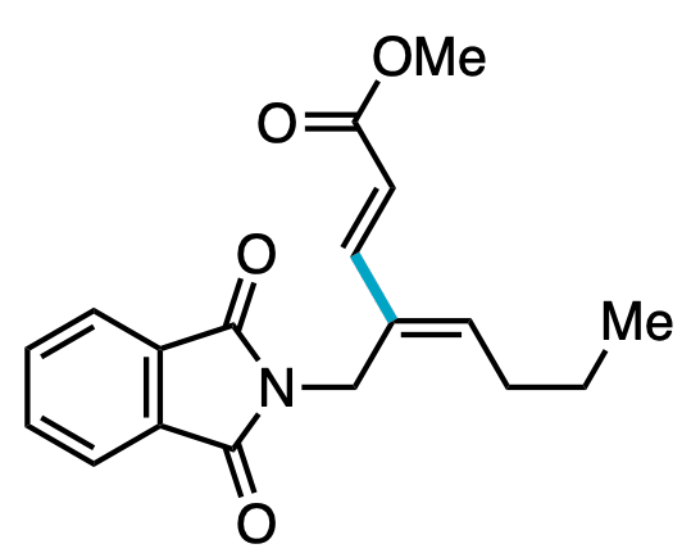

Methyl (2*E*,4*Z*)-4-((1,3-dioxoisoindolin-2-yl)methyl)octa-2,4-dienoate (23): Prepared according to **General Procedure E**. Purified by flash chromatography using ethyl acetate/hexanes. Purified yield: 41 mg, 65%, >98:2 r.r.; >98:2 d.r. **^{1}H-NMR** (500 MHz, $CDCl_3$) δ 7.83 (dd, *J* = 5.4, 3.1 Hz, 2H), 7.70 (dd, *J* = 5.5, 3.0 Hz, 2H), 7.24 (d, *J* = 16.1 Hz, 1H), 6.22 – 6.11 (m, 2H), 4.50 (s, 2H), 3.70 (s, 3H), 2.54 (q, *J* = 7.5 Hz, 2H), 1.60 – 1.49 (m, 2H), 1.00 (t, *J* = 7.4 Hz, 3H). **^{13}C-NMR** (126 MHz, $CDCl_3$) δ 168.25, 168.01, 147.49, 146.83, 134.19, 132.08, 130.98, 123.48, 116.32, 51.62, 34.29, 31.22, 22.46, 14.06. **HRMS** (ESI+) calc: $[M+H]^+$ 314.1387, measured: 314.1379; difference = 2.5 ppm.

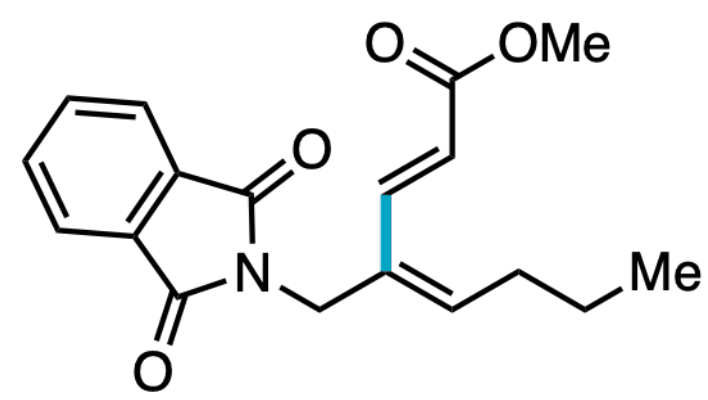

methyl (2*E*,4*E*)-4-((1,3-dioxoisoindolin-2-yl)methyl)octa-2,4-dienoate (24): Prepared according to **General Procedure E**. Purified by flash chromatography using ethyl acetate/hexanes. Purified yield: 42 mg, 67%, >98:2 r.r.; >98:2 d.r. **^{1}H-NMR** (500 MHz, $CDCl_3$) δ 7.85 (dd, *J* = 5.4, 3.0 Hz, 2H), 7.72 (dd, *J* = 5.5, 3.0 Hz, 2H), 7.66 (d, *J* = 16.0 Hz, 1H), 6.25 (d, *J* = 16.0 Hz, 1H), 6.02 (t, *J* = 7.8 Hz, 1H), 4.44 (s, 2H), 3.76 (s, 3H), 2.30 (q, *J* = 7.5 Hz, 2H), 1.45 (h, *J* = 7.4 Hz, 2H), 0.91 (t, *J* = 7.4 Hz, 3H). **^{13}C-NMR** (126 MHz, $CDCl_3$) δ 168.21, 167.93, 142.65, 138.76, 134.21, 132.16, 129.23, 123.57, 118.36, 51.78, 40.46, 30.17, 22.64, 13.83. **HRMS** (ESI+) calc: $[M+H]^+$ 314.1387, measured: 314.1377; difference = 3.2 ppm.

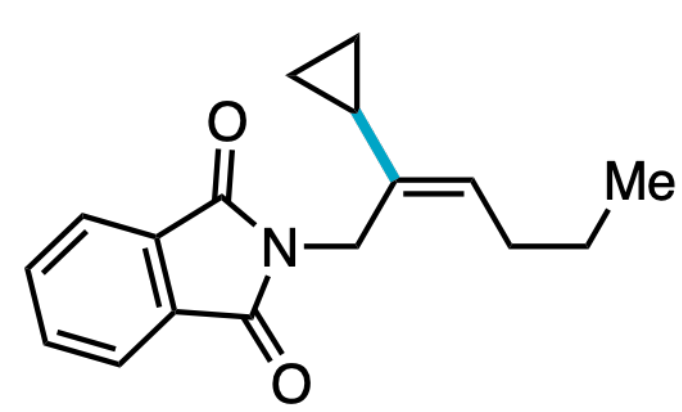

(*Z*)-2-(2-cyclopropylhex-2-en-1-yl)isoindoline-1,3-dione (25) Prepared according to **General Procedure F**. Purified by flash chromatography using ethyl acetate/hexanes. Purified yield: 37 mg, 68%, >98:2 r.r.; >98:2 d.r. **^{1}H-NMR** (500 MHz, $CDCl_3$) δ 7.84 (dd, *J* = 5.5, 3.1 Hz, 2H), 7.71 (dd, *J* = 5.5, 3.1 Hz, 2H), 5.28 (t, *J* = 7.2 Hz, 1H), 4.43 (s, 2H), 2.23 (q, *J* = 7.4 Hz, 2H), 1.43 (h, *J* = 7.4 Hz, 2H), 1.22 (q, *J* = 7.2 Hz, 1H), 0.94 (t, *J* = 7.3 Hz, 3H), 0.48 – 0.37 (m, 2H), 0.31 – 0.17 (m, 2H). **^{13}C-NMR** (126 MHz, $CDCl_3$) δ 168.81, 134.59, 134.25, 132.60, 127.56, 123.56, 38.86, 29.96, 23.30, 15.26, 14.25, 5.22. **HRMS** (ESI+) calc: $[M+H]^+$ 270.1489, measured: 270.1486; difference = 1.1 ppm.

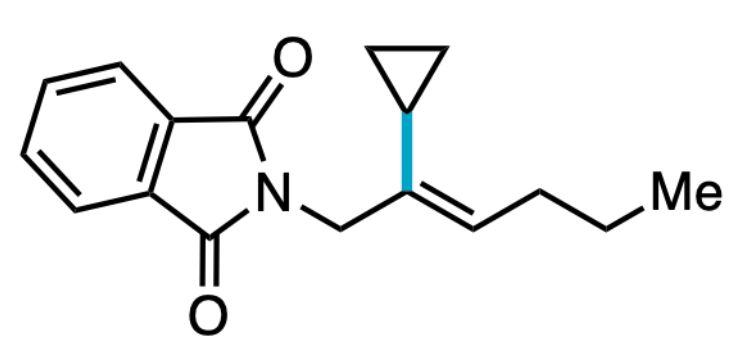

(*E*)-2-(2-cyclopropylhex-2-en-1-yl)isoindoline-1,3-dione (26): Prepared according to **General Procedure F**. Purified by flash chromatography using ethyl acetate/hexanes. Purified yield: 39 mg, 73%, >98:2 r.r.; >98:2 d.r. **^{1}H-NMR** (500 MHz, $CDCl_3$) δ 7.85 (dd, *J* = 5.5, 3.0 Hz, 2H), 7.71 (dd, *J* = 5.5, 3.1 Hz, 2H), 5.28 (t, *J* = 7.2 Hz, 1H), 4.08 (s, 2H), 2.15 (q, *J* = 7.4 Hz, 2H), 1.42 (q, *J* = 6.9 Hz, 1H), 1.35 (h, *J* = 7.3 Hz, 2H), 0.87 (t, *J* = 7.3 Hz, 3H), 0.74 – 0.64 (m, 2H), 0.60 – 0.52 (m, 2H). **^{13}C-NMR** (126 MHz, $CDCl_3$) δ 168.31, 134.01, 132.40, 132.34, 129.22, 123.40, 41.70, 29.84, 22.81, 13.99, 10.97, 4.99. **HRMS** (ESI+) calc: $[M+H]^+$ 270.1489, measured: 270.1487; difference = 0.7 ppm.

6. X-ray Crystallography Data

Data Collection.

A brown crystal with approximate dimensions 0.113 x 0.068 x 0.089 mm^3 was selected under oil under ambient conditions and attached to the tip of a MiTeGen MicroMount©. The crystal was mounted in a stream of cold nitrogen at 100(1) K and centered in the X-ray beam by using a video camera.

The crystal evaluation and data collection were performed on a Bruker D8 VENTURE Photon III four-circle diffractometer with Diamond II IμS Mo Kα (λ = 0.71073 Å) radiation and the detector to crystal distance of 5.0 cm[1].

The initial cell constants were obtained from a 180° φ scan conducted at a 2θ = 30° angle with the exposure time of 1 second per frame. The reflections were successfully indexed by an automated indexing routine built in the APEX6 program. The final cell constants were calculated from a set of 9867 strong reflections from the actual data collection.

The data were collected by using the half sphere data collection routine to survey the reciprocal space to the extent of a half sphere to a resolution of 0.75 Å. A total of 60795 data were harvested by collecting 6 sets of frames with 0.7º scans in ω and φ with an exposure time of 4–8 sec per frame. These highly redundant datasets were corrected for Lorentz and polarization effects. The absorption correction was based on fitting a function to the empirical transmission surface as sampled by multiple equivalent measurements[2].

Structure Solution and Refinement.

The systematic absences in the diffraction data were uniquely consistent for the space group $P2_1/n$ that yielded chemically reasonable and computationally stable results of refinement [3-8].

A successful solution by intrinsic phasing provided most non-hydrogen atoms from the *E*-map. The remaining non-hydrogen atoms were located in an alternating series of least-squares cycles and difference Fourier maps. All non-hydrogen atoms were refined with anisotropic displacement coefficients. All hydrogen atoms were included in the structure factor calculation at idealized positions and were allowed to ride on the neighboring atoms with relative isotropic displacement coefficients.

The asymmetric unit contains one formula unit of $[C_{26}H_{22}NO_2S_2][PF_6]$.

The final least-squares refinement of 344 parameters against 6298 data resulted in residuals R (based on F^2 for $I \geq 2\sigma$) and wR (based on F^2 for all data) of 0.0353 and 0.0926, respectively. The final difference Fourier map was featureless.

Summary

Crystal Data for $C_{26}H_{22}F_6NO_2PS_2$ (M =589.53 g/mol): monoclinic, space group $P2_1/n$ (no. 14), a = 11.176(2) Å, b = 14.583(5) Å, c = 15.834(3) Å, β = 100.940(15)°, V = 2533.7(11) Å^3, Z = 4, T = 100.00 K, μ(MoKα) = 0.344 mm^{-1}, $Dcalc$ = 1.546 g/cm^3, 59524 reflections measured (3.83° ≤ 2Θ ≤ 56.662°), 6298 unique (R_{int} = 0.0724, R_{sigma} = 0.0402) which were used in all calculations. The final R_1 was 0.0353 (I > 2σ(I)) and wR_2 was 0.0926 (all data).

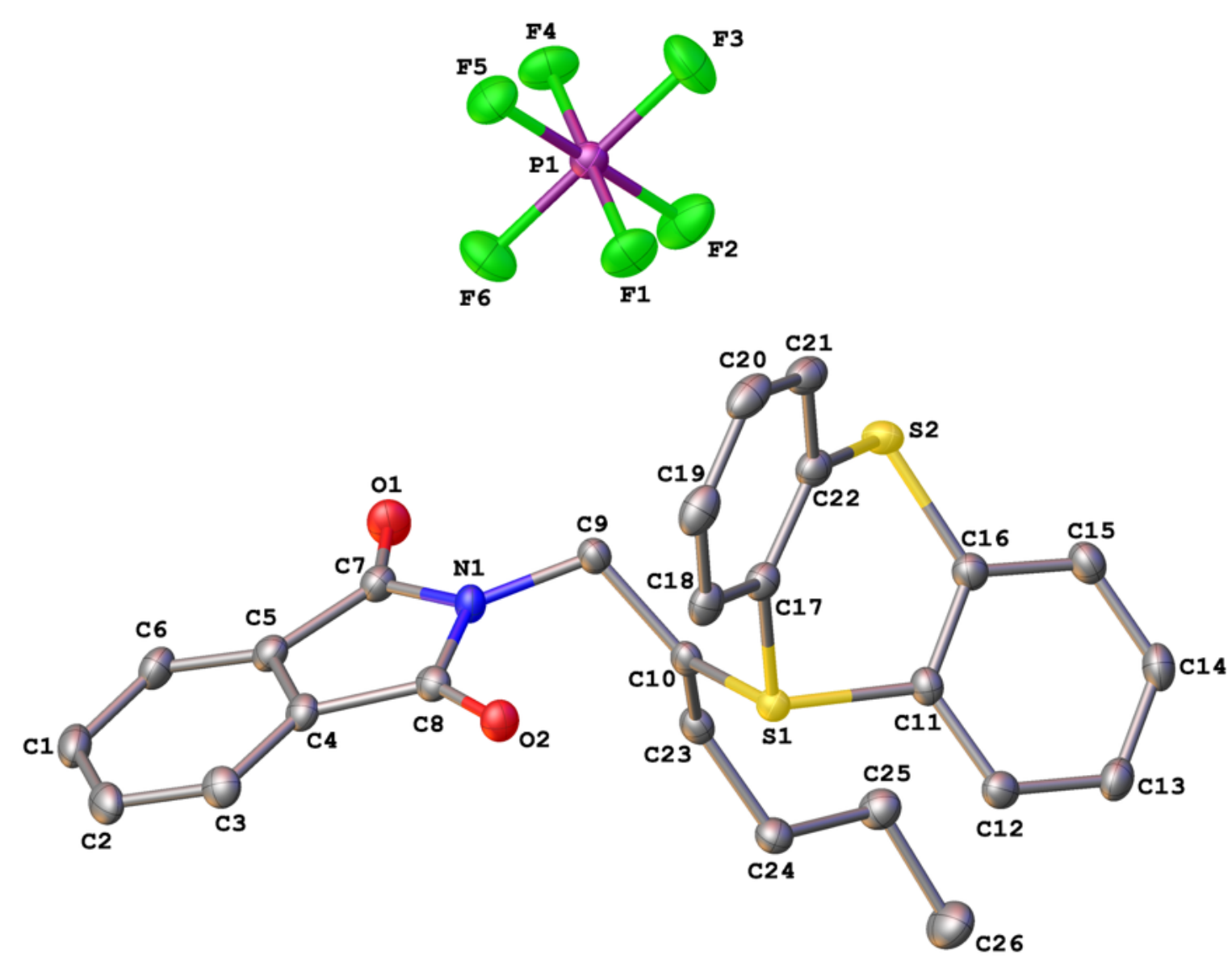

Figure S11. A molecular drawing of **5** shown with 50% probability ellipsoids. All H atoms are omitted.

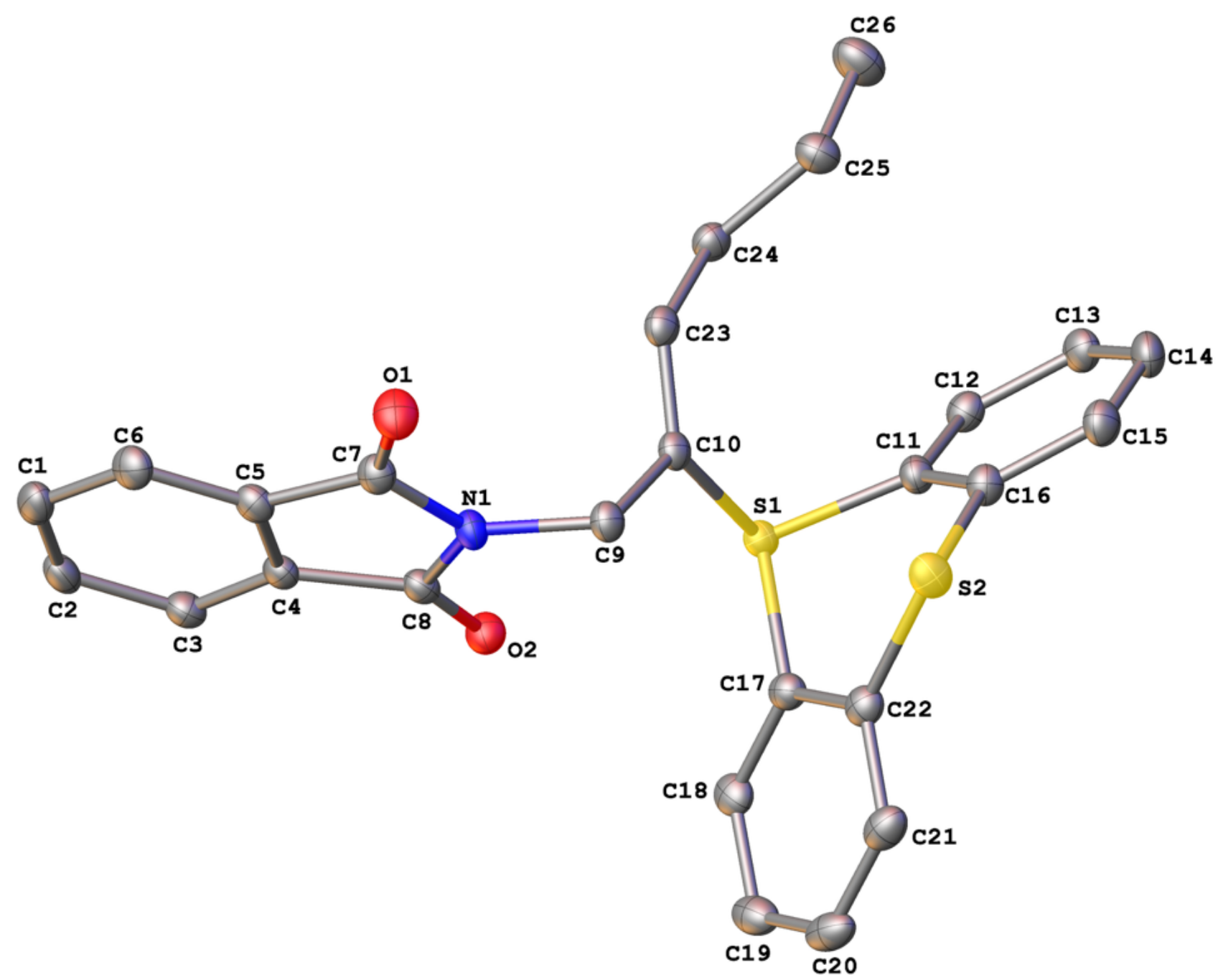

Figure S12. A molecular drawing of the cation in **5** shown with 50% probability ellipsoids. All H atoms and the anion are omitted.

Table S10. Crystal data and structure refinement for **5.**

Identification code	Wickens40
Empirical formula	$[C_{26}H_{22}NO_2S_2][PF_6]$
Formula weight	589.53
Temperature/K	100.00
Crystal system	monoclinic
Space group	$P2_1/n$
a/Å	11.176(2)
b/Å	14.583(5)
c/Å	15.834(3)
α/°	90
β/°	100.940(15)
γ/°	90
Volume/Å^3	2533.7(11)
Z	4
ρ_{calc}g/cm^3	1.546
μ/mm^{-1}	0.344
F(000)	1208.0
Crystal size/mm^3	0.113 × 0.089 × 0.068
Radiation	MoKα (λ = 0.71073)
2Θ range for data collection/°	3.83 to 56.662
Index ranges	$-14 \leq h \leq 14$, $-19 \leq k \leq 19$, $-21 \leq l \leq 20$
Reflections collected	59524
Independent reflections	6298 [R_{int} = 0.0724, R_{sigma} = 0.0402]
Data/restraints/parameters	6298/0/344
Goodness-of-fit on F^2	1.031
Final R indexes [I>=2σ (I)]	R_1 = 0.0353, wR_2 = 0.0853
Final R indexes [all data]	R_1 = 0.0449, wR_2 = 0.0926
Largest diff. peak/hole / e Å^{-3}	0.48/-0.27

Table S11. Bond Lengths for **5**.

**Atom**	**Atom**	**Length/Å**	**Atom**	**Atom**	**Length/Å**
S1	C10	1.8105(15)	C11	C16	1.397(2)
S1	C11	1.7772(16)	C12	C13	1.386(2)
S1	C17	1.7741(16)	C13	C14	1.388(2)
S2	C16	1.7619(16)	C14	C15	1.388(2)
S2	C22	1.7640(17)	C15	C16	1.393(2)
O1	C7	1.2065(19)	C17	C18	1.390(2)
O2	C8	1.2111(19)	C17	C22	1.396(2)
N1	C7	1.4035(19)	C18	C19	1.383(2)
N1	C8	1.3957(19)	C19	C20	1.391(3)
N1	C9	1.4564(19)	C20	C21	1.386(2)
C1	C2	1.389(2)	C21	C22	1.392(2)
C1	C6	1.399(2)	C23	C24	1.495(2)
C2	C3	1.397(2)	C24	C25	1.537(2)
C3	C4	1.381(2)	C25	C26	1.524(2)
C4	C5	1.388(2)	P1	F1	1.6056(10)
C4	C8	1.490(2)	P1	F2	1.5929(11)
C5	C6	1.380(2)	P1	F3	1.6007(12)
C5	C7	1.488(2)	P1	F4	1.5973(10)
C9	C10	1.516(2)	P1	F5	1.5982(11)
C10	C23	1.327(2)	P1	F6	1.5977(11)
C11	C12	1.392(2)			

Table S12. Bond Angles for **5.**

**Atom**	**Atom**	**Atom**	**Angle/°**	**Atom**	**Atom**	**Atom**	**Angle/°**
C11	S1	C10	102.58(7)	C14	C15	C16	119.74(15)
C17	S1	C10	105.67(7)	C11	C16	S2	122.33(12)
C17	S1	C11	99.99(7)	C15	C16	S2	119.02(12)
C16	S2	C22	99.95(7)	C15	C16	C11	118.65(14)
C7	N1	C9	124.86(13)	C18	C17	S1	117.37(12)
C8	N1	C7	112.07(12)	C18	C17	C22	122.17(15)
C8	N1	C9	122.89(12)	C22	C17	S1	120.40(12)
C2	C1	C6	121.01(15)	C19	C18	C17	118.67(15)

Table S12. Bond Angles for **5.**

**Atom**	**Atom**	**Atom**	**Angle/˚**	**Atom**	**Atom**	**Atom**	**Angle/˚**
C1	C2	C3	121.60(15)	C18	C19	C20	120.03(16)
C4	C3	C2	116.83(15)	C21	C20	C19	120.85(16)
C3	C4	C5	121.68(14)	C20	C21	C22	120.05(16)
C3	C4	C8	130.21(14)	C17	C22	S2	122.54(12)
C5	C4	C8	108.04(13)	C21	C22	S2	119.21(13)
C4	C5	C7	108.50(13)	C21	C22	C17	118.21(15)
C6	C5	C4	121.90(14)	C10	C23	C24	130.07(14)
C6	C5	C7	129.51(14)	C23	C24	C25	112.71(13)
C5	C6	C1	116.96(15)	C26	C25	C24	111.30(14)
O1	C7	N1	125.01(14)	F2	P1	F1	90.06(6)
O1	C7	C5	129.50(15)	F2	P1	F3	90.70(7)
N1	C7	C5	105.48(12)	F2	P1	F4	90.18(6)
O2	C8	N1	123.89(14)	F2	P1	F5	179.44(6)
O2	C8	C4	130.27(14)	F2	P1	F6	89.71(7)
N1	C8	C4	105.82(13)	F3	P1	F1	90.18(7)
N1	C9	C10	110.92(12)	F4	P1	F1	179.74(7)
C9	C10	S1	119.51(11)	F4	P1	F3	89.94(6)
C23	C10	S1	117.88(12)	F4	P1	F5	90.32(6)
C23	C10	C9	122.22(14)	F4	P1	F6	90.44(6)
C12	C11	S1	117.82(12)	F5	P1	F1	89.44(6)
C12	C11	C16	121.72(14)	F5	P1	F3	89.56(7)
C16	C11	S1	120.45(12)	F6	P1	F1	89.45(6)
C13	C12	C11	118.57(14)	F6	P1	F3	179.44(6)
C12	C13	C14	120.27(15)	F6	P1	F5	90.02(6)
C15	C14	C13	120.85(15)				

References

1. Bruker AXS LLC (2024). *APEX6.* Version 2024.9-0. Madison, Wisconsin, USA.
2. Krause, L., Herbst-Irmer, R., Sheldrick, G. M. & Stalke, D **2015**, *J. Appl. Cryst. 48*. 3-10.
3. Sheldrick, G. M. **2013b**. *XPREP*. Version 2013/1. Georg-August-Universität Göttingen, Göttingen, Germany.
4. Sheldrick, G. M. **2013a**. The *SHELX* homepage, http://shelx.uni-ac.gwdg.de/SHELX/.
5. Sheldrick, G. M. **2015a**. *Acta Cryst. A*, 71, 3-8.
6. Sheldrick, G. M. **2015b**. *Acta Cryst. C*, 71, 3-8.
7. Dolomanov, O. V., Bourhis, L. J., Gildea, R. J., Howard, J. A. K. & Puschmann, H **2009**. *J. Appl. Crystallogr. 42*, 339-341.
8. Guzei, I. A. **2007-2022**. Programs *Gn*. University of Wisconsin-Madison, Madison, Wisconsin, USA.

7. NMR Spectra

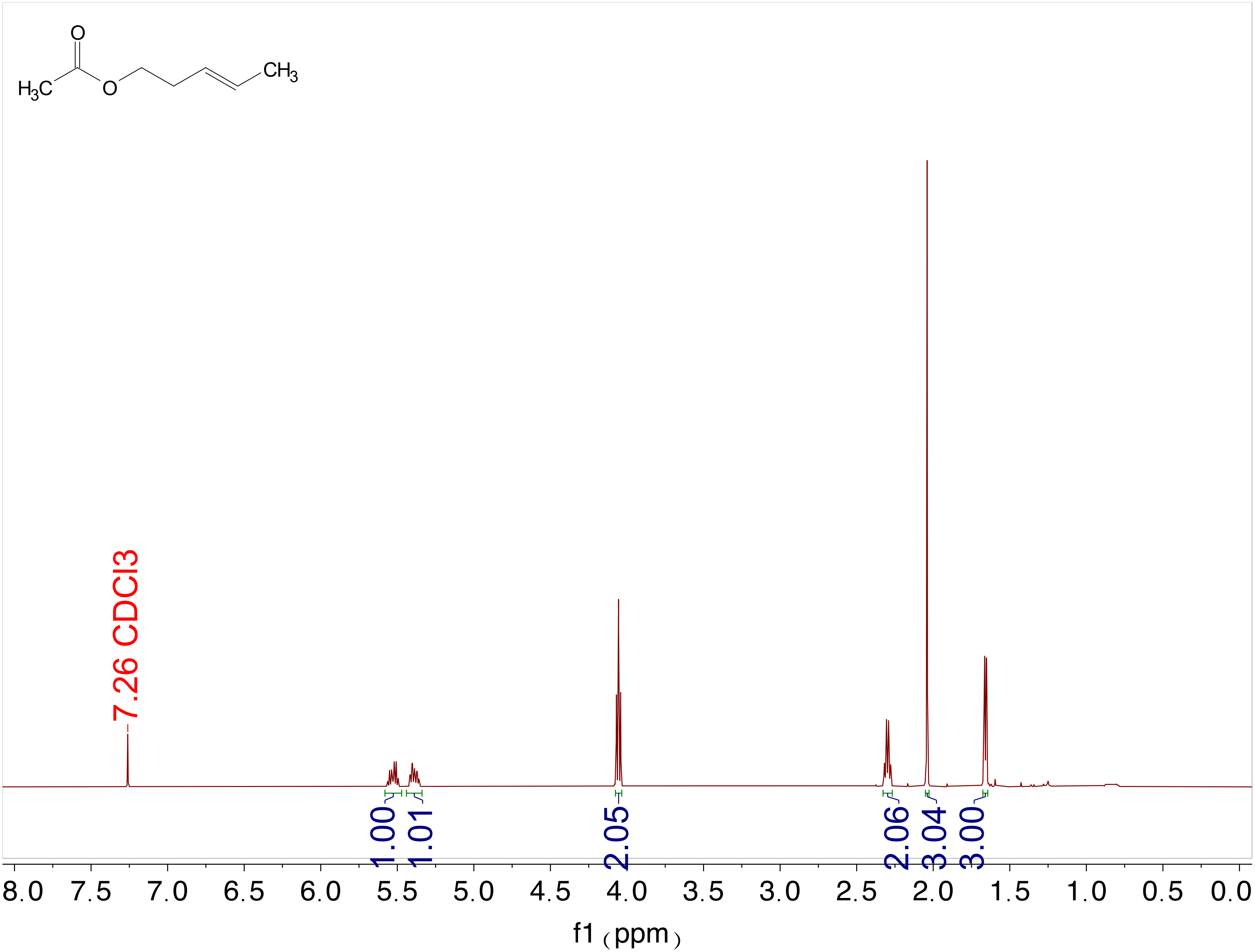

H3C
O
O
CH3
7.26 CDCl3
1.00
1.01
2.05
2.06
3.04
3.00
8.0 7.5 7.0 6.5 6.0 5.5 5.0 4.5 4.0 3.5 3.0 2.5 2.0 1.5 1.0 0.5 0.0
f1 (ppm)

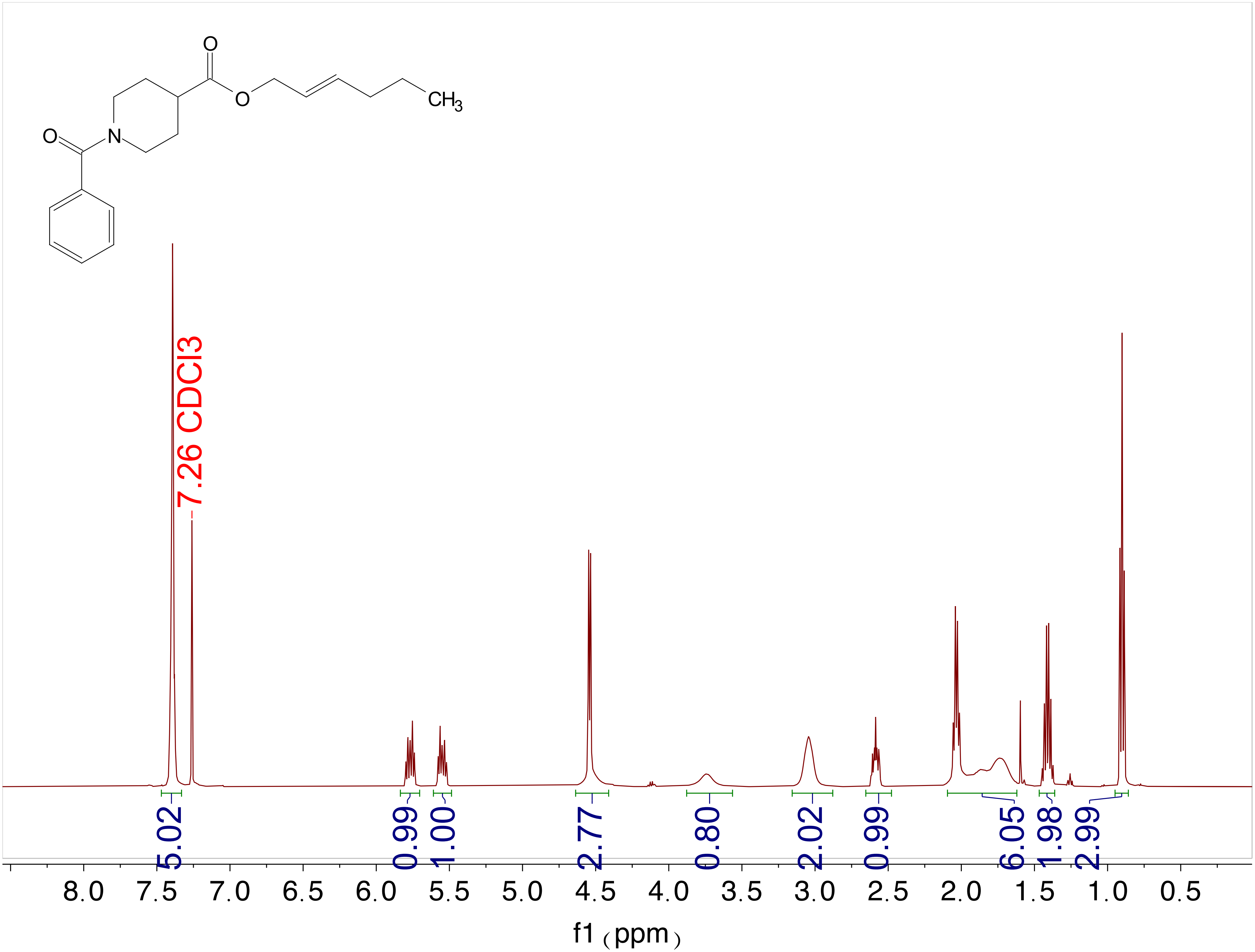

O
O
O
N
CH_3
7.26 CDCl3
5.02
0.99
1.00
2.77
0.80
2.02
0.99
6.05
1.98
2.99
8.0 7.5 7.0 6.5 6.0 5.5 5.0 4.5 4.0 3.5 3.0 2.5 2.0 1.5 1.0 0.5
f1（ppm）

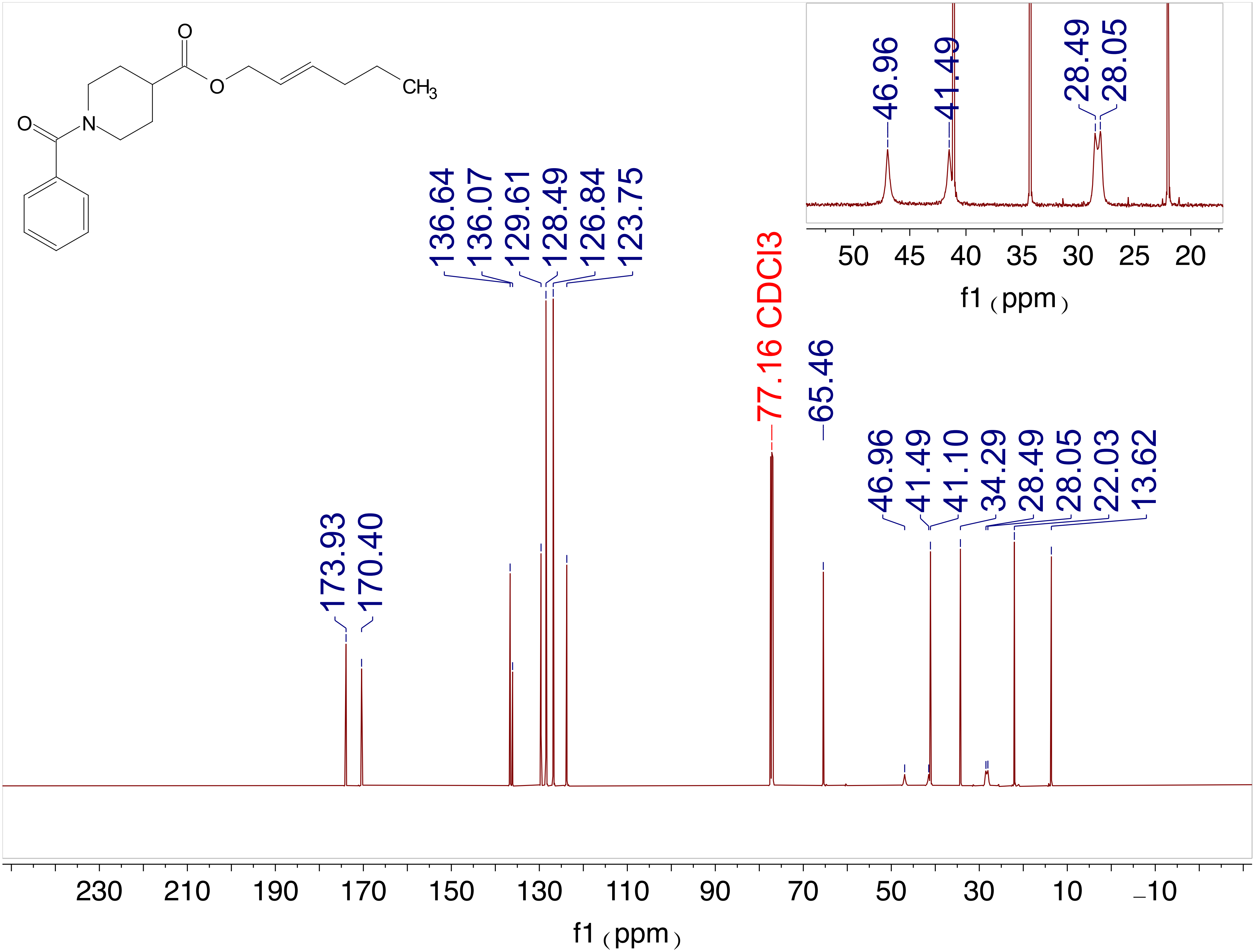

O
O
O
N
CH3
173.93
170.40
136.64
136.07
129.61
128.49
126.84
123.75
77.16 CDCl3
65.46
46.96
41.49
41.10
34.29
28.49
28.05
22.03
13.62
230 210 190 170 150 130 110 90 70 50 30 10 −10
f1 (ppm)
50 45 40 35 30 25 20
f1 (ppm)

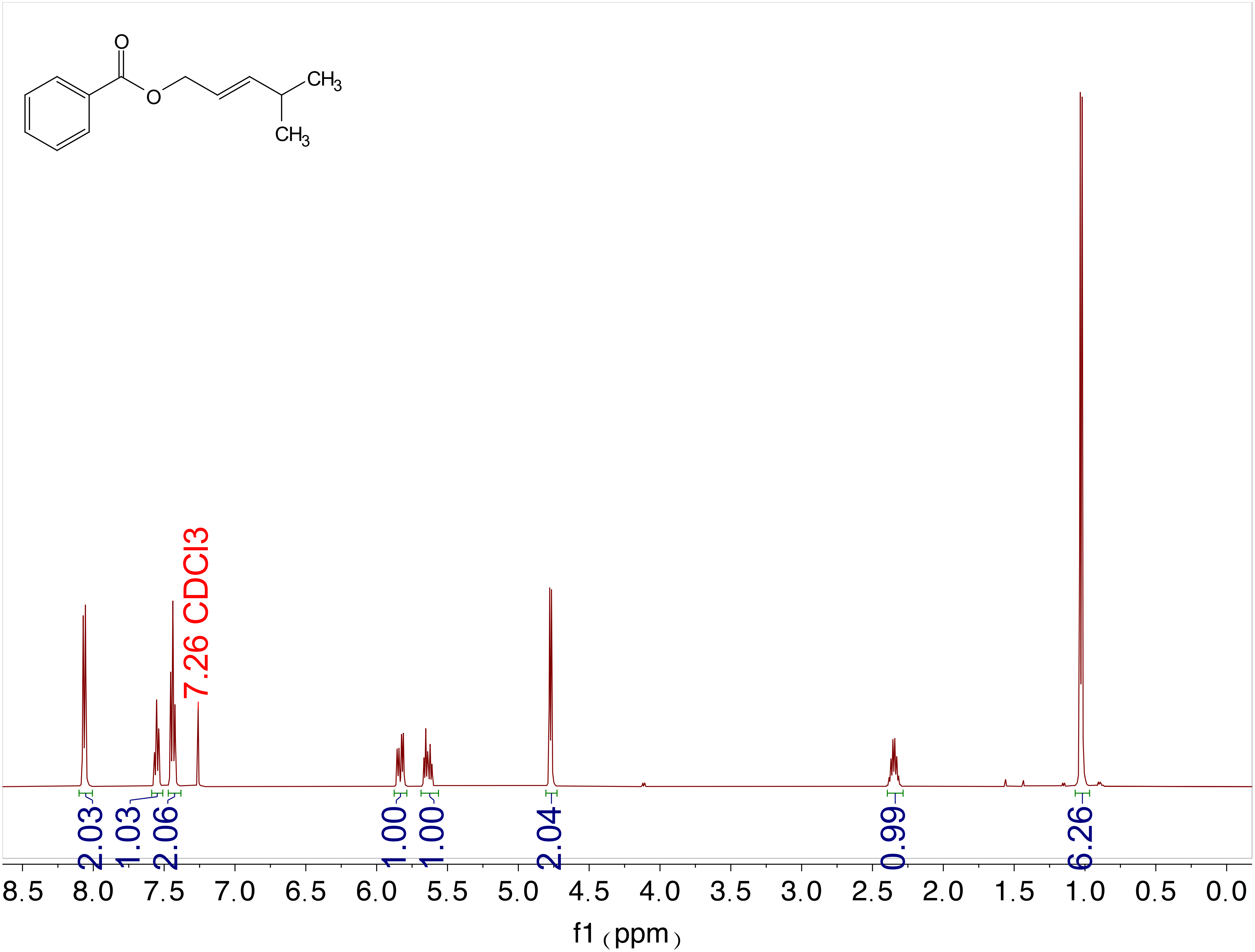

CH3
CH3
O
O
7.26 CDCl3
2.03
1.03
2.06
1.00
1.00
2.04
0.99
6.26
8.5
8.0
7.5
7.0
6.5
6.0
5.5
5.0
4.5
4.0
3.5
3.0
2.5
2.0
1.5
1.0
0.5
0.0
f1 (ppm)

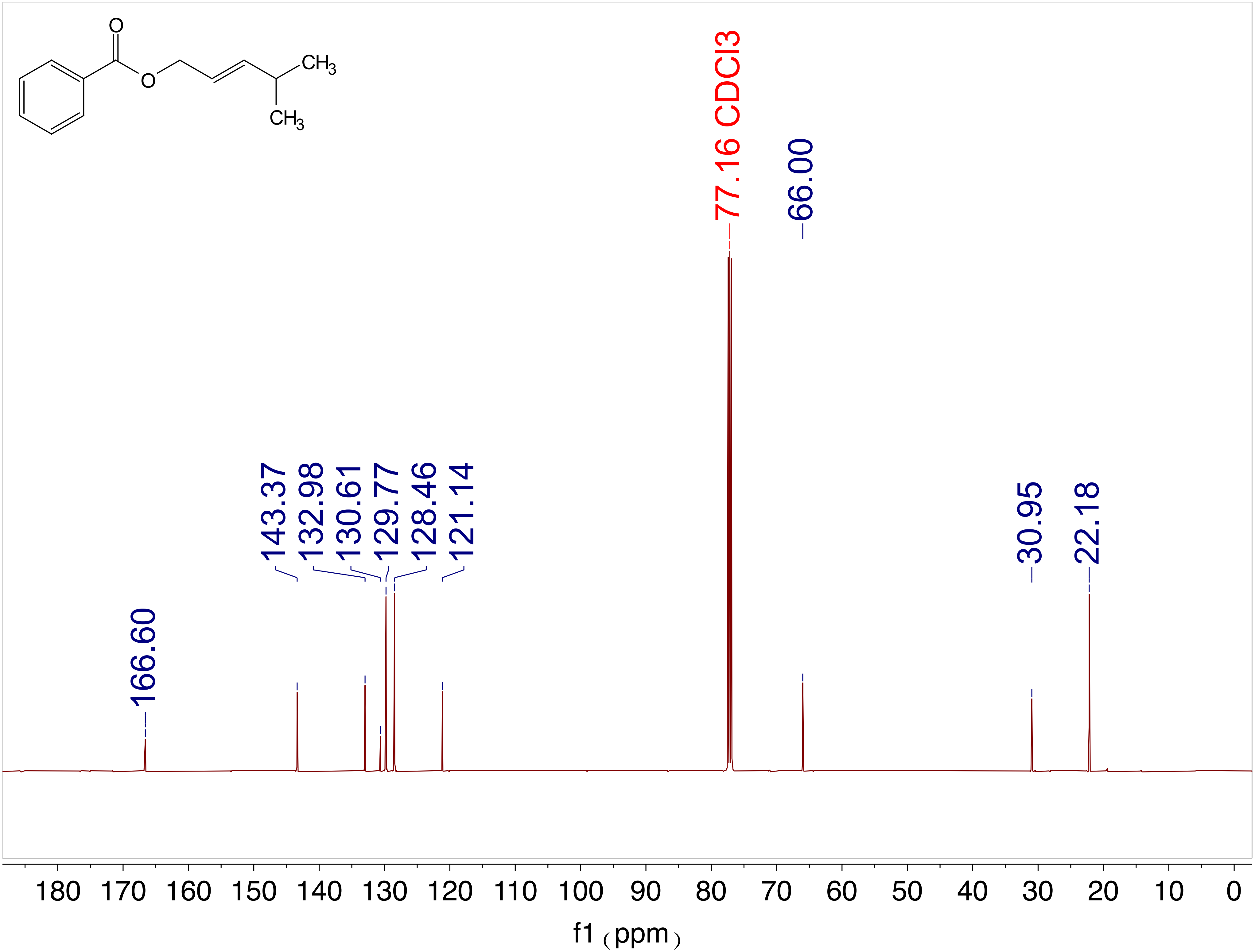

O
O
CH3
CH3
77.16 CDCl3
66.00
166.60
143.37
132.98
130.61
129.77
128.46
121.14
30.95
22.18
180
170
160
150
140
130
120
110
100
90
80
70
60
50
40
30
20
10
0
f1（ppm）

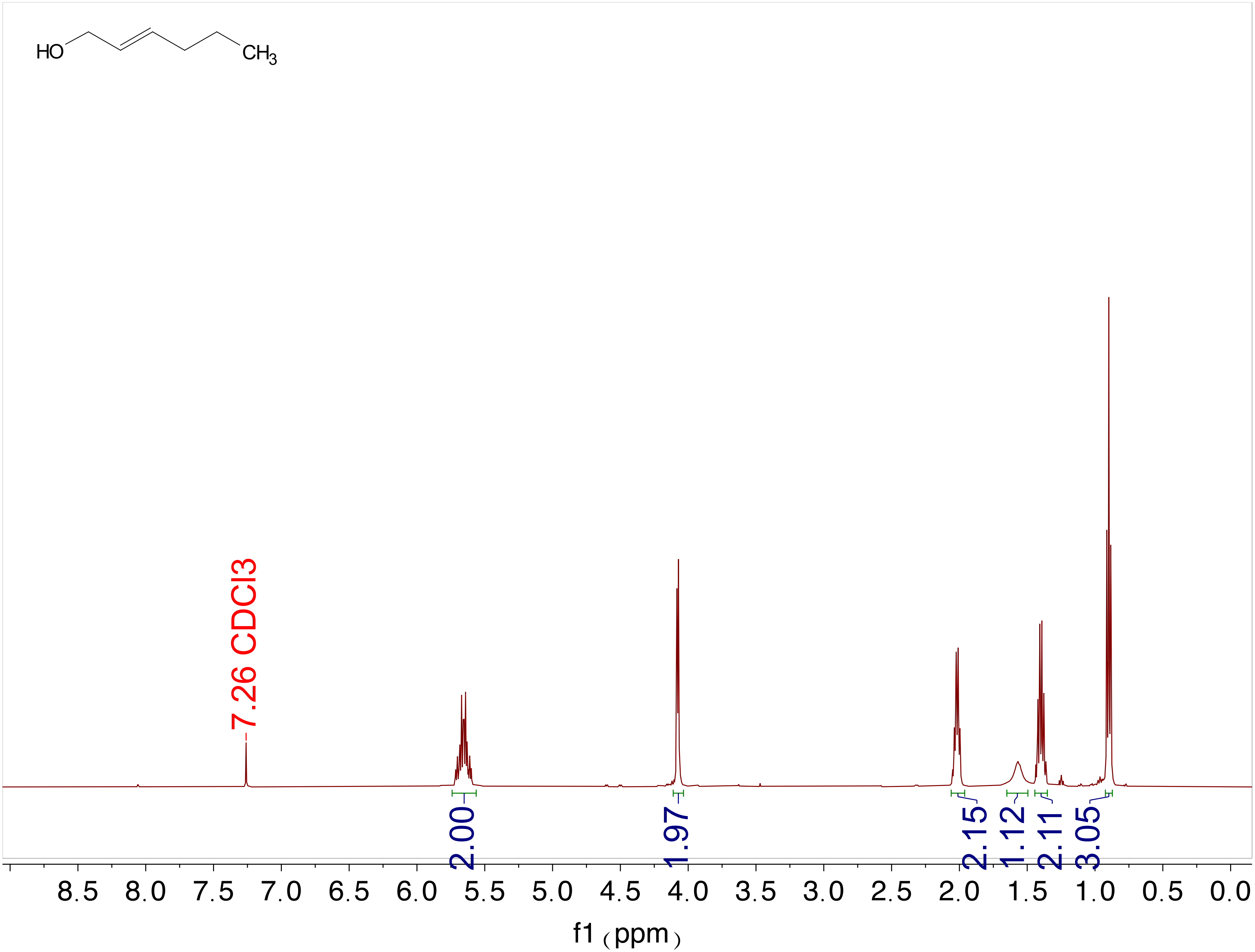
HO
CH3
7.26 CDCl3
2.00
1.97
2.15
1.12
2.11
3.05
8.5
8.0
7.5
7.0
6.5
6.0
5.5
5.0
4.5
4.0
3.5
3.0
2.5
2.0
1.5
1.0
0.5
0.0
f1（ppm）

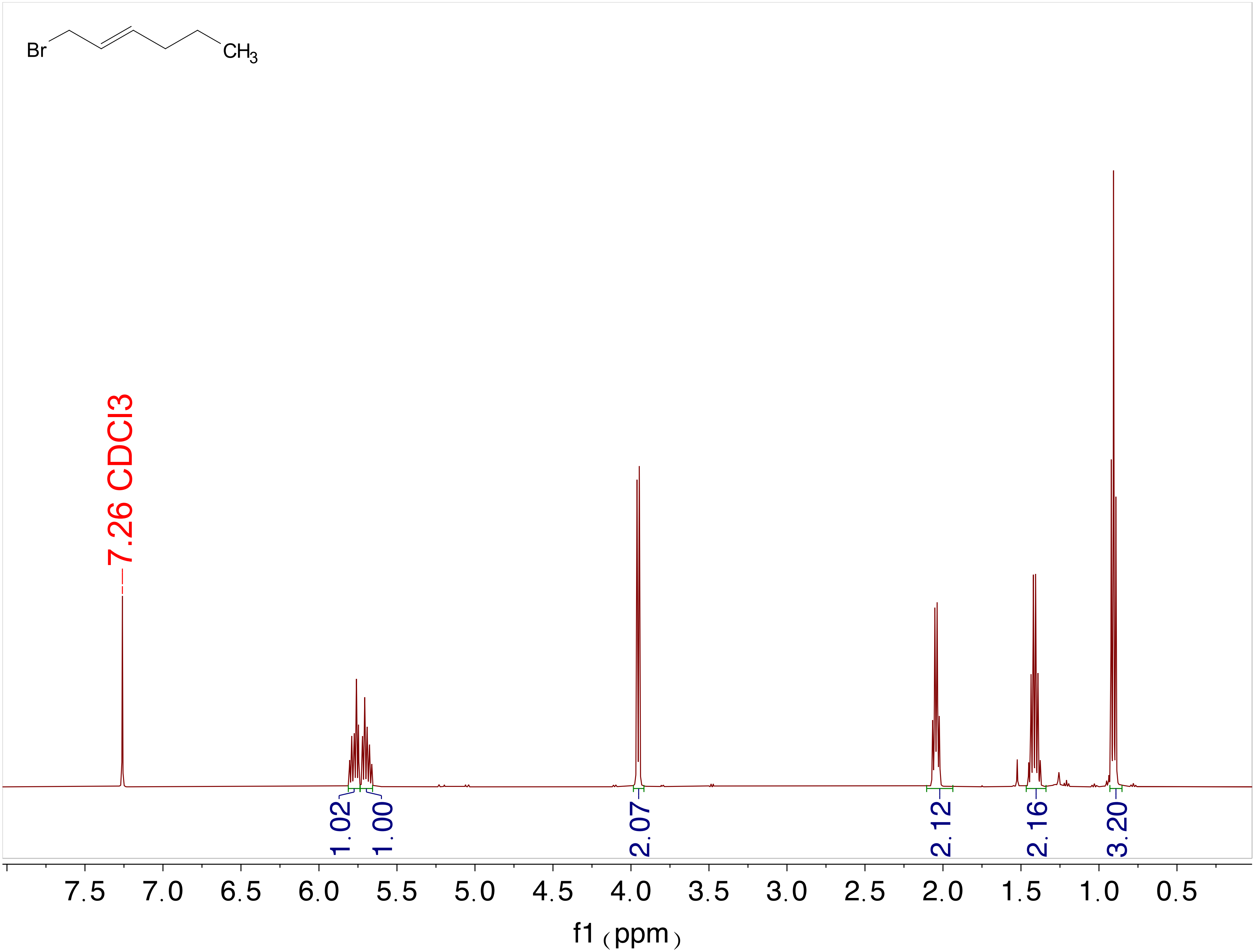

Br
CH3
7.26 CDCl3
1.02
1.00
2.07
2.12
2.16
3.20
7.5 7.0 6.5 6.0 5.5 5.0 4.5 4.0 3.5 3.0 2.5 2.0 1.5 1.0 0.5
f1 (ppm)

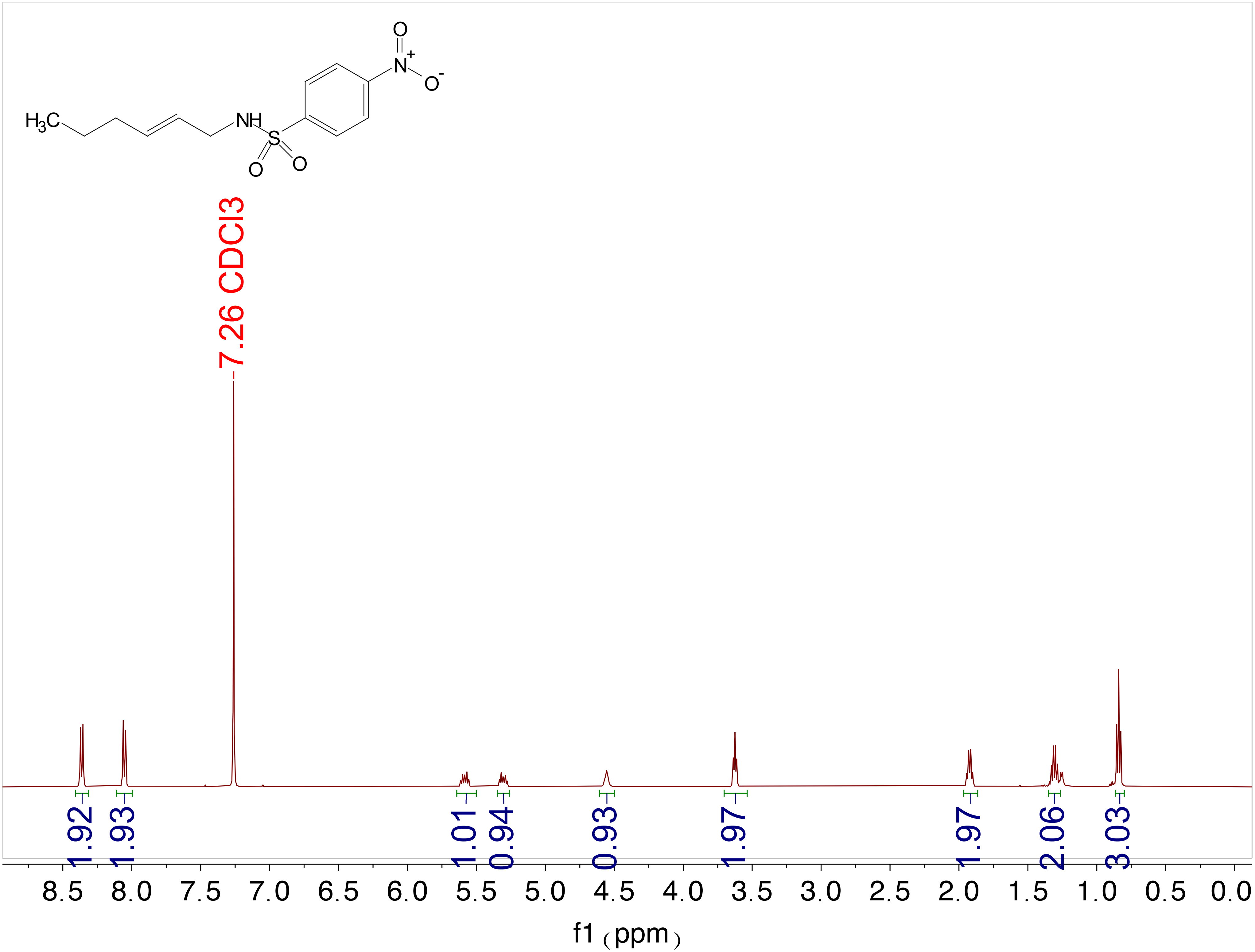

H3C
NH
S
O
O
N+
O
O-
7.26 CDCl3
1.92
1.93
1.01
0.94
0.93
1.97
1.97
2.06
3.03
8.5 8.0 7.5 7.0 6.5 6.0 5.5 5.0 4.5 4.0 3.5 3.0 2.5 2.0 1.5 1.0 0.5 0.0
f1 (ppm)

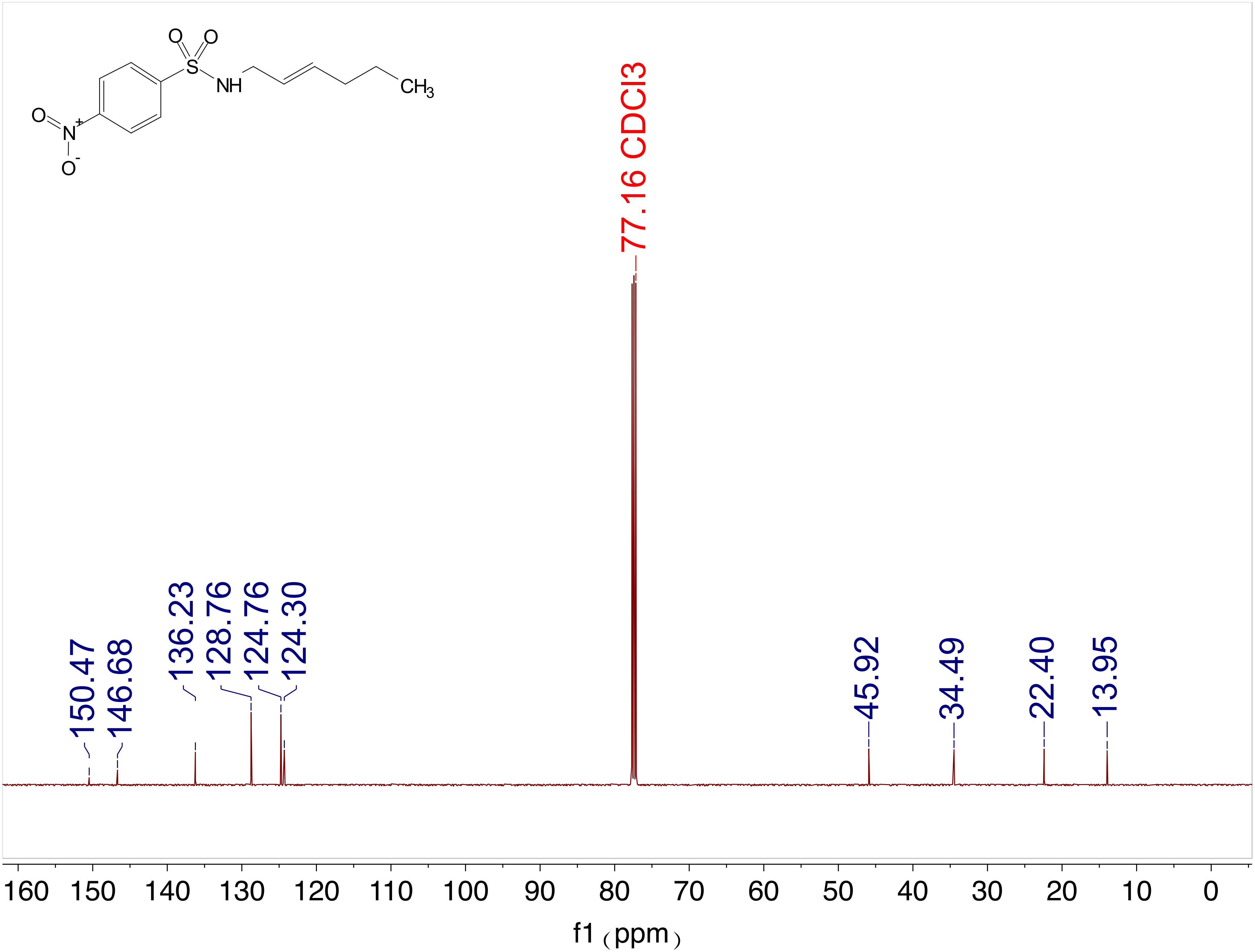
O
O
S
NH
CH3
O
N+
O-
150.47
146.68
136.23
128.76
124.76
124.30
77.16 CDCl3
45.92
34.49
22.40
13.95
160
150
140
130
120
110
100
90
80
70
60
50
40
30
20
10
0
f1 (ppm)

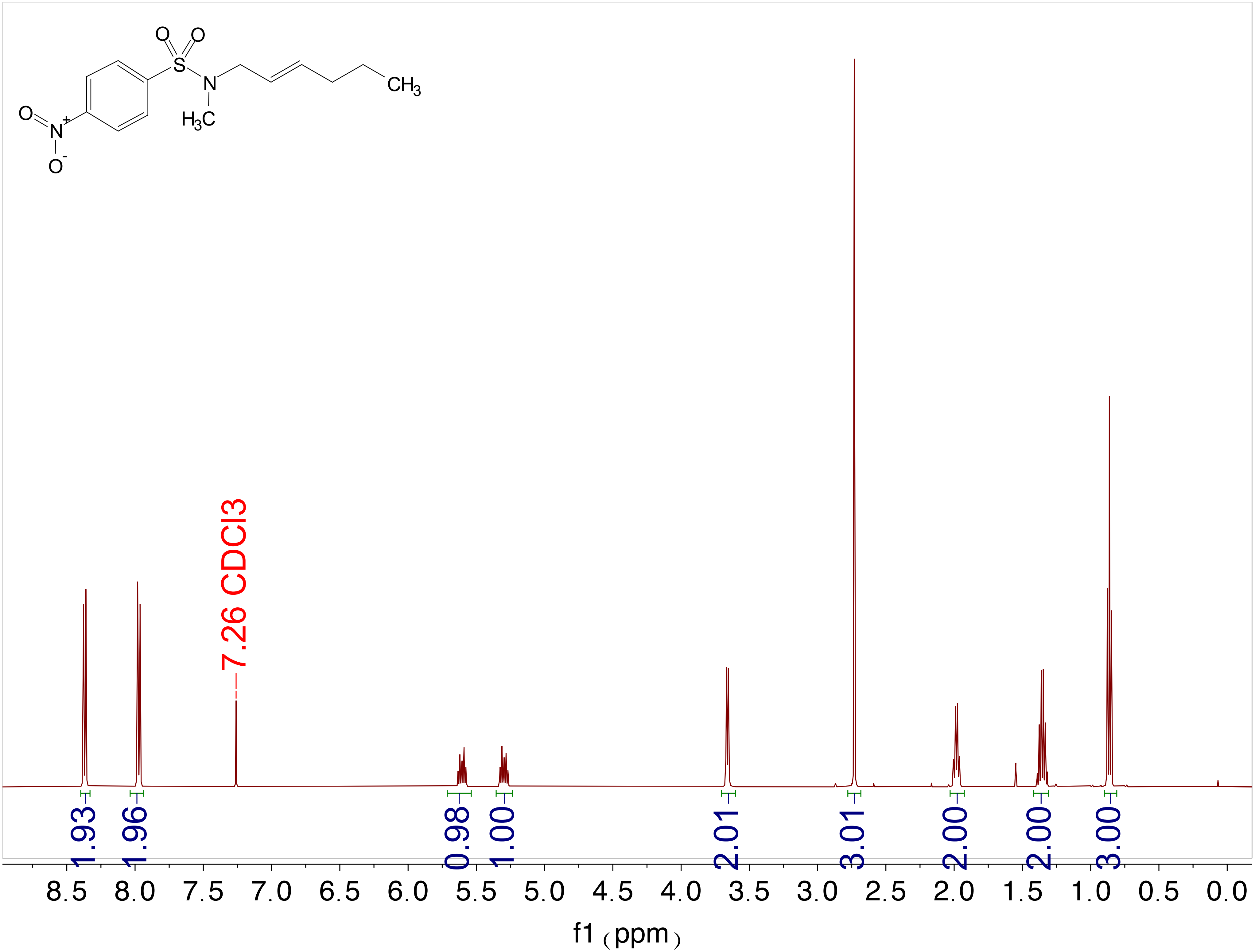

CH3
H3C
7.26 CDCl3
1.93
1.96
0.98
1.00
2.01
3.01
2.00
2.00
3.00
8.5 8.0 7.5 7.0 6.5 6.0 5.5 5.0 4.5 4.0 3.5 3.0 2.5 2.0 1.5 1.0 0.5 0.0
f1（ppm）

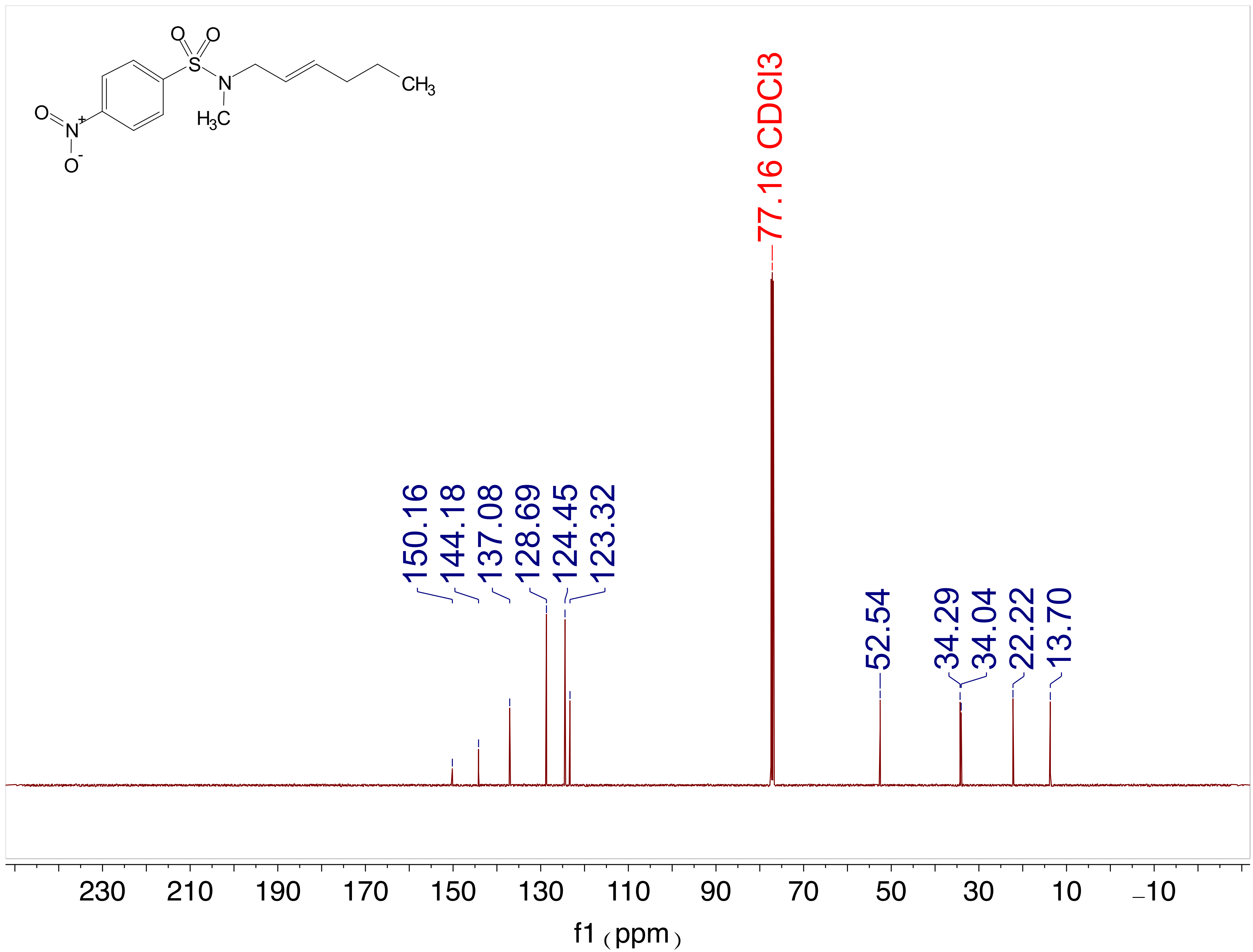

CH3
H3C
77.16 CDCl3
150.16
144.18
137.08
128.69
124.45
123.32
52.54
34.29
34.04
22.22
13.70
230
210
190
170
150
130
110
90
70
50
30
10
–10
f1 (ppm)

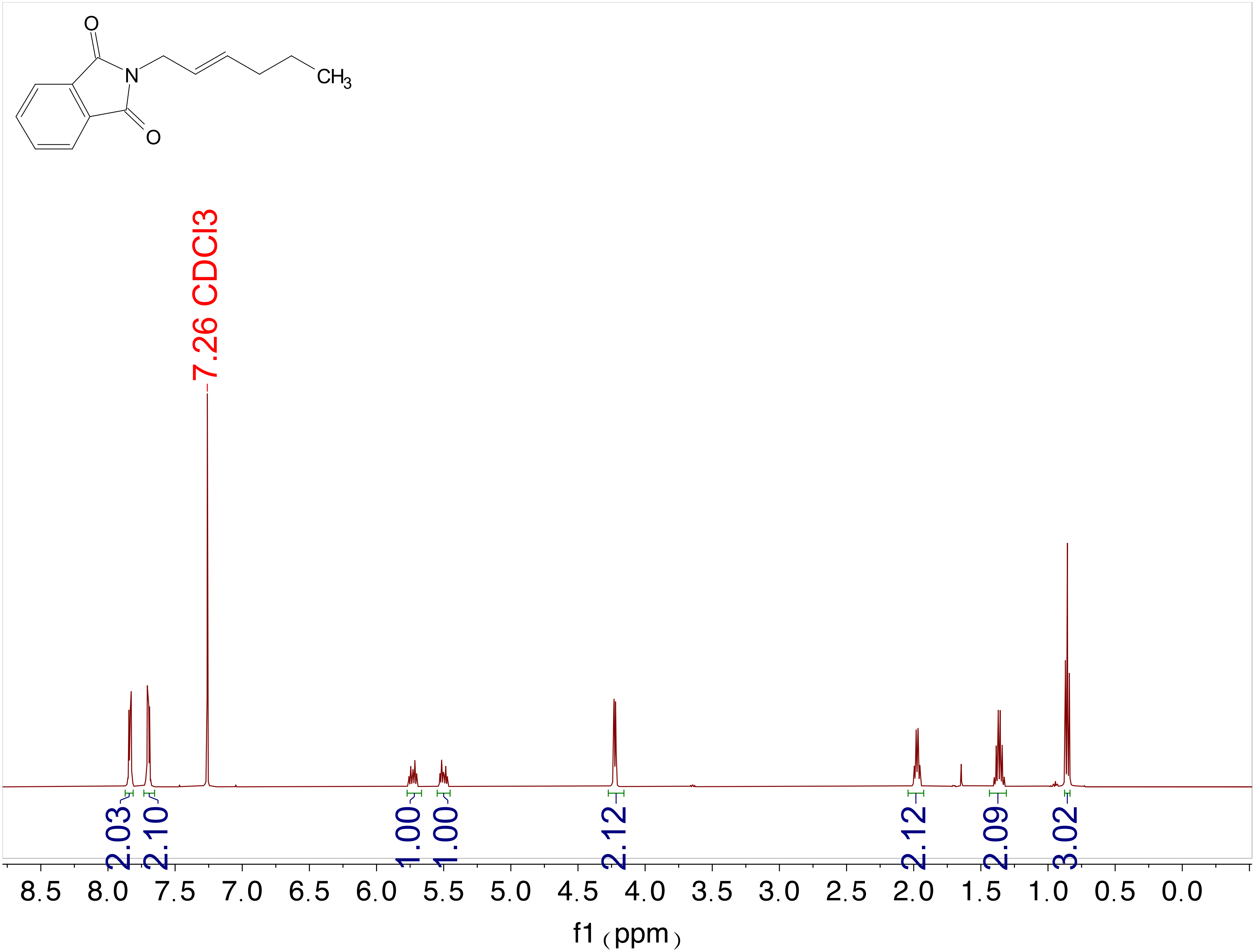

O
N
CH3
O
7.26 CDCl3
2.03
2.10
1.00
1.00
2.12
2.12
2.09
3.02
8.5 8.0 7.5 7.0 6.5 6.0 5.5 5.0 4.5 4.0 3.5 3.0 2.5 2.0 1.5 1.0 0.5 0.0
f1 (ppm)

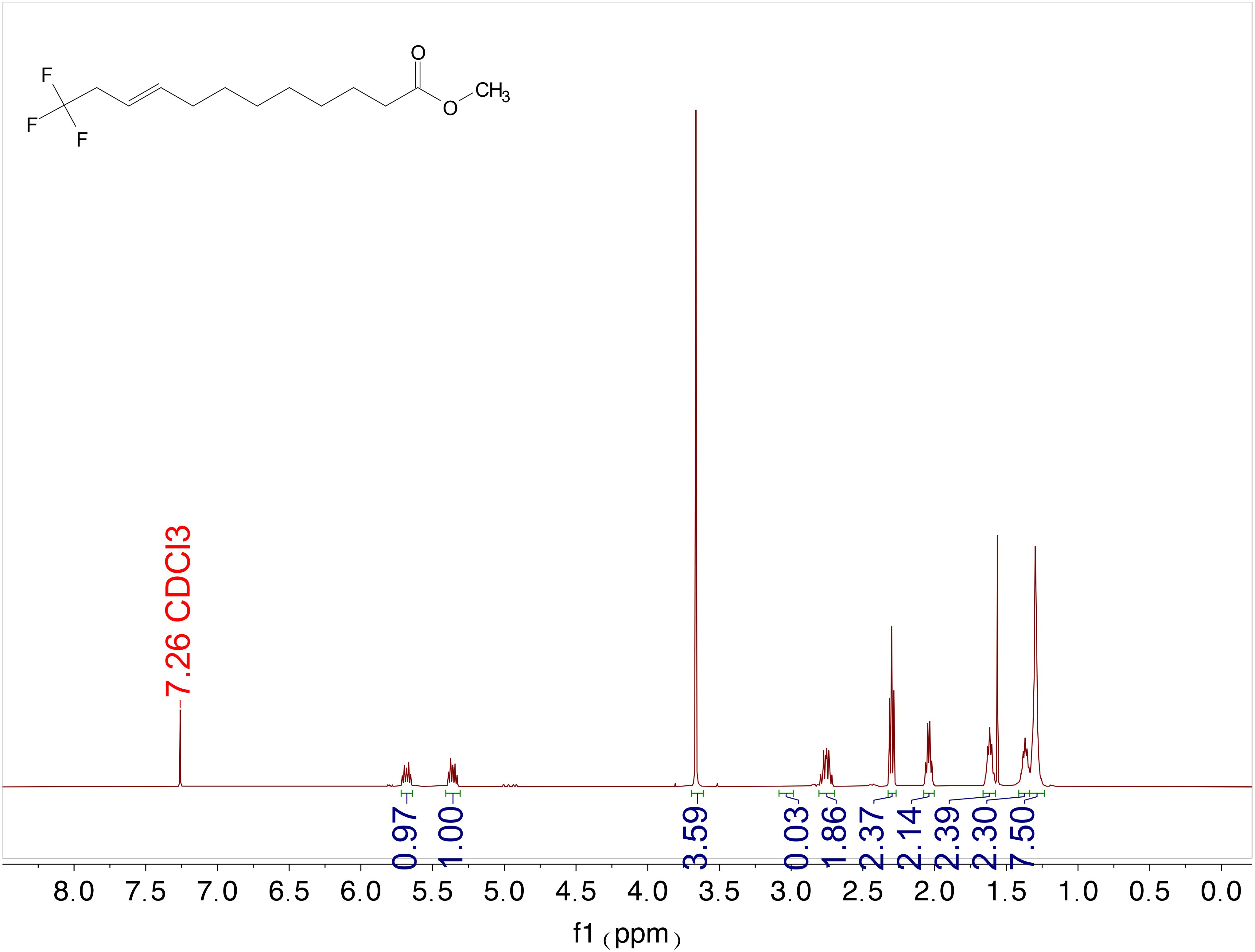

F
F
F
O
O
CH3
7.26 CDCl3
0.97
1.00
3.59
0.03
1.86
2.37
2.14
2.39
2.30
7.50
8.0 7.5 7.0 6.5 6.0 5.5 5.0 4.5 4.0 3.5 3.0 2.5 2.0 1.5 1.0 0.5 0.0
f1 (ppm)

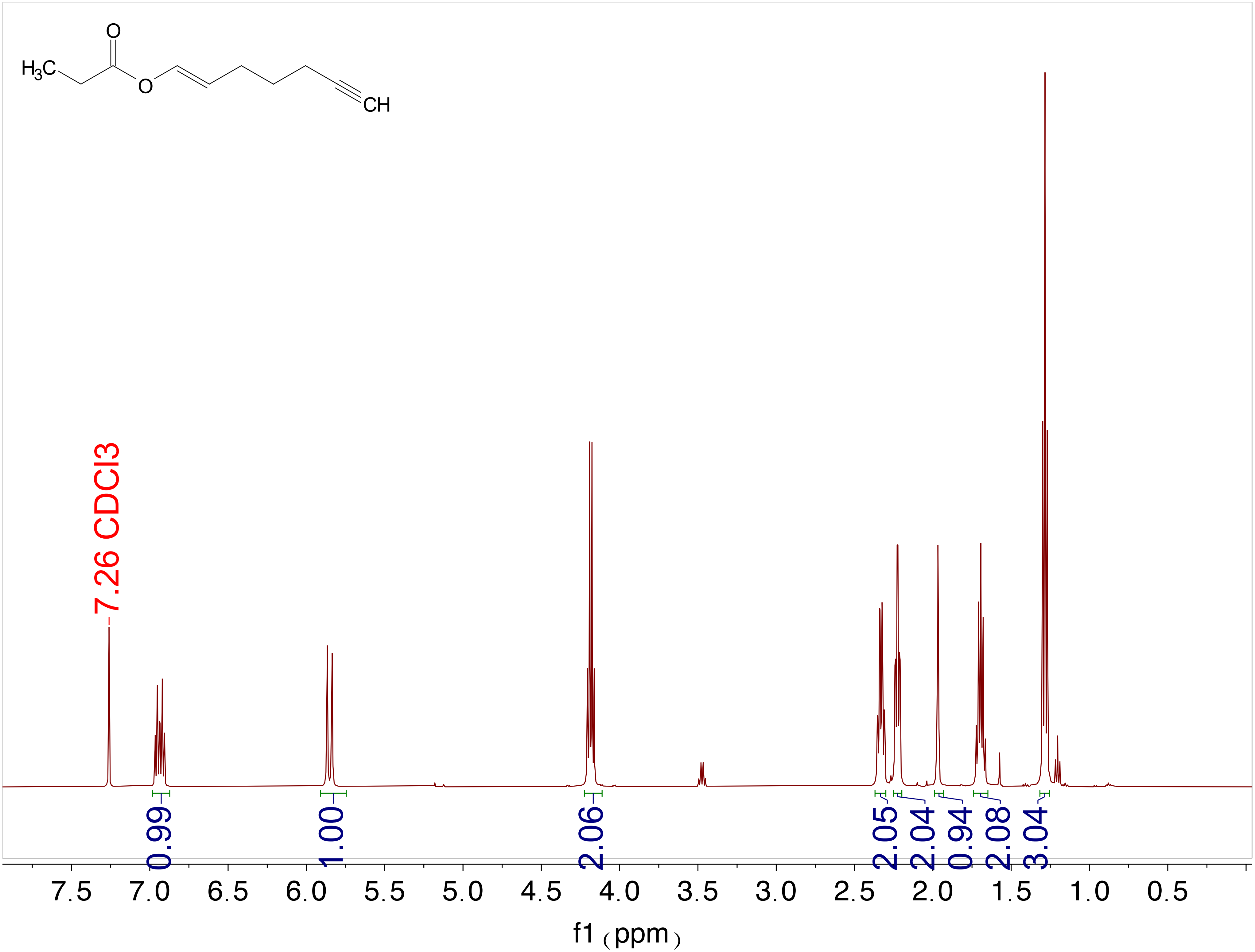

O
H3C
O
CH
7.26 CDCl3
0.99
1.00
2.06
2.05
2.04
0.94
2.08
3.04
7.5
7.0
6.5
6.0
5.5
5.0
4.5
4.0
3.5
3.0
2.5
2.0
1.5
1.0
0.5
f1（ppm）

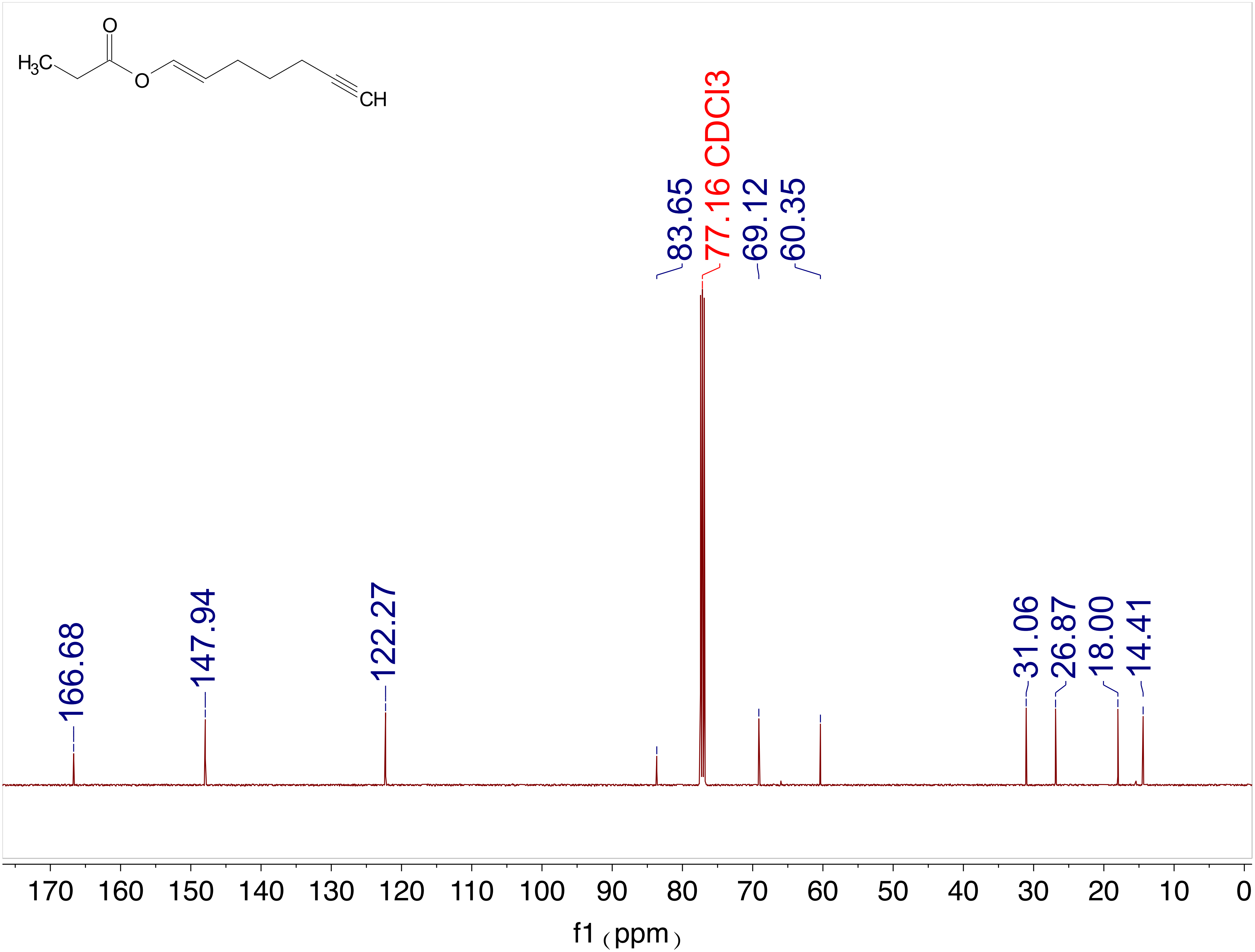

H3C
O
O
CH
166.68
147.94
122.27
83.65
77.16 CDCl3
69.12
60.35
31.06
26.87
18.00
14.41
170 160 150 140 130 120 110 100 90 80 70 60 50 40 30 20 10 0
f1 (ppm)

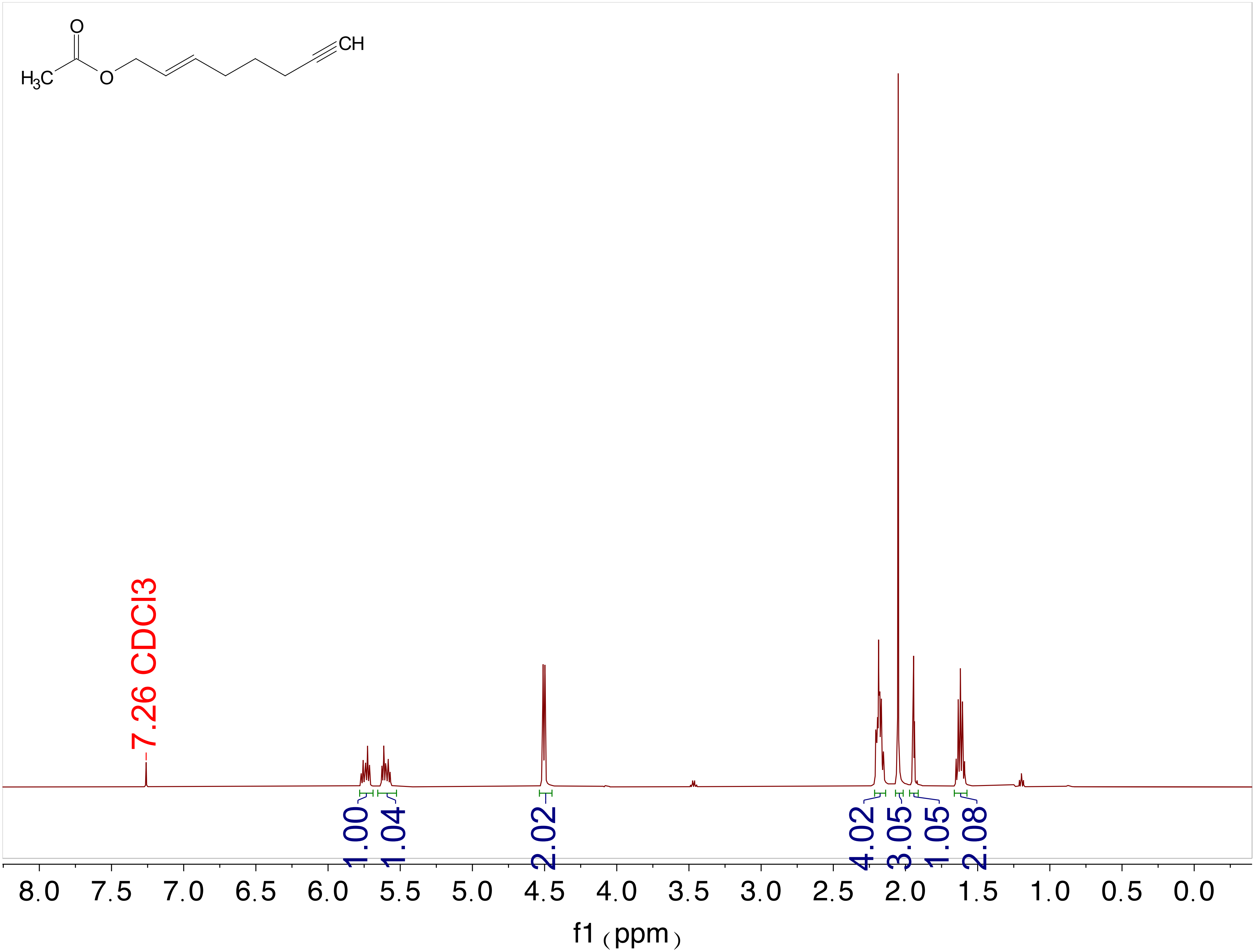

O
H3C
O
CH
7.26 CDCl3
1.00
1.04
2.02
4.02
3.05
1.05
2.08
8.0
7.5
7.0
6.5
6.0
5.5
5.0
4.5
4.0
3.5
3.0
2.5
2.0
1.5
1.0
0.5
0.0
f1 (ppm)

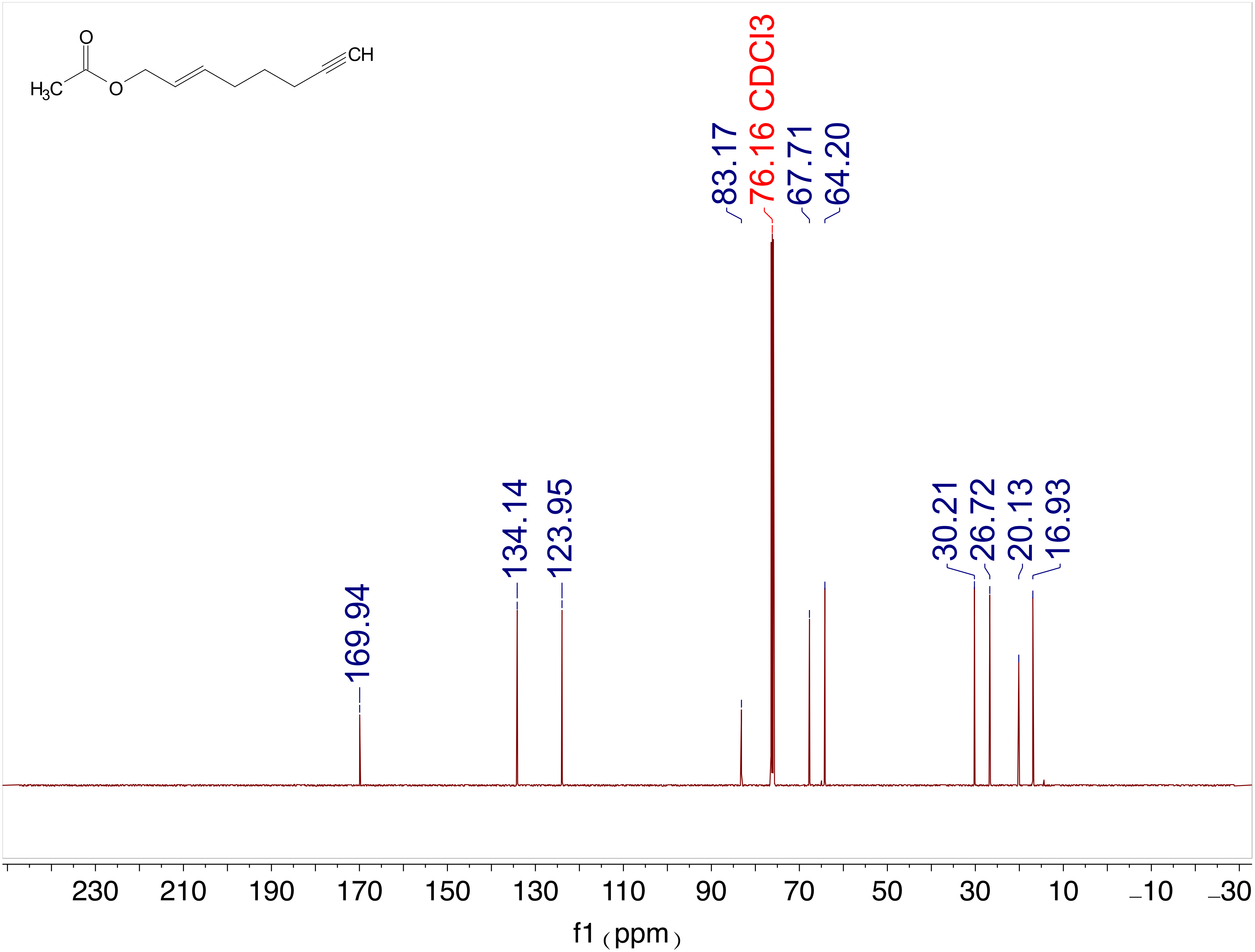

O
H_3C
O
CH
83.17
76.16 CDCl3
67.71
64.20
169.94
134.14
123.95
30.21
26.72
20.13
16.93
230
210
190
170
150
130
110
90
70
50
30
10
–10
–30
f1 (ppm)

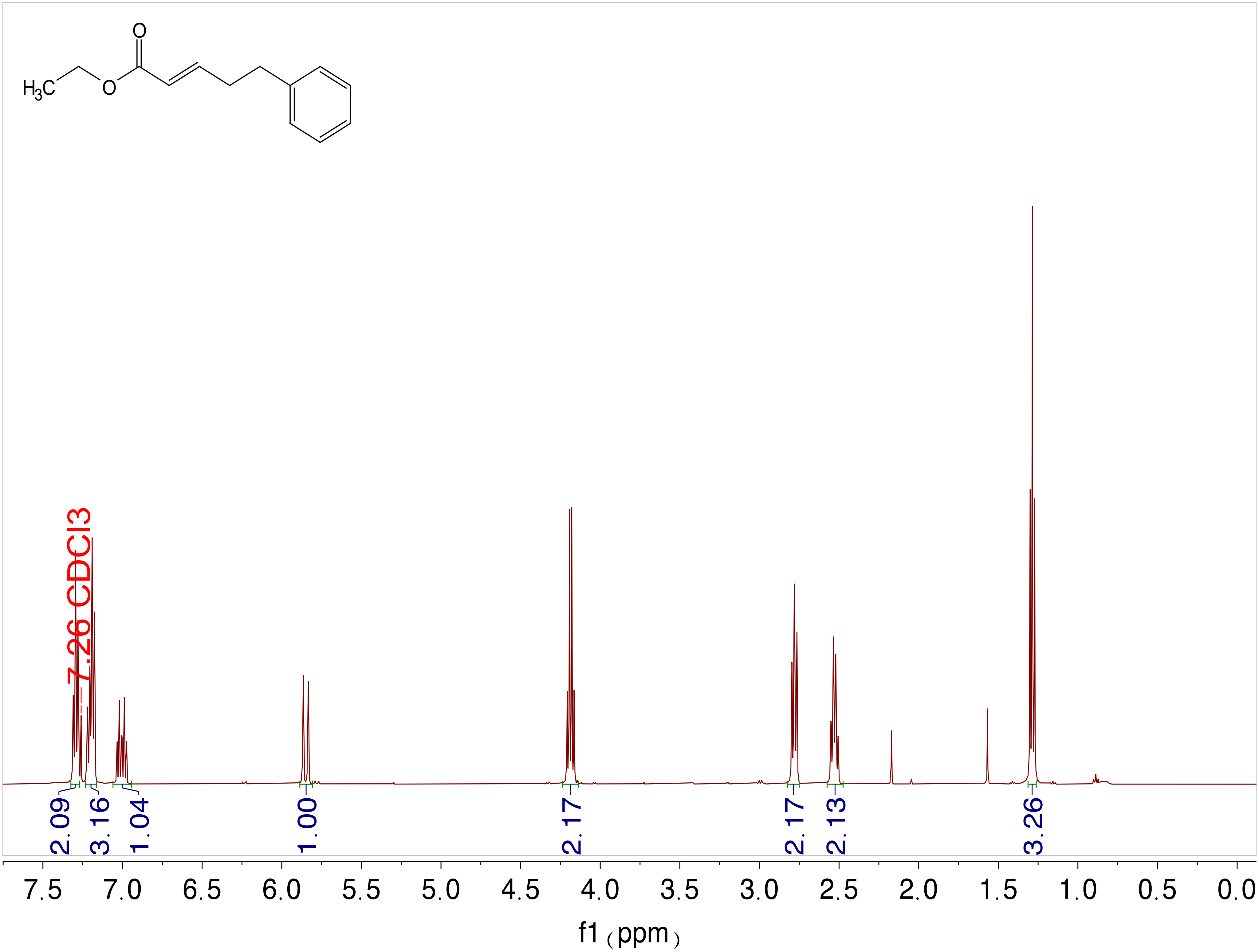

O
O
H_3C
7.26 CDCl3
2.09
3.16
1.04
1.00
2.17
2.17
2.13
3.26
7.5
7.0
6.5
6.0
5.5
5.0
4.5
4.0
3.5
3.0
2.5
2.0
1.5
1.0
0.5
0.0
f1（ppm）

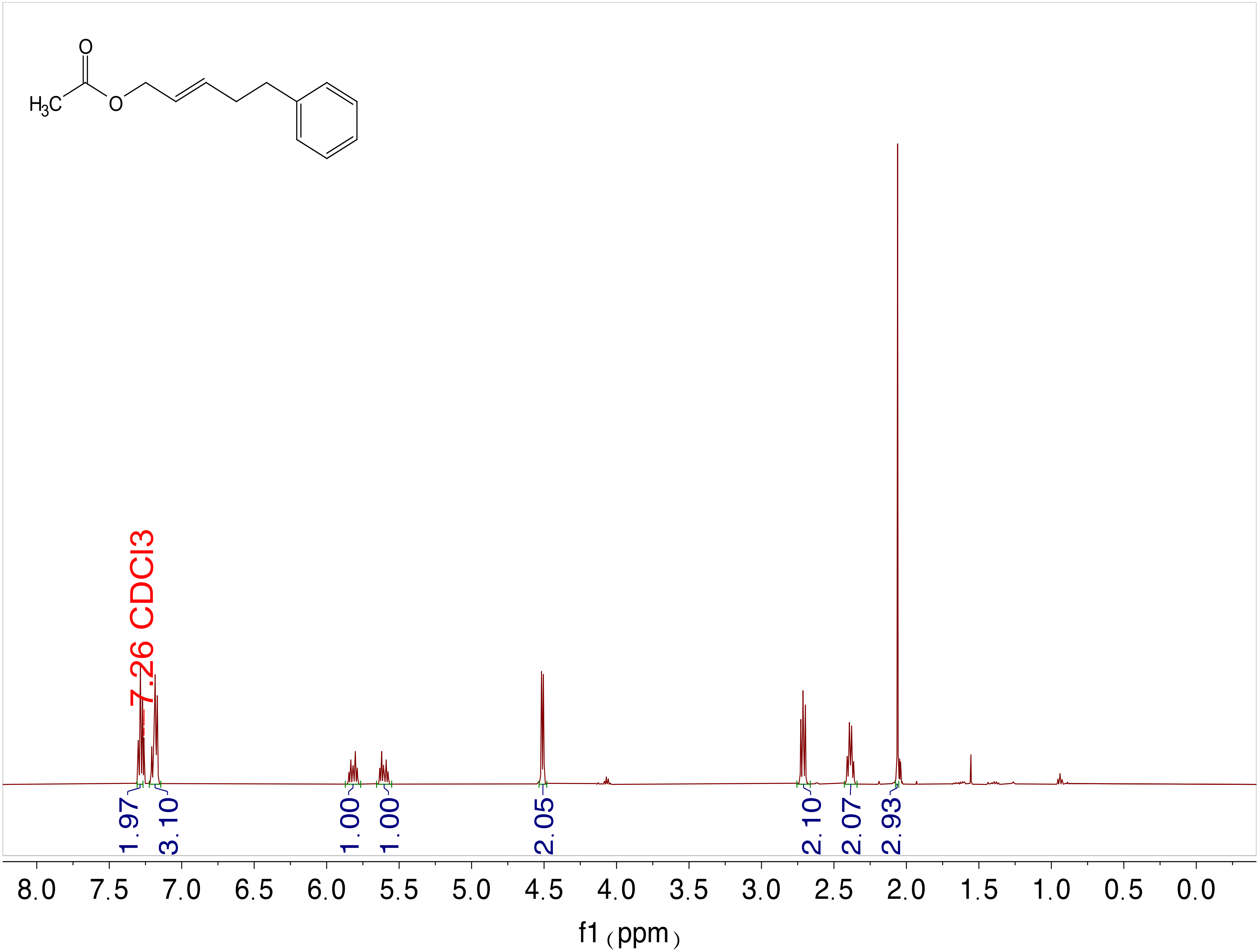

O
H3C
O
7.26 CDCl3
1.97
3.10
1.00
1.00
2.05
2.10
2.07
2.93
8.0
7.5
7.0
6.5
6.0
5.5
5.0
4.5
4.0
3.5
3.0
2.5
2.0
1.5
1.0
0.5
0.0
f1 (ppm)

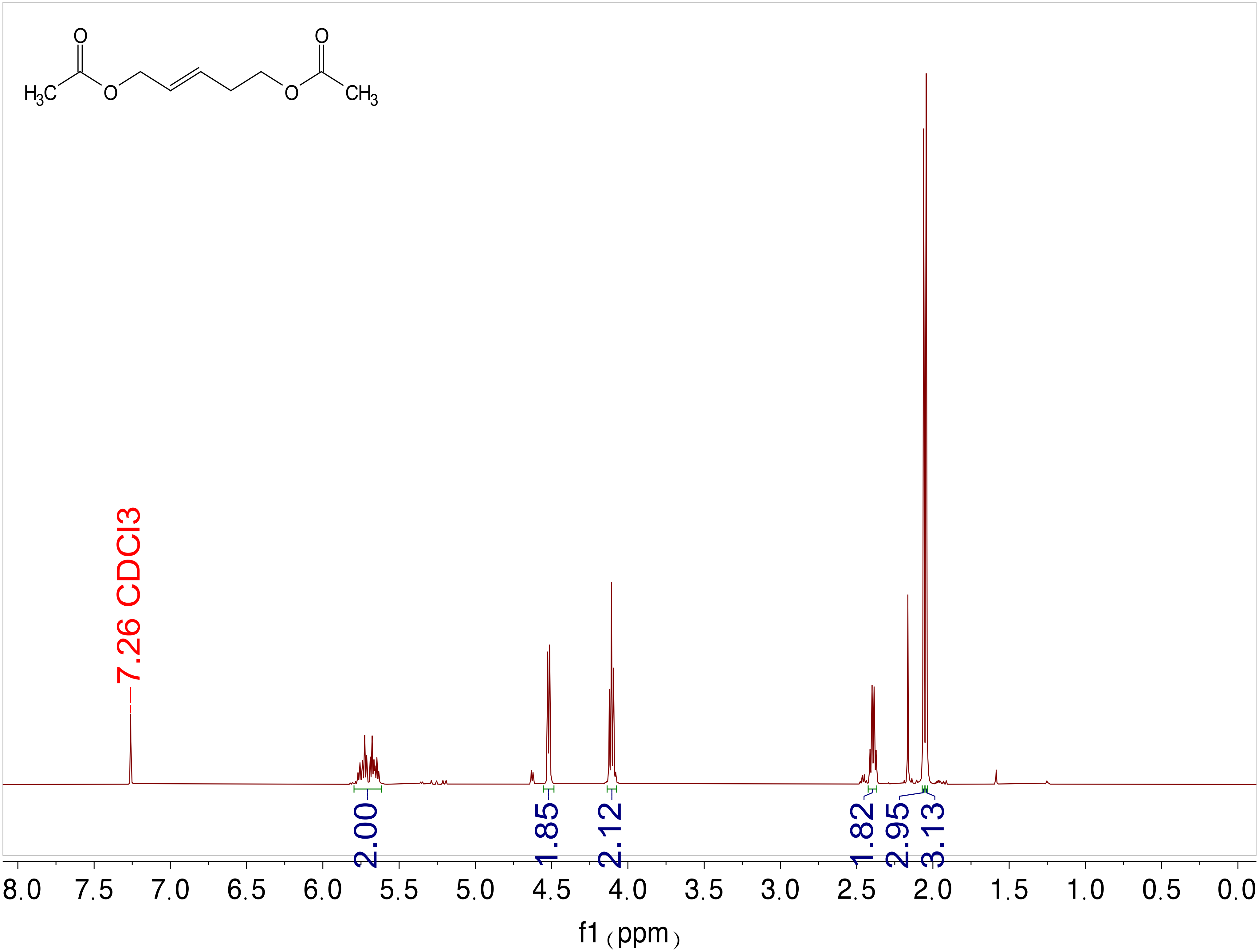

7.26 CDCl3
2.00
1.85
2.12
1.82
2.95
3.13
8.0 7.5 7.0 6.5 6.0 5.5 5.0 4.5 4.0 3.5 3.0 2.5 2.0 1.5 1.0 0.5 0.0
f1 (ppm)

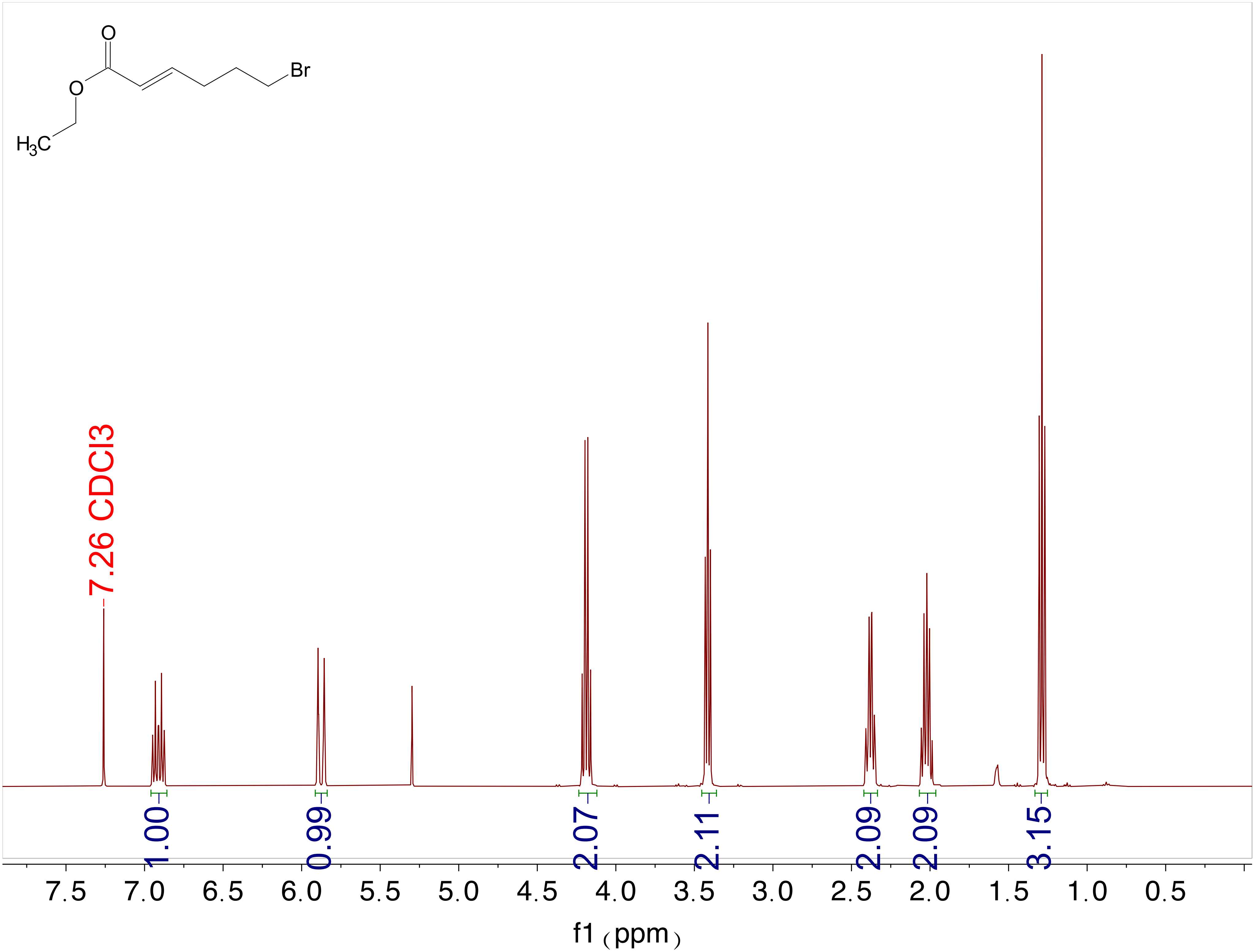

O
O
Br
H3C
7.26 CDCl3
1.00
0.99
2.07
2.11
2.09
2.09
3.15
7.5
7.0
6.5
6.0
5.5
5.0
4.5
4.0
3.5
3.0
2.5
2.0
1.5
1.0
0.5
f1 (ppm)

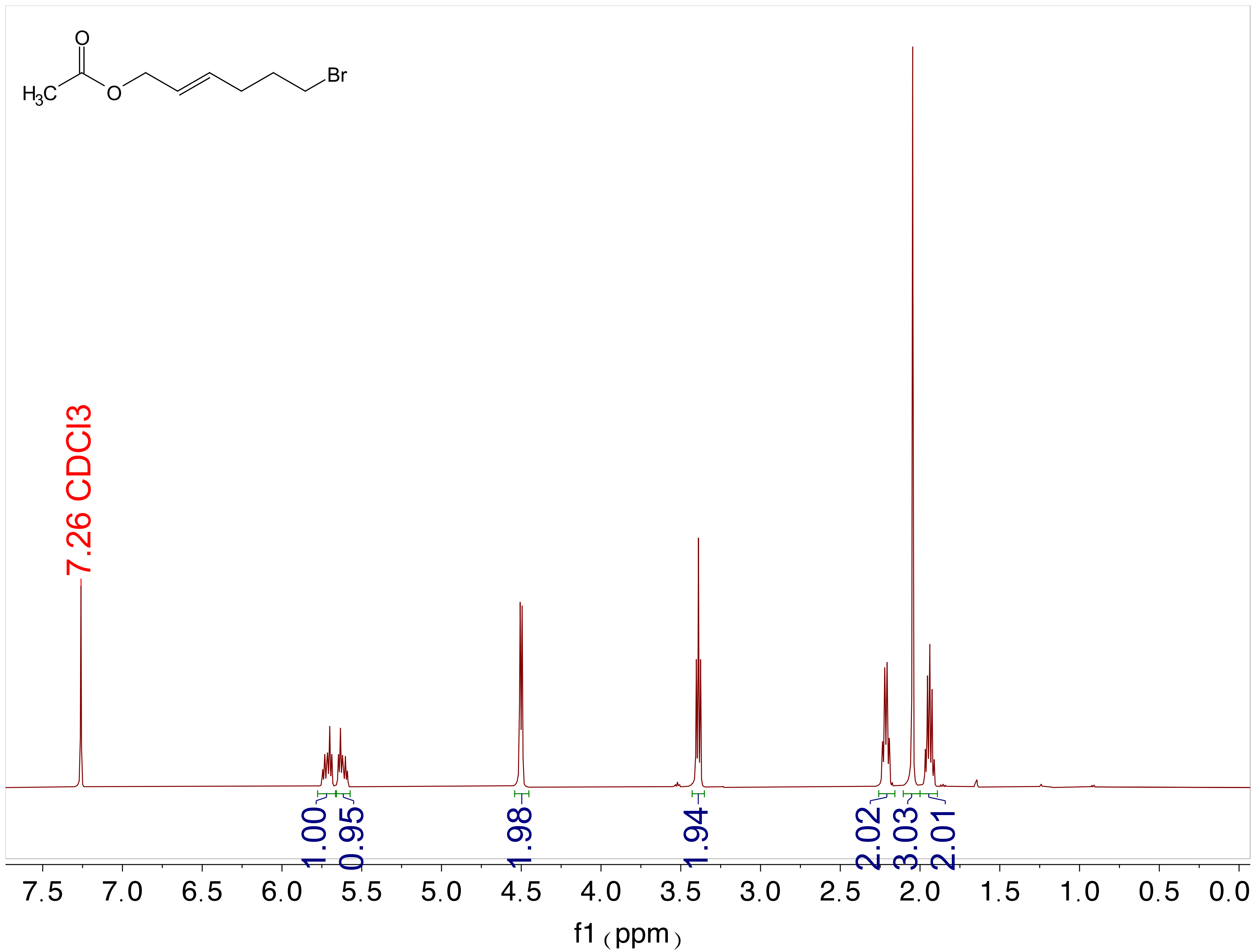
O
H_3C
O
Br
7.26 CDCl3
1.00
0.95
1.98
1.94
2.02
3.03
2.01
7.5
7.0
6.5
6.0
5.5
5.0
4.5
4.0
3.5
3.0
2.5
2.0
1.5
1.0
0.5
0.0
f1 (ppm)

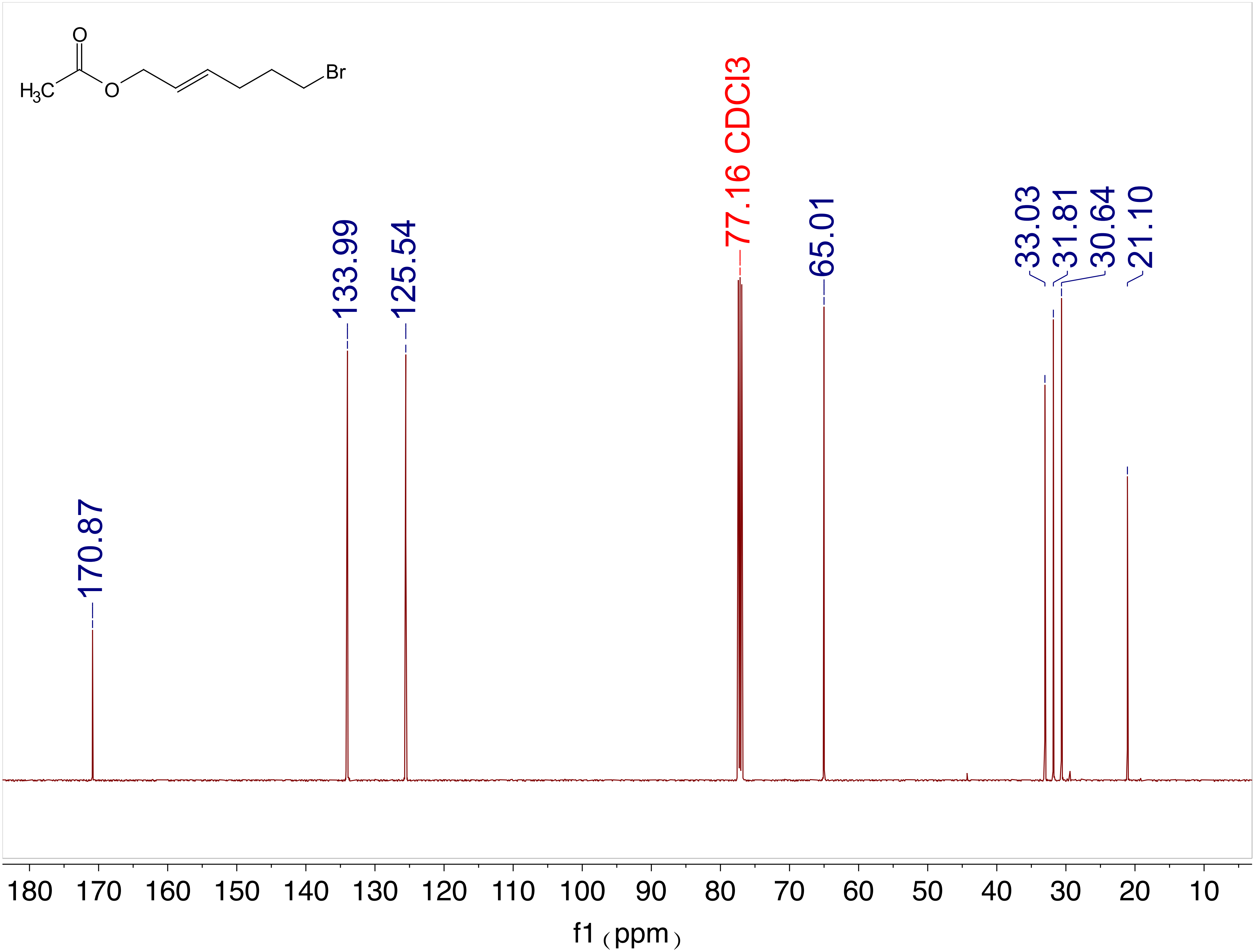

O
H3C
O
Br
170.87
133.99
125.54
77.16 CDCl3
65.01
33.03
31.81
30.64
21.10
180 170 160 150 140 130 120 110 100 90 80 70 60 50 40 30 20 10
f1 (ppm)

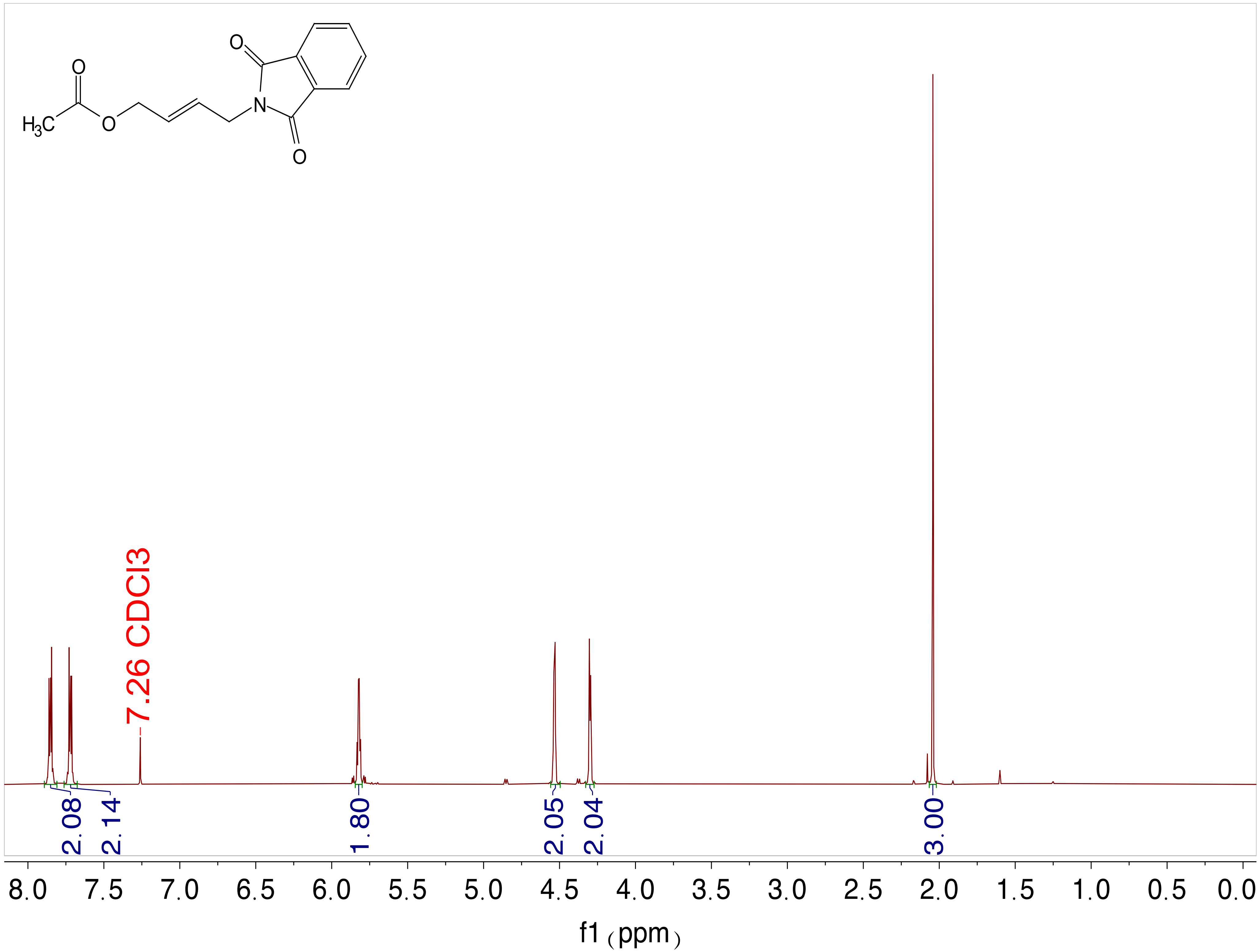

O
O
O
N
O
H_3C
7.26 CDCl3
2.08
2.14
1.80
2.05
2.04
3.00
8.0
7.5
7.0
6.5
6.0
5.5
5.0
4.5
4.0
3.5
3.0
2.5
2.0
1.5
1.0
0.5
0.0
f1 (ppm)

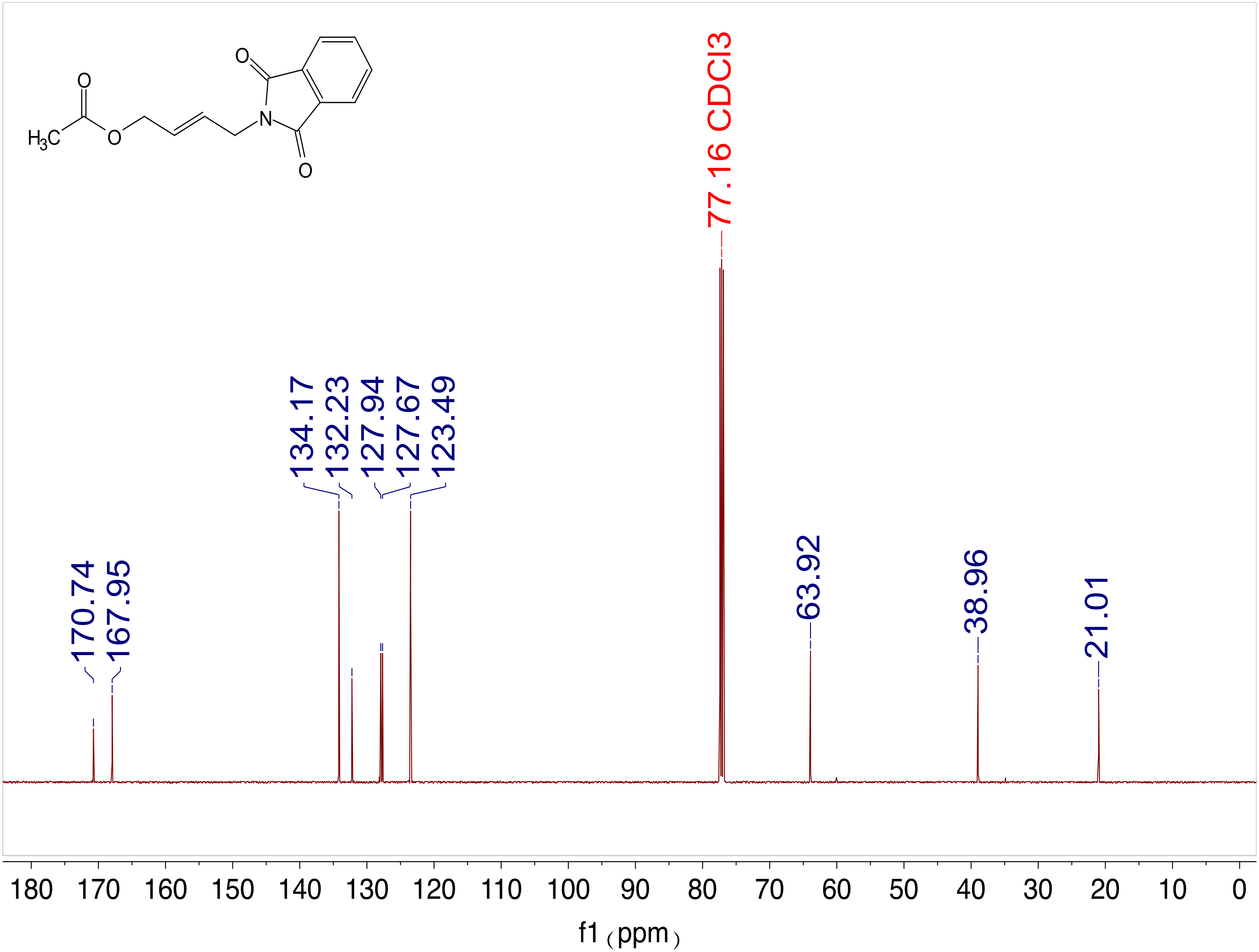

170.74
167.95
134.17
132.23
127.94
127.67
123.49
77.16 CDCl3
63.92
38.96
21.01
180 170 160 150 140 130 120 110 100 90 80 70 60 50 40 30 20 10 0
f1 (ppm)

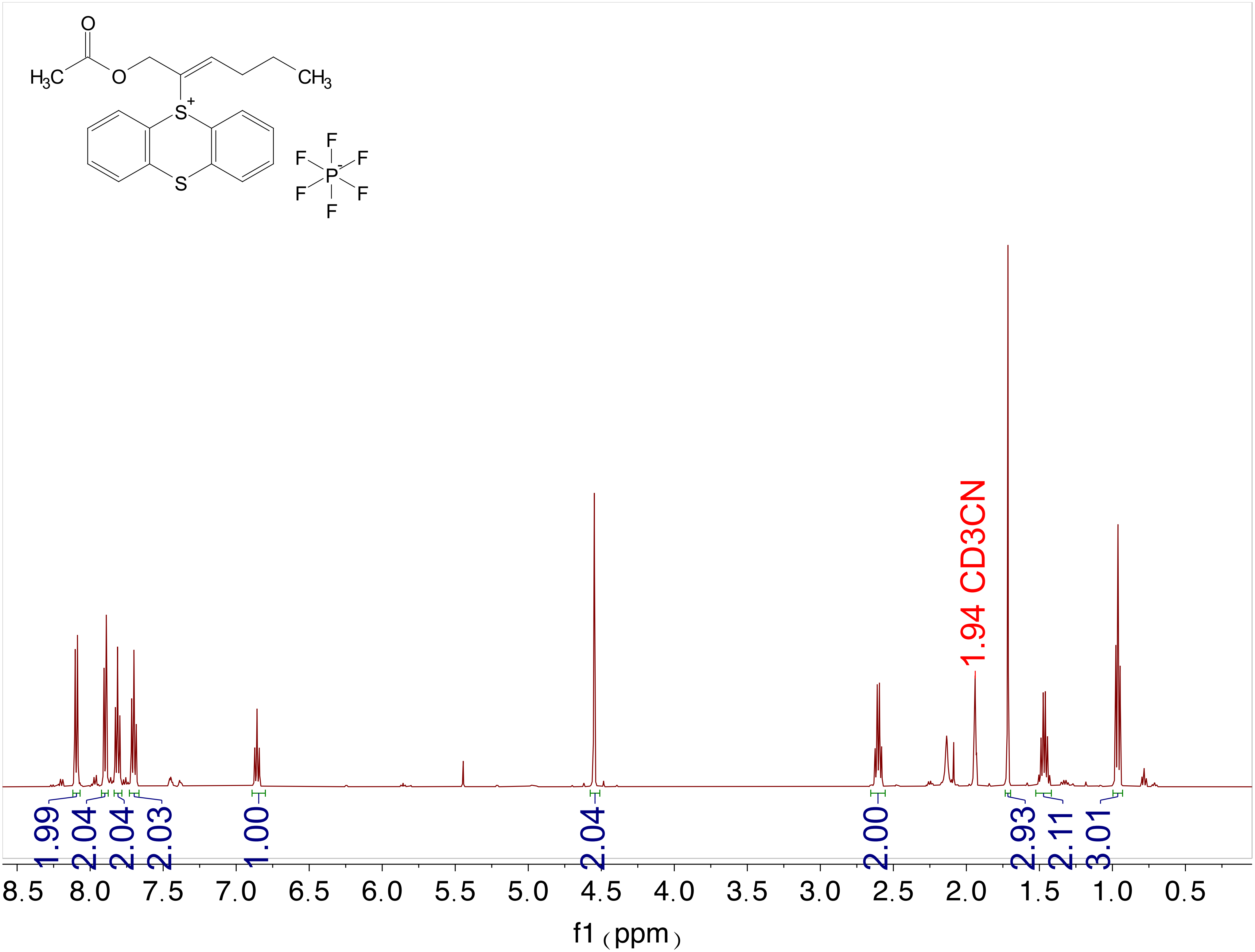

1.94 CD3CN
1.99
2.04
2.04
2.03
1.00
2.04
2.00
2.93
2.11
3.01
8.5
8.0
7.5
7.0
6.5
6.0
5.5
5.0
4.5
4.0
3.5
3.0
2.5
2.0
1.5
1.0
0.5
f1（ppm）

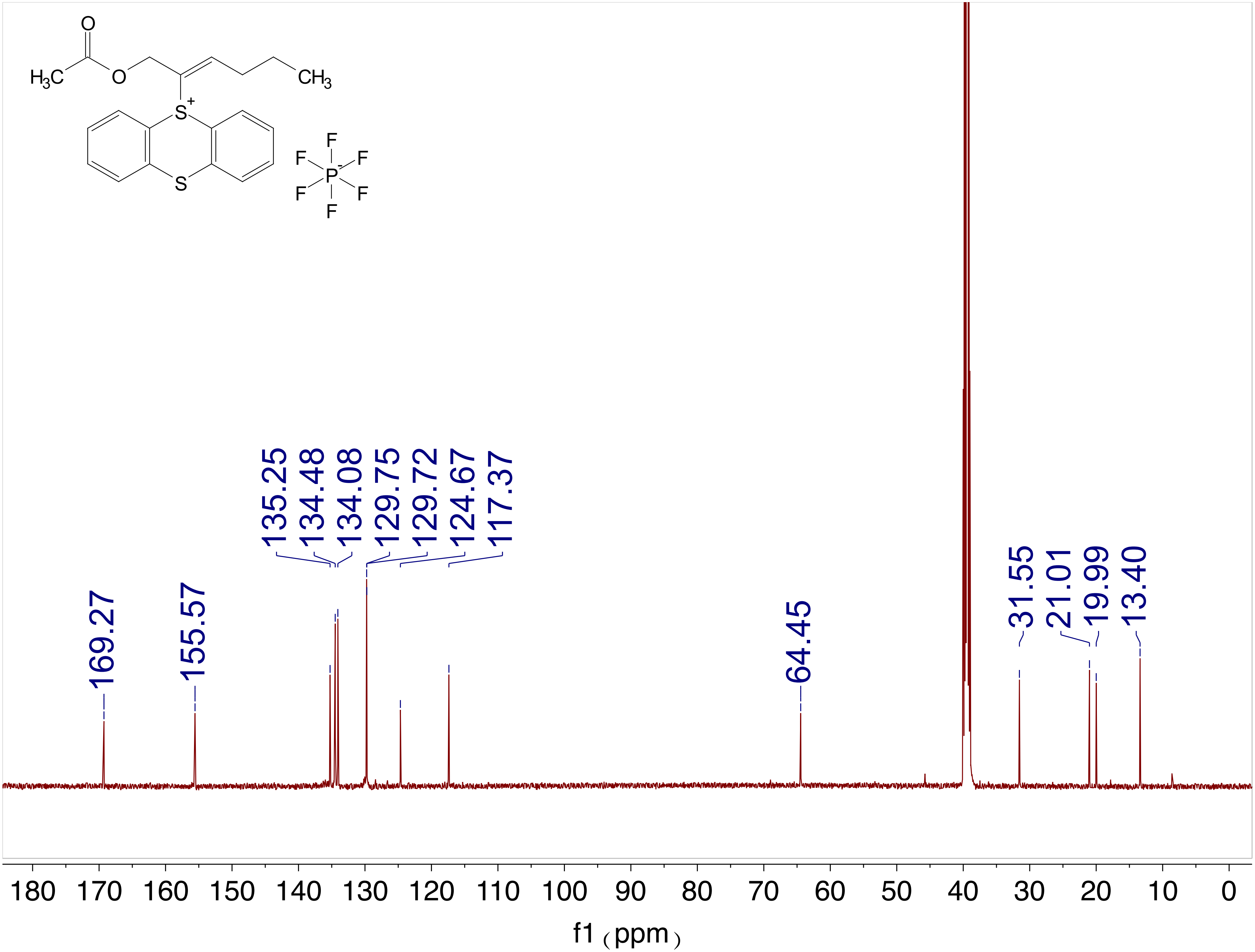
O
H3C
O
CH3
S+
S
F
F
F
P-
F
F
F
169.27
155.57
135.25
134.48
134.08
129.75
129.72
124.67
117.37
64.45
31.55
21.01
19.99
13.40
180
170
160
150
140
130
120
110
100
90
80
70
60
50
40
30
20
10
0
f1 (ppm)

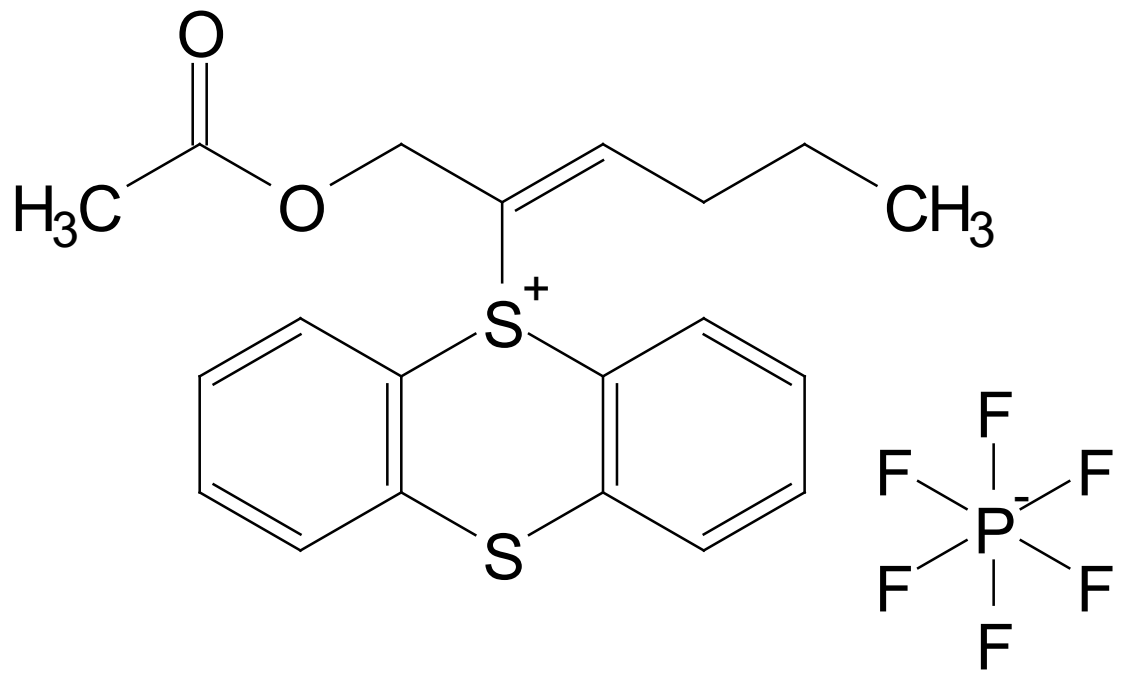

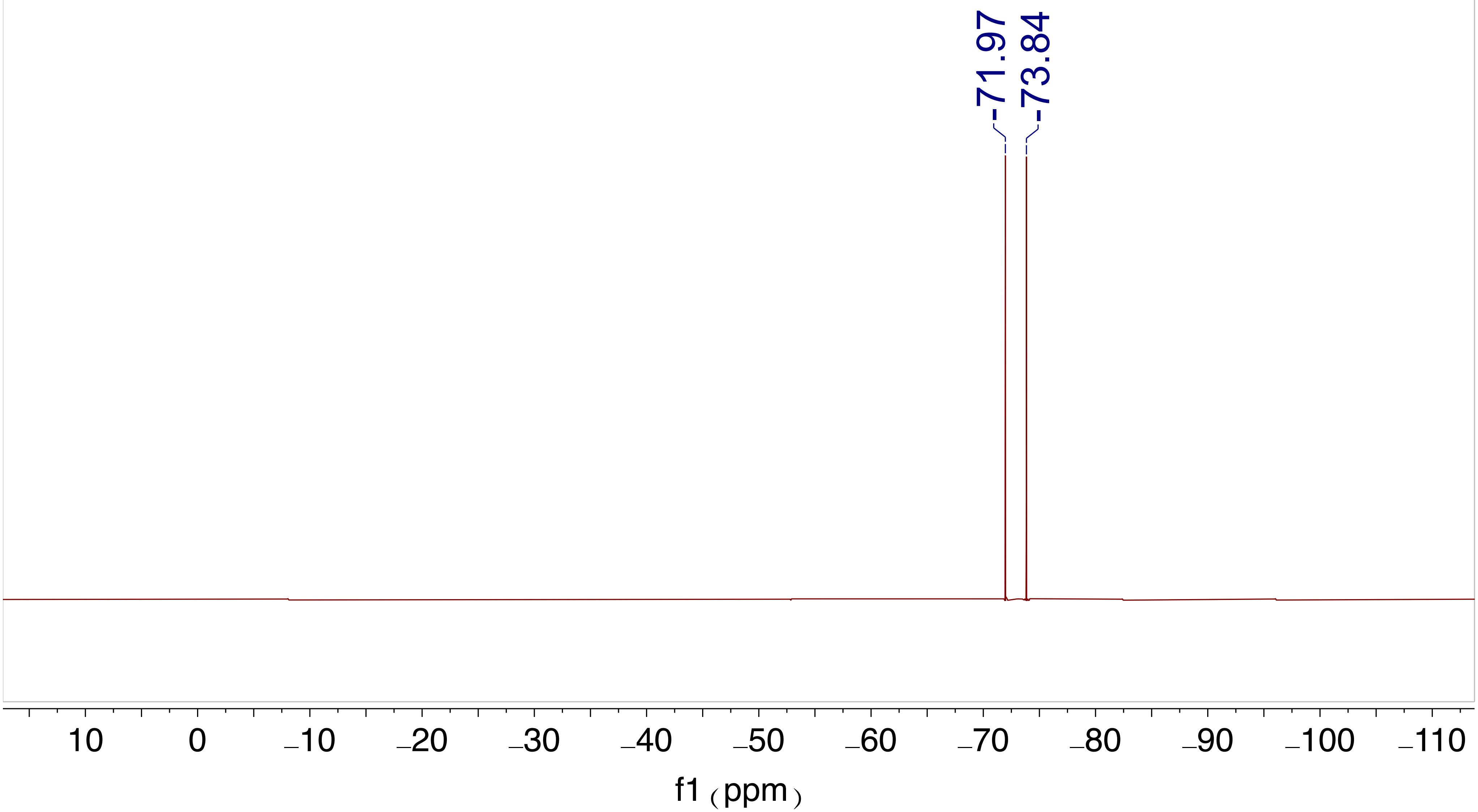

-71.97
-73.84
10
0
–10
–20
–30
–40
–50
–60
–70
–80
–90
–100
–110
f1 (ppm)

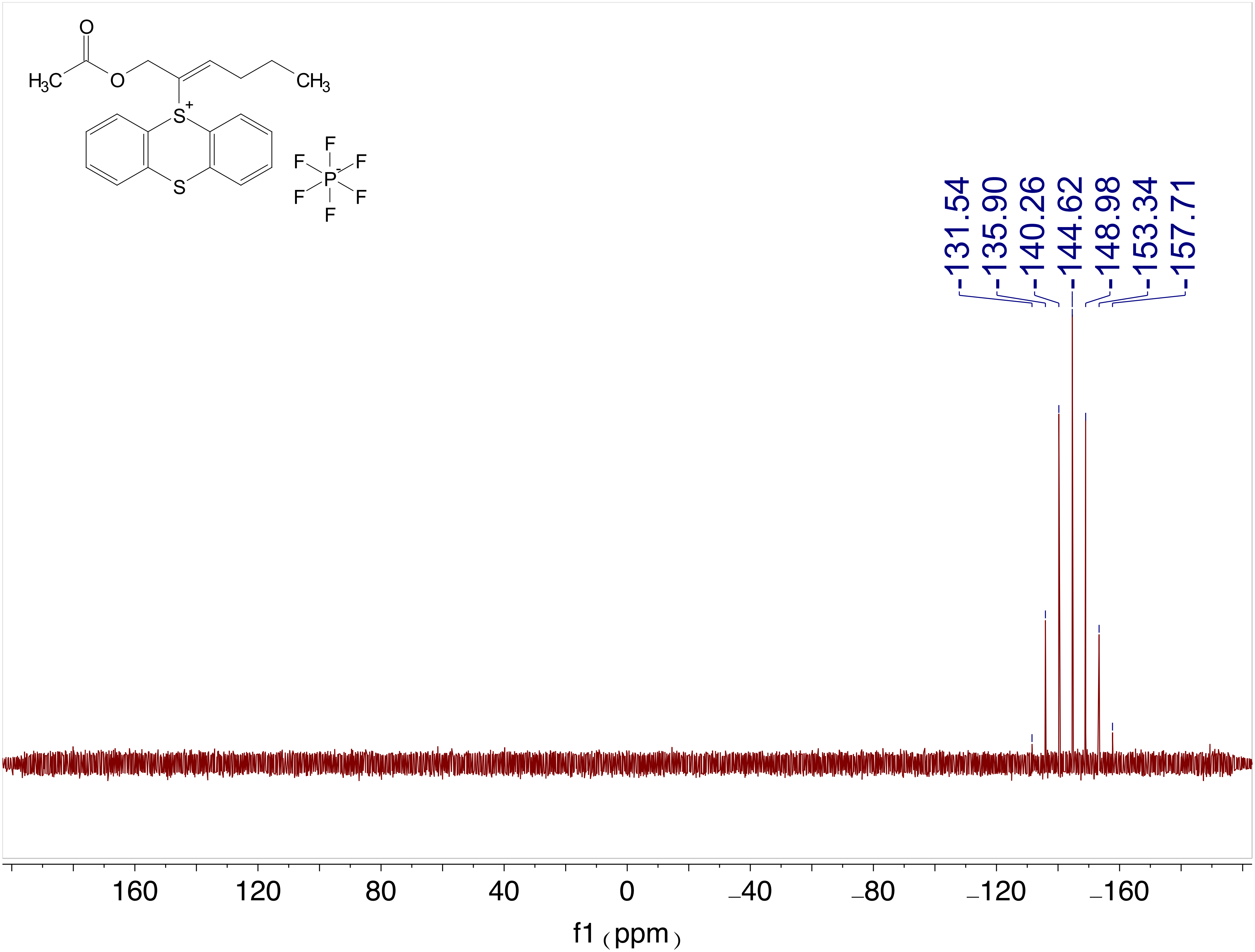

O
H3C
O
CH3
S+
S
F
F
F
P-
F
F
F
-131.54
-135.90
-140.26
-144.62
-148.98
-153.34
-157.71
160
120
80
40
0
–40
–80
–120
–160
f1 (ppm)

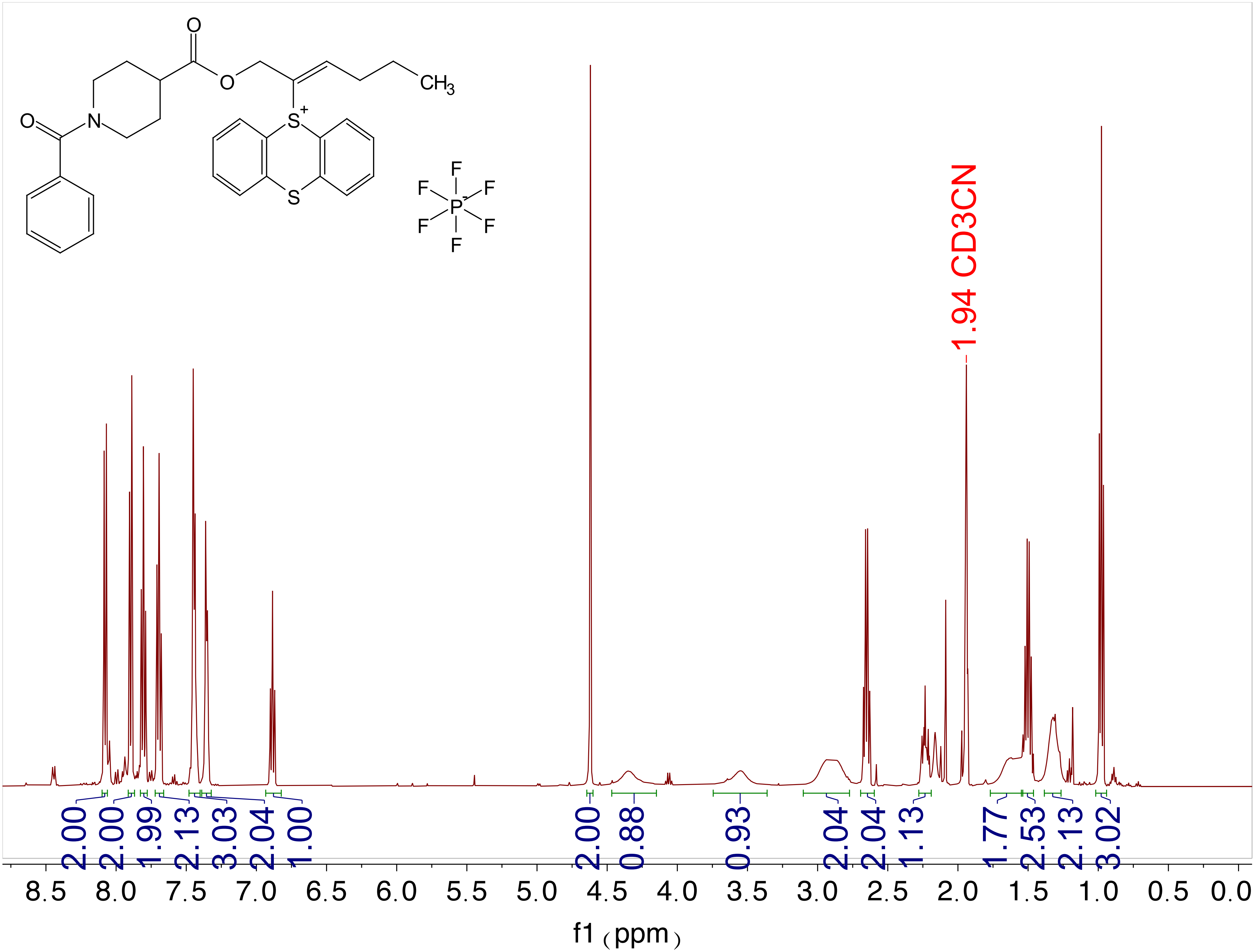
1.94 CD3CN
2.00
2.00
1.99
2.13
3.03
2.04
1.00
2.00
0.88
0.93
2.04
2.04
1.13
1.77
2.53
2.13
3.02
8.5 8.0 7.5 7.0 6.5 6.0 5.5 5.0 4.5 4.0 3.5 3.0 2.5 2.0 1.5 1.0 0.5 0.0
f1 (ppm)

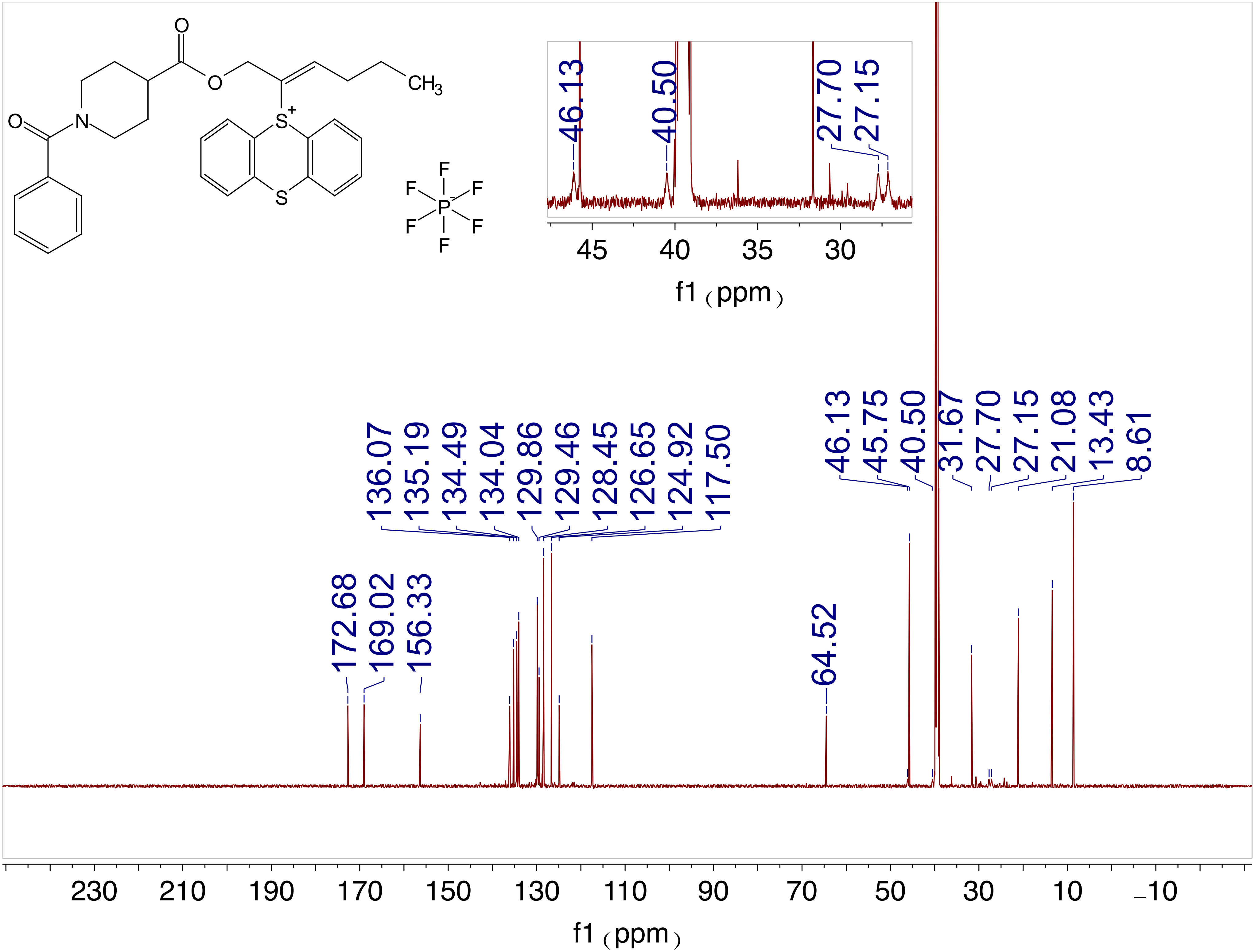

CH3
F
P
46.13
40.50
27.70
27.15
45
40
35
30
f1 (ppm)
172.68
169.02
156.33
136.07
135.19
134.49
134.04
129.86
129.46
128.45
126.65
124.92
117.50
64.52
46.13
45.75
40.50
31.67
27.70
27.15
21.08
13.43
8.61
230
210
190
170
150
130
110
90
70
50
30
10
–10
f1 (ppm)

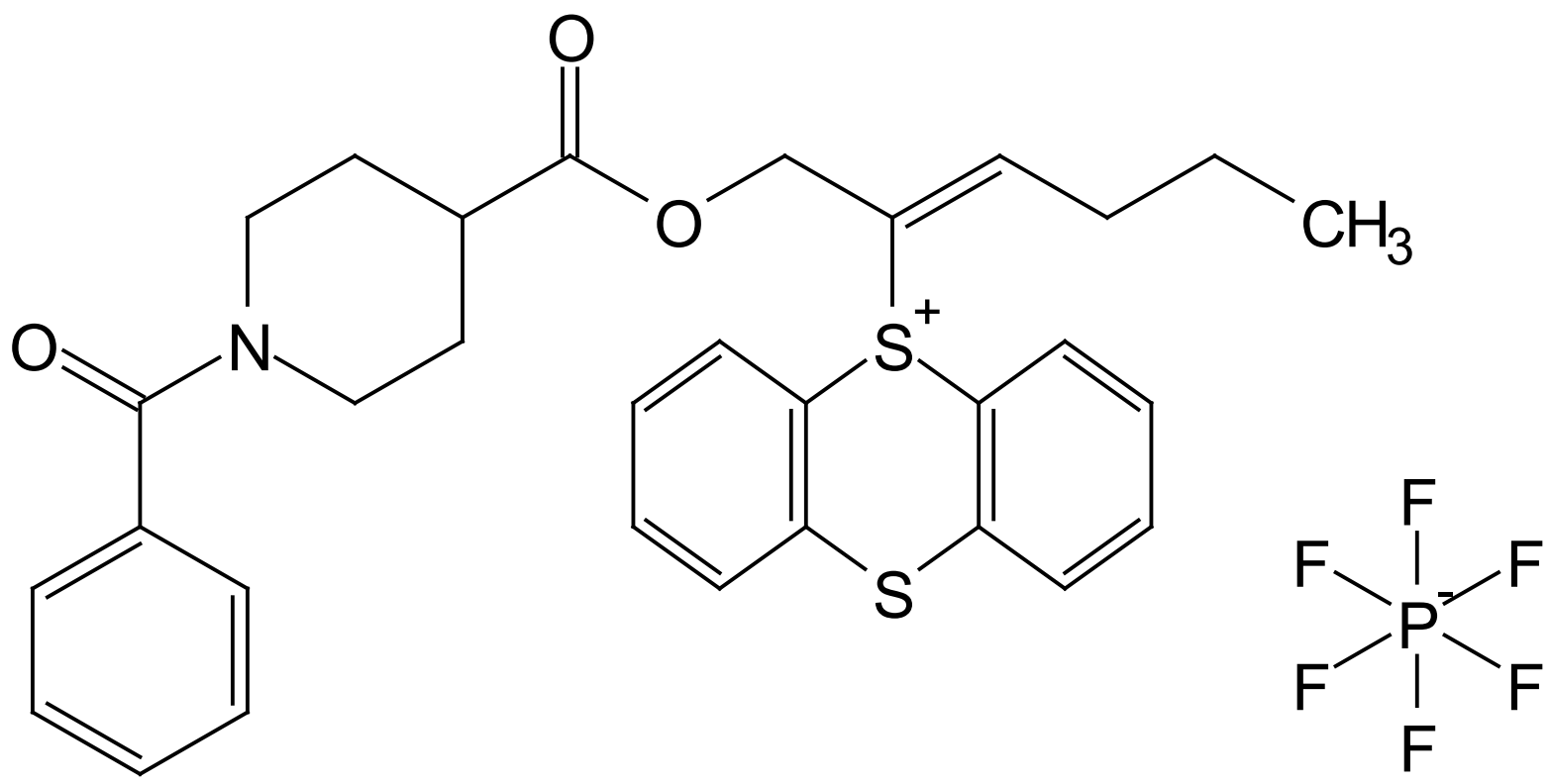

O
O
N
O
S+
S
CH3
F
F
F
F
F
F
P

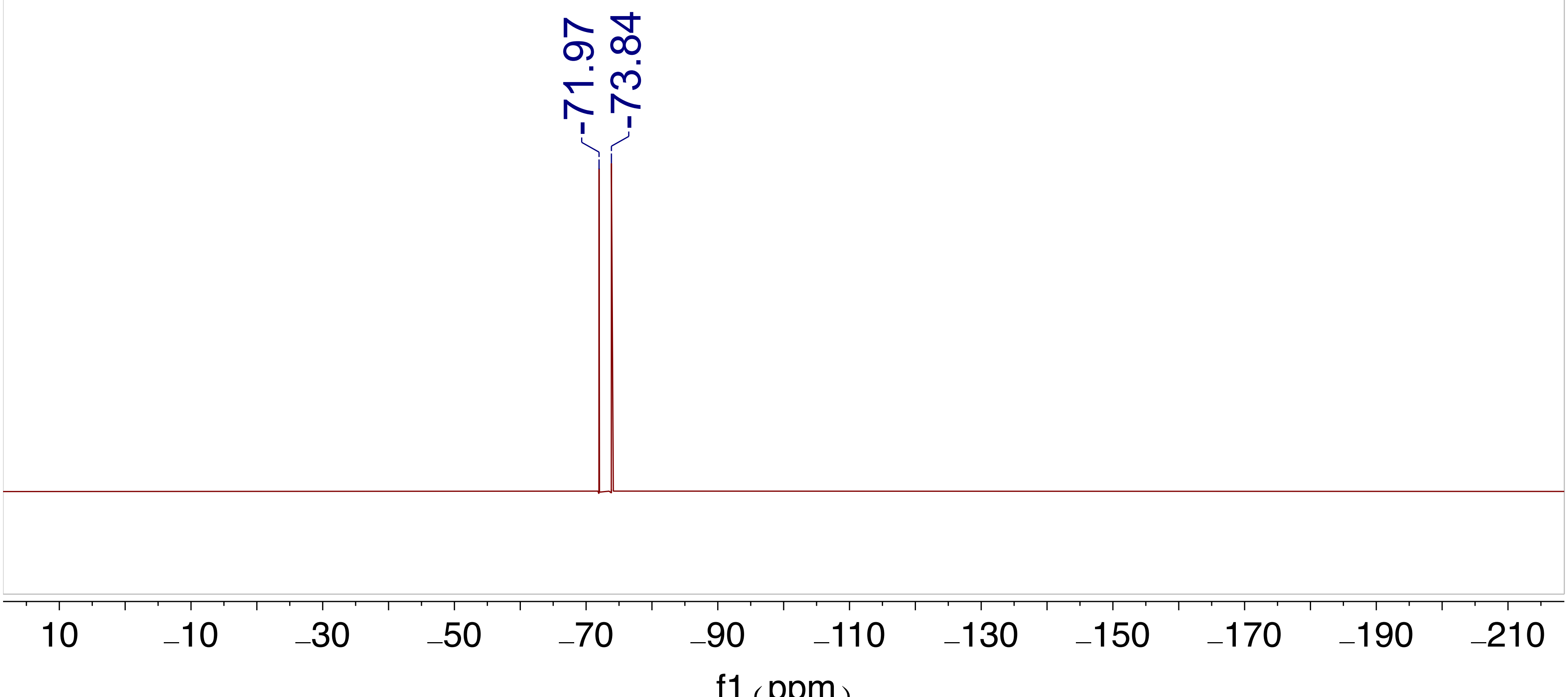

-71.97
-73.84
10
–10
–30
–50
–70
–90
–110
–130
–150
–170
–190
–210
f1（ppm）

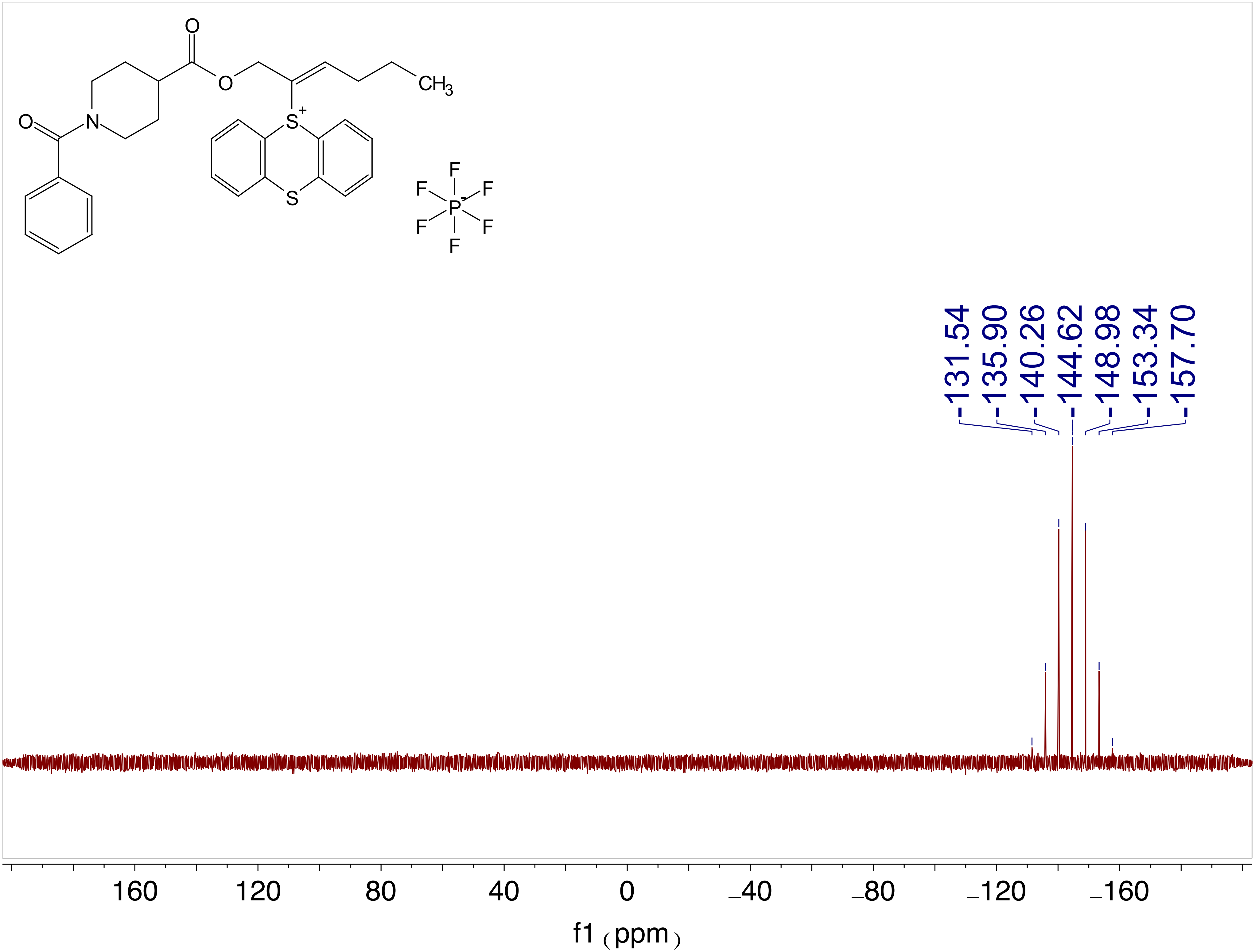

O
O
CH3
O
N
S+
S
F
F
F
P-
F
F
F
-131.54
-135.90
-140.26
-144.62
-148.98
-153.34
-157.70
160
120
80
40
0
-40
-80
-120
-160
f1 (ppm)

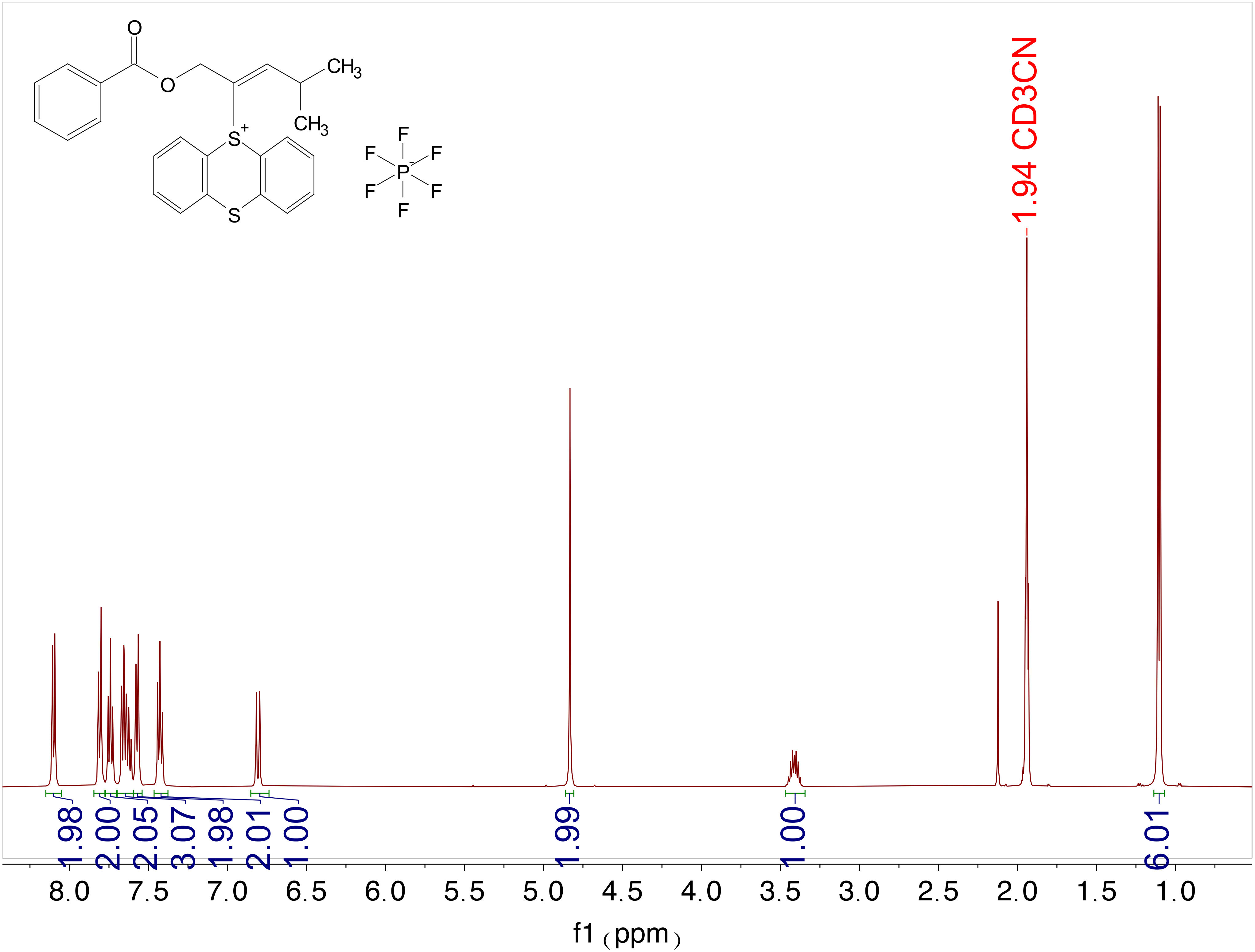

CH3
CH3
O
O
S+
S
F
F
F
P-
F
F
F
1.94 CD3CN
1.98
2.00
2.05
3.07
1.98
2.01
1.00
1.99
1.00
6.01
8.0 7.5 7.0 6.5 6.0 5.5 5.0 4.5 4.0 3.5 3.0 2.5 2.0 1.5 1.0
f1 (ppm)

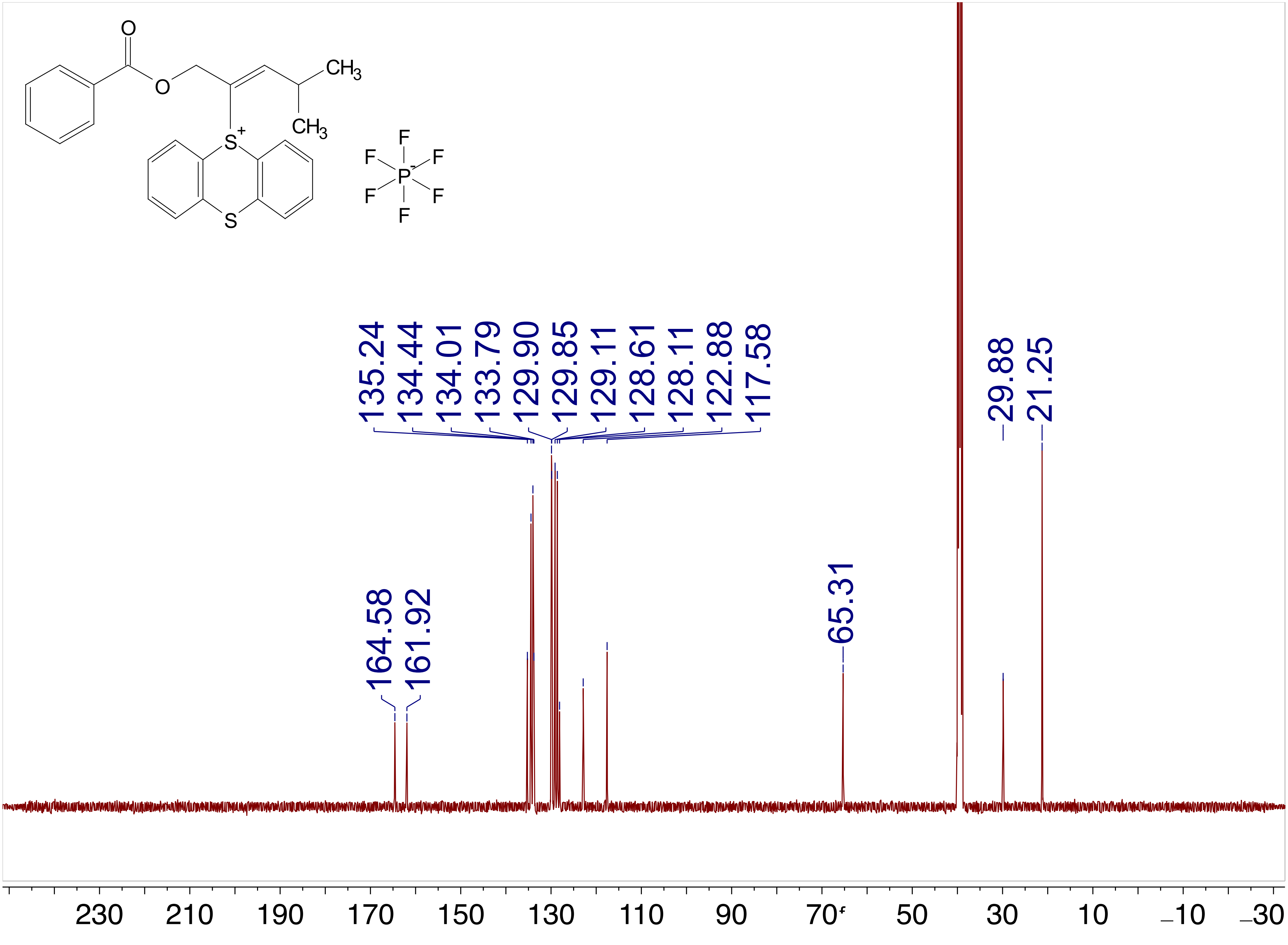

O
O
CH3
CH3
S+
S
F
F
F
P−
F
F
F
135.24
134.44
134.01
133.79
129.90
129.85
129.11
128.61
128.11
122.88
117.58
29.88
21.25
164.58
161.92
65.31
230
210
190
170
150
130
110
90
70
50
30
10
–10
–30
f1 (ppm)

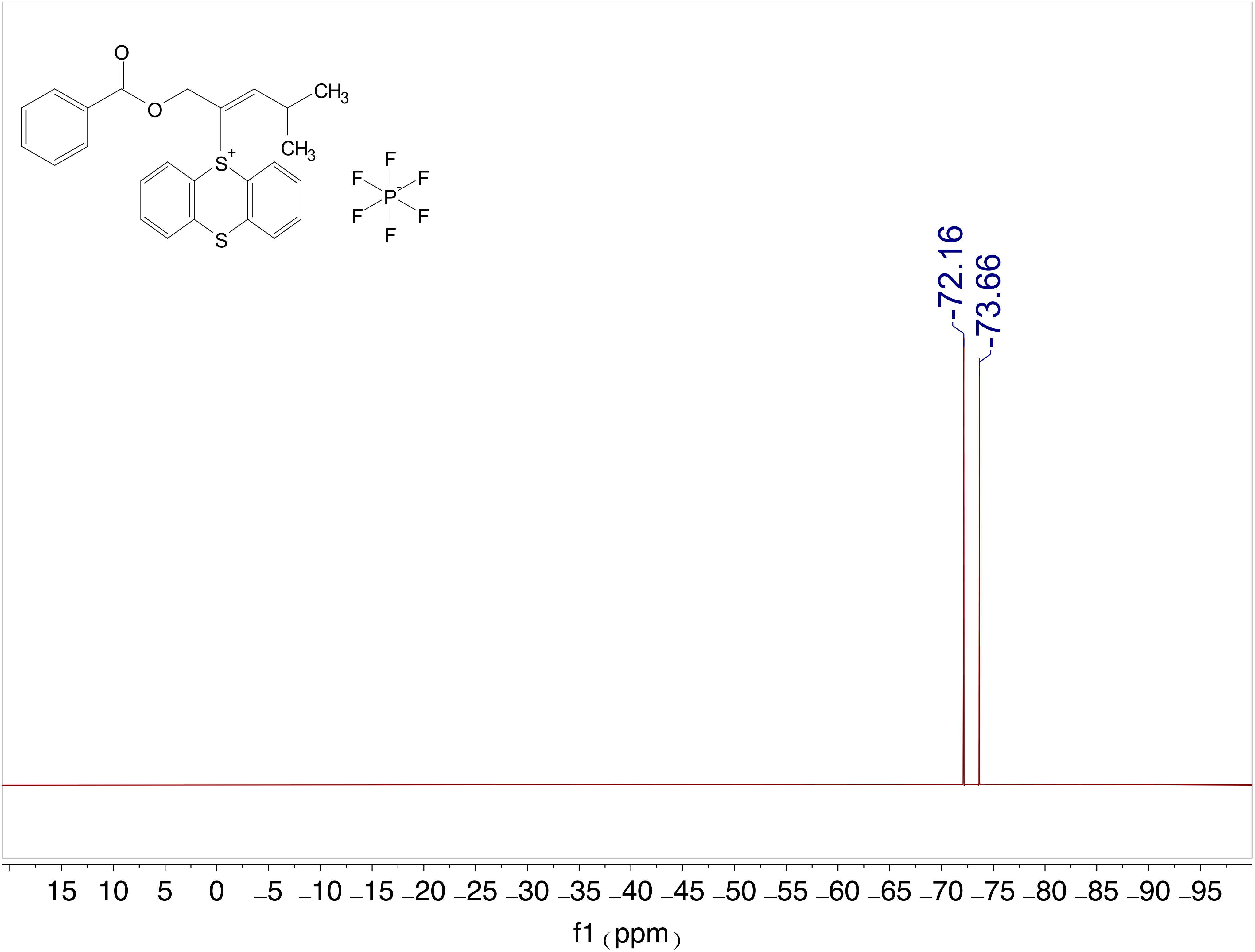

O
O
CH3
CH3
S+
S
F
F
F
P-
F
F
F
-72.16
-73.66
15
10
5
0
-5
-10
-15
-20
-25
-30
-35
-40
-45
-50
-55
-60
-65
-70
-75
-80
-85
-90
-95
f1 (ppm)

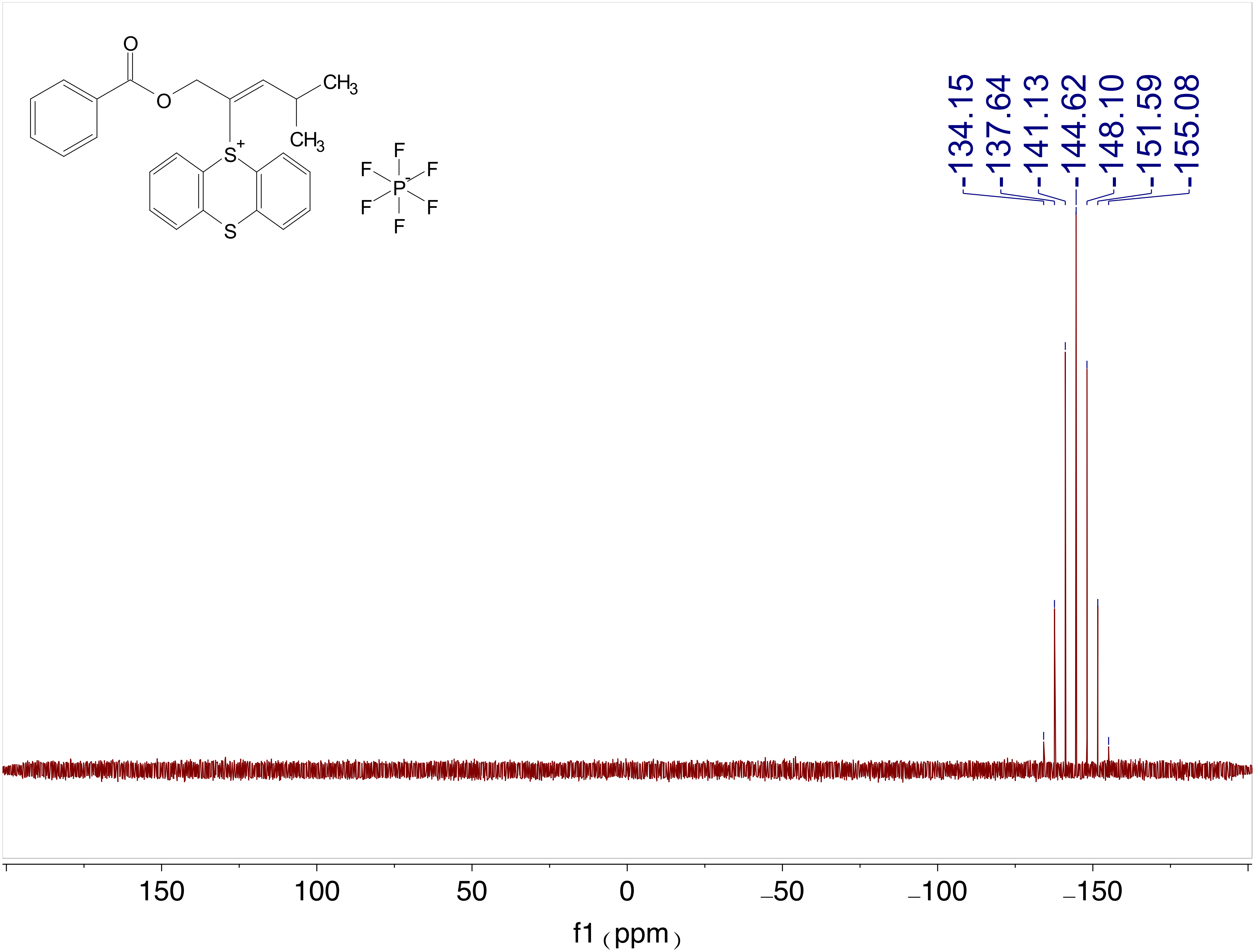

O
O
CH3
CH3
S+
S
F
F
F
P-
F
F
F
-134.15
-137.64
-141.13
-144.62
-148.10
-151.59
-155.08
150
100
50
0
–50
–100
–150
f1 (ppm)

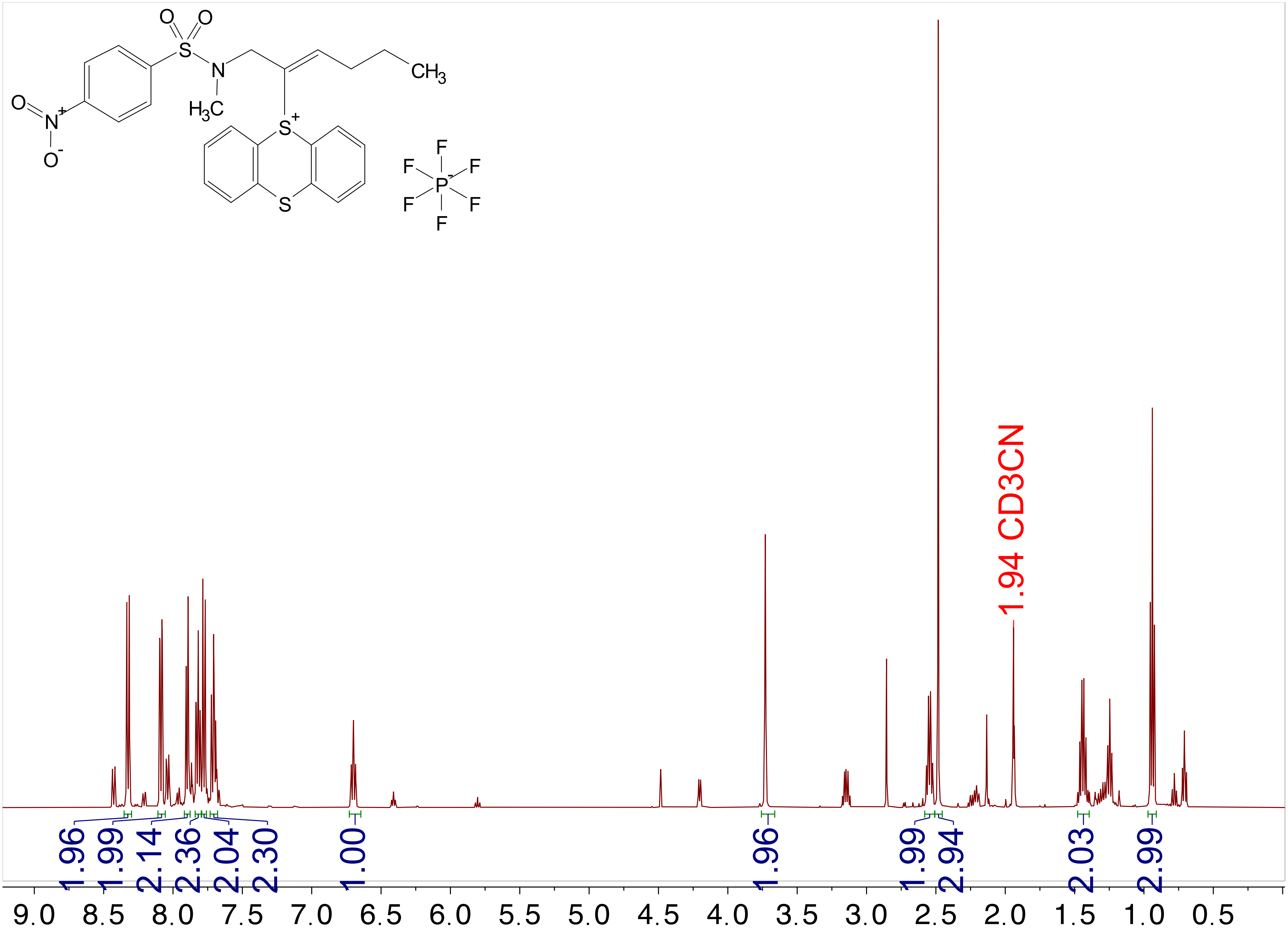

O
O
S
N
CH3
H3C
O
N+
O-
S+
S
F
F
F
P-
F
F
F
1.94 CD3CN
1.96
1.99
2.14
2.36
2.04
2.30
1.00
1.96
1.99
2.94
2.03
2.99
9.0 8.5 8.0 7.5 7.0 6.5 6.0 5.5 5.0 4.5 4.0 3.5 3.0 2.5 2.0 1.5 1.0 0.5
f1（ppm）

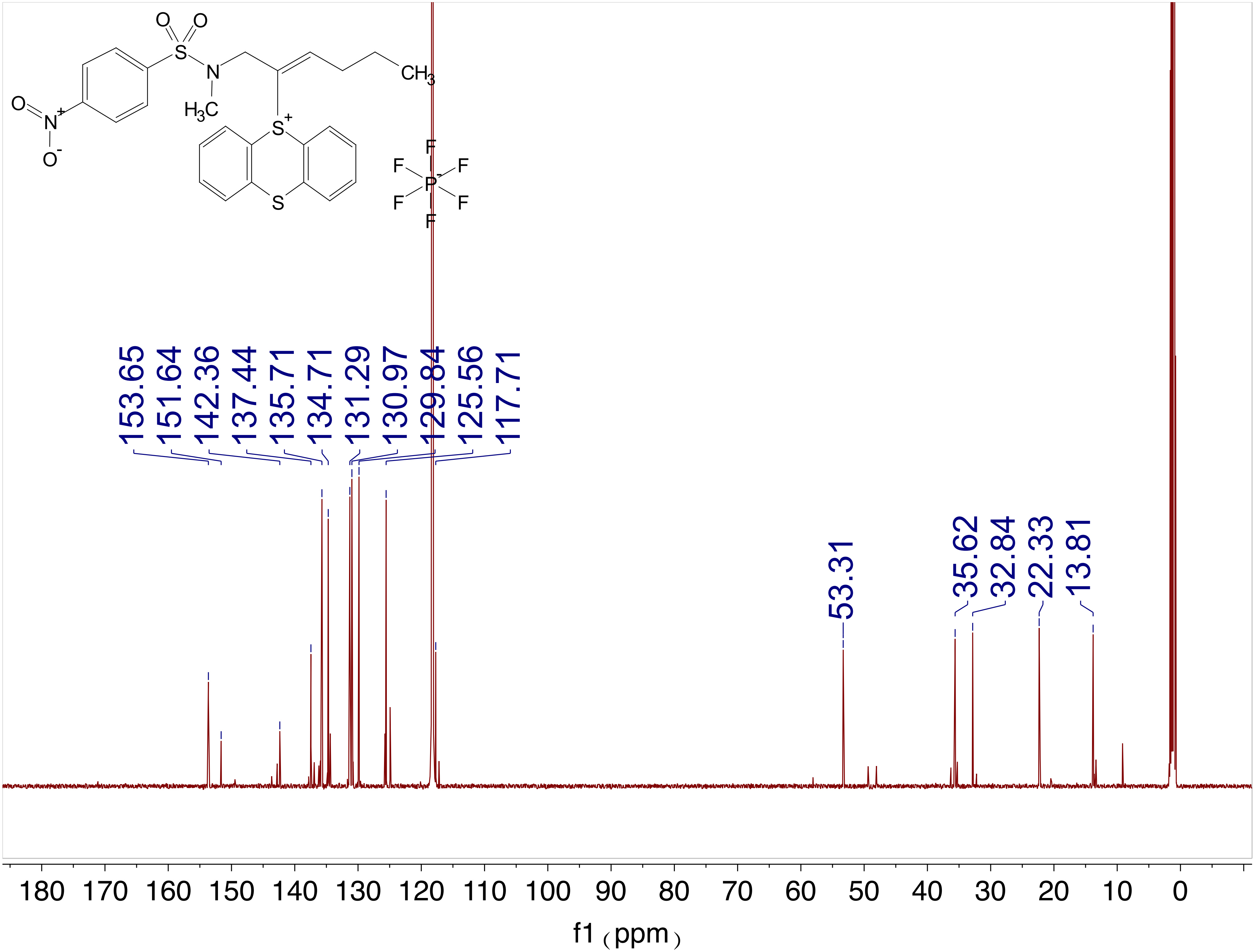
153.65
151.64
142.36
137.44
135.71
134.71
131.29
130.97
129.84
125.56
117.71
53.31
35.62
32.84
22.33
13.81
180 170 160 150 140 130 120 110 100 90 80 70 60 50 40 30 20 10 0
f1（ppm）

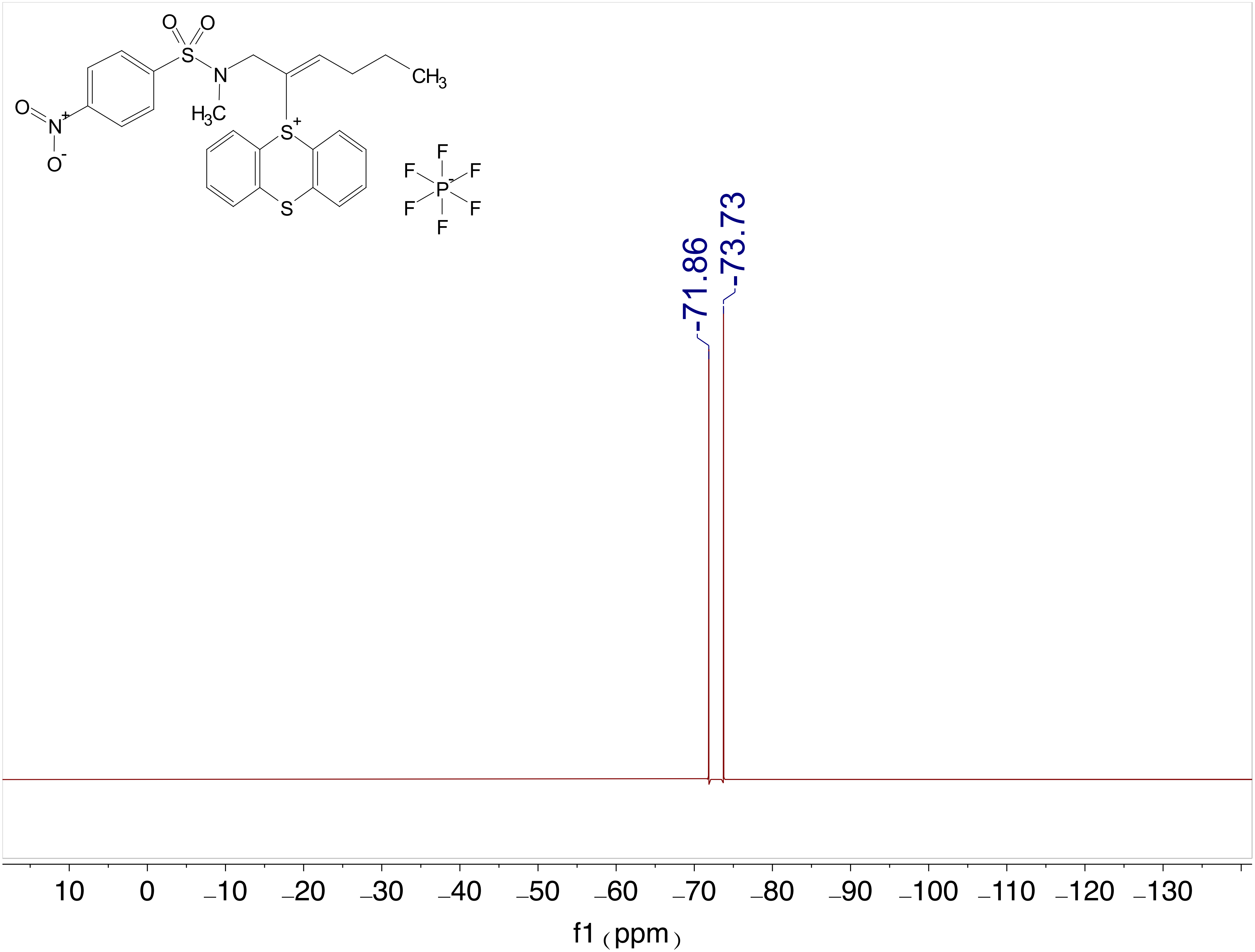

-71.86
-73.73
10
0
–10
–20
–30
–40
–50
–60
–70
–80
–90
–100
–110
–120
–130
f1 (ppm)

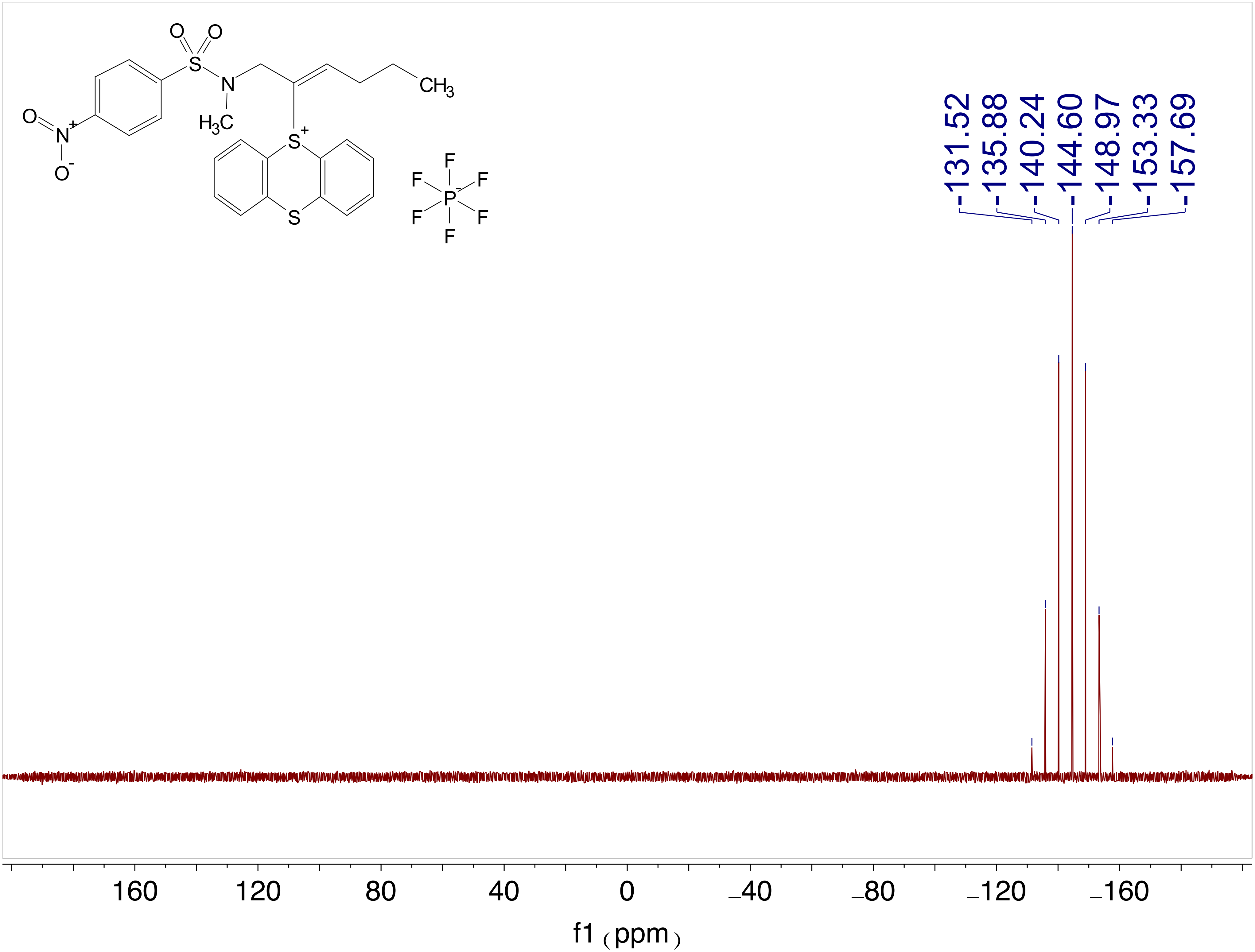

-131.52
-135.88
-140.24
-144.60
-148.97
-153.33
-157.69
160
120
80
40
0
–40
–80
–120
–160
f1 (ppm)

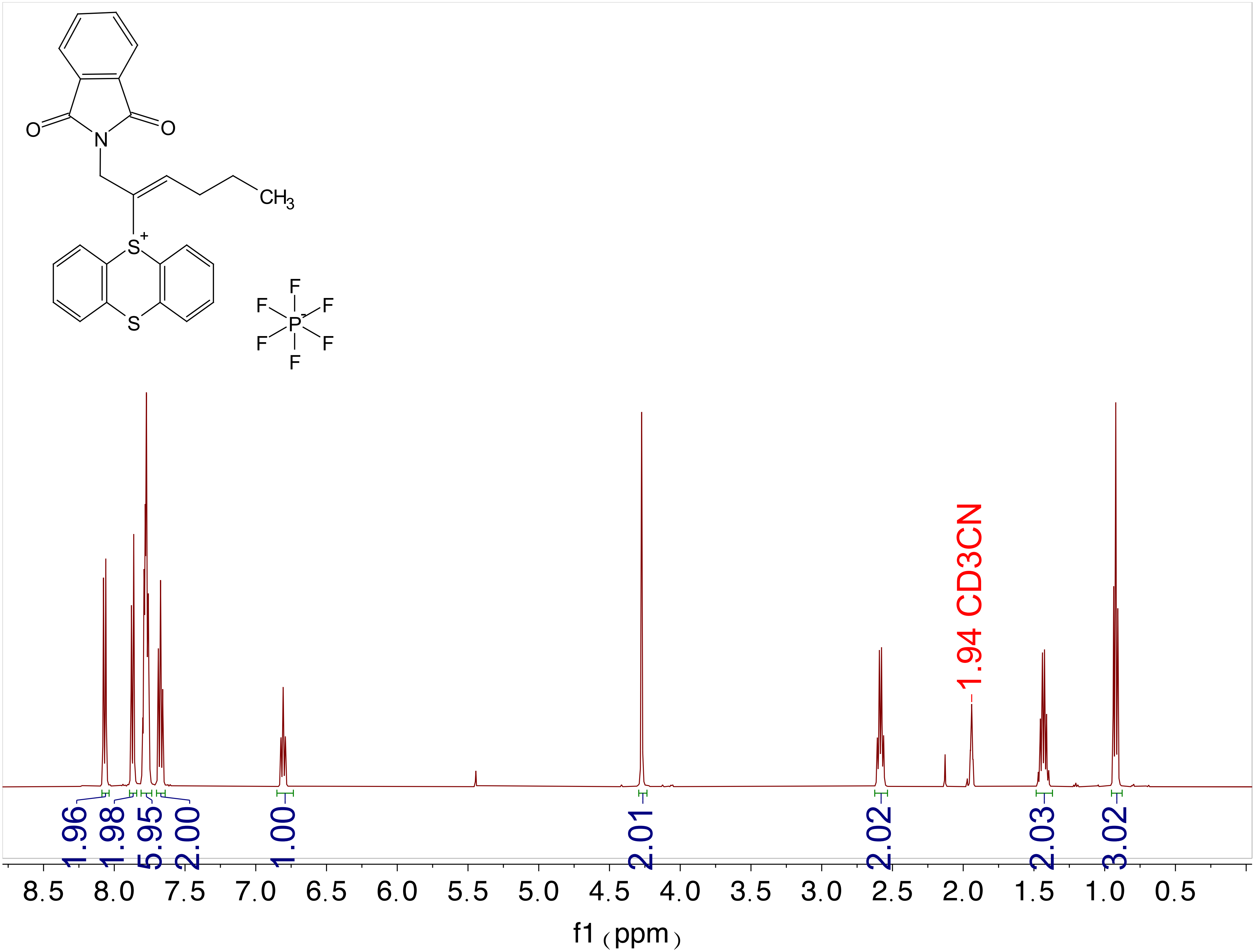

O
N
O
CH3
S+
S
F
F
F
P-
F
F
F
1.94 CD3CN
1.96
1.98
5.95
2.00
1.00
2.01
2.02
2.03
3.02
8.5
8.0
7.5
7.0
6.5
6.0
5.5
5.0
4.5
4.0
3.5
3.0
2.5
2.0
1.5
1.0
0.5
f1 (ppm)

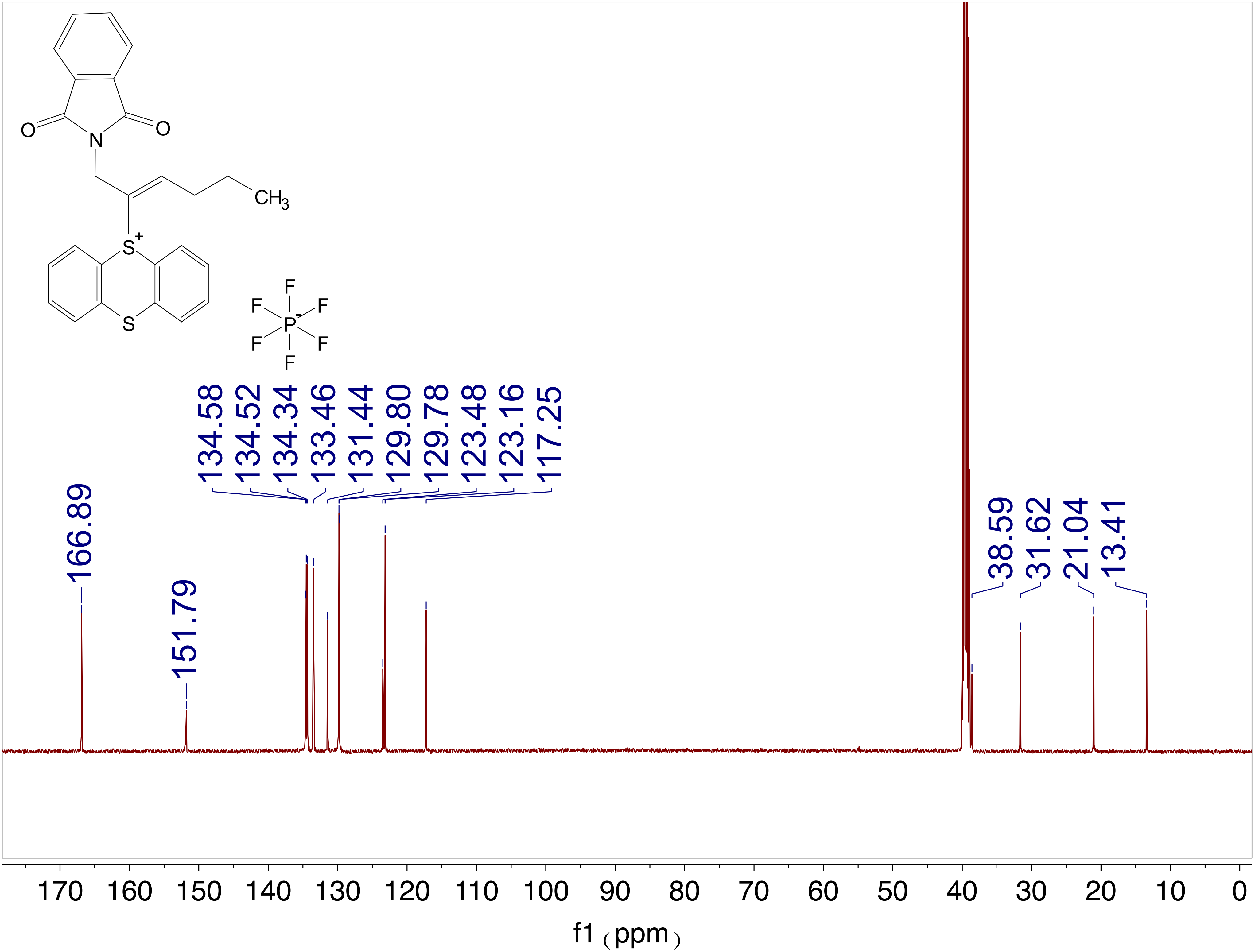
O
N
O
CH3
S+
S
F
F
F
P-
F
F
F
166.89
151.79
134.58
134.52
134.34
133.46
131.44
129.80
129.78
123.48
123.16
117.25
38.59
31.62
21.04
13.41
170 160 150 140 130 120 110 100 90 80 70 60 50 40 30 20 10 0
f1（ppm）

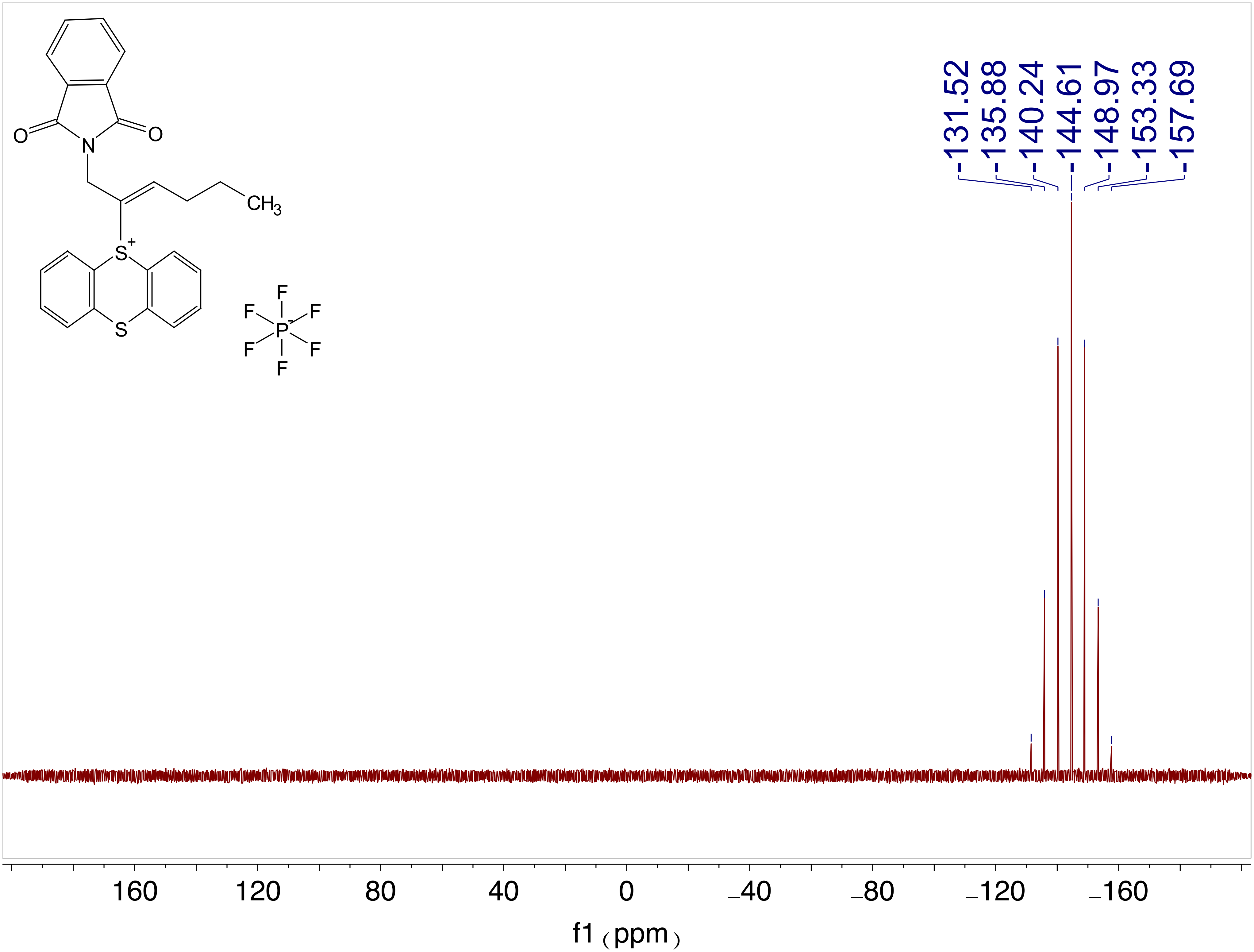

-131.52
-135.88
-140.24
-144.61
-148.97
-153.33
-157.69
CH3
O
O
N
S+
S
F
F
F
P
F
F
F
160
120
80
40
0
–40
–80
–120
–160
f1 (ppm)

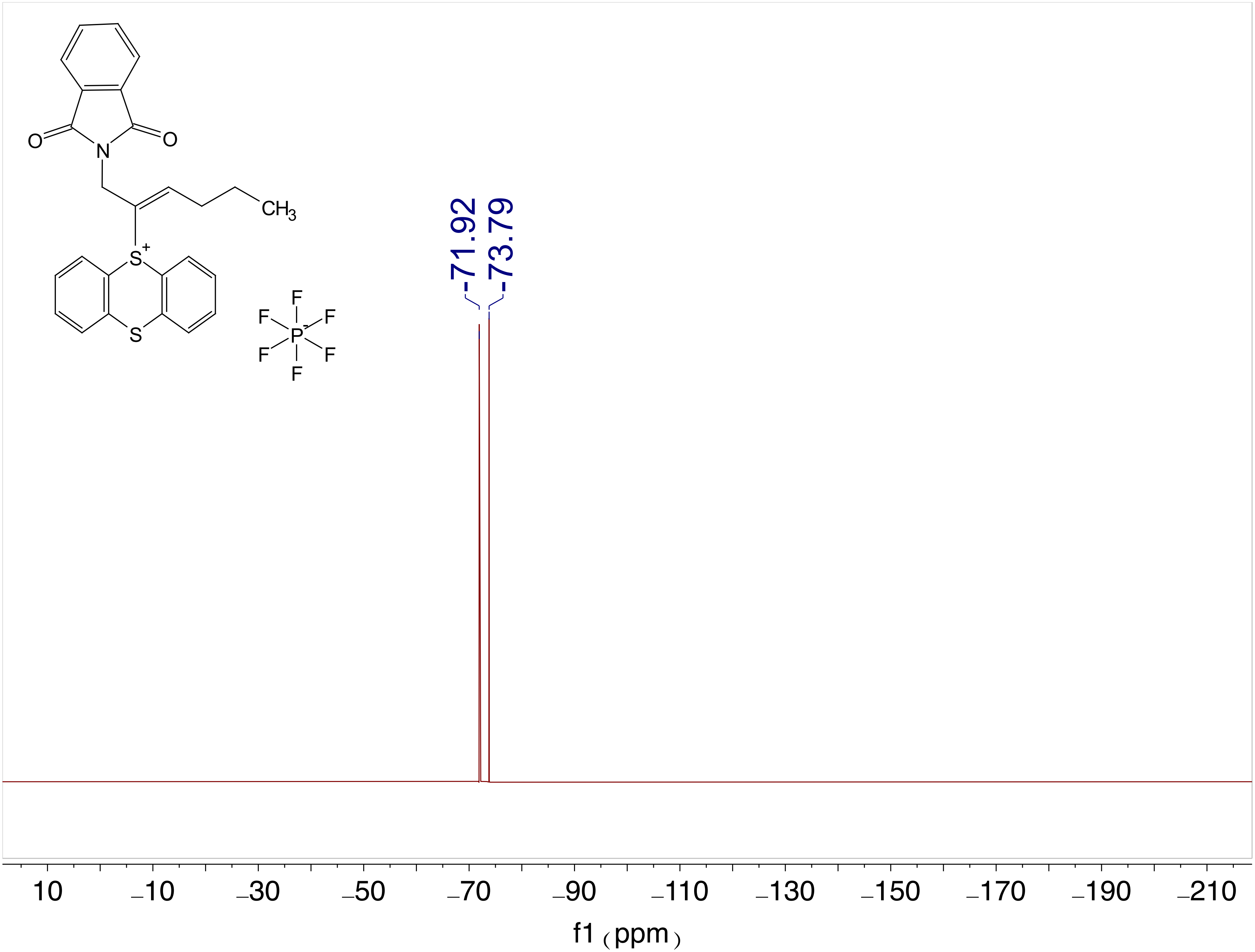

O
N
O
CH3
S+
S
F
F
F
P-
F
F
F
-71.92
-73.79
10
–10
–30
–50
–70
–90
–110
–130
–150
–170
–190
–210
f1 (ppm)

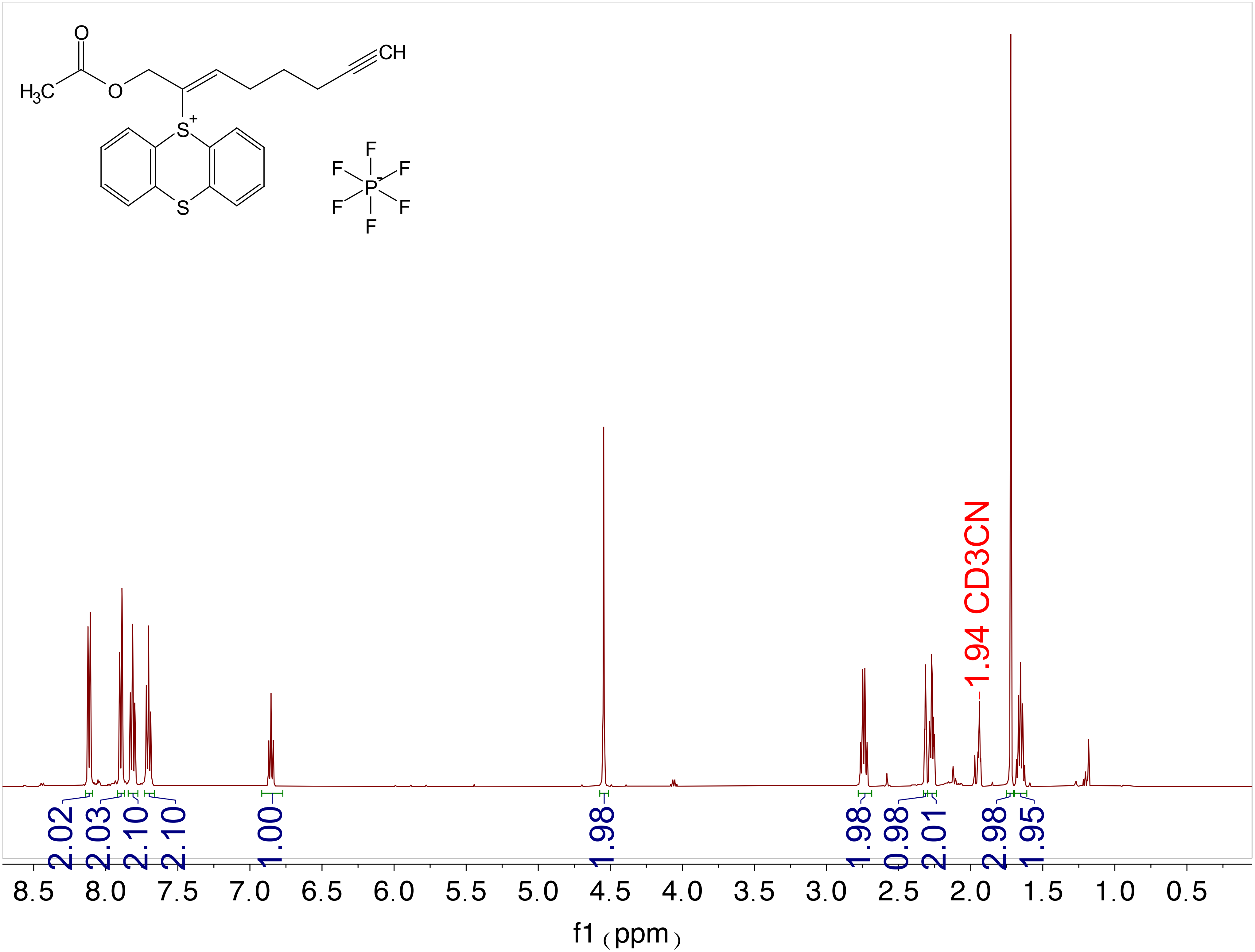

O
H3C
O
CH
S+
S
F
F
F
P-
F
F
F
1.94 CD3CN
2.02
2.03
2.10
2.10
1.00
1.98
1.98
0.98
2.01
2.98
1.95
8.5 8.0 7.5 7.0 6.5 6.0 5.5 5.0 4.5 4.0 3.5 3.0 2.5 2.0 1.5 1.0 0.5
f1 (ppm)

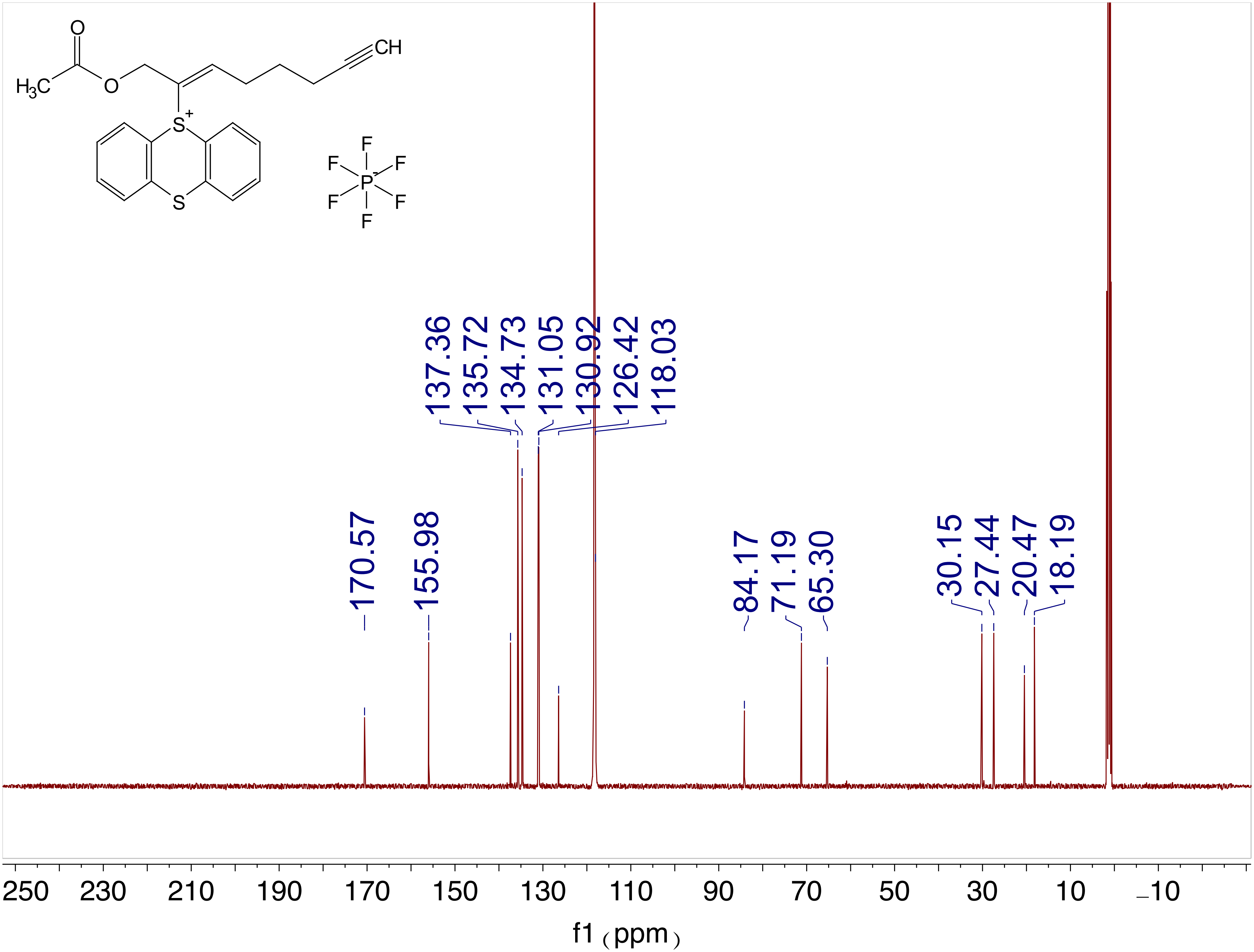

170.57
155.98
137.36
135.72
134.73
131.05
130.92
126.42
118.03
84.17
71.19
65.30
30.15
27.44
20.47
18.19
250 230 210 190 170 150 130 110 90 70 50 30 10 –10
f1 (ppm)

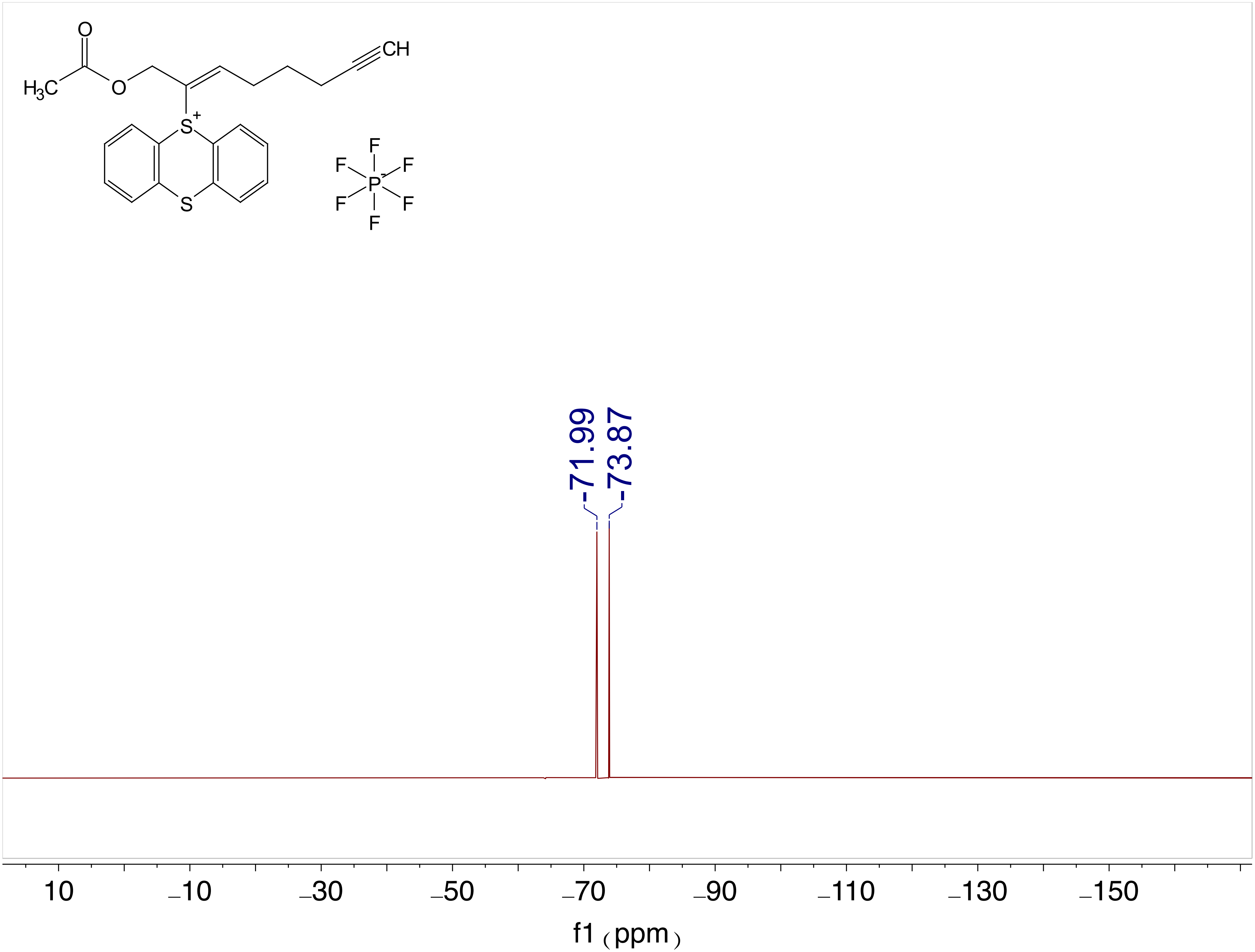

O
H_3C
O
CH
S+
S
F
F
F
P
F
F
F
-71.99
-73.87
10
–10
–30
–50
–70
–90
–110
–130
–150
f1 (ppm)

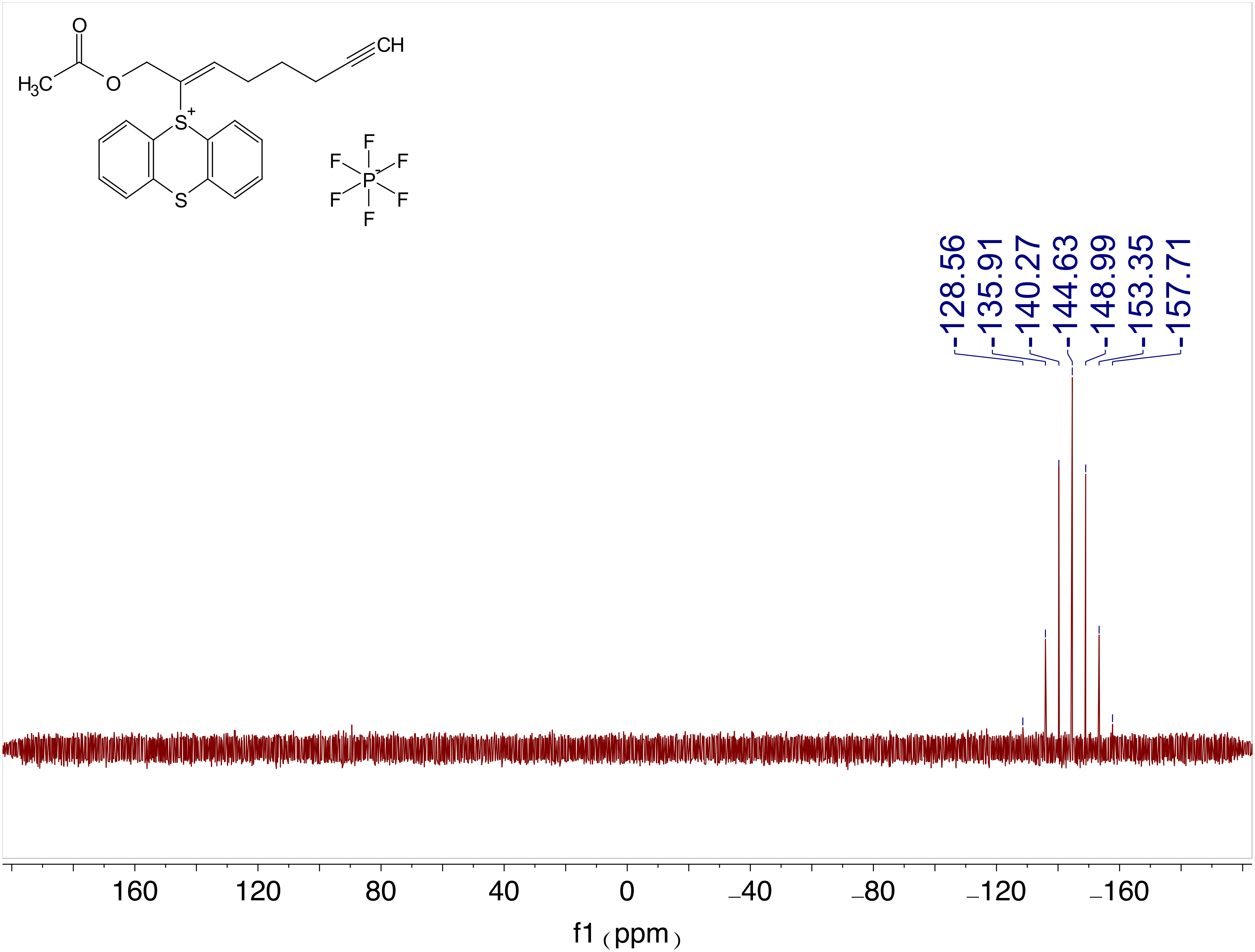
O
CH
H3C
O
S+
S
F
F
F
P−
F
F
F
-128.56
-135.91
-140.27
-144.63
-148.99
-153.35
-157.71
160
120
80
40
0
−40
−80
−120
−160
f1 (ppm)

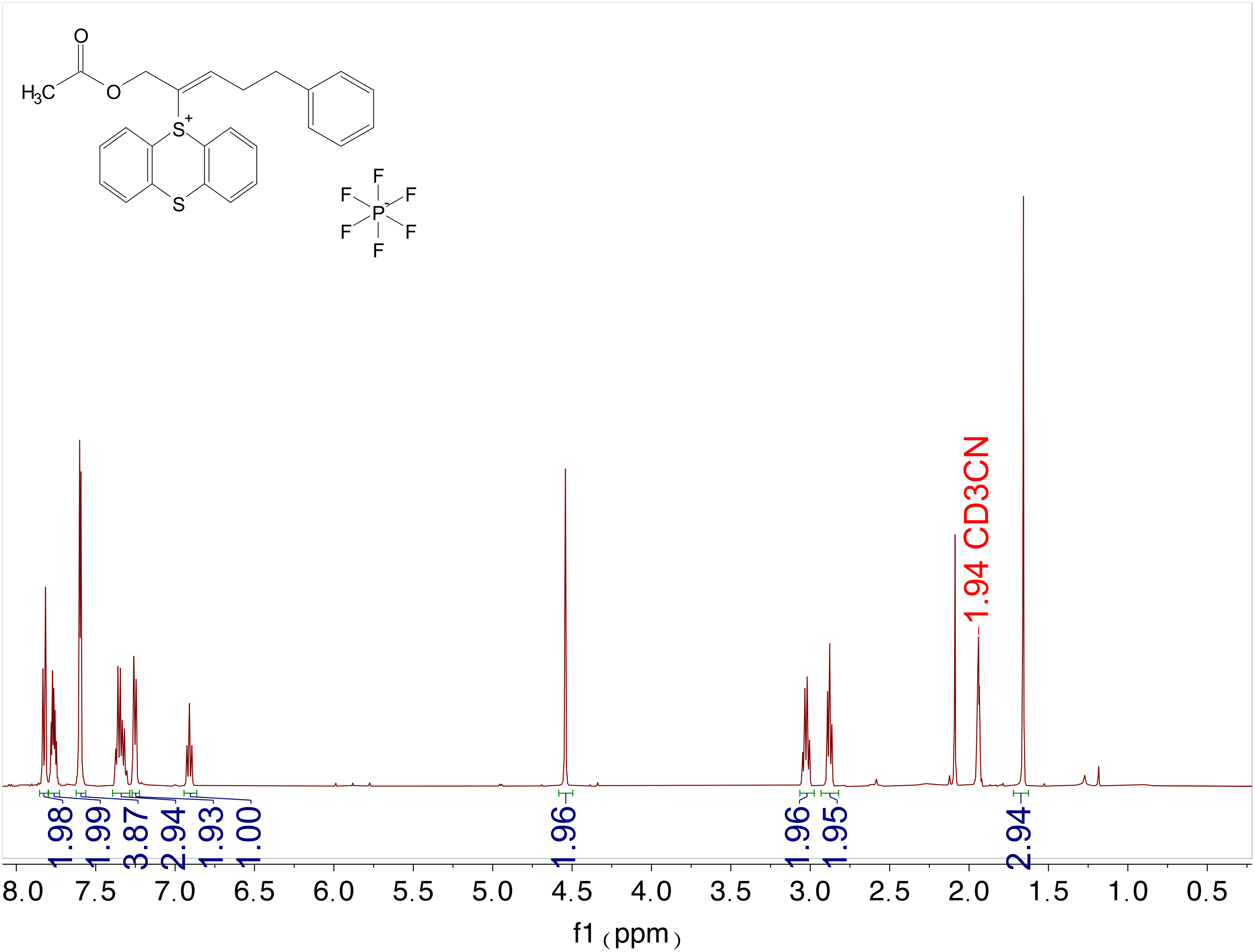

O
H_3C
O
S+
S
F
P-
1.94 CD3CN
1.98
1.99
3.87
2.94
1.93
1.00
1.96
1.96
1.95
2.94
8.0 7.5 7.0 6.5 6.0 5.5 5.0 4.5 4.0 3.5 3.0 2.5 2.0 1.5 1.0 0.5
f1（ppm）

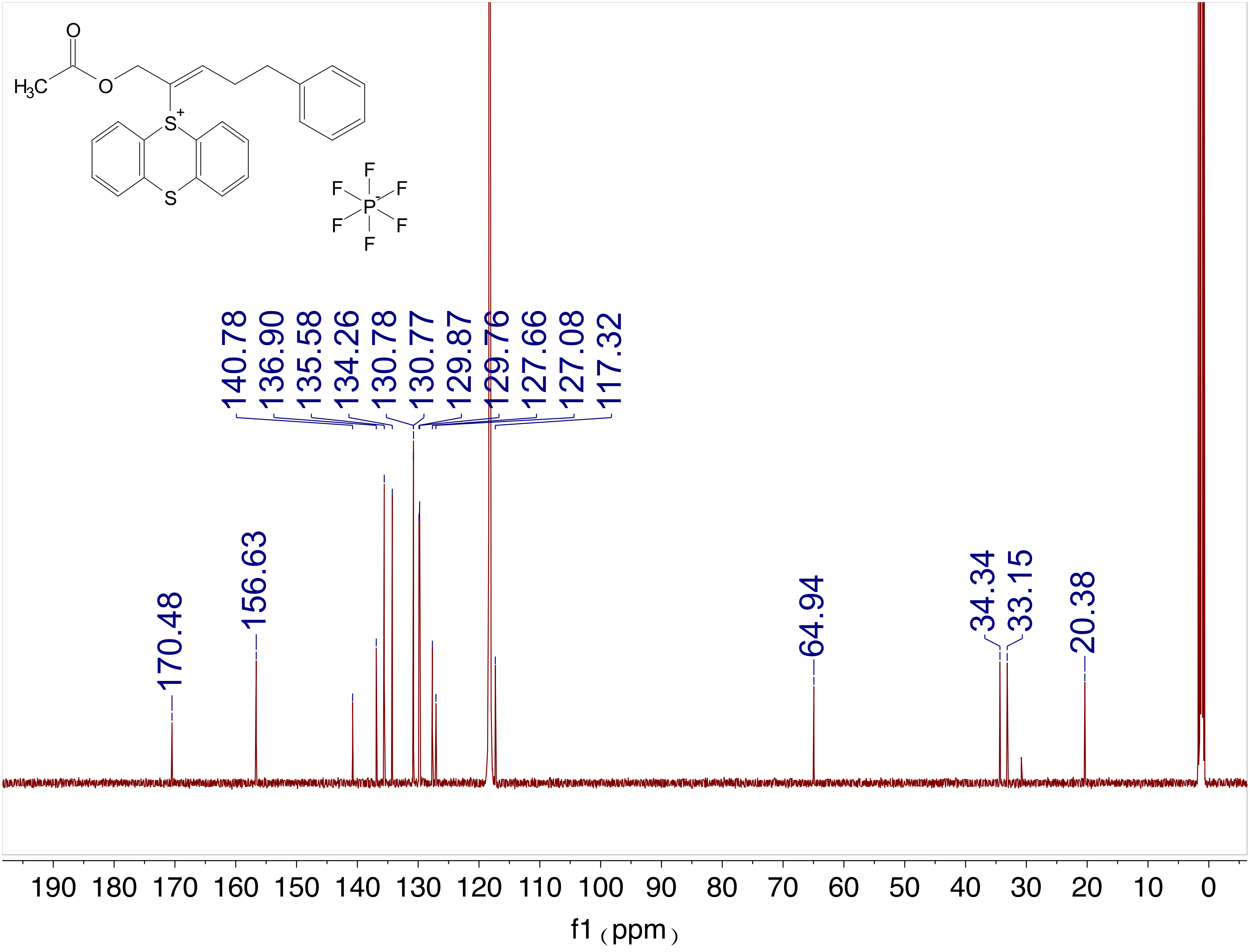

O
H3C
O
S+
S
F
F
F
P-
F
F
F
140.78
136.90
135.58
134.26
130.78
130.77
129.87
129.76
127.66
127.08
117.32
170.48
156.63
64.94
34.34
33.15
20.38
190 180 170 160 150 140 130 120 110 100 90 80 70 60 50 40 30 20 10 0
f1 (ppm)

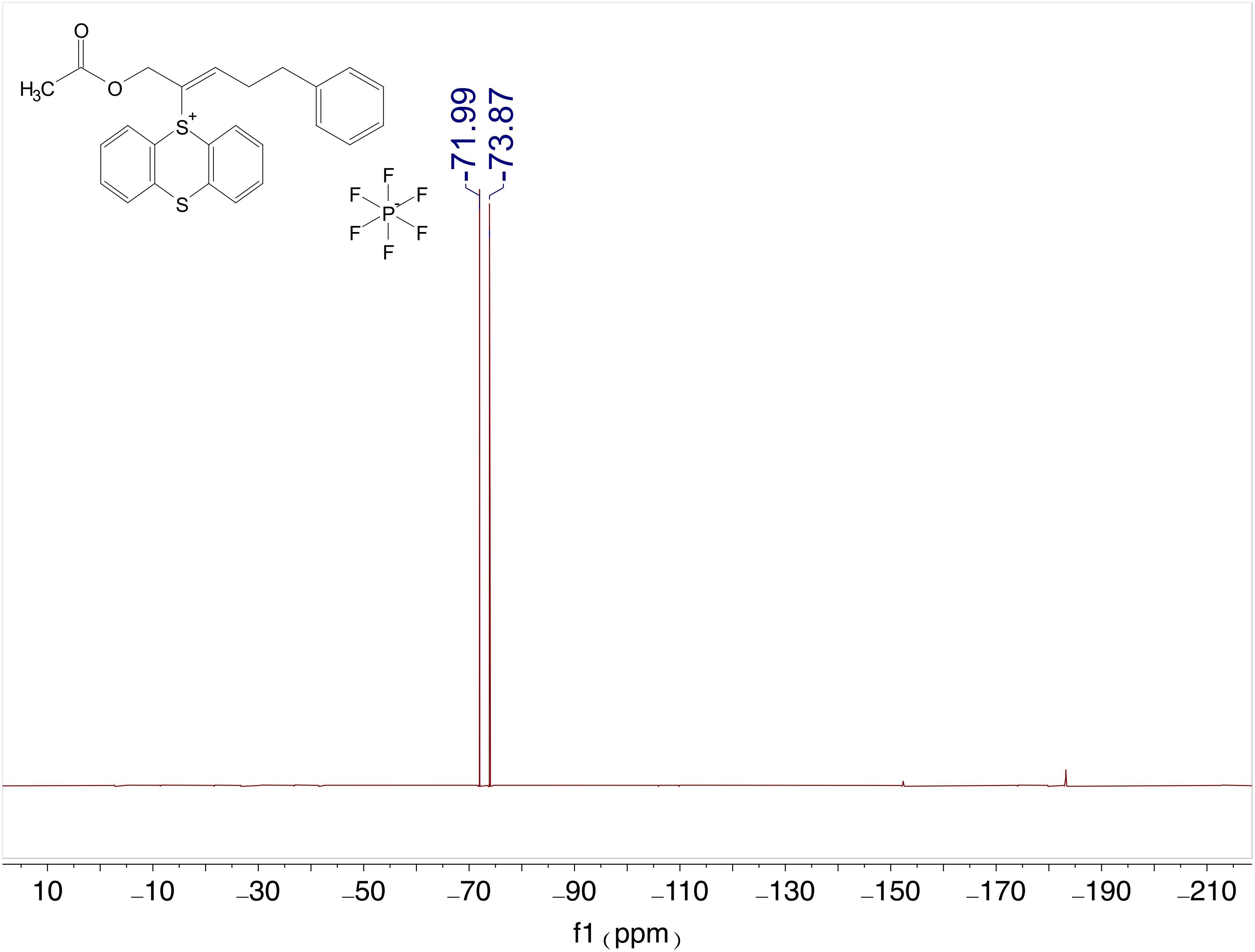

-71.99
-73.87
H3C
O
O
S+
S
F
F
F
P-
F
F
F
10
–10
–30
–50
–70
–90
–110
–130
–150
–170
–190
–210
f1（ppm）

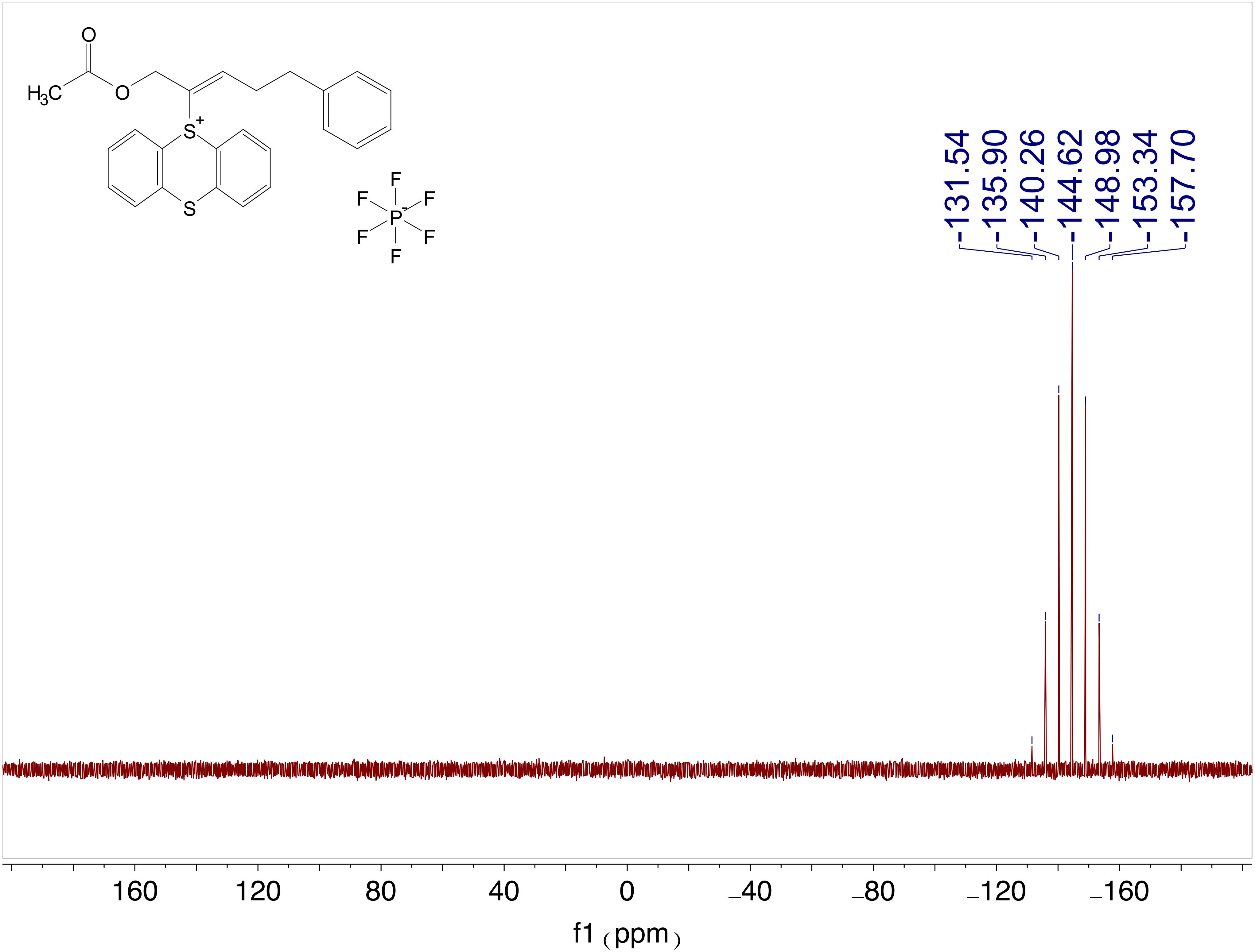

-131.54
-135.90
-140.26
-144.62
-148.98
-153.34
-157.70
160
120
80
40
0
–40
–80
–120
–160
f1 (ppm)

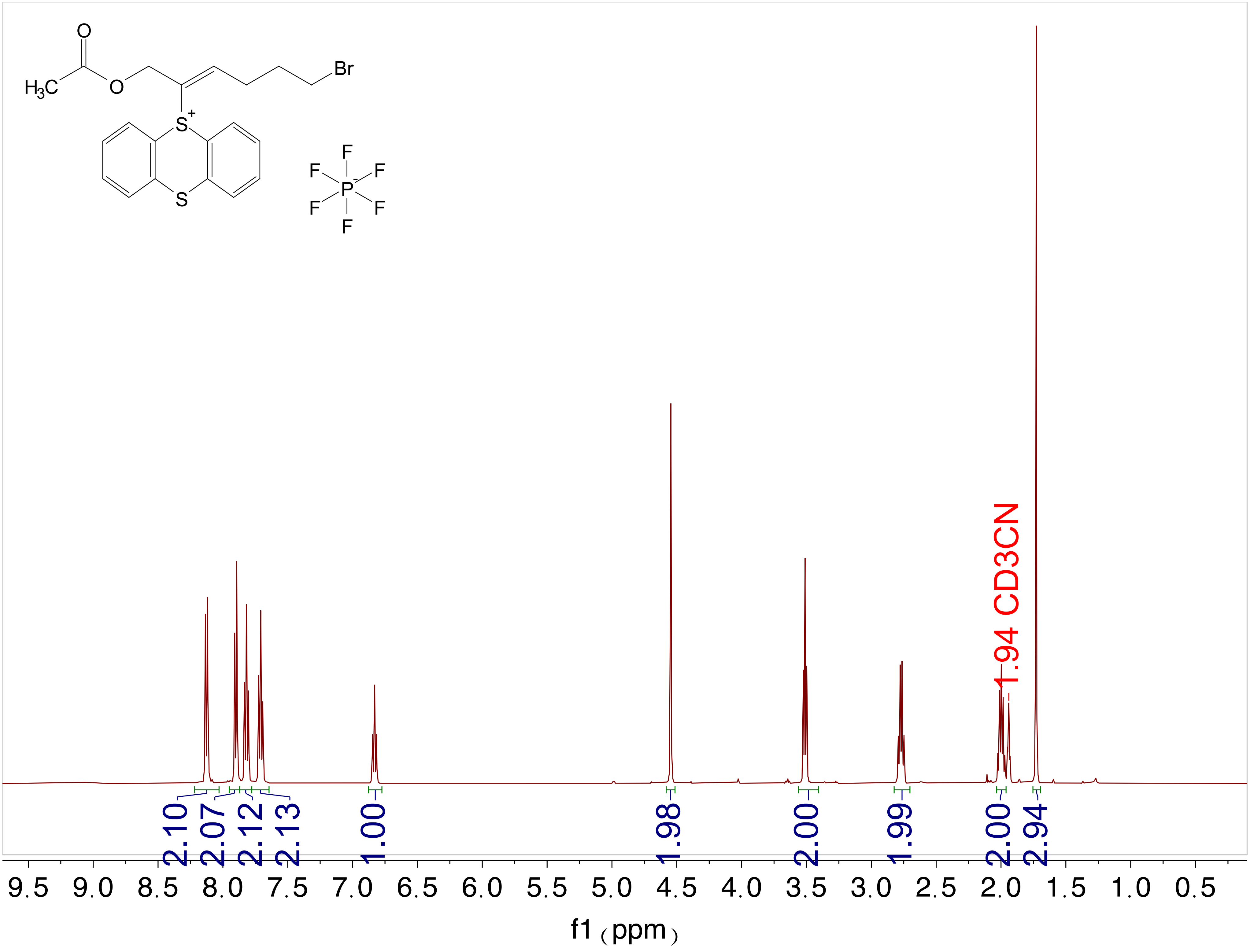

O
H_3C
O
Br
S+
S
F
F
F
P
F
F
F
1.94 CD3CN
2.10
2.07
2.12
2.13
1.00
1.98
2.00
1.99
2.00
2.94
9.5 9.0 8.5 8.0 7.5 7.0 6.5 6.0 5.5 5.0 4.5 4.0 3.5 3.0 2.5 2.0 1.5 1.0 0.5
f1（ppm）

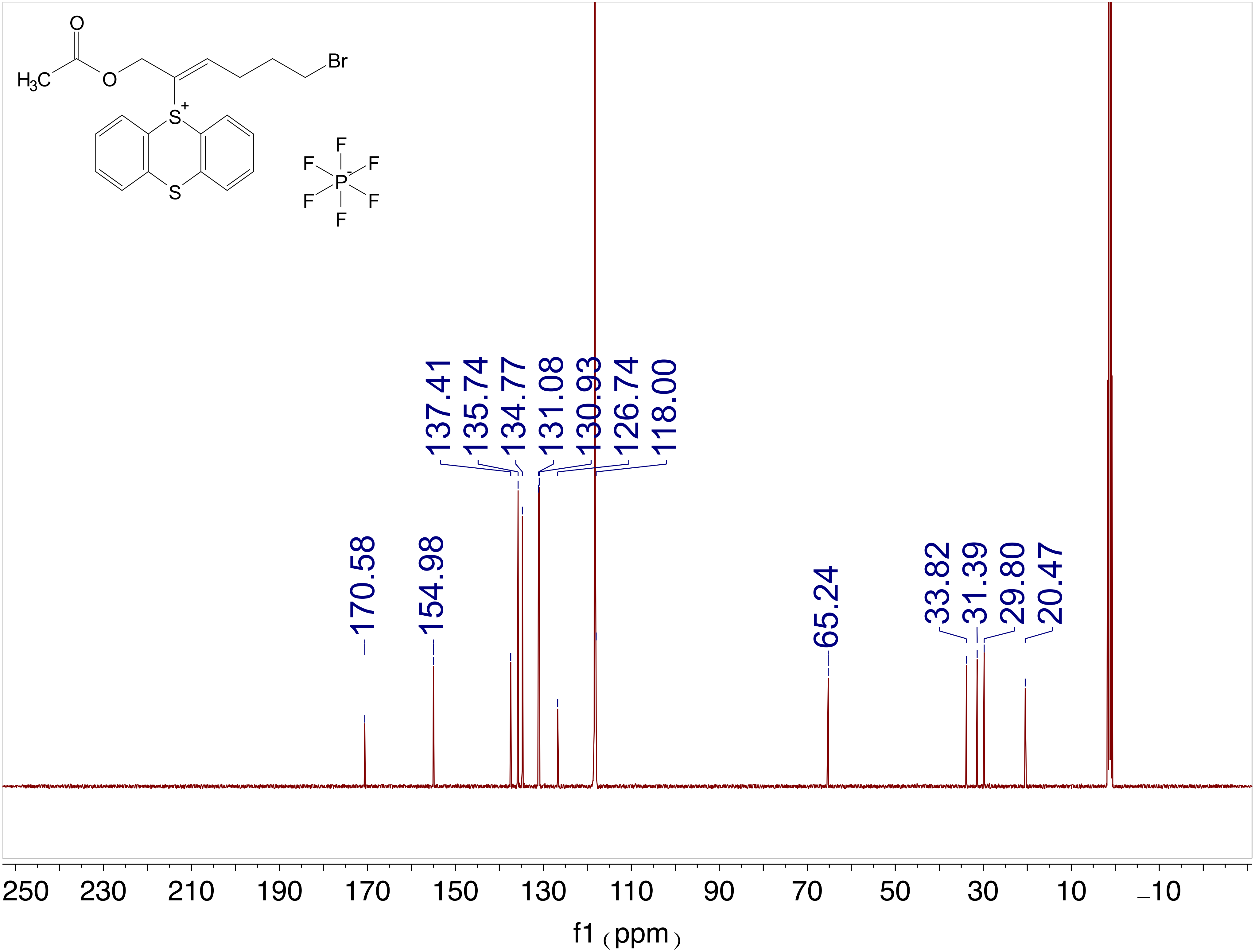

O
H_3C
O
Br
S^+
S
F
F
F
P
F
F
F
137.41
135.74
134.77
131.08
130.93
126.74
118.00
170.58
154.98
65.24
33.82
31.39
29.80
20.47
250
230
210
190
170
150
130
110
90
70
50
30
10
–10
f1 (ppm)

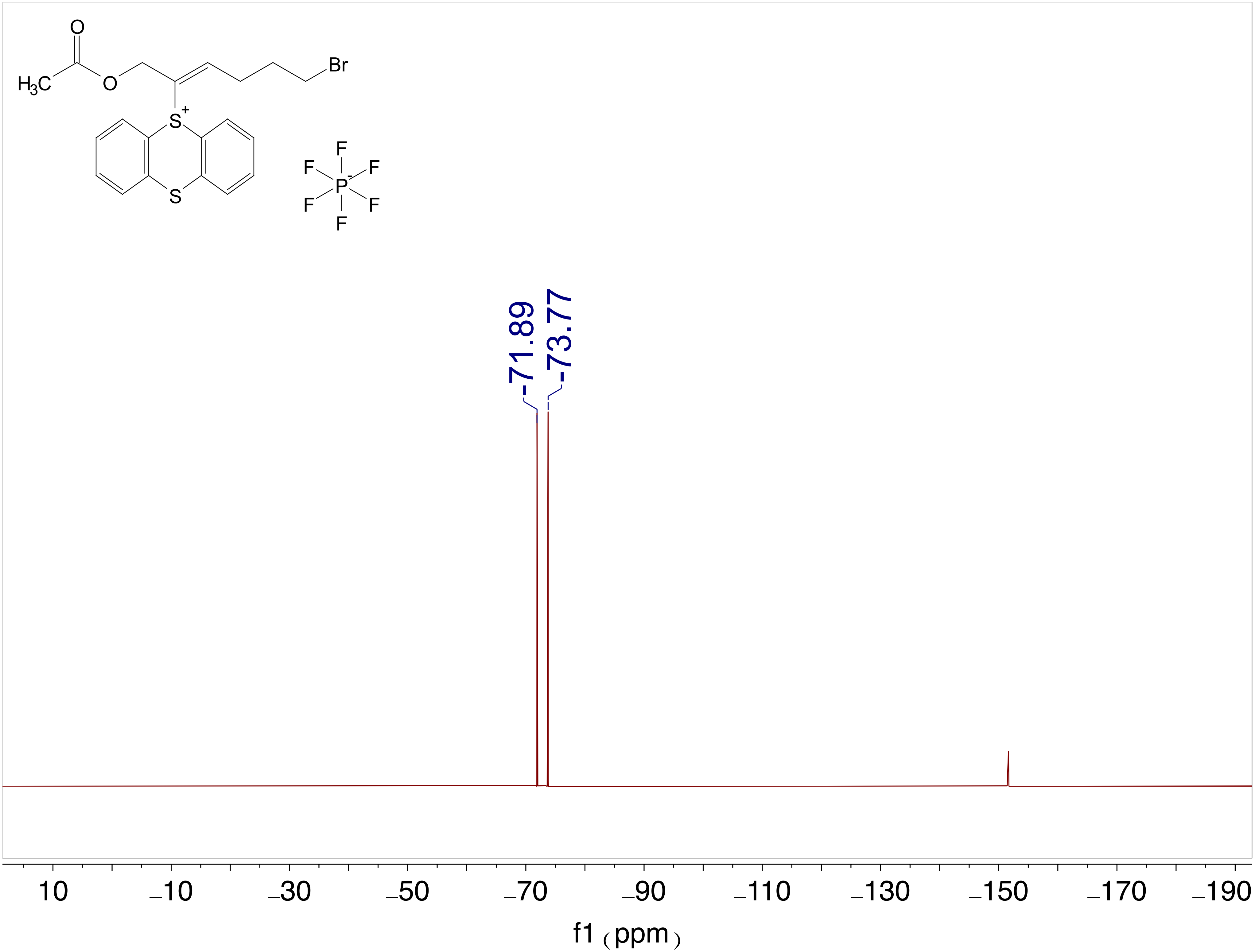

O
H_3C
O
Br
S+
S
F
F
F
P-
F
F
F
-71.89
-73.77
10
–10
–30
–50
–70
–90
–110
–130
–150
–170
–190
f1 (ppm)

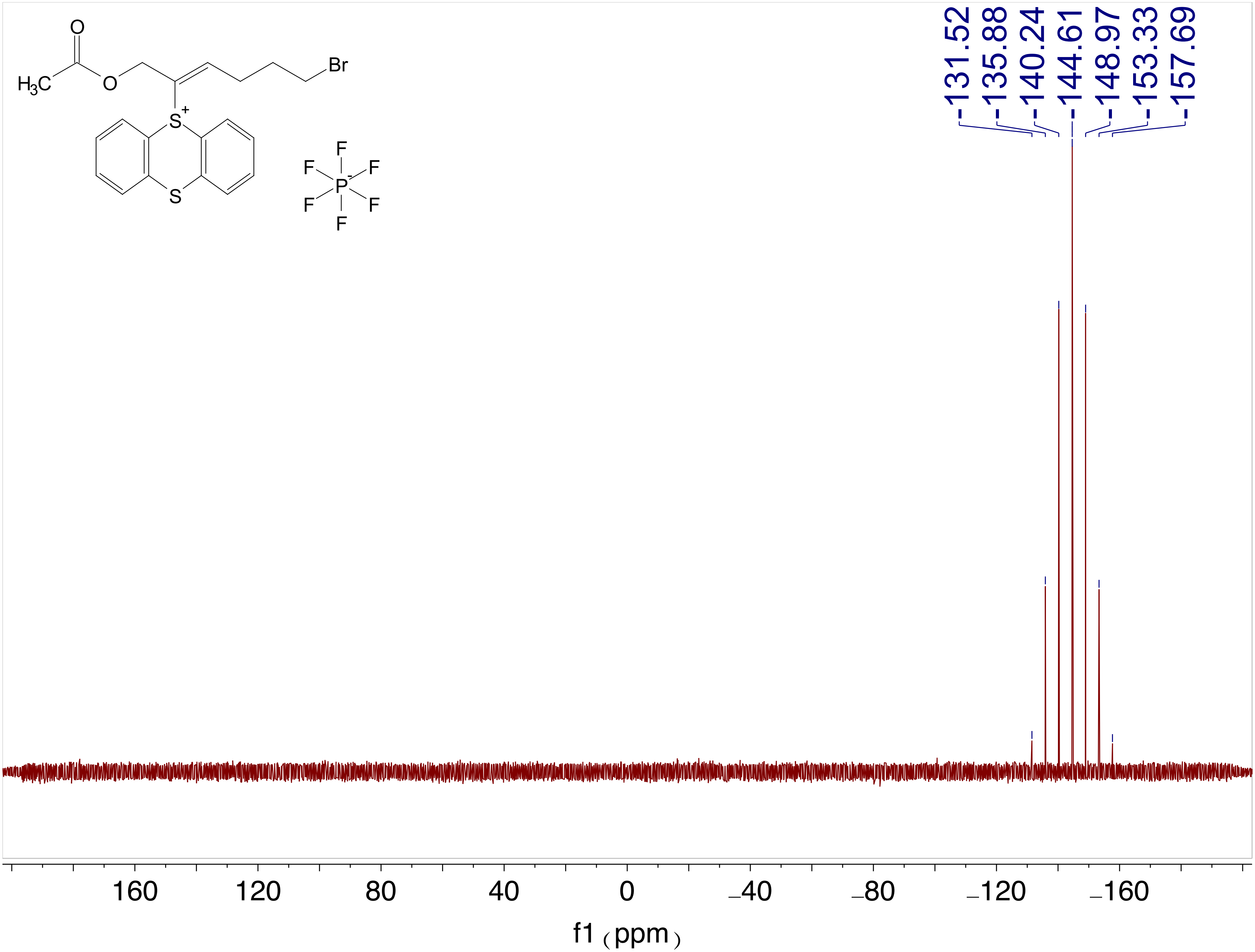

-131.52
-135.88
-140.24
-144.61
-148.97
-153.33
-157.69
160
120
80
40
0
–40
–80
–120
–160
f1 (ppm)

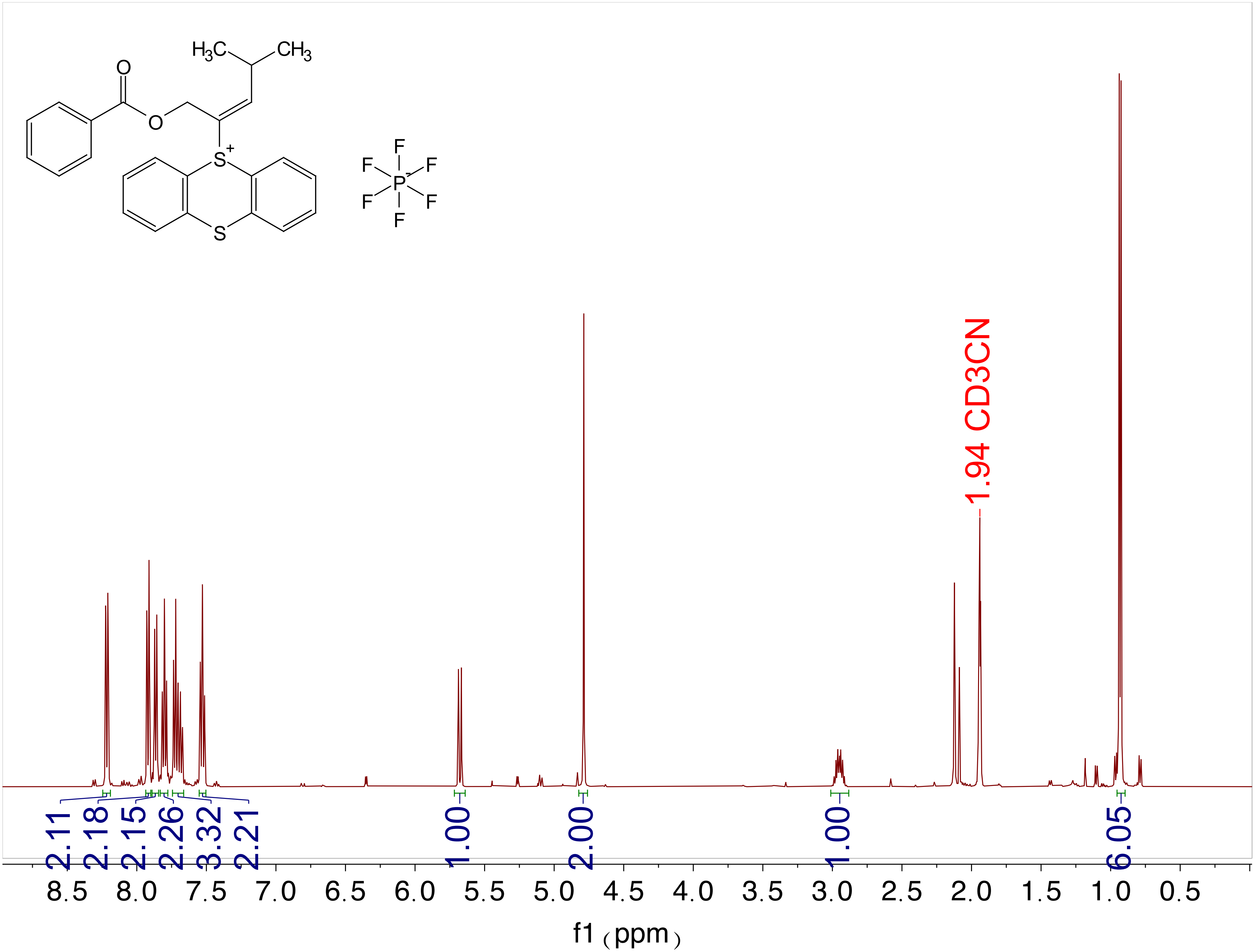

H3C
CH3
O
O
S+
S
F
P-
1.94 CD3CN
2.11
2.18
2.15
2.26
3.32
2.21
1.00
2.00
1.00
6.05
8.5 8.0 7.5 7.0 6.5 6.0 5.5 5.0 4.5 4.0 3.5 3.0 2.5 2.0 1.5 1.0 0.5
f1 (ppm)

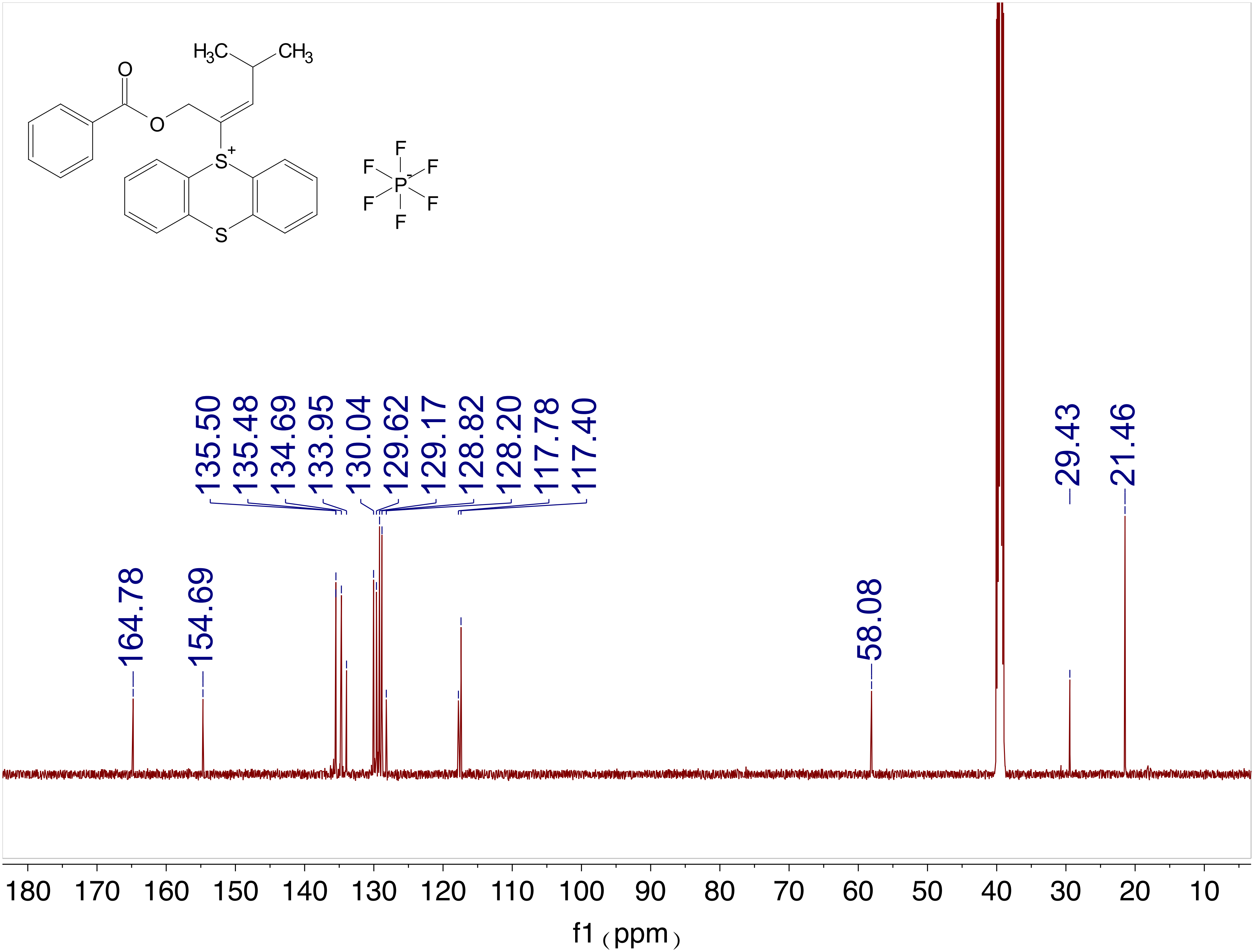

O
O
H3C
CH3
S+
S
F
F
F
P-
F
F
F
164.78
154.69
135.50
135.48
134.69
133.95
130.04
129.62
129.17
128.82
128.20
117.78
117.40
58.08
29.43
21.46
180
170
160
150
140
130
120
110
100
90
80
70
60
50
40
30
20
10
f1 (ppm)

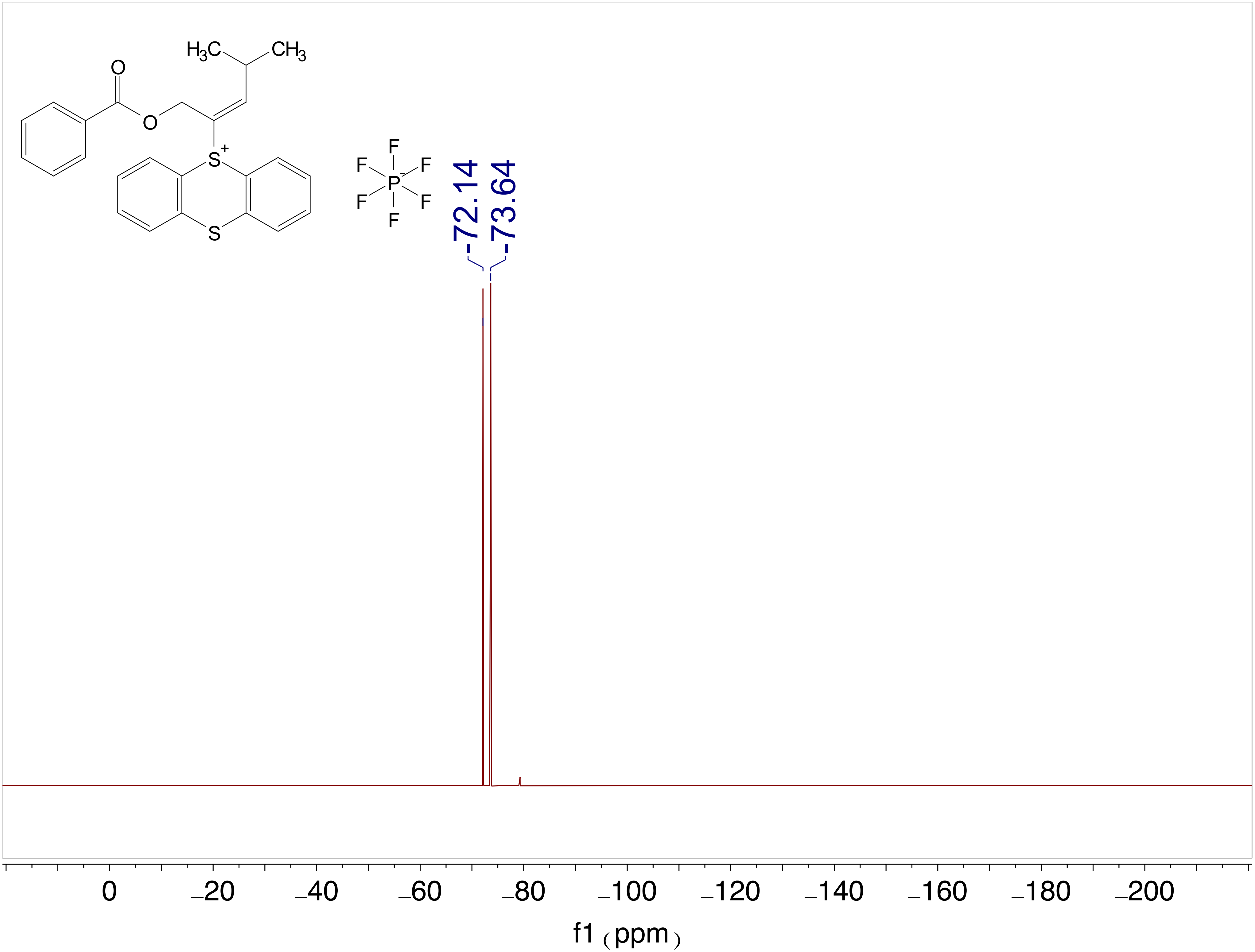

O
O
H3C
CH3
S+
S
F
F
F
P-
F
F
F
-72.14
-73.64
0
-20
-40
-60
-80
-100
-120
-140
-160
-180
-200
f1 (ppm)

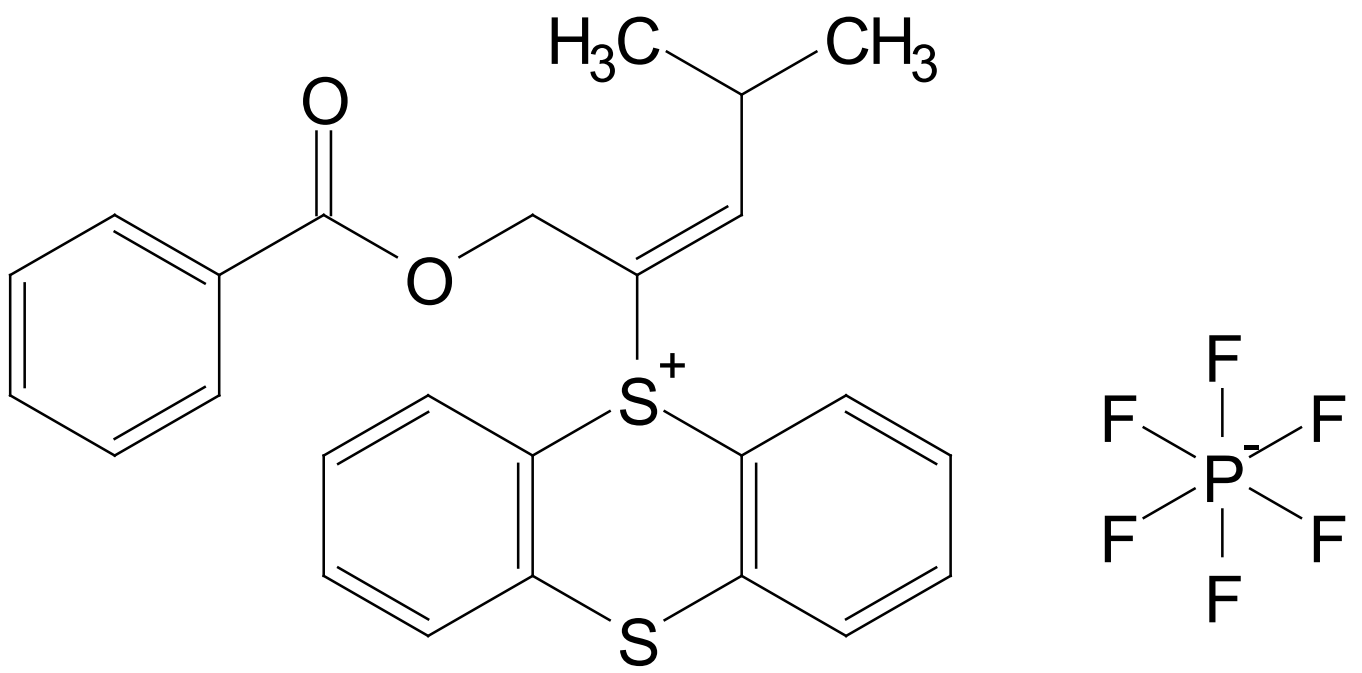

O
O
H3C
CH3
S+
S
F
F
F
P
F
F
F

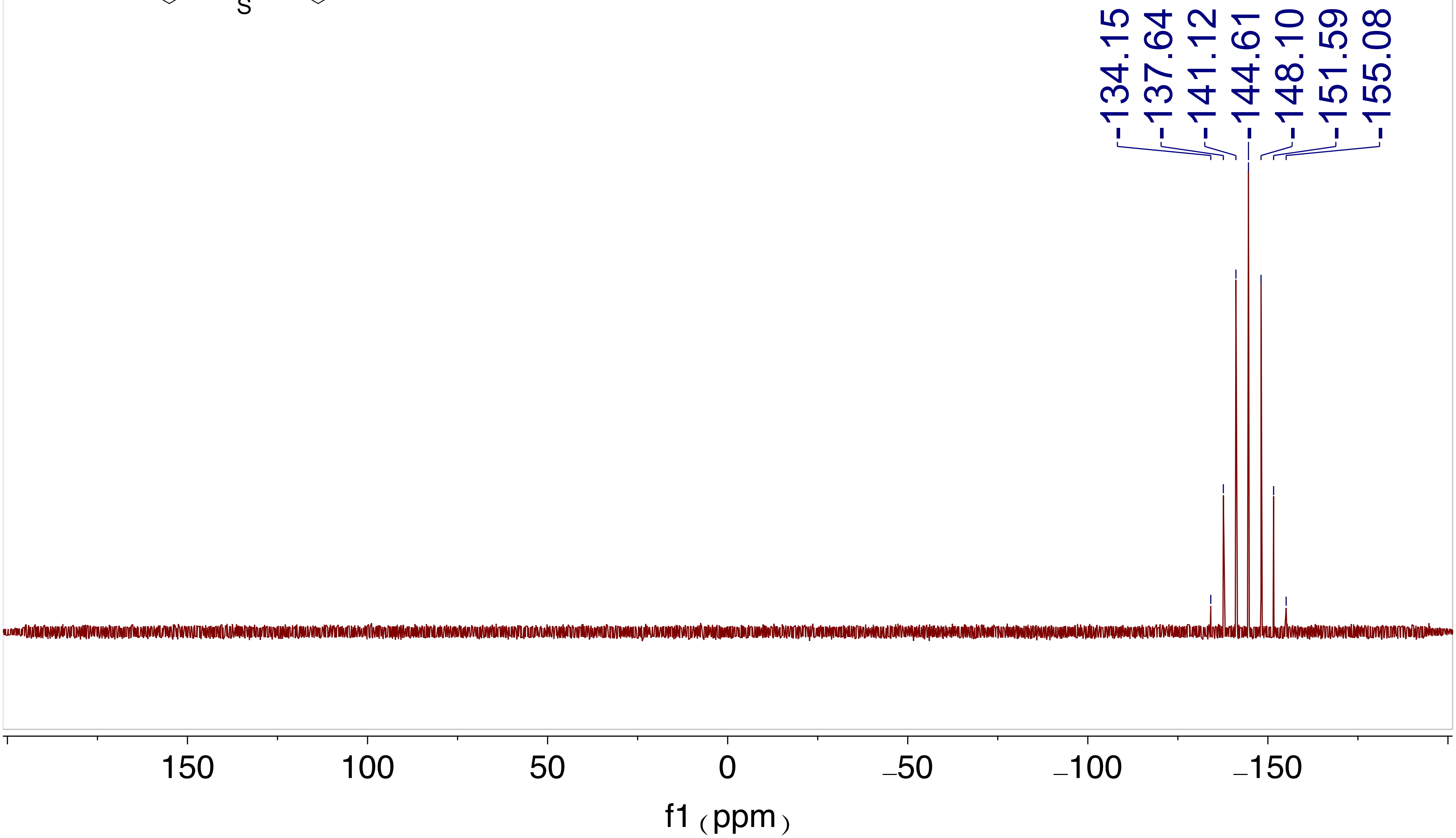

-134.15
-137.64
-141.12
-144.61
-148.10
-151.59
-155.08
150
100
50
0
–50
–100
–150
f1 (ppm)

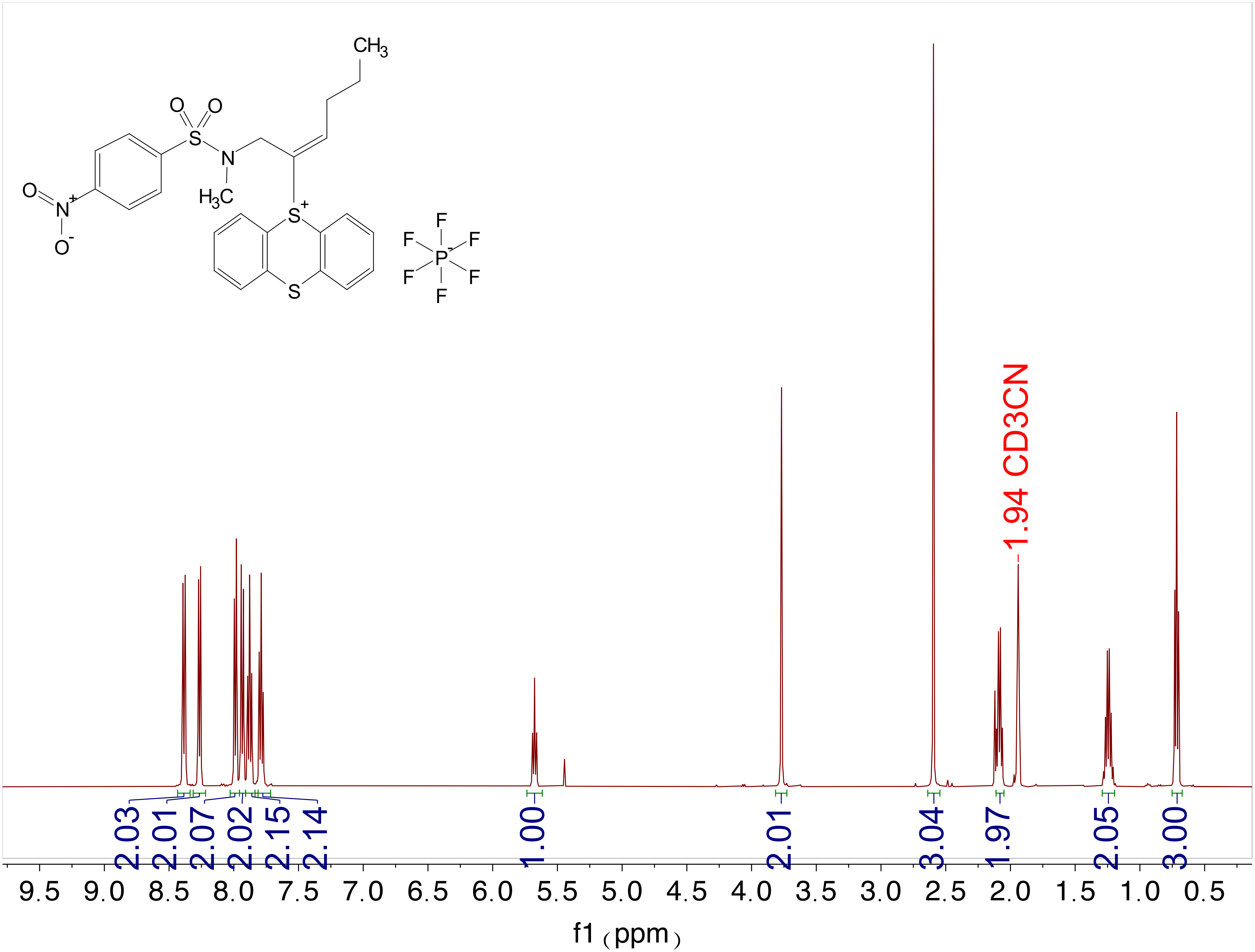

CH3
O
O
S
N
H3C
O
N+
O-
S+
S
F
F
F
P-
F
F
F
1.94 CD3CN
2.03
2.01
2.07
2.02
2.15
2.14
1.00
2.01
3.04
1.97
2.05
3.00
9.5
9.0
8.5
8.0
7.5
7.0
6.5
6.0
5.5
5.0
4.5
4.0
3.5
3.0
2.5
2.0
1.5
1.0
0.5
f1 (ppm)

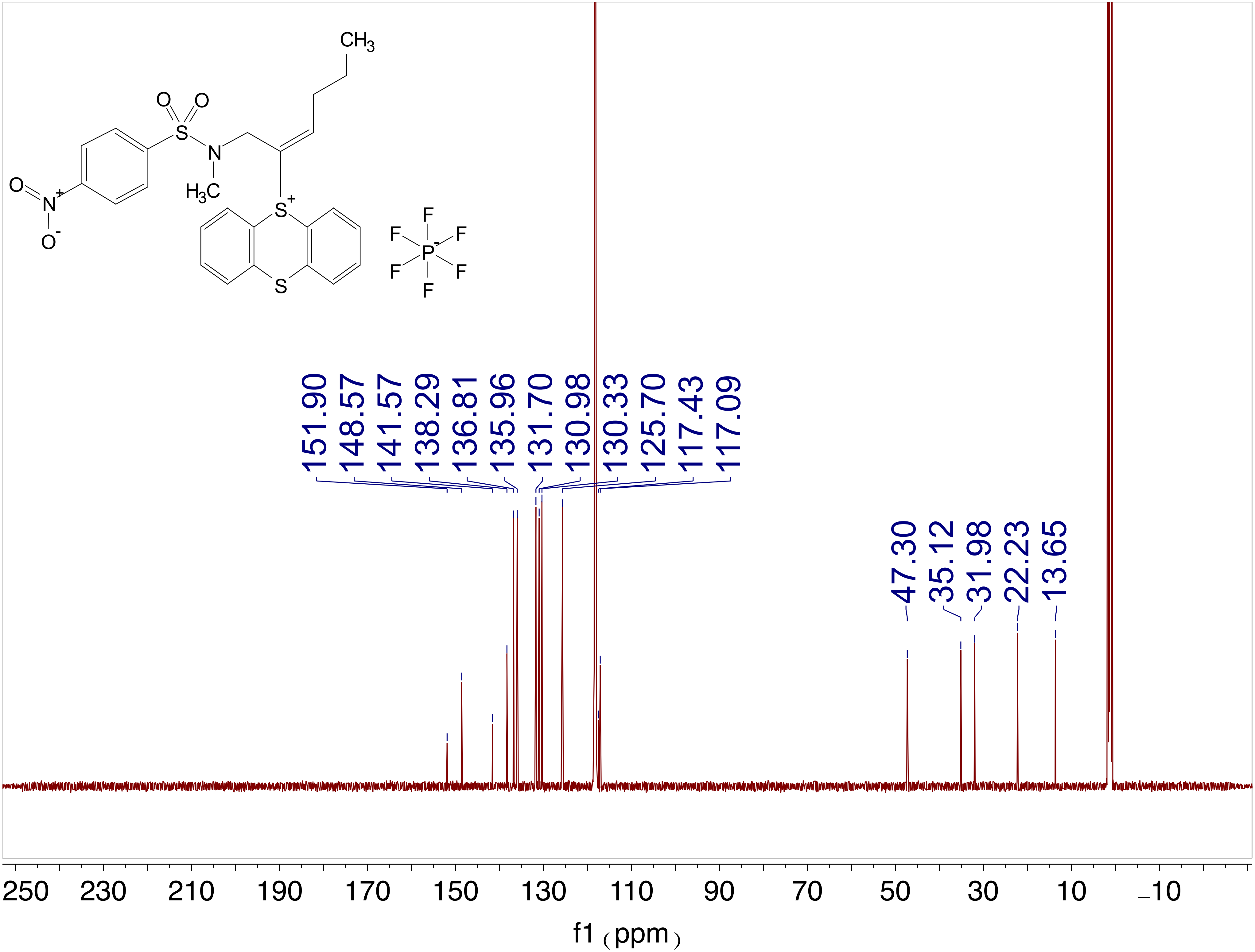

CH3
O
O
S
N
H3C
O
N+
O-
S+
S
F
F
F
P-
F
F
F
151.90
148.57
141.57
138.29
136.81
135.96
131.70
130.98
130.33
125.70
117.43
117.09
47.30
35.12
31.98
22.23
13.65
250
230
210
190
170
150
130
110
90
70
50
30
10
–10
f1 (ppm)

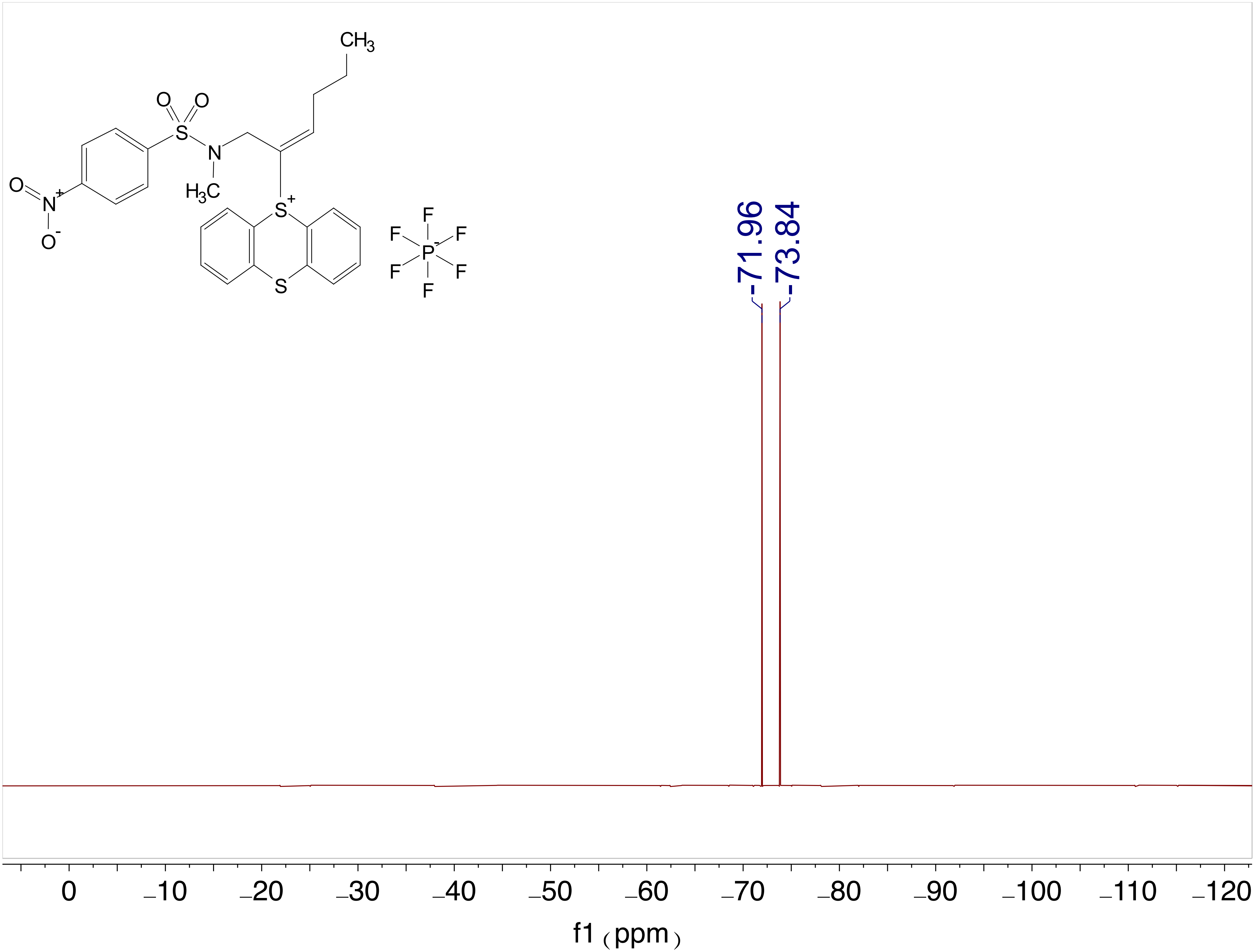

-71.96
-73.84
0
–10
–20
–30
–40
–50
–60
–70
–80
–90
–100
–110
–120
f1（ppm）

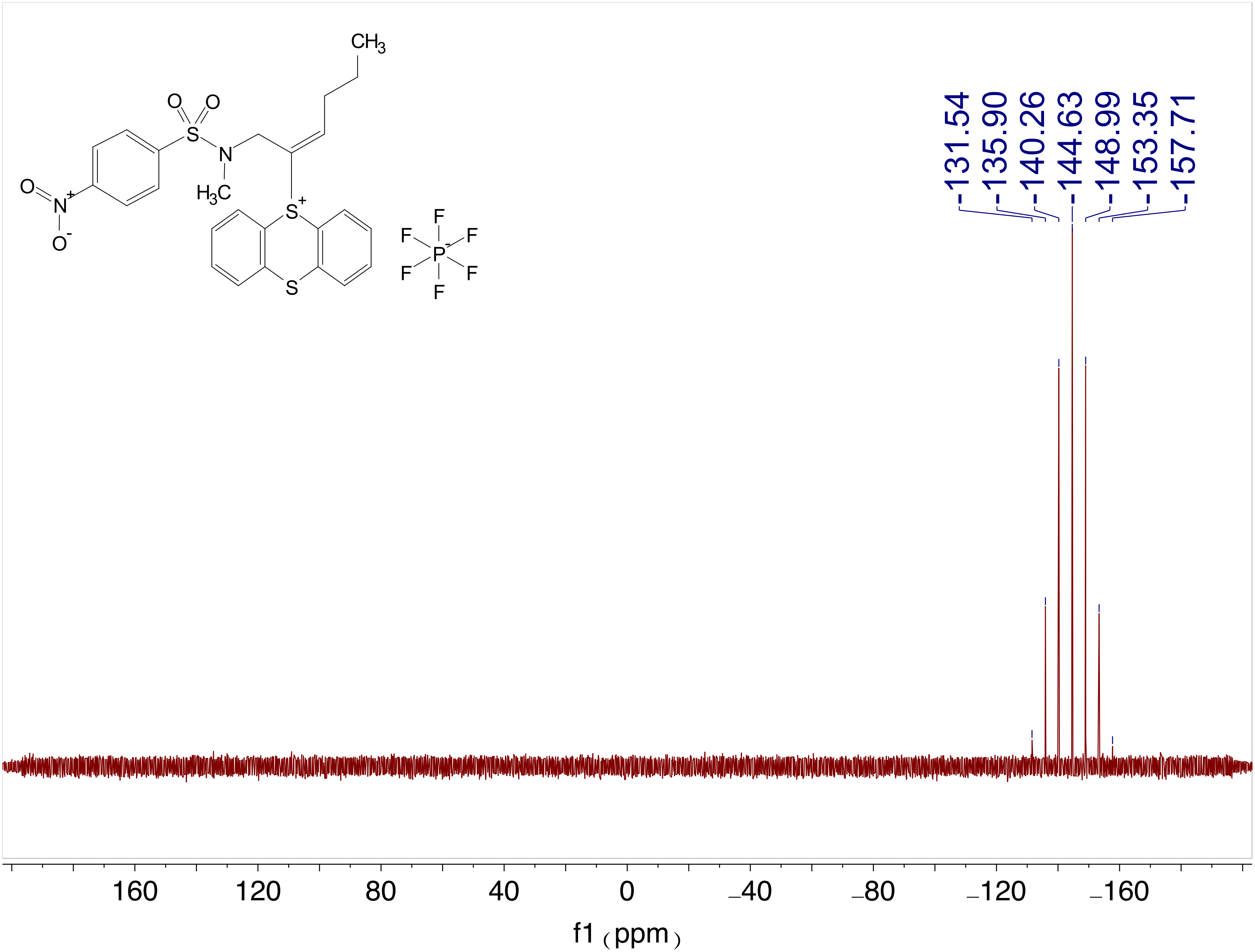

CH3
O
O
S
N
H3C
O
N+
O-
S+
S
F
F
F
P-
F
F
F
-131.54
-135.90
-140.26
-144.63
-148.99
-153.35
-157.71
160
120
80
40
0
–40
–80
–120
–160
f1 (ppm)

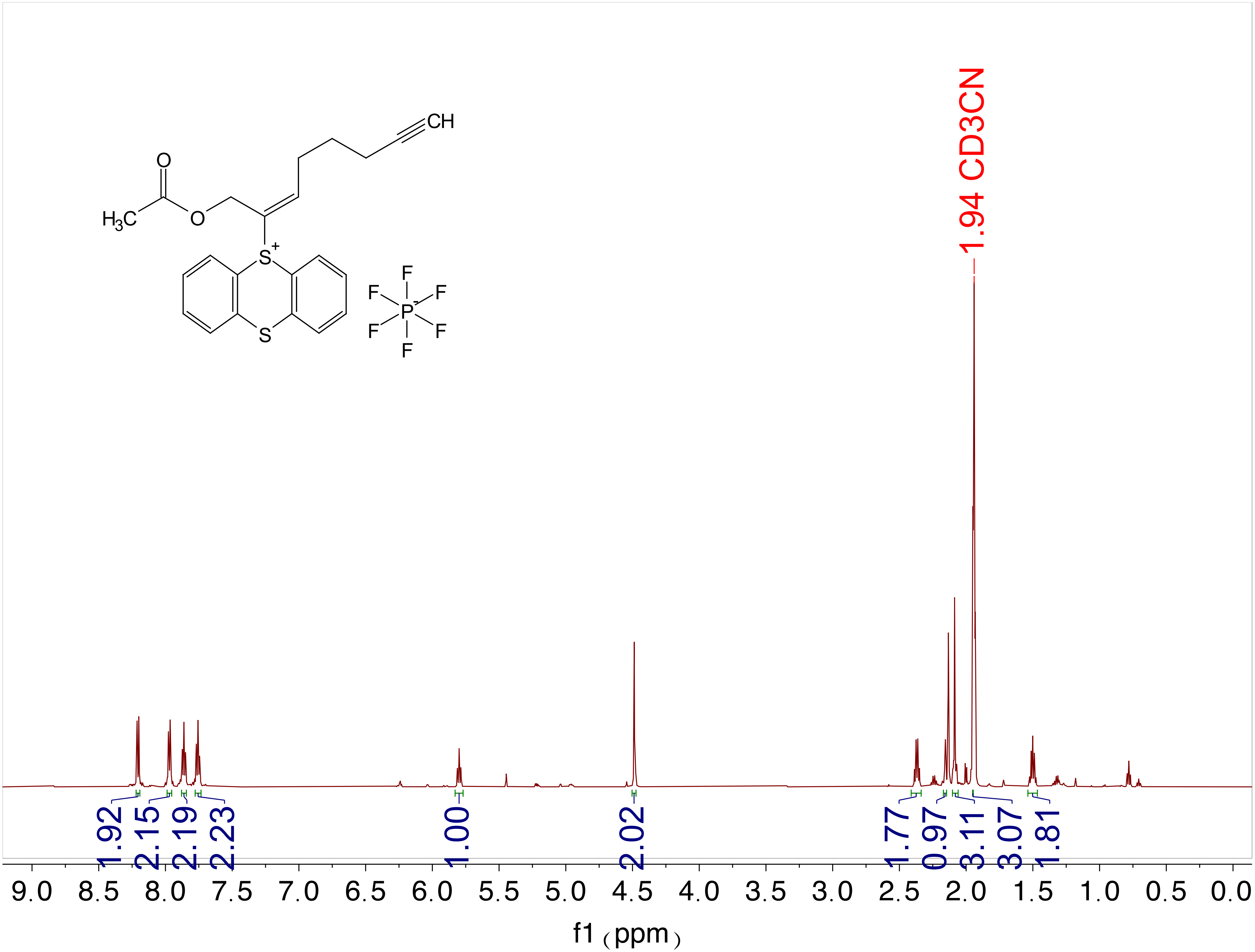

CH
O
H_3C
O
S^+
S
F
F
F
P^-
F
F
F
1.94 CD3CN
1.92
2.15
2.19
2.23
1.00
2.02
1.77
0.97
3.11
3.07
1.81
9.0
8.5
8.0
7.5
7.0
6.5
6.0
5.5
5.0
4.5
4.0
3.5
3.0
2.5
2.0
1.5
1.0
0.5
0.0
f1（ppm）

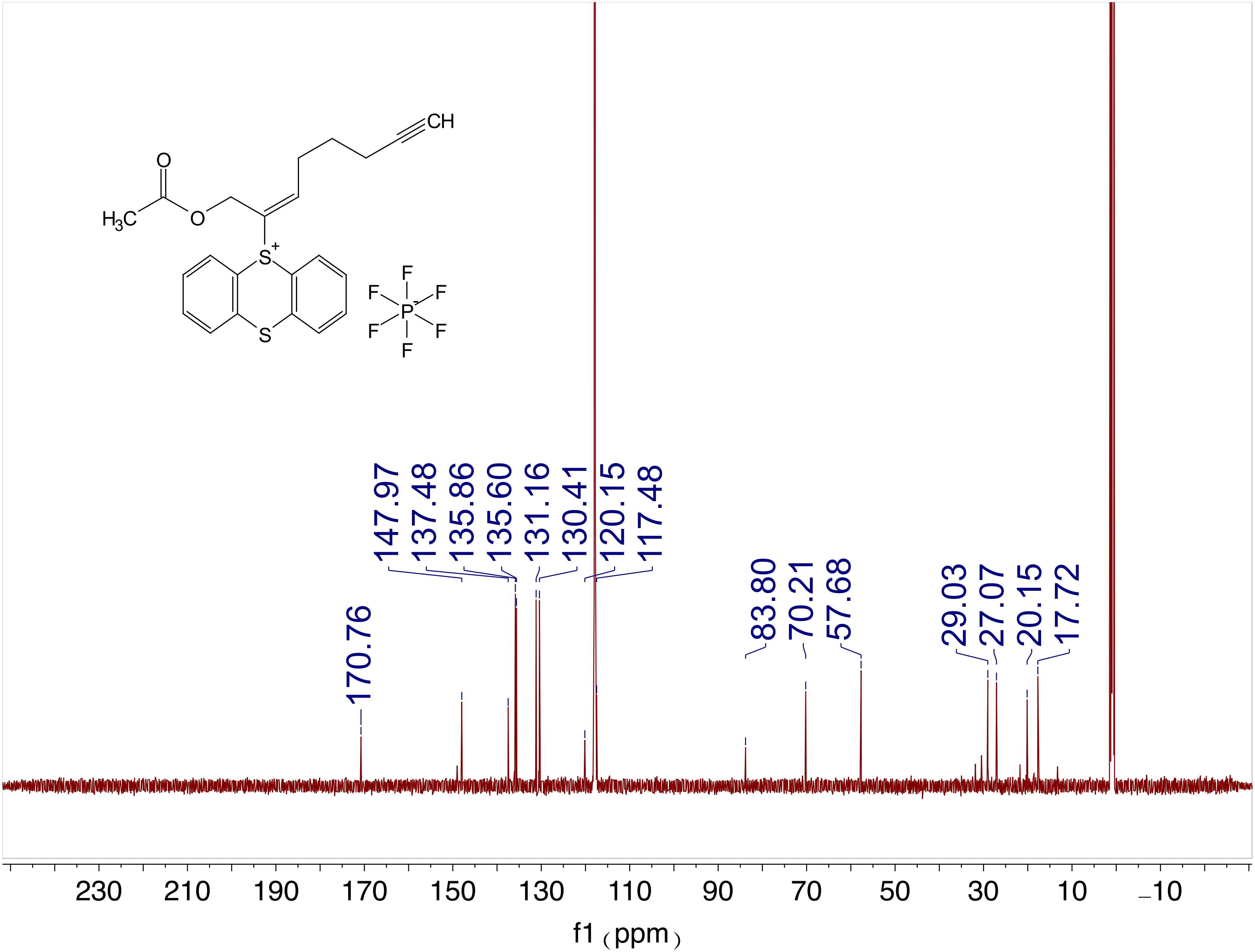

CH
O
H_3C
O
S^+
S
F
F
F
P^-
F
F
F
170.76
147.97
137.48
135.86
135.60
131.16
130.41
120.15
117.48
83.80
70.21
57.68
29.03
27.07
20.15
17.72
230
210
190
170
150
130
110
90
70
50
30
10
–10
f1 (ppm)

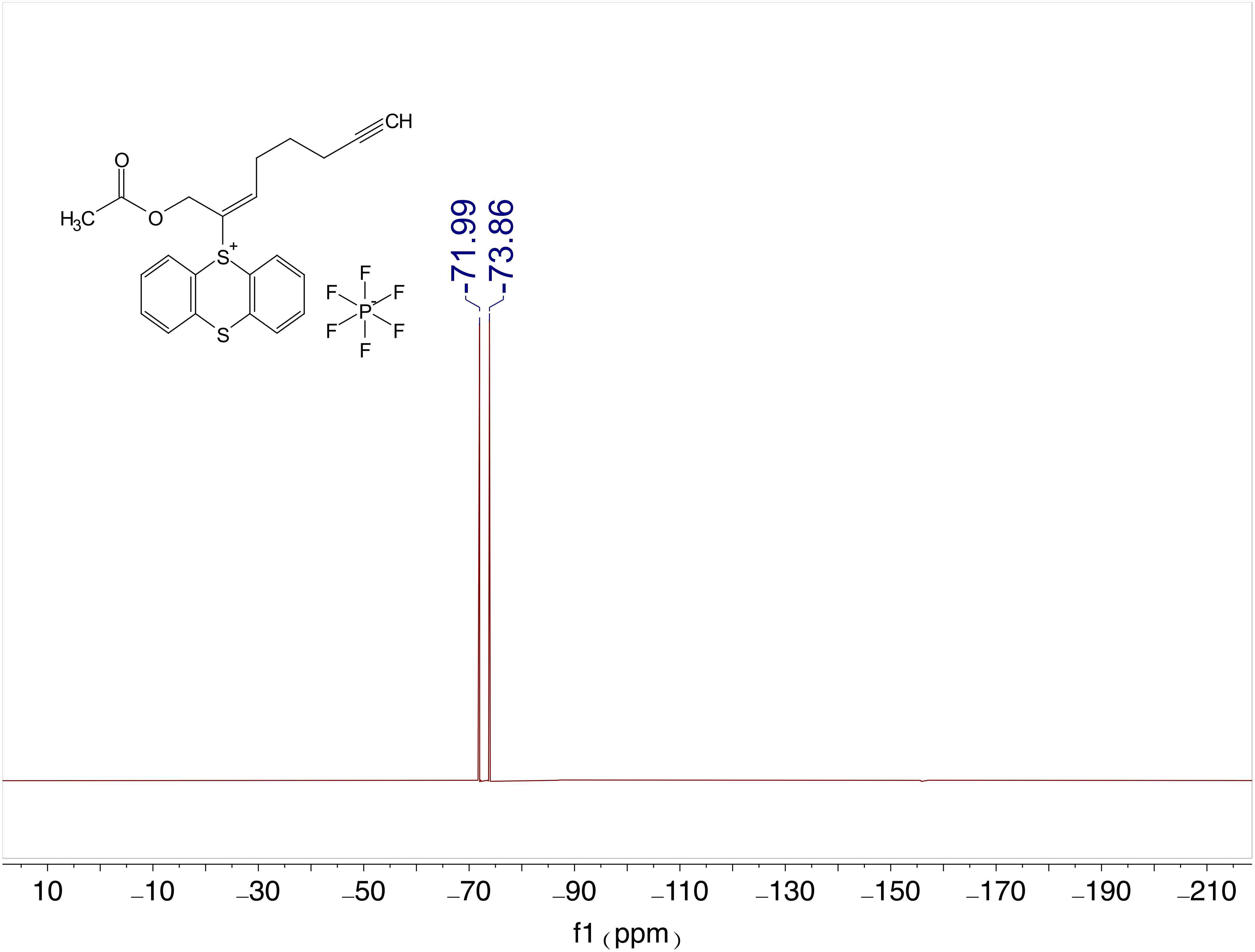

CH
O
H_3C
O
S+
S
F
F
F
P-
F
F
F
-71.99
-73.86
10
–10
–30
–50
–70
–90
–110
–130
–150
–170
–190
–210
f1（ppm）

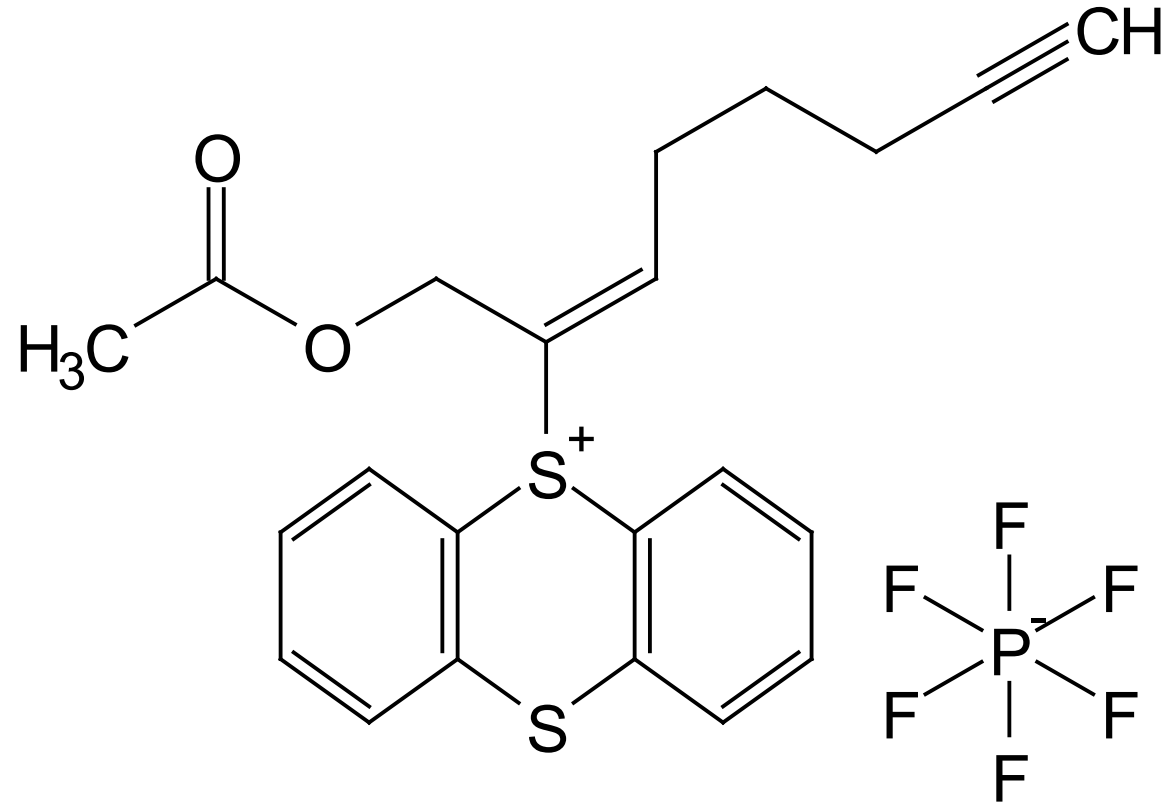

CH
O
H_3C
O
S+
S
F
F
F
P-
F
F
F

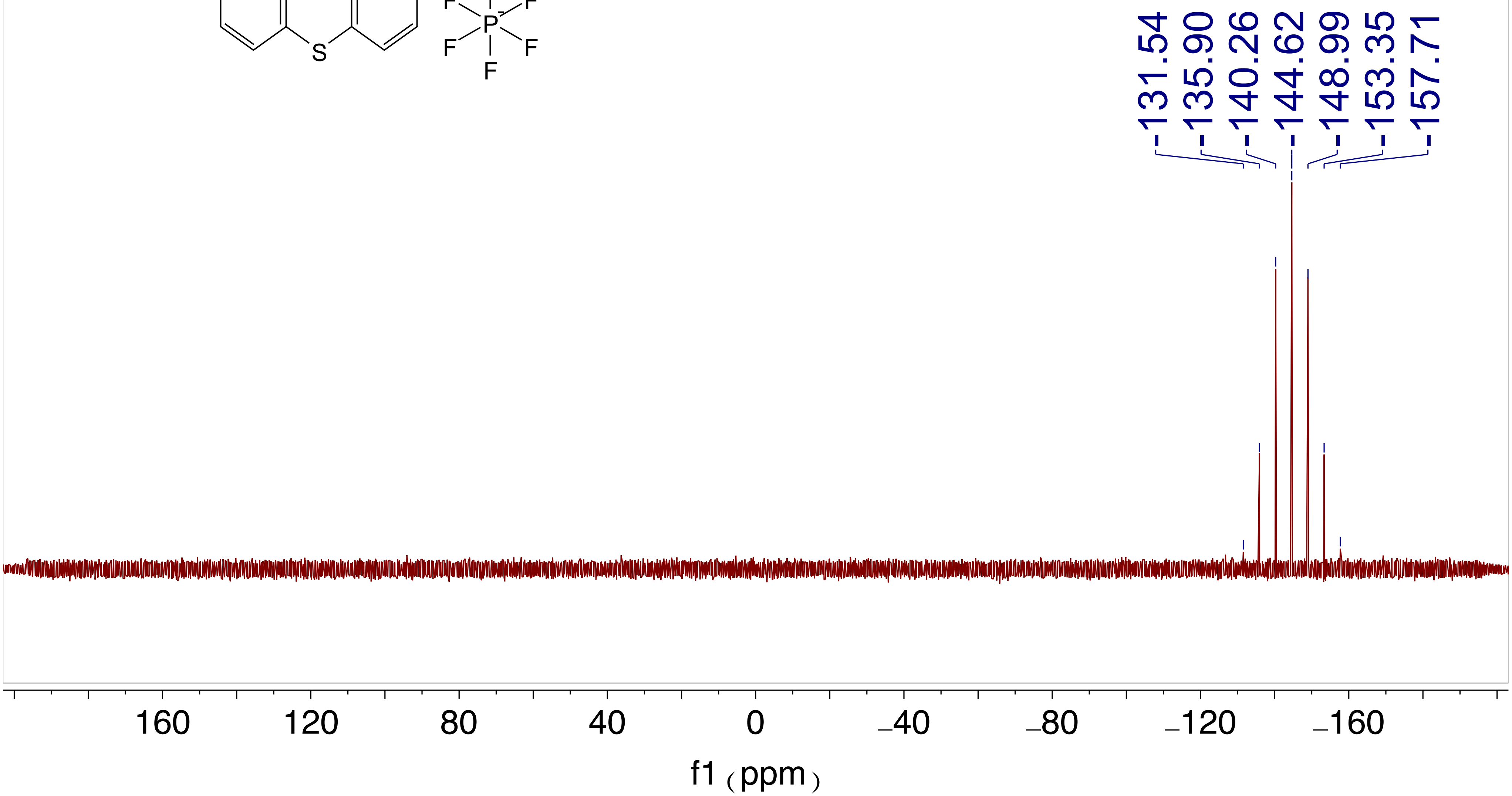

-131.54
-135.90
-140.26
-144.62
-148.99
-153.35
-157.71
160
120
80
40
0
–40
–80
–120
–160
f1 (ppm)

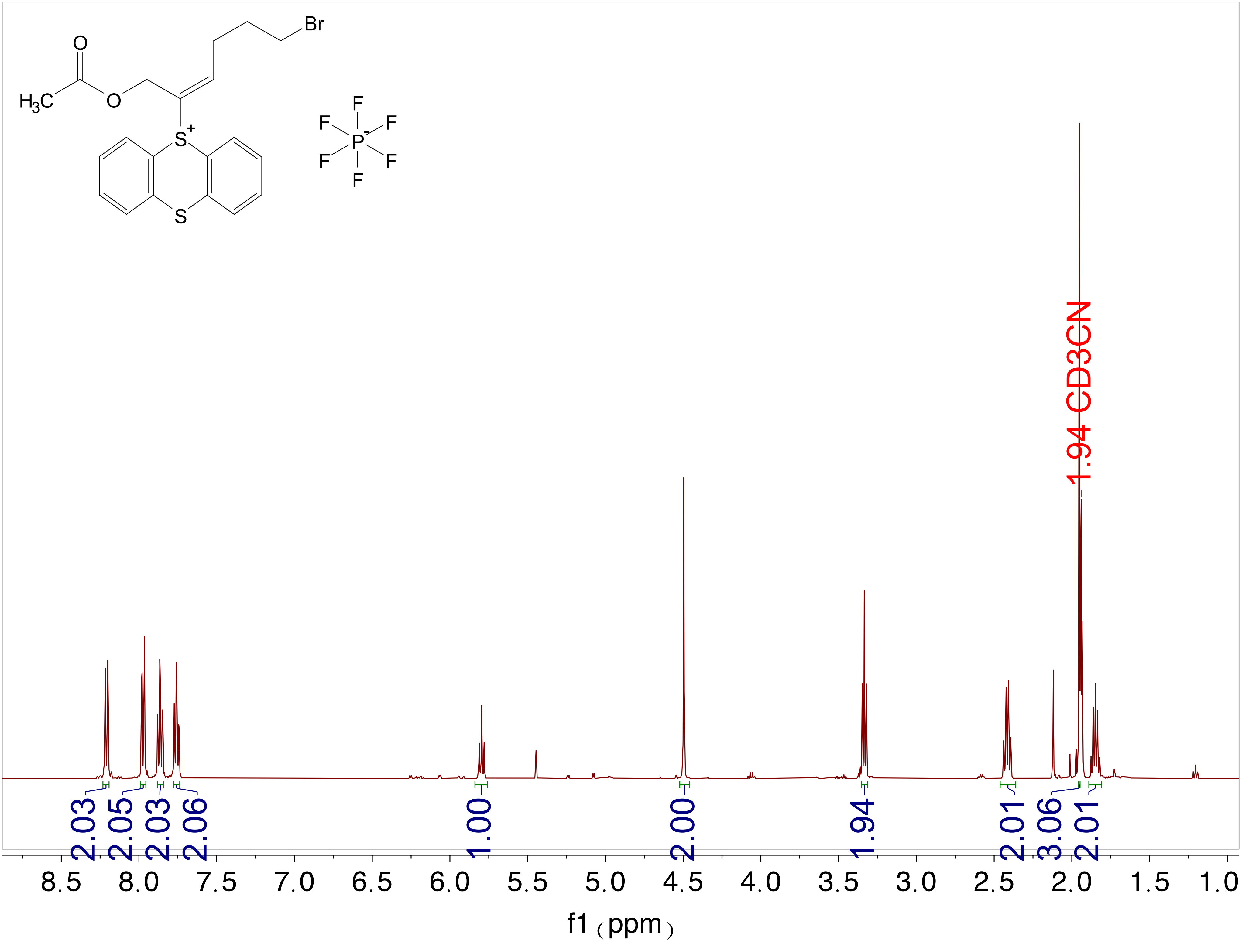
Br
O
H3C
O
S+
S
F
F
F
P-
F
F
F
1.94 CD3CN
2.03
2.05
2.03
2.06
1.00
2.00
1.94
2.01
3.06
2.01
8.5
8.0
7.5
7.0
6.5
6.0
5.5
5.0
4.5
4.0
3.5
3.0
2.5
2.0
1.5
1.0
f1（ppm）

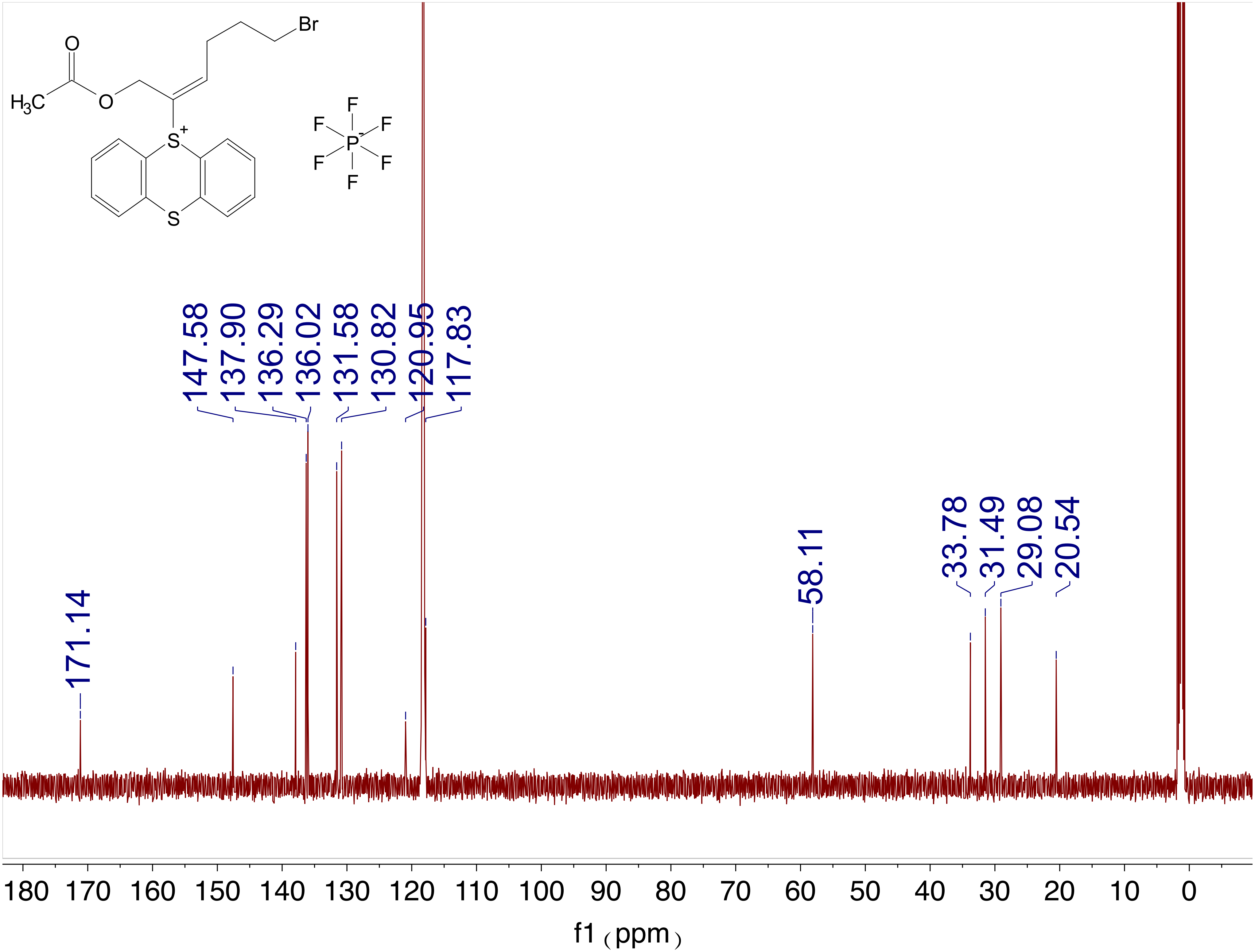

Br
O
H_3C
O
S^+
S
F
F
F
P^-
F
F
F
171.14
147.58
137.90
136.29
136.02
131.58
130.82
120.95
117.83
58.11
33.78
31.49
29.08
20.54
180 170 160 150 140 130 120 110 100 90 80 70 60 50 40 30 20 10 0
f1（ppm）

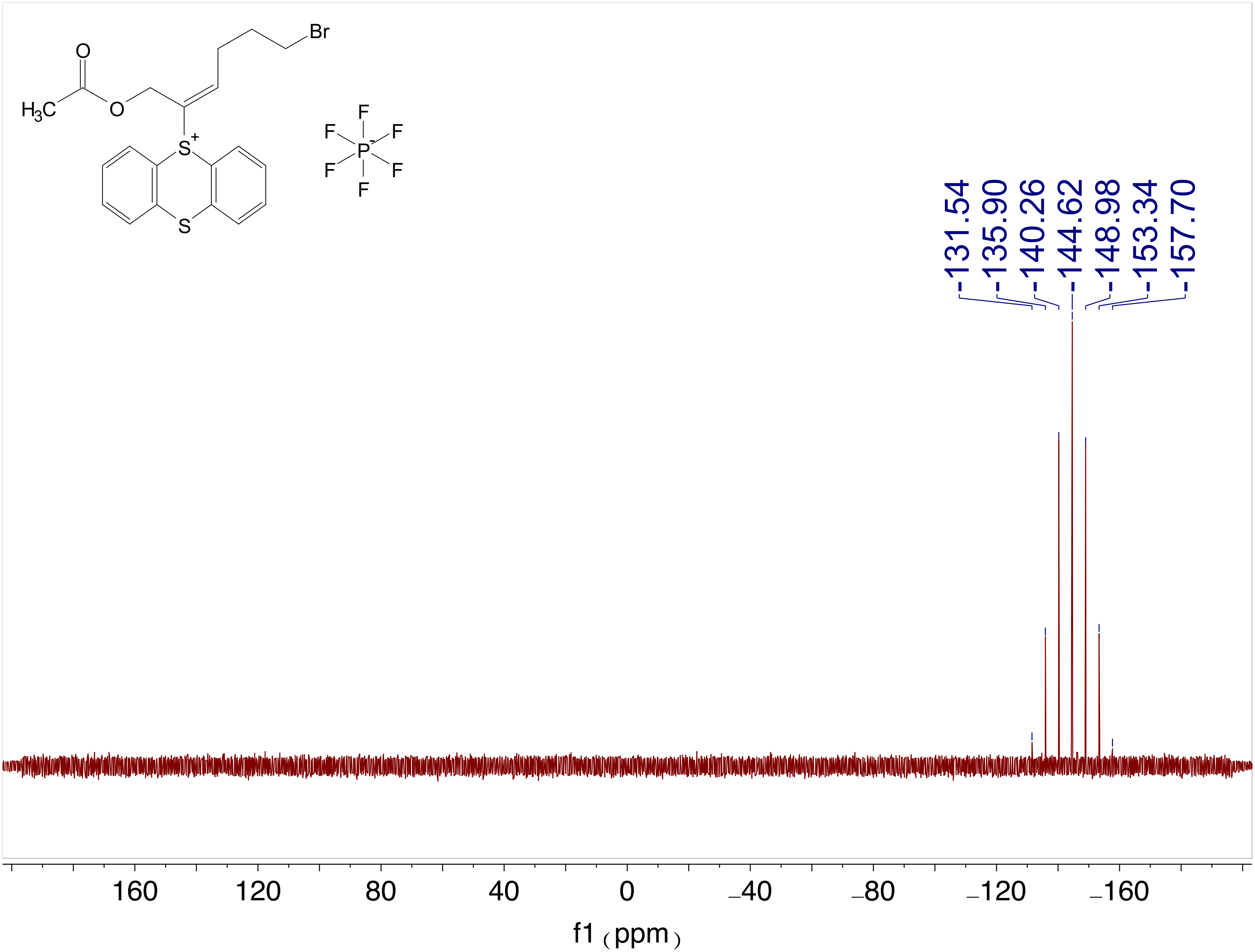

Br
O
H3C
O
S+
S
F
F
F
P-
F
F
F
-131.54
-135.90
-140.26
-144.62
-148.98
-153.34
-157.70
160
120
80
40
0
-40
-80
-120
-160
f1 (ppm)

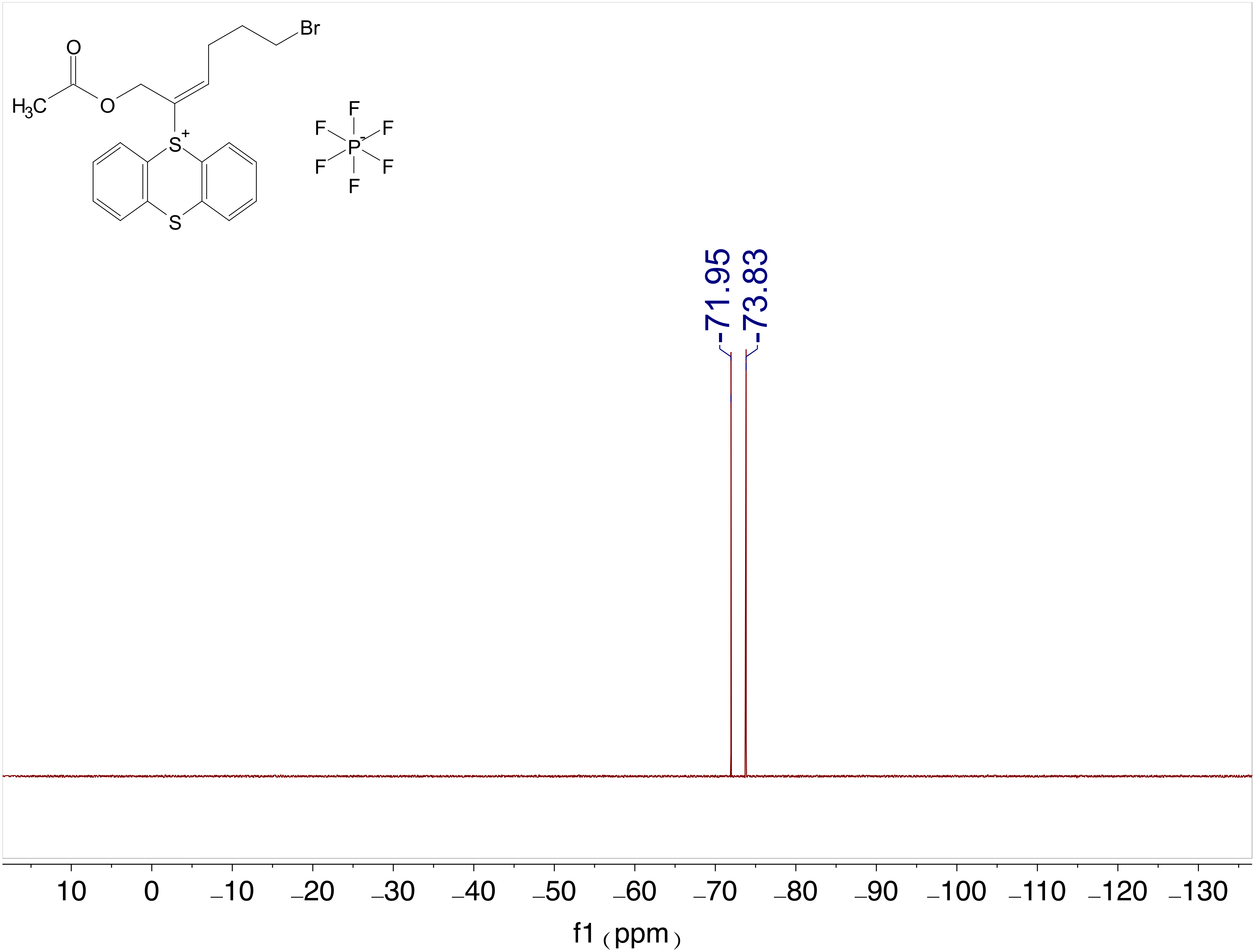

Br
O
H_3C
O
S^+
S
F
F
F
P^-
F
F
F
-71.95
-73.83
10
0
–10
–20
–30
–40
–50
–60
–70
–80
–90
–100
–110
–120
–130
f1 (ppm)

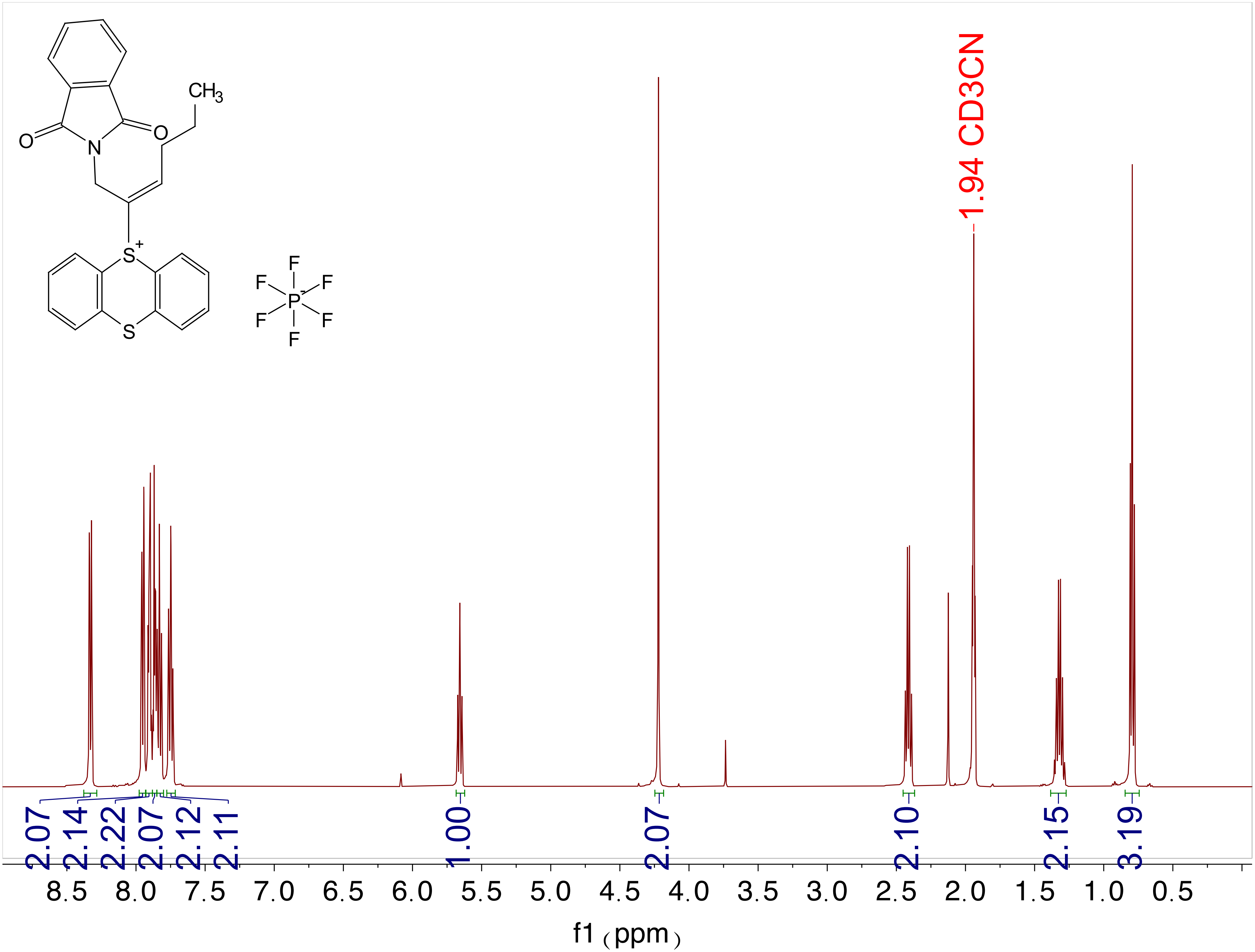
CH3
O
N
O
S+
S
F
F
P−
F
F
F
F
1.94 CD3CN
2.07
2.14
2.22
2.07
2.12
2.11
1.00
2.07
2.10
2.15
3.19
8.5
8.0
7.5
7.0
6.5
6.0
5.5
5.0
4.5
4.0
3.5
3.0
2.5
2.0
1.5
1.0
0.5
f1 (ppm)

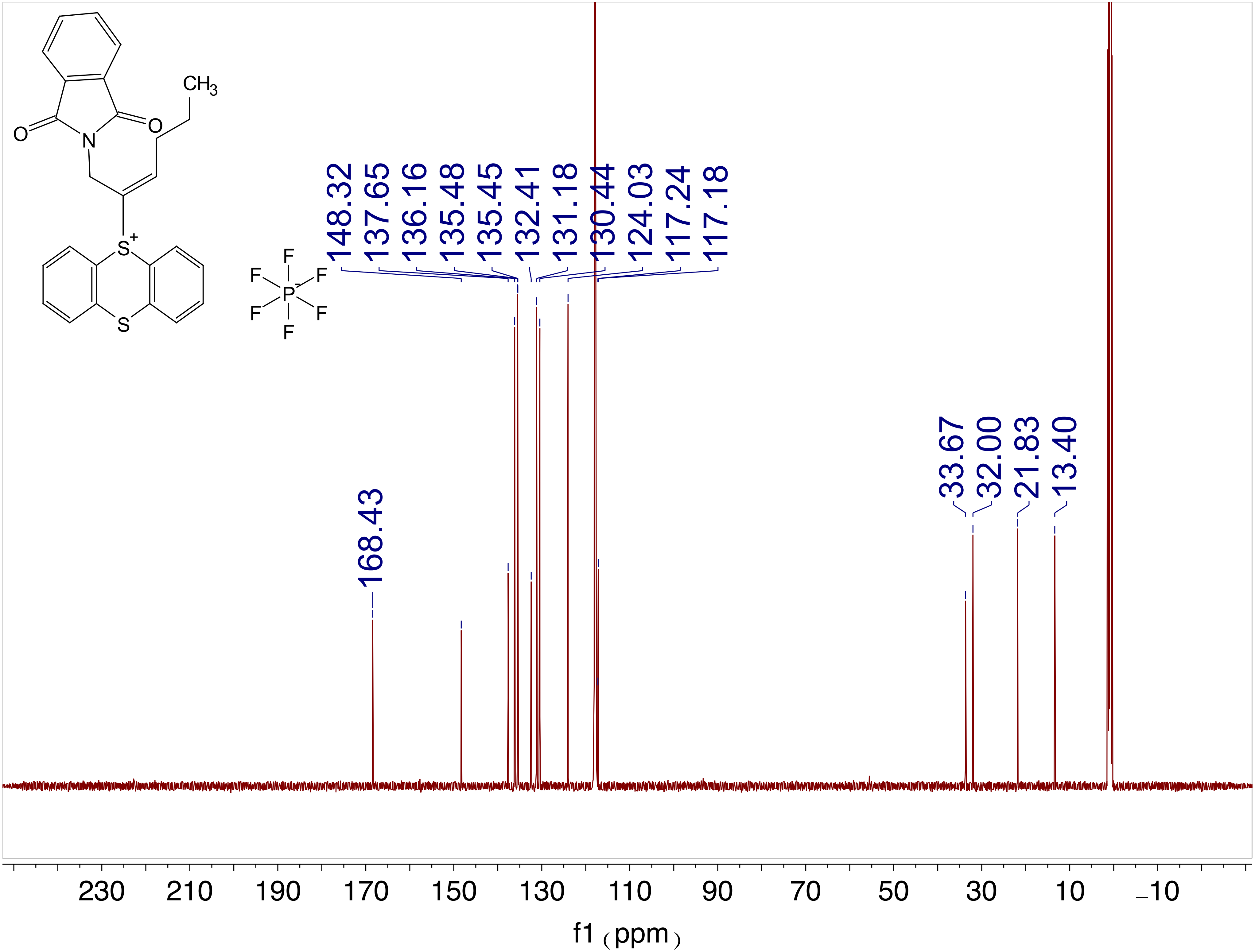
CH3
O
N
O
S+
S
F
F
F
P-
F
F
F
148.32
137.65
136.16
135.48
135.45
132.41
131.18
130.44
124.03
117.24
117.18
168.43
33.67
32.00
21.83
13.40
230
210
190
170
150
130
110
90
70
50
30
10
–10
f1 (ppm)

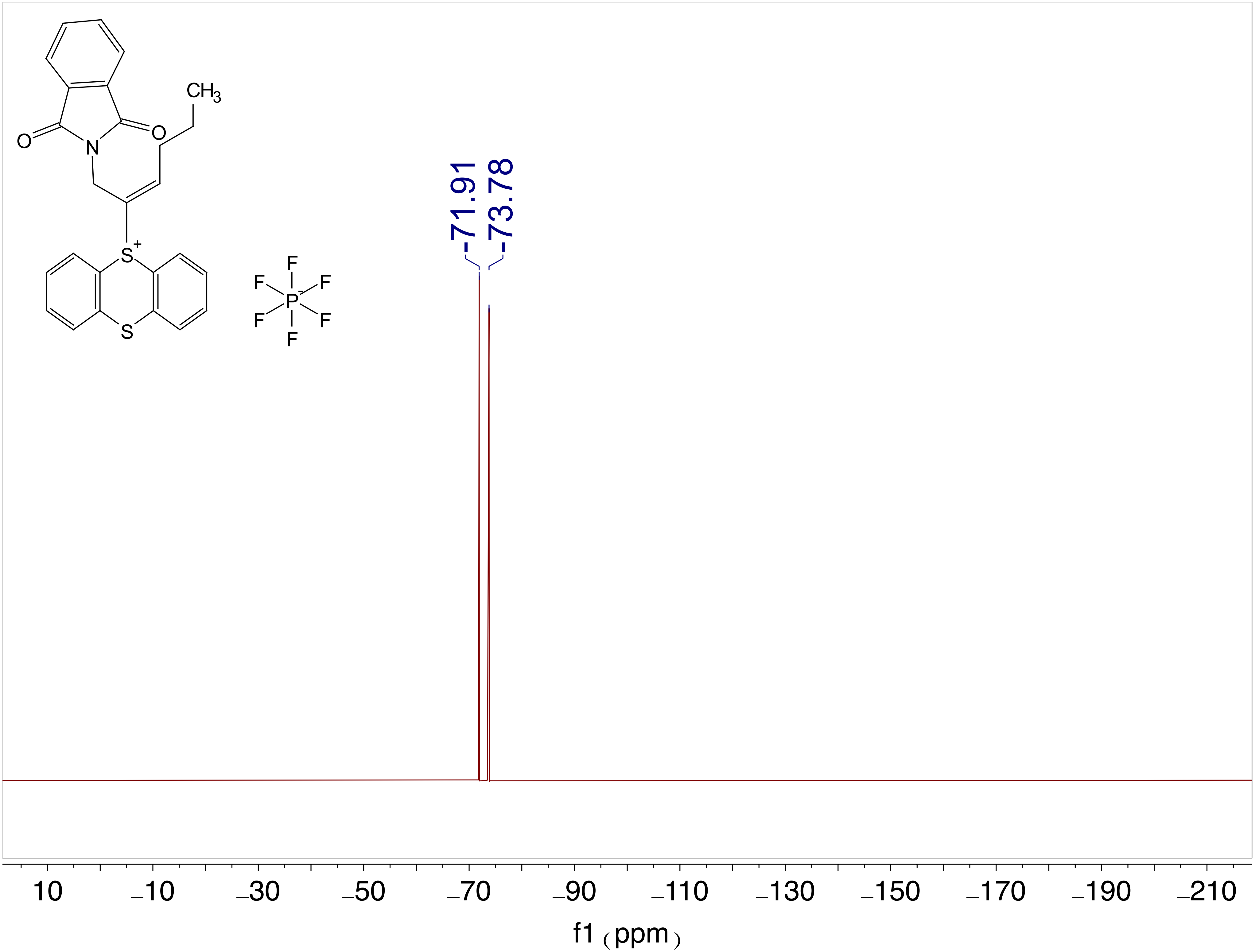

CH3
O
O
N
S+
S
F
F
F
P-
F
F
F
-71.91
-73.78
10
–10
–30
–50
–70
–90
–110
–130
–150
–170
–190
–210
f1（ppm）

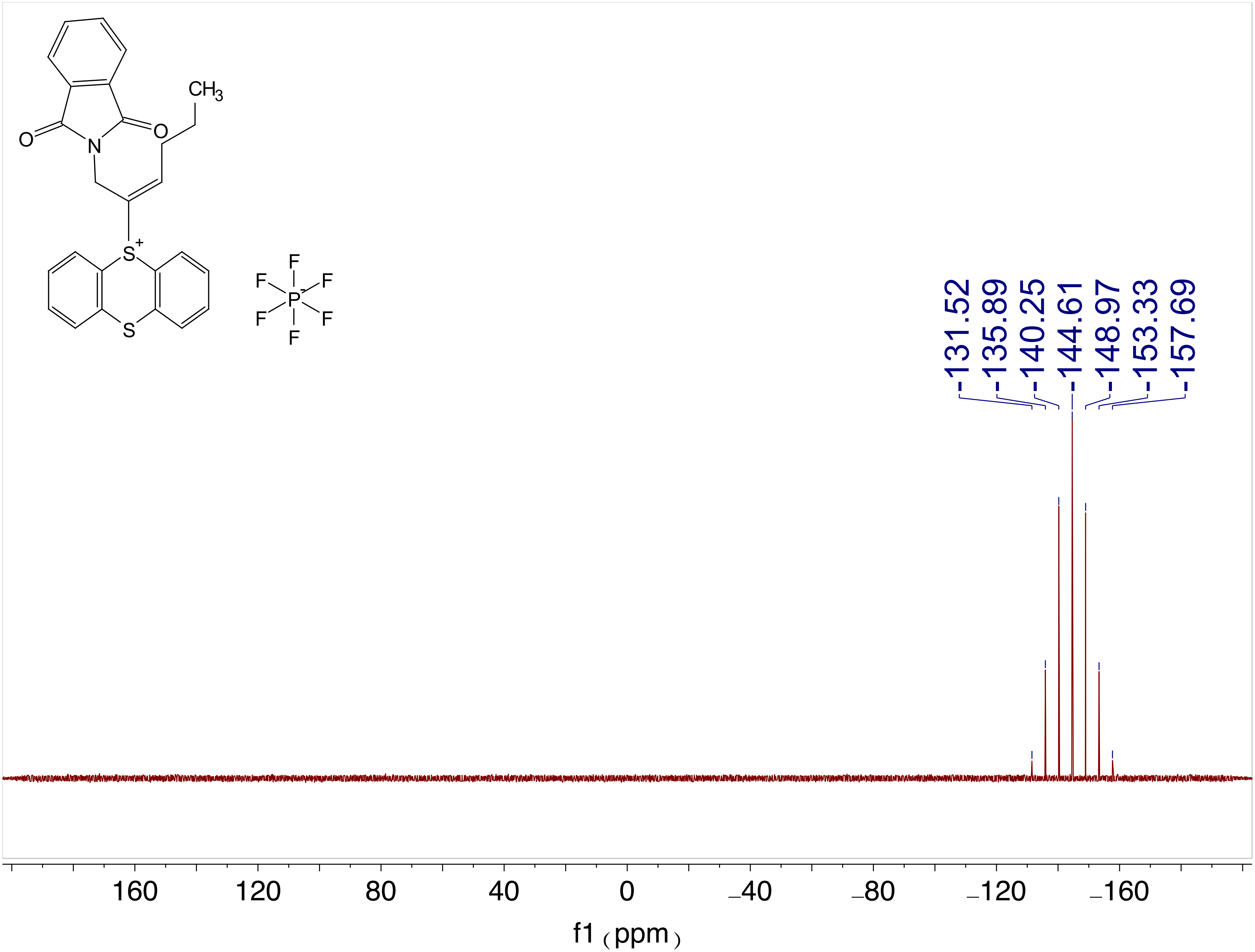

CH3
O
N
O
S+
S
F
F
P
F
F
F
F
-131.52
-135.89
-140.25
-144.61
-148.97
-153.33
-157.69
160
120
80
40
0
–40
–80
–120
–160
f1 (ppm)

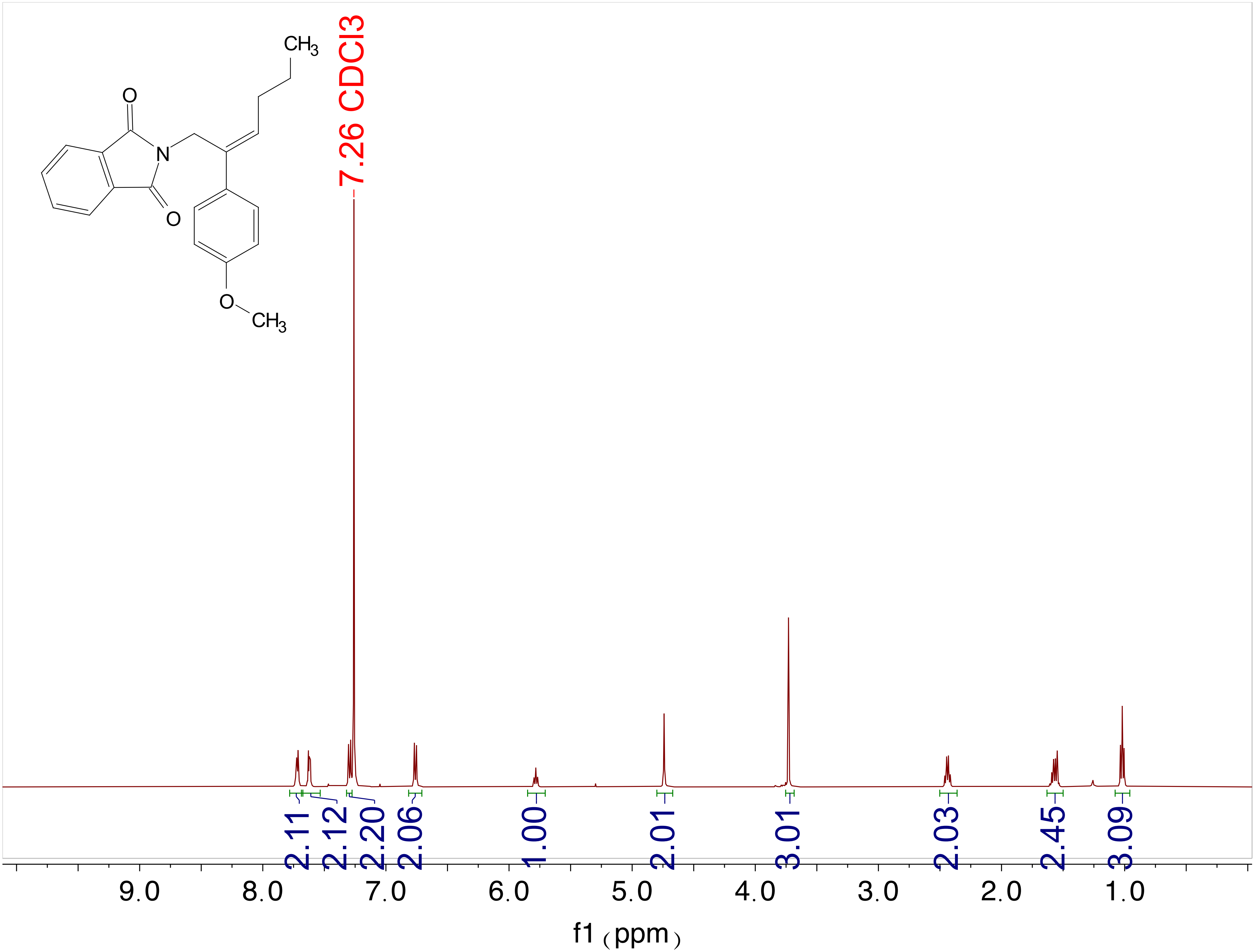
7.26 CDCl3
2.11
2.12
2.20
2.06
1.00
2.01
3.01
2.03
2.45
3.09
9.0
8.0
7.0
6.0
5.0
4.0
3.0
2.0
1.0
f1 (ppm)
CH3
O
N
O
O
CH3

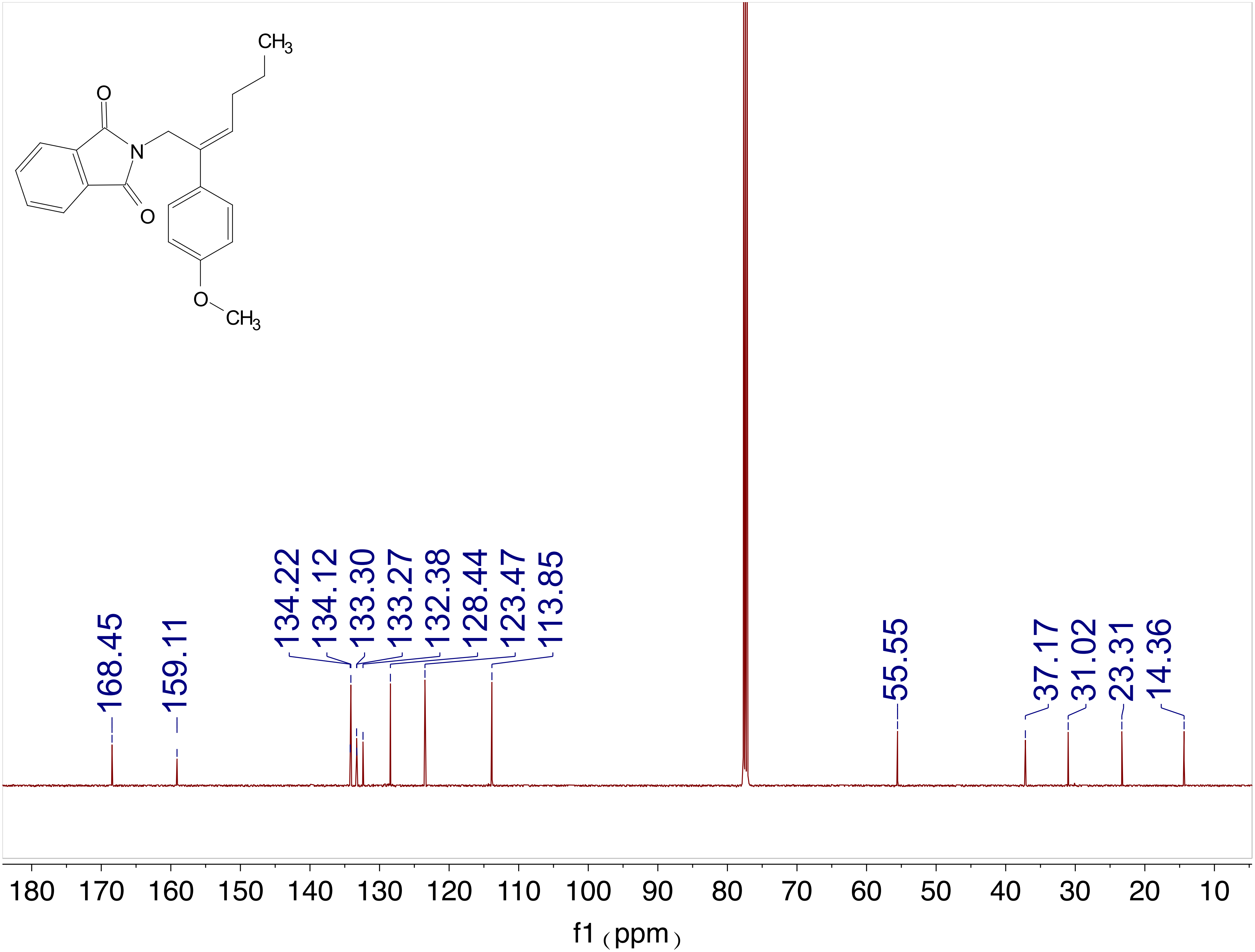
CH3
O
N
O
O
CH3
168.45
159.11
134.22
134.12
133.30
133.27
132.38
128.44
123.47
113.85
55.55
37.17
31.02
23.31
14.36
180 170 160 150 140 130 120 110 100 90 80 70 60 50 40 30 20 10
f1（ppm）

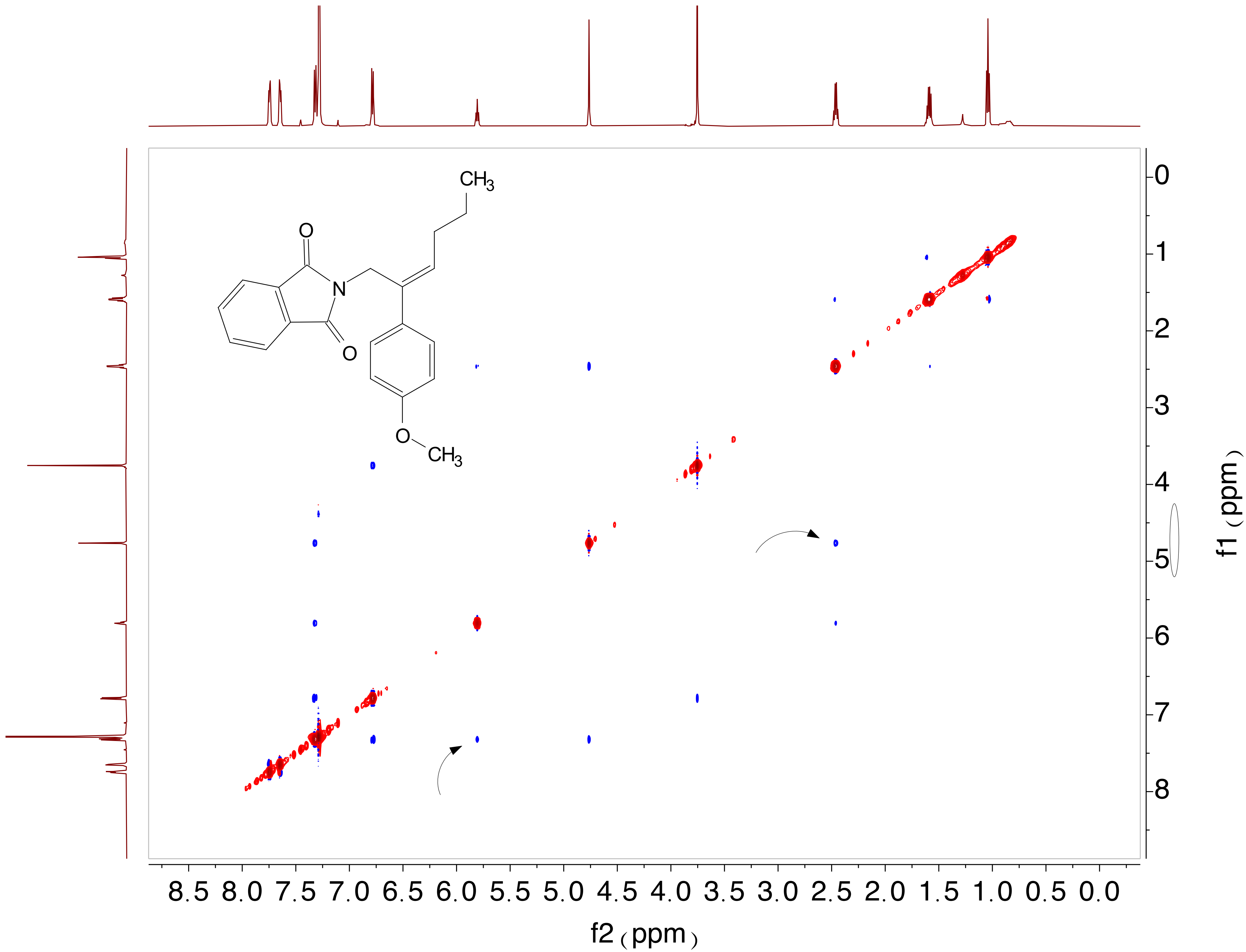

CH3
O
N
O
O
CH3
0
1
2
3
4
5
6
7
8
f1 (ppm)
8.5 8.0 7.5 7.0 6.5 6.0 5.5 5.0 4.5 4.0 3.5 3.0 2.5 2.0 1.5 1.0 0.5 0.0
f2 (ppm)

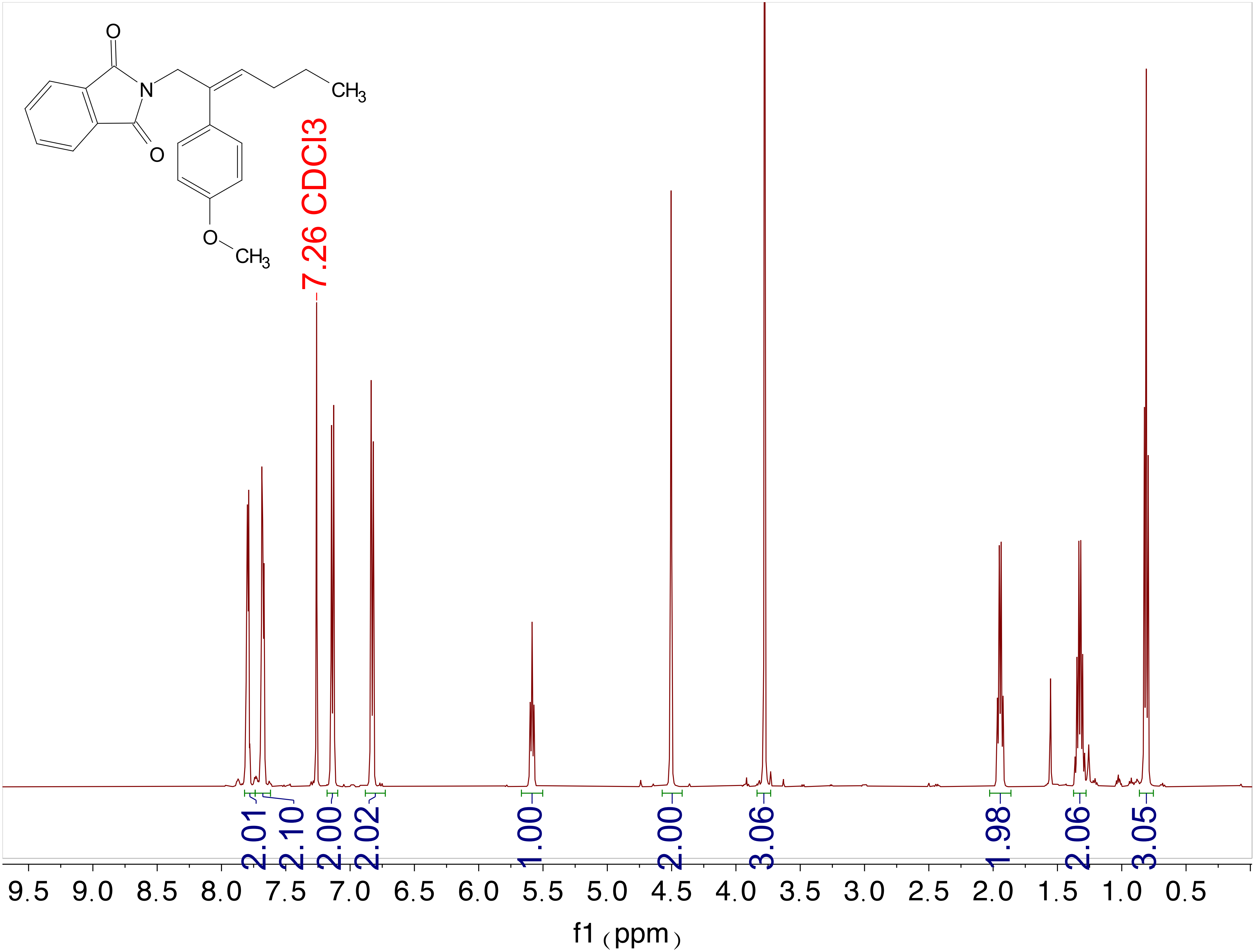

O
N
O
CH3
O
CH3
7.26 CDCl3
2.01
2.10
2.00
2.02
1.00
2.00
3.06
1.98
2.06
3.05
9.5 9.0 8.5 8.0 7.5 7.0 6.5 6.0 5.5 5.0 4.5 4.0 3.5 3.0 2.5 2.0 1.5 1.0 0.5
f1 (ppm)

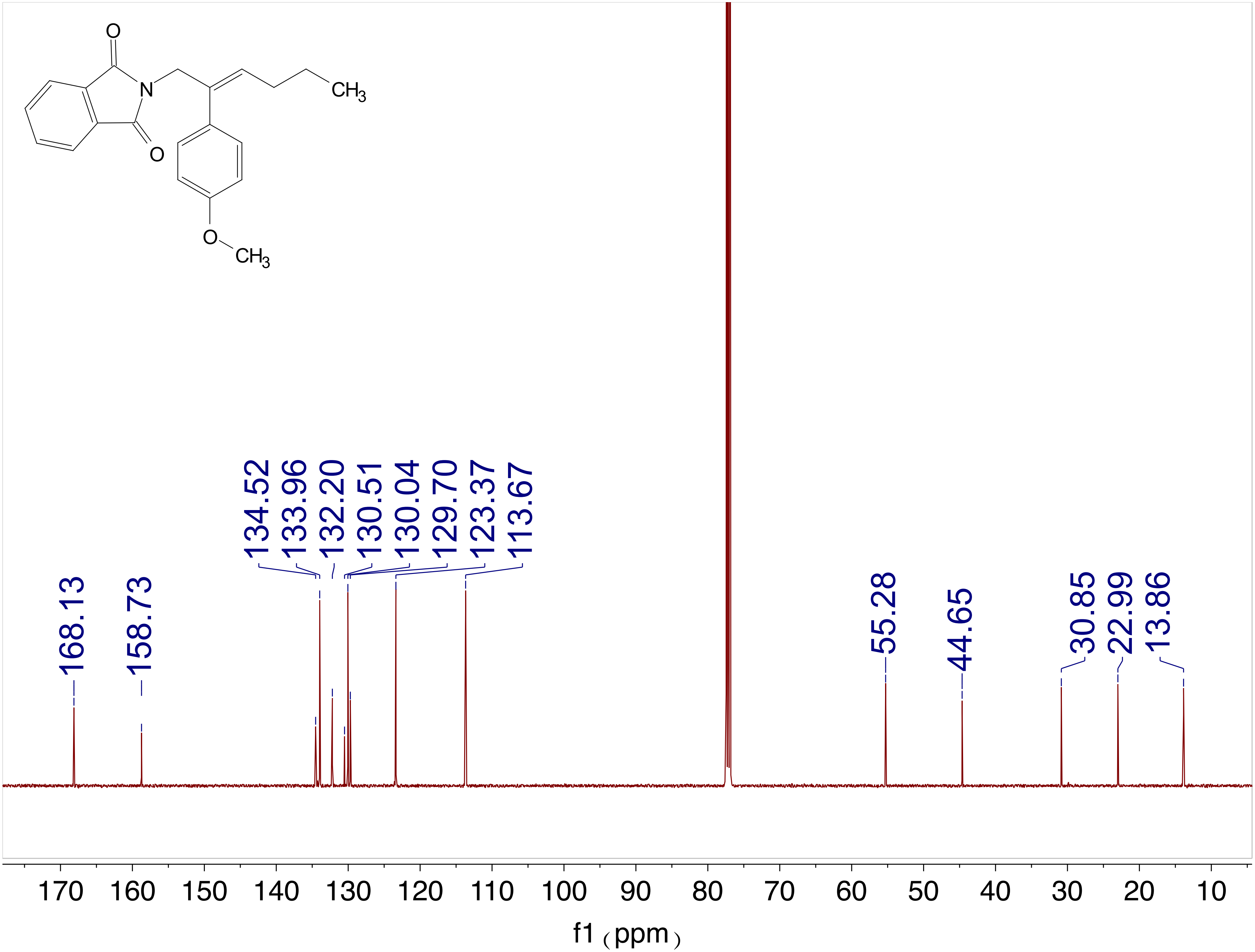
O
N
O
CH3
O
CH3
168.13
158.73
134.52
133.96
132.20
130.51
130.04
129.70
123.37
113.67
55.28
44.65
30.85
22.99
13.86
170 160 150 140 130 120 110 100 90 80 70 60 50 40 30 20 10
f1 (ppm)

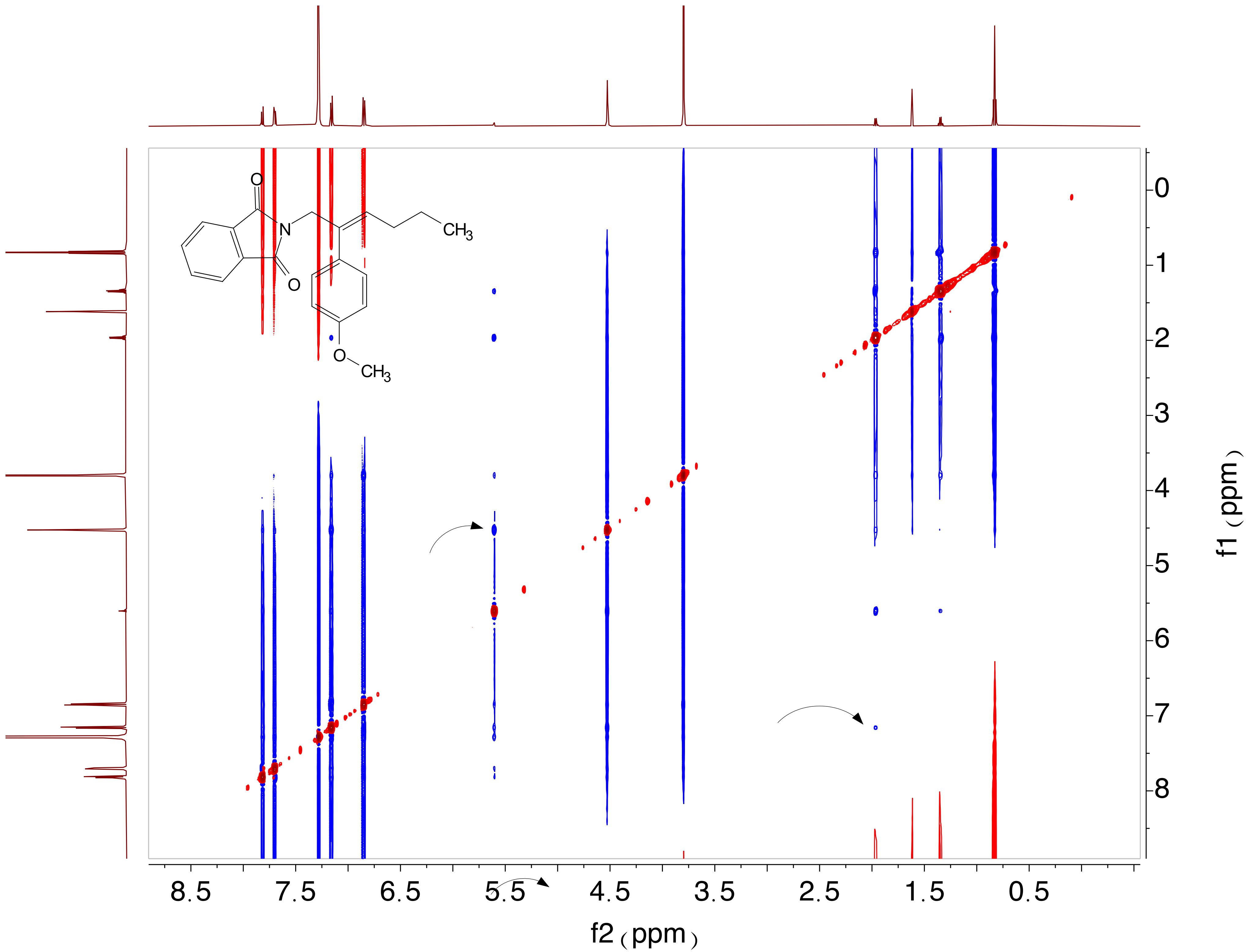

O
N
O
CH3
O
CH3
8.5
7.5
6.5
5.5
4.5
3.5
2.5
1.5
0.5
f2 (ppm)
0
1
2
3
4
5
6
7
8
f1 (ppm)

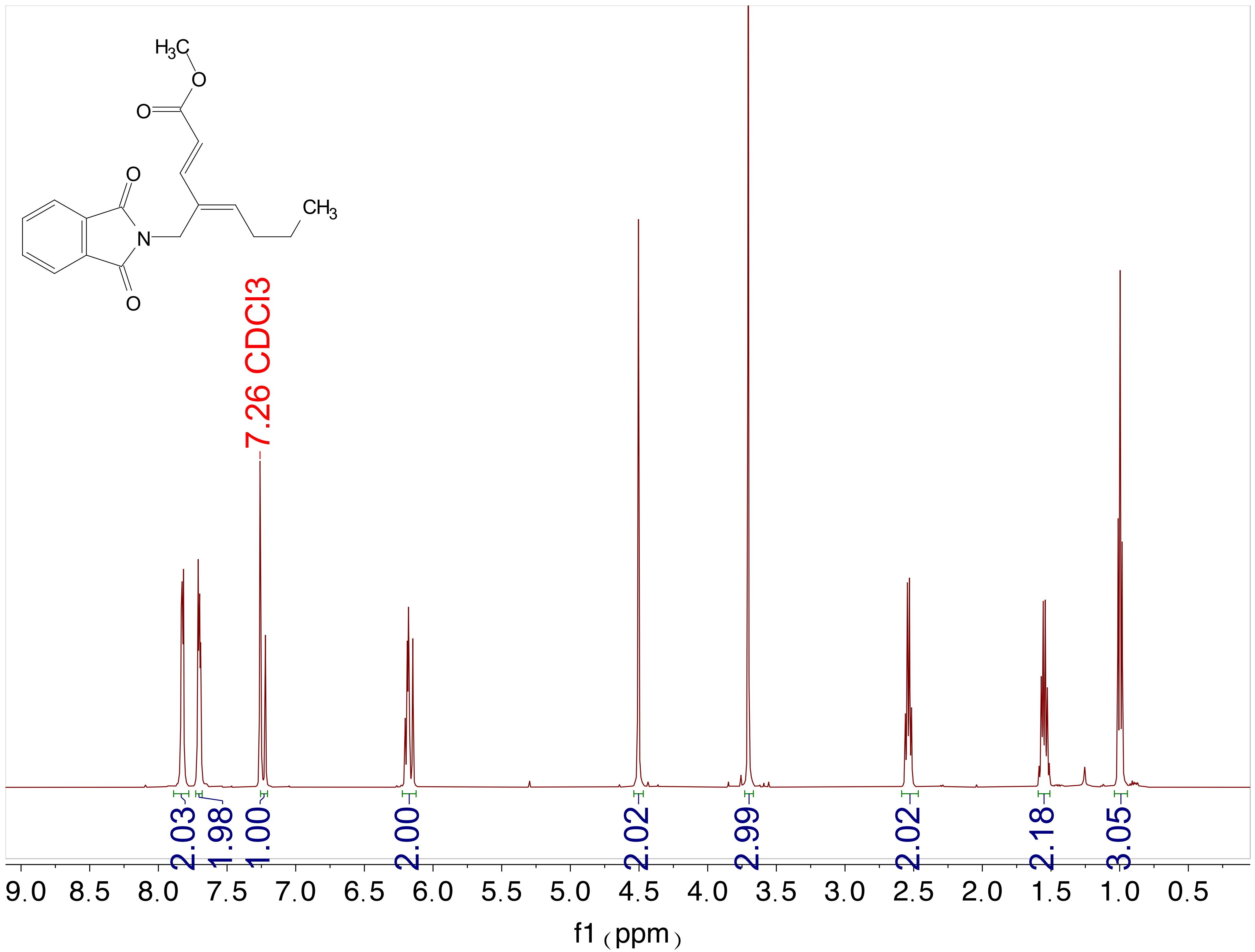

H3C
O
O
O
N
CH3
O
7.26 CDCl3
2.03
1.98
1.00
2.00
2.02
2.99
2.02
2.18
3.05
9.0 8.5 8.0 7.5 7.0 6.5 6.0 5.5 5.0 4.5 4.0 3.5 3.0 2.5 2.0 1.5 1.0 0.5
f1（ppm）

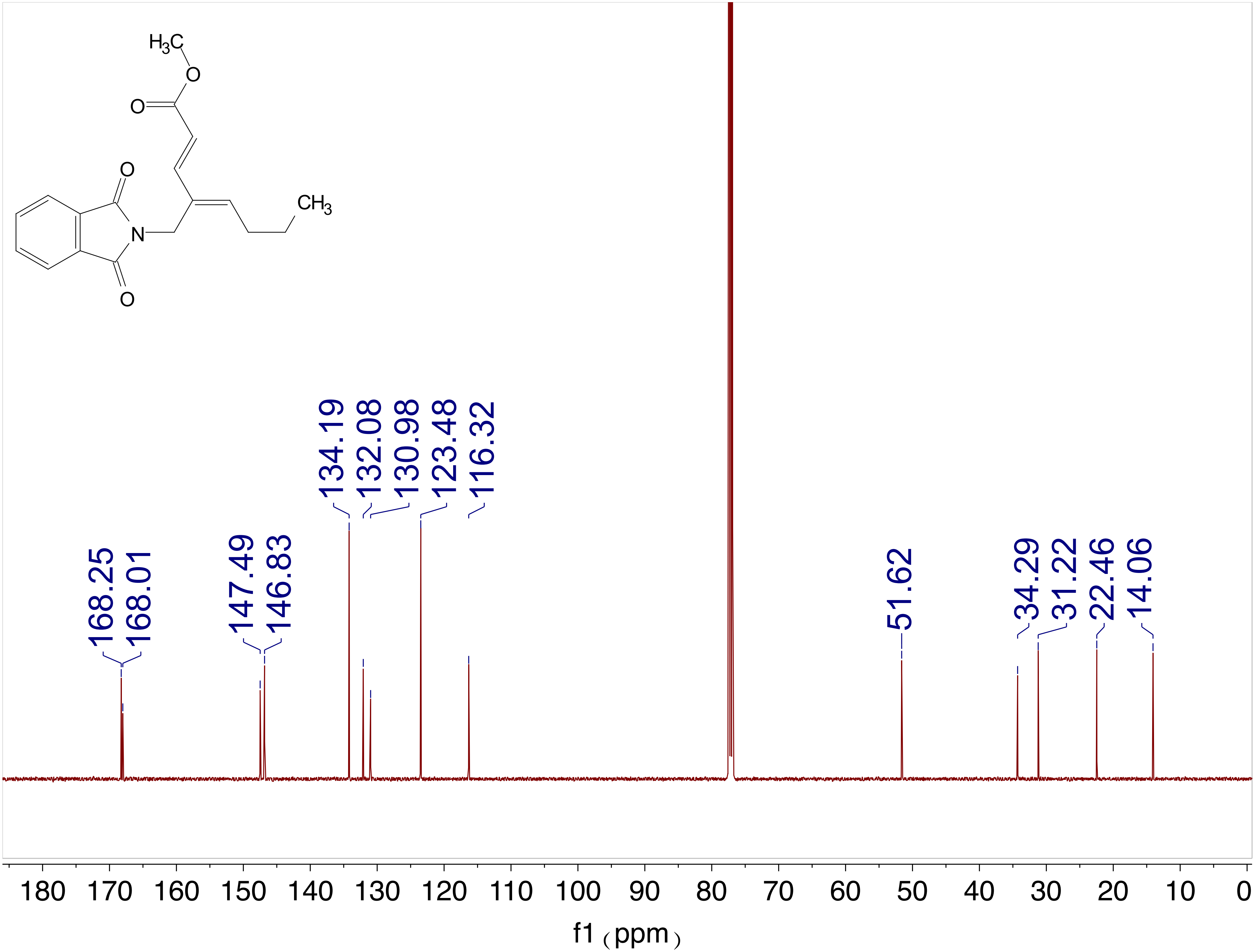

H3C
O
O
O
N
O
CH3
168.25
168.01
147.49
146.83
134.19
132.08
130.98
123.48
116.32
51.62
34.29
31.22
22.46
14.06
180 170 160 150 140 130 120 110 100 90 80 70 60 50 40 30 20 10 0
f1 (ppm)

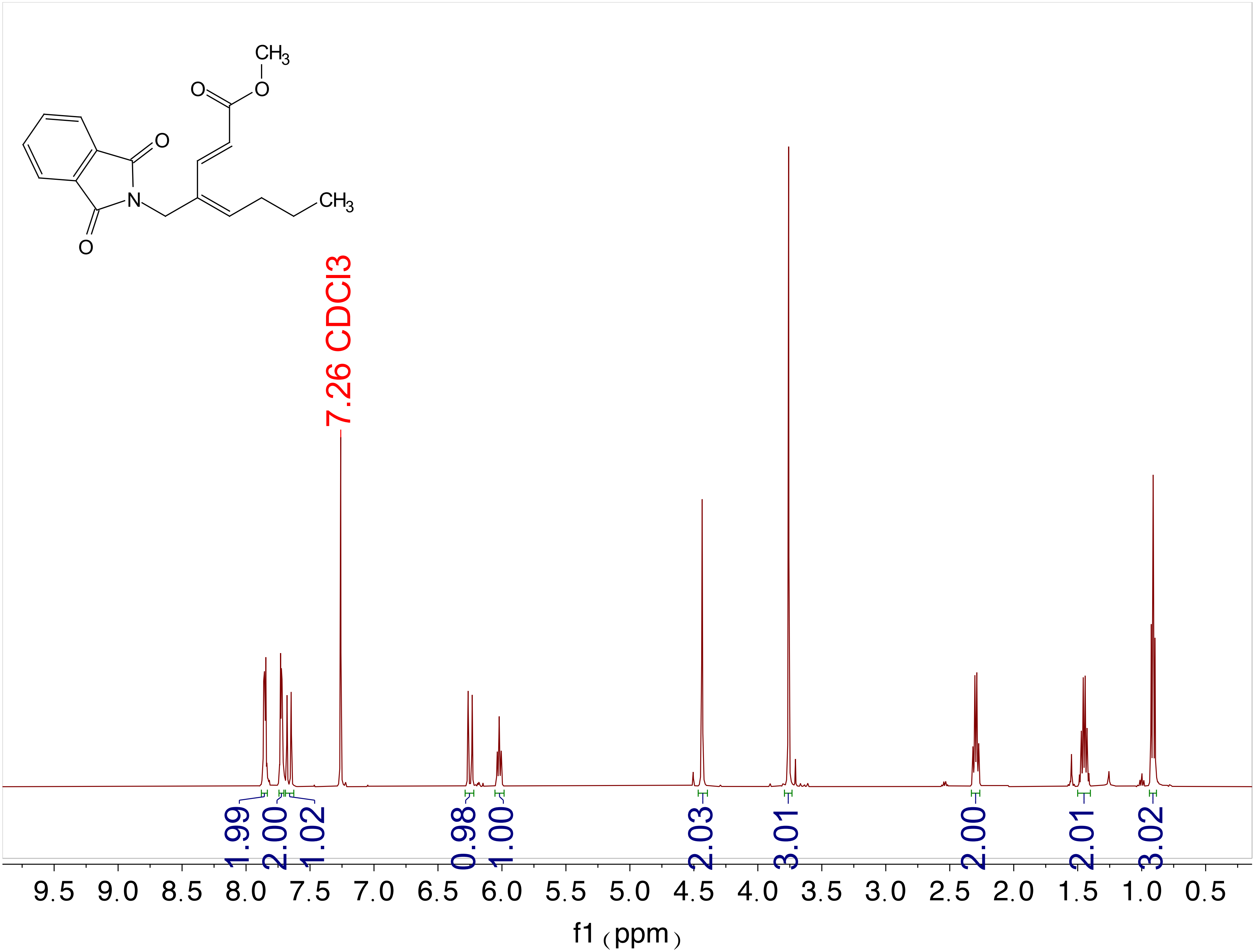

CH3
O
O
O
O
N
CH3
7.26 CDCl3
1.99
2.00
1.02
0.98
1.00
2.03
3.01
2.00
2.01
3.02
9.5 9.0 8.5 8.0 7.5 7.0 6.5 6.0 5.5 5.0 4.5 4.0 3.5 3.0 2.5 2.0 1.5 1.0 0.5
f1 (ppm)

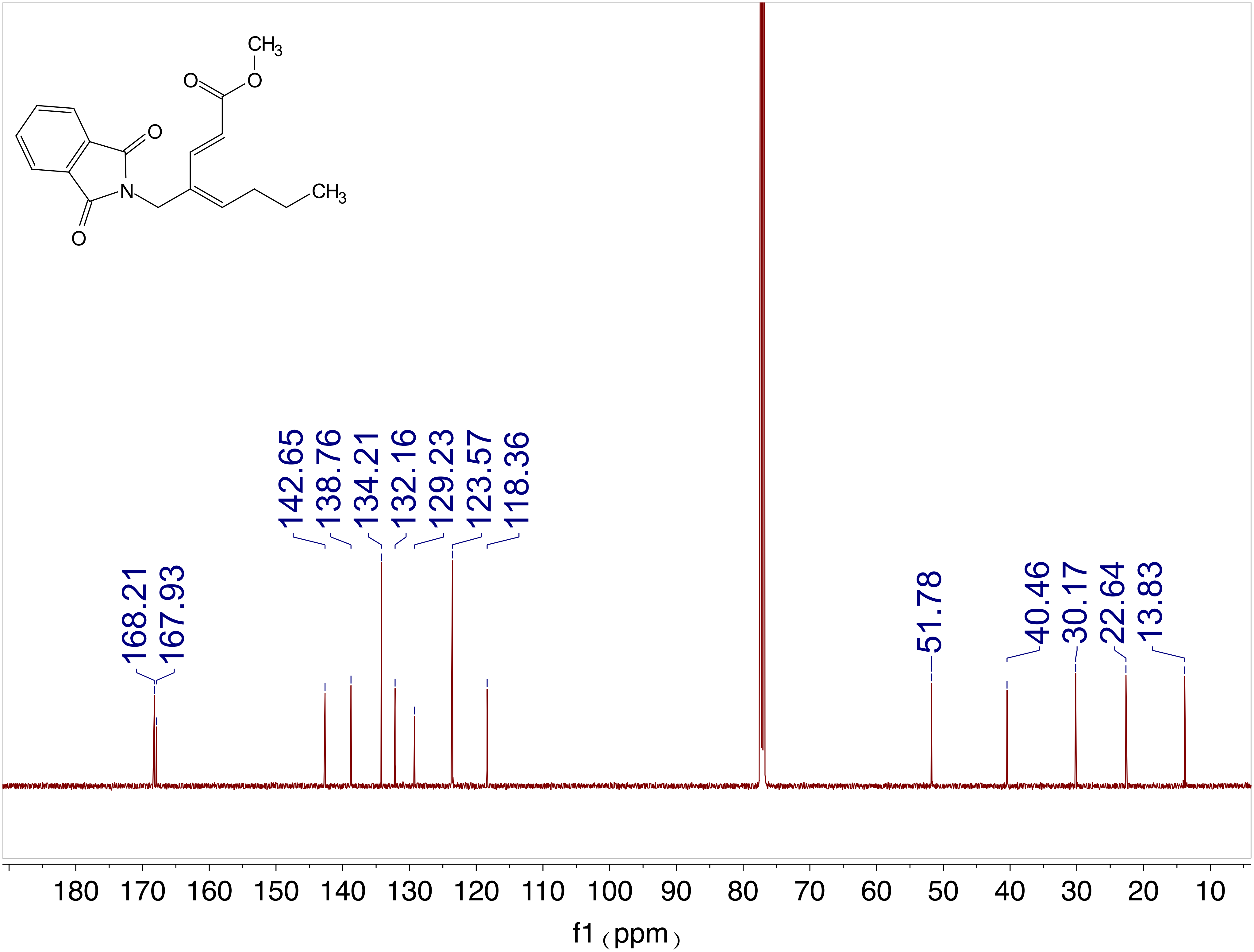

CH3
O
O
O
N
O
CH3
168.21
167.93
142.65
138.76
134.21
132.16
129.23
123.57
118.36
51.78
40.46
30.17
22.64
13.83
180 170 160 150 140 130 120 110 100 90 80 70 60 50 40 30 20 10
f1 (ppm)

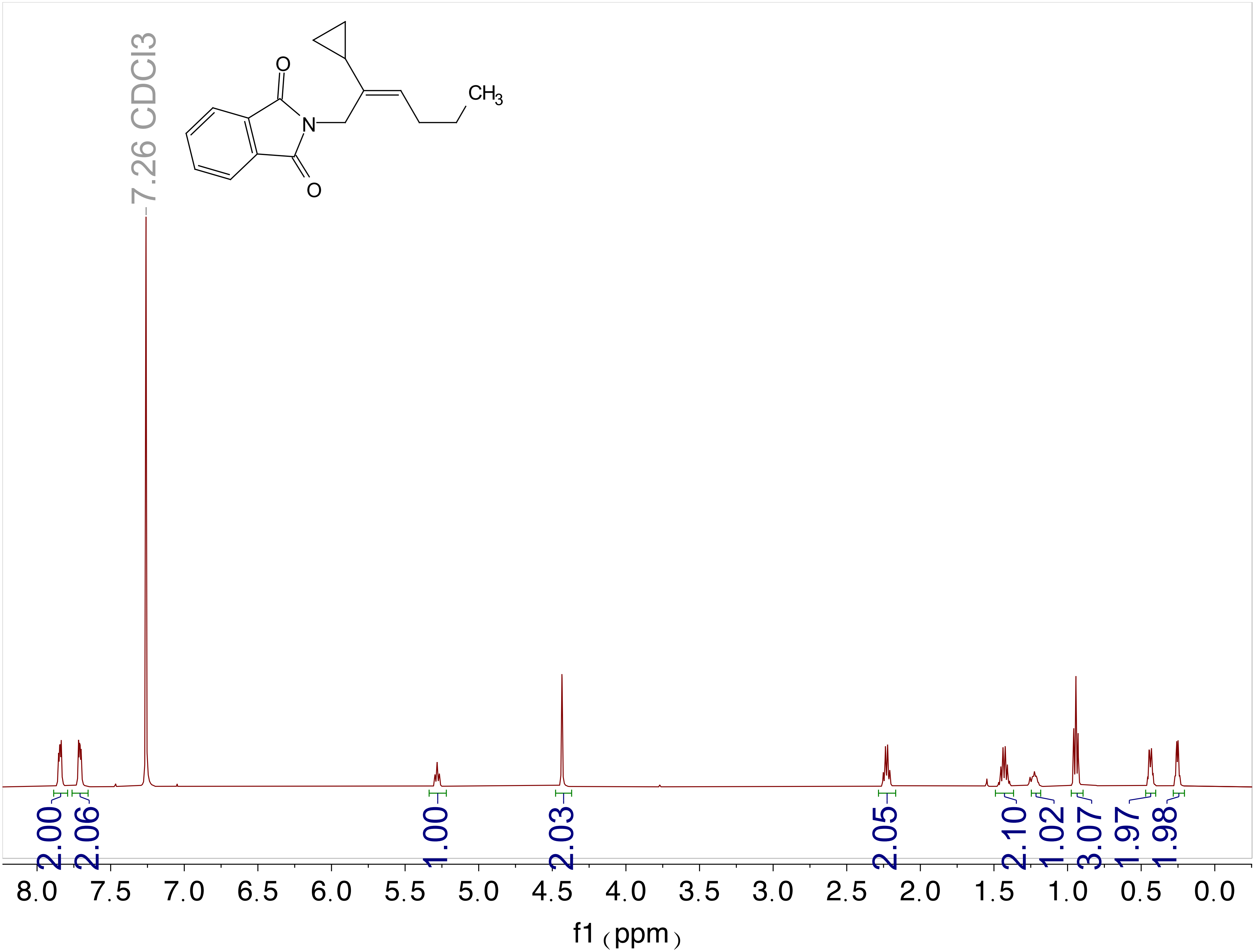

7.26 CDCl3
O
N
O
CH3
2.00
2.06
1.00
2.03
2.05
2.10
1.02
3.07
1.97
1.98
8.0 7.5 7.0 6.5 6.0 5.5 5.0 4.5 4.0 3.5 3.0 2.5 2.0 1.5 1.0 0.5 0.0
f1 (ppm)

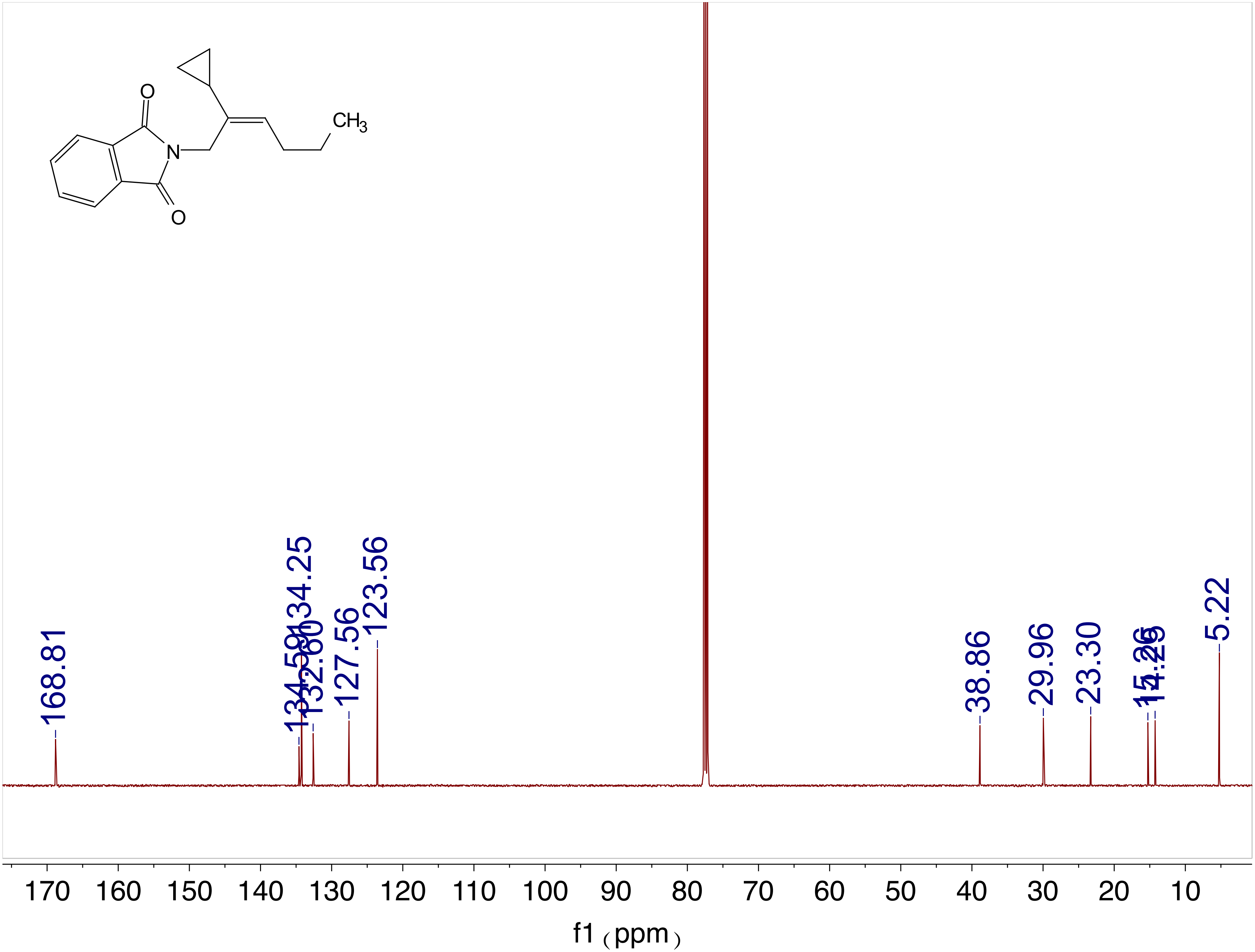
O
O
N
CH3
168.81
134.25
132.60
127.56
123.56
38.86
29.96
23.30
5.22
170 160 150 140 130 120 110 100 90 80 70 60 50 40 30 20 10
f1 (ppm)

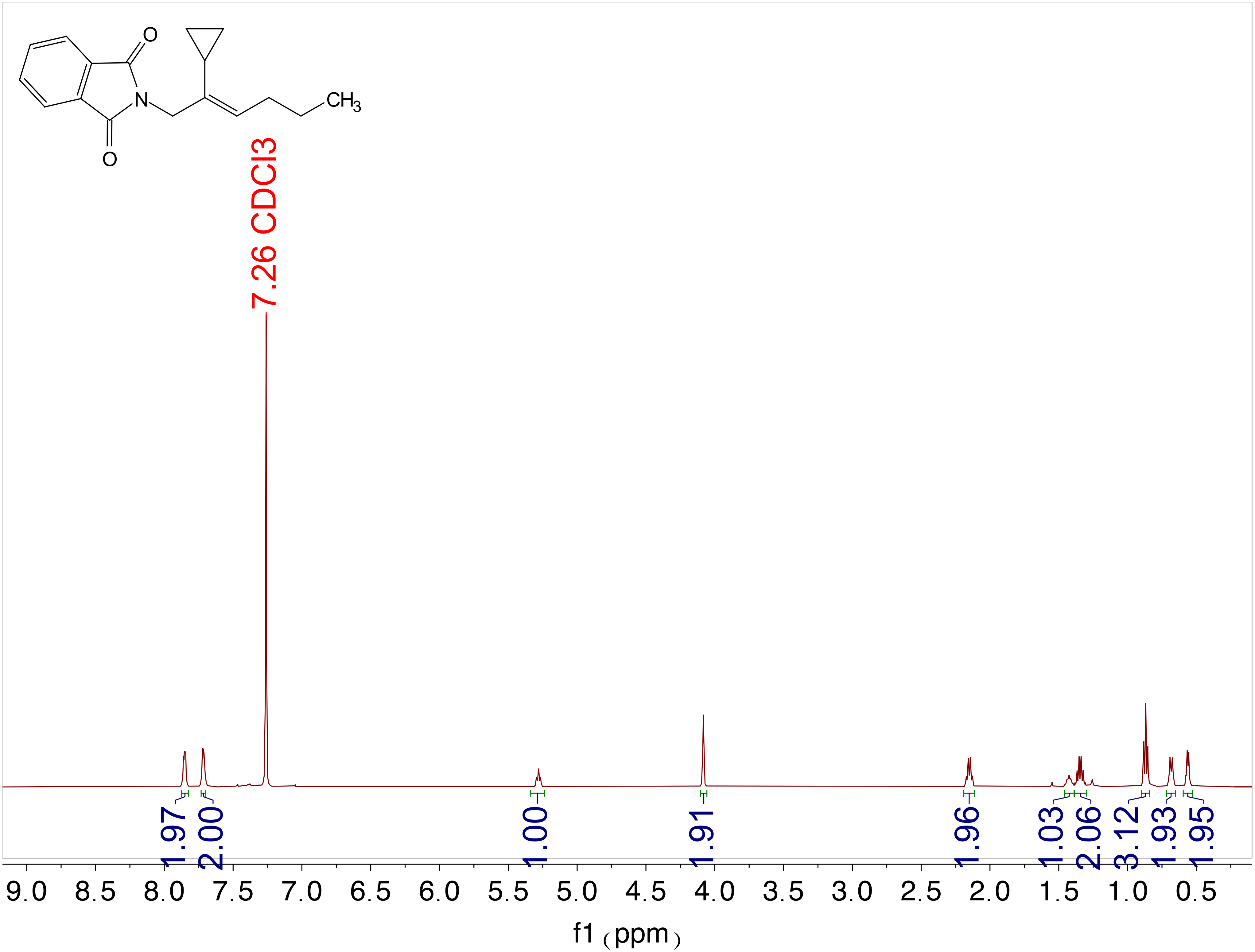
O
N
O
CH3
7.26 CDCl3
1.97
2.00
1.00
1.91
1.96
1.03
2.06
3.12
1.93
1.95
9.0
8.5
8.0
7.5
7.0
6.5
6.0
5.5
5.0
4.5
4.0
3.5
3.0
2.5
2.0
1.5
1.0
0.5
f1 (ppm)

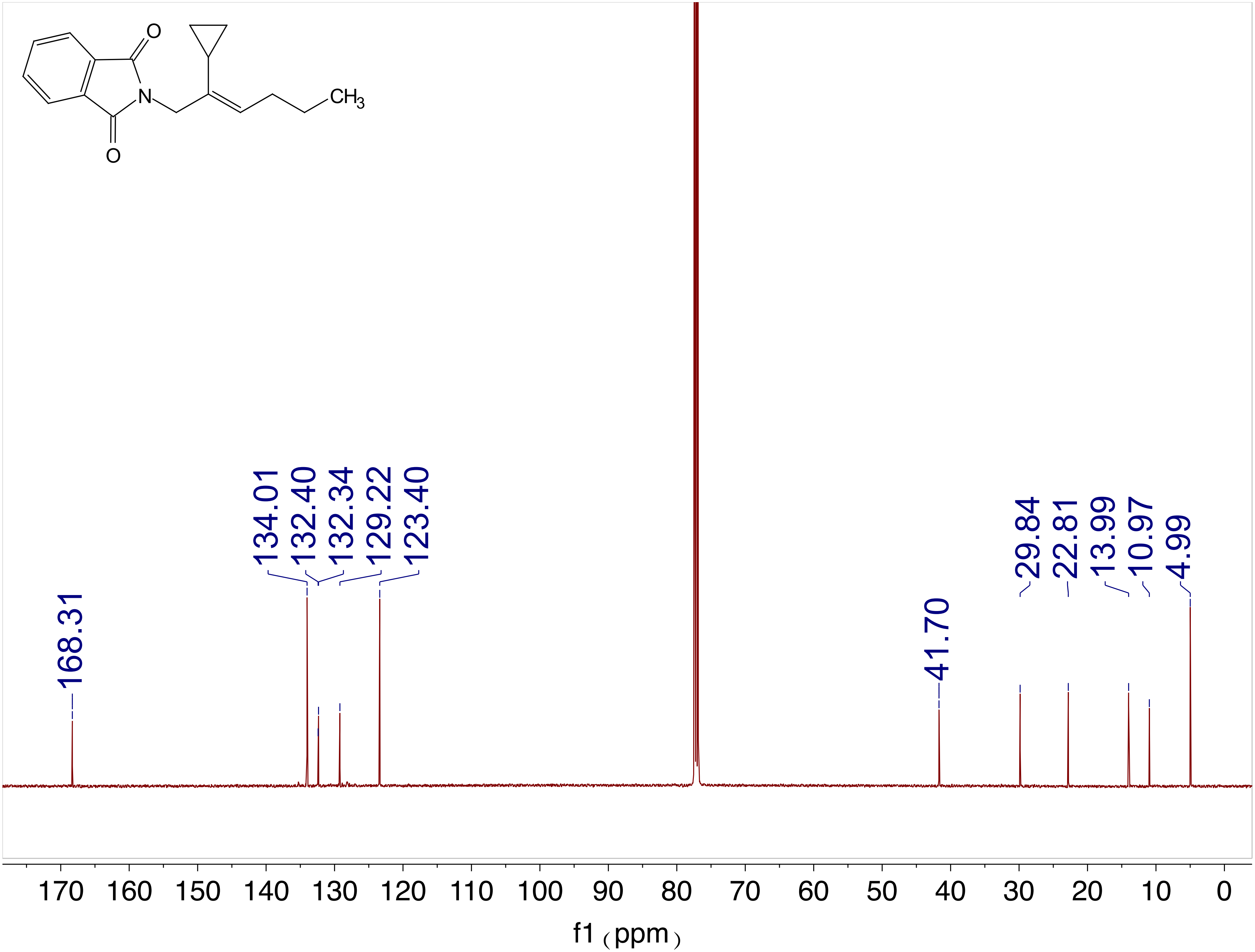

O
O
N
CH3
168.31
134.01
132.40
132.34
129.22
123.40
41.70
29.84
22.81
13.99
10.97
4.99
170 160 150 140 130 120 110 100 90 80 70 60 50 40 30 20 10 0
f1 (ppm)
